# Solid‐State Emissive Aroyl‐*S*,*N*‐Ketene Acetals with Tunable Aggregation‐Induced Emission Characteristics

**DOI:** 10.1002/anie.201916396

**Published:** 2020-03-10

**Authors:** Lukas Biesen, Nithiya Nirmalananthan‐Budau, Katrin Hoffmann, Ute Resch‐Genger, Thomas J. J. Müller

**Affiliations:** ^1^ Institut für Organische Chemie und Makromolekulare Chemie Heinrich-Heine-Universität Düsseldorf Universitätsstrasse 1 40225 Düsseldorf Germany; ^2^ Division Biophotonics Bundesanstalt für Materialforschung und -prüfung (BAM) Department 1 Richard-Willstätter-Strasse 11 12489 Berlin Germany; ^3^ Institut für Chemie und Biochemie Freie Universität Berlin Takustrasse 3 14195 Berlin Germany

**Keywords:** aggregation-induced emission, aroyl-*S*,*N*-ketene acetals, fluorescence, photophysical properties, solid-state emission

## Abstract

*N*‐Benzyl aroyl‐*S*,*N*‐ketene acetals can be readily synthesized by condensation of aroyl chlorides and *N*‐benzyl 2‐methyl benzothiazolium salts in good to excellent yields, yielding a library of 35 chromophores with bright solid‐state emission and aggregation‐induced emission characteristics. Varying the substituent from electron‐donating to electron‐withdrawing enables the tuning of the solid‐state emission color from deep blue to red.

## Graphical abstract

**A diverse library** of 35 *N*‐benzyl aroyl*S*,*N*‐ketene acetals with tunable color of the solid‐state emission and aggregation‐induced emission characteristics is readily synthesized by condensation of aroyl chlorides and *N*‐benzyl 2‐methyl benzothiazolium salts.

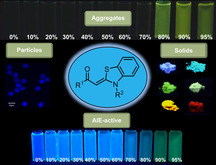

Many otherwise promising fluorophores with attractive photophysical properties, such as cyanine or xanthene dyes, suffer from dye–dye interactions resulting in aggregation‐caused quenching (ACQ), and commonly the emission of most chromophores is significantly quenched in the solid state.^1^ In 2001, Tang observed the opposite phenomenon of ACQ for the dye 1‐methyl‐1,2,3,4,5‐pentaphenylsilole and introduced the term aggregation‐induced emission (AIE). AIE dyes are typically non‐ or barely emissive in dilute solution but show a strong emission upon aggregation.^2^ Although the AIE mechanism is not yet completely understood, the occurrence of emission upon dye aggregation is commonly rationalized in terms of a restriction of intramolecular motions (RIM).^3^

Since Tang's discovery, AIE‐active compounds gained considerable interest owing to their enormous application potential.^4^ For instance, AIE‐active chromophores have been employed as reporters for mitochondria‐targeted cancer therapy, cancer ablation,^5^ and as nanoprobes for tumor‐targeted bioimaging or as biosensors.^6^ Fluorophores showing AIE are promising candidates for novel signal enhancement strategies in assays or improved nanoparticle (NP)‐based bioimaging approaches as such dyes can enable higher dye loading densities than conventional ACQ dyes.6c, 6d Besides bioanalytical applications1c AIE chromophores have also been successfully applied in optoelectronic devices.^7^ For example, for applications such as organic light‐emitting diodes (OLEDs), there is a continuous interest in luminophores revealing solid‐state emission.4c, 4d Many AIE systems utilize tetraphenylethene (TPE) and its derivatives6c and there are also some reports of anthracene or carbazole based structures.1c

In recent years we could demonstrate that also quite polar heterocyclic systems, such as indolone merocyanines and quinoxalines motifs,^8^, ^9^, ^10^ can be AIE active.6d, 11 We therefore became interested in molecules with pronounced charge‐transfer (CT) character, which could finally provide access to blue emissive AIE chromophores. We reasoned that the aroyl *S*,*N*‐ketene acetals could be interesting candidates with AIE characteristics tunable by variation of the substitution pattern. While aroyl‐*S*,*N*‐ketene acetals are known as building blocks for enlarged heterocycles^12^ and benzothiazole motifs have been previously utilized in AIE systems,^13^ *N*‐benzyl aroyl‐*S*,*N*‐ketene acetals have not been yet studied with respect to their synthesis, photophysical properties and AIE behavior. Only the trifluoromethyl‐substituted aroyl‐*S*,*N*‐ketene acetals **3 z** and **3 aa** were synthesized before and used as acid precursors and radical initiators, but have not been spectroscopically studied.^14^ Herein, we report the highly diverse, rapid modular synthesis of *N*‐benzyl aroyl *S*,*N*‐ketene acetals and their emission characteristics, with special emphasis dedicated to their AIE behavior.

Aroyl‐*S*,*N*‐ketene acetals **3** were synthesized as a library of 35 compounds in a straightforward condensation by base mediated addition–elimination of benzothiazolium salts **2** and acid chlorides **1** in moderate to excellent yields (Scheme 1, Table 1).

**Scheme 1:**
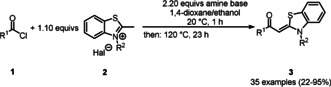
Synthesis of aroyl‐*S*,*N*‐ketene acetals **3**.

**Table 1:** Condensation synthesis of aroyl‐*S*,*N*‐ketene acetals **3**.^[a]^

Entry	R^1^	R^2^	Product (yield)^[b]^
1	4‐Me_2_NC_6_H_4_	4‐BrC_6_H_4_CH_2_	**3 a** (68 %)
2	4‐Me_2_NC_6_H_4_	PhCH_2_	**3 b** (72 %)
3	4‐MeOC_6_H_4_	4‐BrC_6_H_4_CH_2_	**3 c** (69 %)
4	4‐MeOC_6_H_4_	PhCH_2_	**3 d** (52 %)
5	4‐*t*BuC_6_H_4_	4‐BrC_6_H_4_CH_2_	**3 e** (48 %)
6	4‐*t*BuC_6_H_4_	PhCH_2_	**3 f** (65 %)
7	4‐MeC_6_H_4_	4‐BrC_6_H_4_CH_2_	**3 g** (80 %)
8	4‐MeC_6_H_4_	PhCH_2_	**3 h** (65 %)
9	Ph	4‐BrC_6_H_4_CH_2_	**3 i** (58 %)
10	Ph	PhCH_2_	**3 j** (72 %)
11	Ph	Me	**3 k** (94 %)
12	*trans*‐4‐FC_6_H_4_CH=CH	4‐BrC_6_H_4_CH_2_	**3 l** (22 %)
13	*trans*‐4‐FC_6_H_4_CH=CH	PhCH_2_	**3 m** (31 %)
14	4‐FC_6_H_4_	4‐BrC_6_H_4_CH_2_	**3 n** (69 %)
15	4‐FC_6_H_4_	PhCH_2_	**3 o** (85 %)
16	3‐FC_6_H_4_	4‐BrC_6_H_4_CH_2_	**3 p** (65 %)
17	2‐FC_6_H_4_	PhCH_2_	**3 q** (49 %)
18	4‐ClC_6_H_4_	4‐BrC_6_H_4_CH_2_	**3 r** (83 %)
19	4‐ClC_6_H_4_	PhCH_2_	**3 s** (88 %)
20	6‐Cl‐pyridine‐3‐yl	4‐BrC_6_H_4_CH_2_	**3 t** (27 %)
21	6‐Cl‐pyridine‐3‐yl	PhCH_2_	**3 u** (27 %)
22	4‐BrC_6_H_4_	4‐BrC_6_H_4_CH_2_	**3 v** (55 %)
23	4‐BrC_6_H_4_	PhCH_2_	**3 w** (88 %)
24	4‐IC_6_H_4_	4‐BrC_6_H_4_CH_2_	**3 x** (59 %)
25	4‐IC_6_H_4_	PhCH_2_	**3 y** (65 %)
26	4‐F_3_CC_6_H_4_	4‐BrC_6_H_4_CH_2_	**3 z** (72 %)
27	4‐F_3_CC_6_H_4_	PhCH_2_	**3 aa** (50 %)
28	4‐NCC_6_H_4_	4‐BrC_6_H_4_CH_2_	**3 ab** (51 %)
29	4‐NCC_6_H_4_	PhCH_2_	**3 ac** (66 %)
30	4‐O_2_NC_6_H_4_	4‐BrC_6_H_4_CH_2_	**3 ad** (44 %)
31	4‐O_2_NC_6_H_4_	PhCH_2_	**3 ae** (73 %)
32	thiophen‐2‐yl	4‐BrC_6_H_4_CH_2_	**3 af** (72 %)
33	thiophen‐2‐yl	PhCH_2_	**3 ag** (87 %)
34	furan‐2‐yl	4‐BrC_6_H_4_CH_2_	**3 ah** (77 %)
35	furan‐2‐yl	PhCH_2_	**3 ai** (95 %)

[a] All reactions were performed on 1.0 mmol scale: **1** (1.0 mmol), **2** (1.1 mmol), amine base (2.2 mmol) in 1,4‐dioxanes/ethanol 5:2 (7.0 mL) were stirred at room temp for 1 h and at 120 °C for 23 h. [b] Yields after flash chromatography on silica gel.

The condensations were performed in a mixture of 1,4‐dioxane and ethanol with triethylamine as a base, except for the compounds **3 a** and **3 b** (Table 1, entries 1 and 2), where diisopropylethylamine in 1,4‐dioxane was used. Acid chloride **2** can bear electron‐donating and electron‐withdrawing substituents in *ortho*‐, *meta*‐, and *para*‐position. In addition, heterocyclic acid chlorides, such as thiophenoyl, furoyl and pyridinoyl chlorides are also applicable in this method (for details, see Supporting Information, Table S4). For preparing our spectroscopic reference chromophores **3** besides *N*‐benzyl 2‐methylthiazolium bromides **2 a** and **2 b**, 2,3‐dimethylthiazolium iodide (**2 c**) was also successfully transformed.

The absorption maxima of the investigated series of aroyl‐*S*,*N*‐ketene acetals **3** cover a narrow wavelength region and vary between 378 and 413 nm (Table S2 and Figure S1). TD‐DFT calculations (B3LYP/6‐31G**, applying PCM on 17 selected chromophores **3** with ethanol as the solvent as implemented in Gaussian 09^15^) reveal a good agreement of the longest wavelength absorption maxima of the calculated and experimentally obtained spectra of the dye solution (Table S9). Essentially the absorption maxima can be assigned to HOMO–LUMO transitions with considerable CT character, typical for merocyanines. Therefore, for further rational design of this type of chromophore quantum chemical calculations can be favorably applied as a tool for property prediction.

Aroyl‐*S*,*N*‐ketene acetals **3** neither luminesce in dilute ethanol solutions nor in other organic solvents except for the dimethylamino‐substituted derivatives **3 a** and **3 b**. However, all aroyl‐*S*,*N*‐ketene acetals **3** are emissive in the solid state. Moreover, their emission color varies considerably depending on the electronic nature of the substituents in *para*‐position of the aroyl moiety. Consequently, they can be a readily distinguished by their emission color (Figure 1, top).

**Figure 1:**
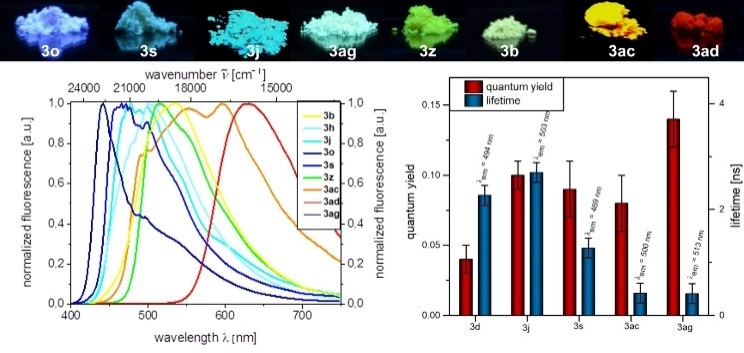
Top: Solid‐state fluorescence of selected aroyl‐*S*,*N*‐ketene acetals **3** (*λ*_exc_=365 nm, UV lamp) revealing emission color tuning by substitution pattern. Bottom, left: normalized solid‐state emission spectra obtained with a calibrated fluorometer. Bottom, right: absolutely measured fluorescence quantum yields and fluorescence lifetime of selected aroyl‐*S*,*N*‐ketene acetals **3** (*λ*_exc_=*λ*_abs,max_ at *T*=298 K).

Within the dye series studied, *para*‐fluoro substituted derivative **3 o** reveals the shortest wavelength emission maximum *λ*_em_ (*λ*_em_=442 nm) while *para*‐nitro substituted compound **3 ad** shows the most red‐shifted emission maximum (*λ*_em_=630 nm; for further details, see Table S2 and Figure S2). The results of the fluorescence quantum yield (*Φ*_f_) and fluorescence lifetime (*τ*) measurements are displayed in the right panel of Figure 1. In the solid state, dye **3 ag** shows the highest *Φ*_f_ of 0.14, but the shortest *τ* of 0.411 ns of this series, while dye **3 d** reveals the lowest *Φ*_f_ of 0.04 and shows nevertheless a relatively long *τ* of 2.27 ns. These features suggest that the rotational motions are restricted in the solid state. The sometimes inconsistent trends of *Φ*_f_ and *τ* point to different processes determining the photophysics of these dyes. The *Φ*_f_ values of **3 a** and **3 b** which are also emissive in solution were determined to *Φ*_f_=0.07 in ethanol for both dyes relative to coumarin 343 (*Φ*_f_=0.63 in ethanol).^16^ The dimethylamino substituted derivatives **3 a** and **3 b** display a weak positive solvatochromism (for further details, see Tables S2 and S6, Figures S2–S6 and S51,S52).

The absence of fluorescence in solution in conjunction with the observation of strong solid‐state emission encouraged us to perform AIE studies with our dyes. Aroyl‐*S*,*N*‐ketene acetals **3** are soluble in common, polar organic solvents, such as acetonitrile, THF, and ethanol, but they are insoluble in water. Samples of aroyl‐*S*,*N*‐ketene acetals were hence diluted in different mixtures of organic solvents and water with ratios varying from 0 to 99 %. The most intriguing results were obtained with ethanol/water mixtures which are subsequently discussed in detail.

Upon increasing the water content, the solubility of the hydrophobic dyes are significantly reduced favoring the formation of dye aggregates. Up to a water fraction of 70 % the aroyl‐*S*,*N*‐ketene acetals did at maximum fluoresce very weakly (*Φ*_f_<0.01). When increasing the water content above 80 %, compounds **3** started to aggregate, resulting in a considerable enhancement in *Φ*_f_ and in an increase in *τ* (see Figure 2). These effects are ascribed to the blocking of non‐radiative pathways depopulating the excited singlet state by an aggregation‐induced RIM.^15^ For most dyes the fluorescence intensity decreased again at water fractions above 90 % as the compounds precipitated. The hydrodynamic diameters of the resulting dye aggregates were determined to be between 160 and 425 nm for the dyes **3 d**, **3 j**, **3 s**, **3 ac**, and **3 ag** with dynamic light scattering. This confirms that aroyl‐*S*,*N*‐ketene acetals represent a novel class of AIE chromophores with substitution pattern control of the AIE behavior (Figure 2). However, while each derivative emits in the solid state, irrespective of the substituent on the nitrogen of the benzothiazole moiety, this substituent clearly affects the AIE behavior. For example, the *N*‐methyl derivative **3 k** does not fluoresce upon aggregation at the dye concentration used for all the dyes in our AIE studies. This demonstrates that the benzylic substituent of the aroyl‐*S*,*N*‐ketene acetals **3** is responsible for the occurrence of AIE characteristics (Figure 2). The most significant aggregation‐induced increase in fluorescence intensity was found for *para*‐methyl substituted dye **3 g** and the *para*‐fluoro substituted chromophore **3 o** that both reveal a 20‐fold fluorescence enhancement upon dye aggregation.

**Figure 2:**
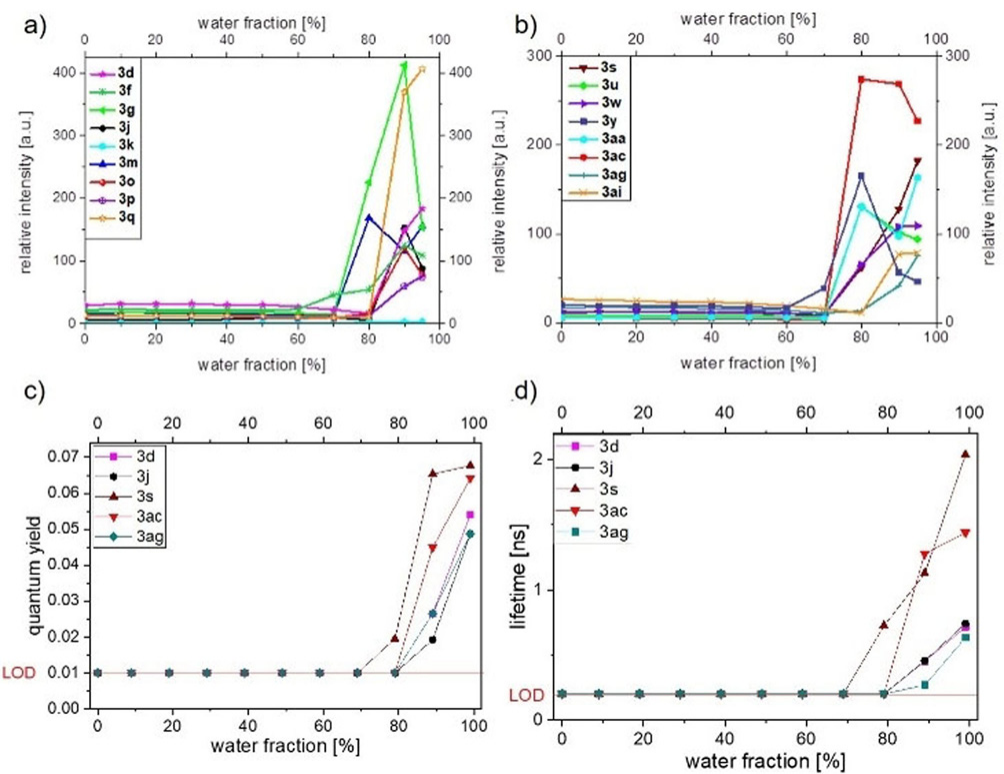
Emission intensity (a,b), *Φ*_f_ (c), and fluorescence lifetime (d) of selected aroyl‐*S*,*N*‐ketene acetals **3** as a function of the water fraction of the ethanol/water mixtures. The measurements were performed at *T*=298 K with a dye concentration *c*(**3**)=10^−7^ m, the fluorescence was excited at the respective absorption maximum *λ*_exc_=*λ*_max_).

To highlight the fluorescence properties of this class of AIE chromophores, exemplarily the emission spectra and visual evolution of the fluorescence are shown in Figure 3 for compound **3 ac** in ethanol/water mixtures with steadily increasing water content. At a water content of 89 % compound **3 ac** starts to become emissive. The maximum fluorescence is observed at a water content of 99 %. Furthermore, *Φ*_f_ increased to 0.06 and *τ* to 1.4 ns (for further details, see Supporting Information, Section S6).

**Figure 3:**
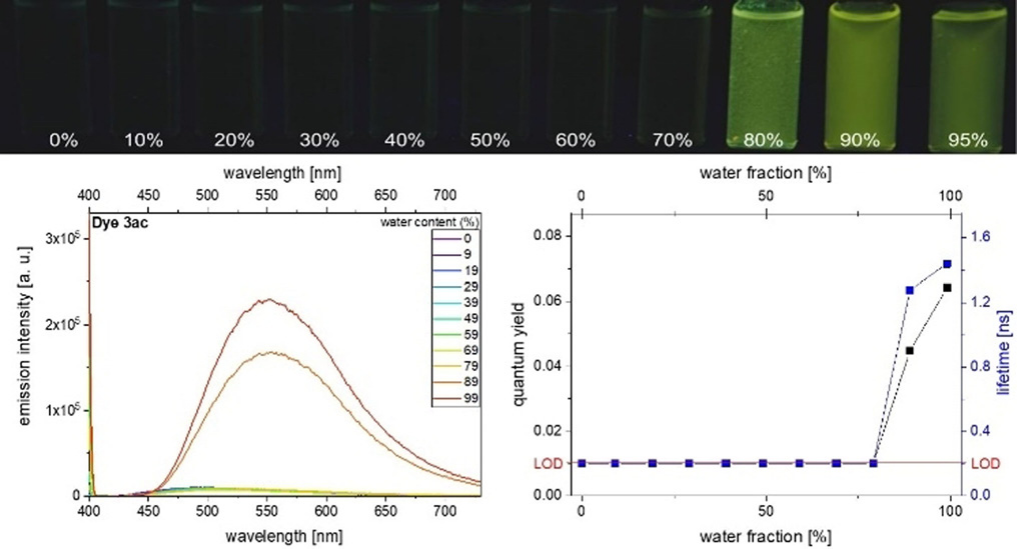
Top: visual impression of the AIE features of **3 ac** in ethanol/water mixtures of increasing water content upon excitation with a UV‐lamp (*λ*_exc_=365 nm). Bottom, left: emission spectra of **3 ac** obtained at different water fractions of the ethanol/water solvent mixtures. Bottom, right: corresponding *Φ*_f_ data (recorded at *T*=298 K, *c*(**3 ac**)=10^−7^ m, *λ*_exc_=*λ*_abs,max_).

Interestingly, the dimethylamino‐substituted dyes **3 a** and **3 b** represent exceptions from the typical AIE behavior of aroyl‐*S*,*N*‐ketene acetals as they fluoresce in ethanol solution and exhibit ACQ upon aggregation. The continuous red‐shift from 461 to 500 nm in emission introduced by the increasing water content suggests polarity‐induced quenching with increasing CT character of the dyes (see Section S6).

The intense solid‐state fluorescence of the dyes **3** together with their AIE behavior encouraged us to study their fluorescence properties when encapsulated in an nonpolar solid matrix, such as polystyrene, to underline the potential of AIE emitters for staining applications. For this purpose, dyes **3 d**, **3 j**, **3 s**, **3 ac**, and **3 ag** were incorporated into 8 μm‐sized carboxy‐functionalized polystyrene particles (PSP) from Kisker using a straightforward swelling procedure from Behnke et al.^17^ This encapsulation is expected to sterically restrict or at least reduce intramolecular rotations of the PSP‐entrapped dye molecules. Additionally, a high encapsulation concentration of the dyes (6 mm) was chosen to encourage dye‐dye interactions in PSP. Microscopic studies of single dye‐stained particles confirm the homogeneous dye staining of the PSP and a slight change in particle shape (Figure 4, right). As shown in Figure 4 (left panel), the fluorescence excitation and emission spectra of dye **3 ac** in PSP are hypsochromically shifted compared to the corresponding spectra of the dye in ethanol and dye aggregates in ethanol–water mixtures. This is ascribed to the reduced polarity of the dye molecules faced in the nonpolar polymer matrix.

**Figure 4:**
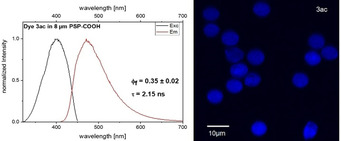
Top: Normalized fluorescence excitation and emission spectra (*λ*_exc_=404 nm) of dispersed 8 μm‐sized PSP loaded with dye **3 ac** (left) and CLSM image of the dye‐loaded PSP (right).

Moreover, PSP encapsulation leads to a significantly higher *Φ*_f_ of 0.35 exceeding the *Φ*_f_ values observed for this dye in ethanol, ethanol–water mixtures, and in the solid state. This trend is also reflected by a prolonged *τ* of 2.15 ns. The fluorescence features of dyes **3 d**, **3 j**, **3 s**, and **3 ag** shown in the Supporting Information (see Figure S54 and Table S8) reveal similar trends.

In summary, a library of 35 compounds of a new class of aroyl‐*S*,*N*‐ketene acetals with tunable AIE characteristics could be readily synthesized in good to excellent yields and the spectroscopic properties of these dyes were studied in solution, in the solid state, and under conditions introducing dye aggregation. These results revealed substitution pattern control of the AIE effects. While the R^1^ substituent is essentially responsible for the color of the chromophores, *N*‐benzyl substitution of the benzothiazolidene donor is most crucial for the occurrence of AIE effects. These novel AIE chromophores are well suited for implementation in optoelectronic devices. Current work is directed to develop diversity oriented one‐pot syntheses for accessing π‐conjugation extended aroyl‐*S*,*N*‐ketene acetals with tunable AIE characteristics.

## Conflict of interest

The authors declare no conflict of interest.

## Supporting information

As a service to our authors and readers, this journal provides supporting information supplied by the authors. Such materials are peer reviewed and may be re‐organized for online delivery, but are not copy‐edited or typeset. Technical support issues arising from supporting information (other than missing files) should be addressed to the authors.

Supplementary
