# Supplementary material for: Solid‐State Emissive Aroyl‐S,N‐Ketene Acetals with Tunable Aggregation‐Induced Emission Characteristics
Source: Angew Chem Int Ed Engl. 2020 Mar 10;59(25):10037–41. doi: 10.1002/anie.201916396 (PMC7317214; doi:10.1002/anie.201916396)
Supplement: Supplementary file 1 — Supplementary [file ANIE-59-10037-s001.pdf]

Angewandte Chemie
Eine Zeitschrift der Gesellschaft Deutscher Chemiker

Supporting Information

Solid-State Emissive Aroyl-*S*,*N*-Ketene Acetals with Tunable Aggregation-Induced Emission Characteristics

*Lukas Biesen, Nithiya Nirmalananthan-Budau, Katrin Hoffmann, Ute Resch-Genger, and Thomas J. J. Müller**

anie_201916396_sm_miscellaneous_information.pdf

Supporting information

Table of contents

1 General considerations ... 3

2 Overview of synthesized aroyl-*S,N*-ketene acetals 3 ... 6

3 Overview of photophysical properties of aroyl-*S,N*-ketene acetals 3 ... 10

4 Syntheses ... 15

4.1 Synthesis of starting materials ... 15

4.1.1 General procedure (**GPI**) Synthesis of benzothiazoliumbromides **2**[3] ... 15

4.1.1.1 Spectroscopic data ... 16

3-Benzyl-2-methylbenzo[*d*]thiazol-3-iumbromide (**2a**)[3] ... 16

3-(4-Bromobenzyl)-2-methylbenzo[*d*]thiazol-3-iumbromide (**2b**) ... 17

2,3-Dimethylbenzo[*d*]thiazol-3-iumiodide (**2c**)[4] ... 18

4.2 Synthesis and analytical data of aroyl-*S,N*-ketene acetals 3 ... 19

4.2.1 General procedure (**GPII**) for the synthesis of aroyl-*S,N*-ketene acetals **3** ... 19

4.2.1.1 Spectroscopic data ... 25

(*Z*)-2-(3-(4-Bromobenzyl)benzo[*d*]thiazol-2(3*H*)-ylidene)-1-(4-((dimethylamino)phenyl) ethan-1-one (**3a**) ... 25

(*Z*)-2-(3-Benzyl)benzo[*d*]thiazol-2(3*H*)-ylidene)-1-(4-((dimethylamino)phenyl)ethan-1-one (**3b**) ... 26

(*Z*)-2-(3-(4-Bromobenzyl)benzo[*d*]thiazol-2(3*H*)-ylidene)-1-(4-(methoxyphenyl)ethan-1-one (**3c**) ... 27

(*Z*)-2-(3-Benzyl)benzo[*d*]thiazol-2(3*H*)-ylidene)-1-(4-(methoxyphenyl)ethan-1-one (**3d**) ... 28

(*Z*)-2-(3-(4-Bromobenzyl)benzo[*d*]thiazol-2(3*H*)-ylidene)-1-(4-*tert*-butylphenyl)ethan-1-one (**3e**) ... 29

(*Z*)-2-(3-Benzyl)benzo[*d*]thiazol-2(3*H*)-ylidene)-1-(4-*tert*-butylphenyl)ethan-1-one (**3f**) ... 30

(*Z*)-2-(3-(4-Bromobenzyl)benzo[*d*]thiazol-2(3*H*)-ylidene)-1-(*p*-tolyl)ethan-1-one (**3g**) ... 31

(*Z*)-2-(3-Benzyl)benzo[*d*]thiazol-2(3*H*)-ylidene)-1-(*p*-tolyl)ethan-1-one (**3h**) ... 32

(*Z*)-2-(3-(4-Bromobenzyl)benzo[*d*]thiazol-2(3*H*)-ylidene)-1-phenylethan-1-one (**3i**) ... 33

(*Z*)-2-(3-Benzylbenzo[*d*]thiazol-2(3*H*)-ylidene)-1-phenylethan-1-one (**3j**) ... 34

(*Z*)-2-(3-Methyl)benzo[*d*]thiazol-2(3*H*)-ylidene)-1-phenylethan-1-one (**3k**)[5] ... 35

(1*Z*)(3*E*)-2-(3-(4-Bromobenzyl)benzo[*d*]thiazol-2(3*H*)-ylidene)-1-(4-fluorophenyl)but-3-en-2-one (**3l**) . 36

(1*Z*)(3*E*)-2-(3-Benzyl)benzo[*d*]thiazol-2(3*H*)-ylidene)-1-(4-fluorophenyl)but-3-en-2-one (**3m**) ... 37

(*Z*)-2-(3-(4-Bromobenzyl)benzo[*d*]thiazol-2(3*H*)-ylidene)-1-(4-fluoro)phenylethan-1-one (**3n**) ... 38

(*Z*)-2-(3-Benzyl)benzo[*d*]thiazol-2(3*H*)-ylidene)-1-(4-fluoro)phenylethan-1-one (**3o**) ... 39

(*Z*)-2-(3-Benzyl)benzo[*d*]thiazol-2(3*H*)-ylidene)-1-(3-fluoro)phenylethan-1-one (**3p**) ... 40

(*Z*)-2-(3-Benzyl)benzo[*d*]thiazol-2(3*H*)-ylidene)-1-(2-fluoro)phenylethan-1-one (**3q**) 41
(*Z*)-2-(3-(4-Bromobenzyl)benzo[*d*]thiazol-2(3*H*)-ylidene)-1-(4-chloro)phenylethan-1-one (**3r**) 42
(*Z*)-2-(3-Benzyl)benzo[*d*]thiazol-2(3*H*)-ylidene)-1-(4-chloro)phenylethan-1-one (**3s**) 43
(*Z*)-2-(3-(4-Bromobenzyl)benzo[*d*]thiazol-2(3*H*)-yliden)-1-(6-chloropyridine-3-yl)phenylethan-1-one (**3t**) 44
(*Z*)-2-(3-Benzyl)benzo[*d*]thiazol-2(3*H*)-ylidene)-1-(6-chloropyridine-3-yl)phenylethan-1-one (**3u**) 45
(*Z*)-2-(3-(4-Bromobenzyl)benzo[*d*]thiazol-2(3*H*)-ylidene)-1-(4-bromophenyl)ethan-1-one (**3v**) 46
(*Z*)-2-(3-Benzyl)benzo[*d*]thiazol-2(3*H*)-ylidene)-1-(4-bromophenyl)ethan-1-one (**3w**) 47
(*Z*)-2-(3-(4-Bromobenzyl)benzo[*d*]thiazol-2(3*H*)-ylidene)-1-(4-iodophenyl)ethan-1-one (**3x**) 48
(*Z*)-2-(3-Benzyl)benzo[*d*]thiazol-2(3*H*)-ylidene)-1-(4-iodophenyl)ethan-1-one (**3y**) 49
(*Z*)-2-(3-(4-Bromobenzyl)benzo[*d*]thiazol-2(3*H*)-ylidene)-1-(4-(trifluoromethyl)-phenyl)ethan-1-one (**3z**)[6] 50
(*Z*)-2-(3-Benzyl)benzo[*d*]thiazol-2(3*H*)-ylidene)-1-(4-(trifluoromethyl)phenyl)ethan-1-one (**3aa**)[6] 51
(*Z*)-4-(2-(3-(4-Bromobenzyl)benzo[*d*]thiazol-2(3*H*)-ylidene)acetyl)benzonitrile (**3ab**) 52
(*Z*)-4-(2-(3-Benzylbenzo[*d*]thiazol-2(3*H*)-ylidene)acetyl)benzonitrile (**3ac**) 53
(*Z*)-2-(3-(4-Bromobenzyl)benzo[*d*]thiazol-2(3*H*)-ylidene)-1-(4-nitrophenyl)ethan-1-one (**3ad**) 54
(*Z*)-2-(3-Benzyl)benzo[*d*]thiazol-2(3*H*)-ylidene)-1-(4-nitrophenyl)ethan-1-one (**3ae**) 55
(*Z*)-2-(3-(4-Bromobenzyl)benzo[*d*]thiazol-2(3*H*)-ylidene)-1-(thiophen-2-yl)ethan-1-one (**3af**) 56
(*Z*)-2-(3-Benzyl)benzo[*d*]thiazol-2(3*H*)-ylidene)-1-(thiophen-2-yl)ethan-1-one (**3ag**) 57
(*Z*)-2-(3-(4-Bromobenzyl)benzo[*d*]thiazol-2(3*H*)-ylidene)-1-(furan-2-yl)ethan-1-one (**3ah**) 58
(*Z*)-2-(3-Benzyl)benzo[*d*]thiazol-2(3*H*)-ylidene)-1-(furan-2-yl)ethan-1-one (**3ai**) 59
5 NMR spectra 60
6 Absorption and emission spectra 96
6.1 Solvatochromism study of aroyl-*S,N*-ketene acetal 3b 124
6.2 *Hammett* correlation 125
6.3 Fluorescence excitation and emisison spectra of dye-loaded PS 127
7 DFT calculations 129
7.2 Computed xyz coordinates of selected aroyl-*S,N*-ketene acetals 3 130
8 Literature 149

1 General considerations

Reactions were carried out in dried and sintered *Schlenk* tubes or round bottom flasks under nitrogen atmosphere. Solvents were dried by a solvent purification system MB-SPS-800 of the company *MBraun Inertgas-Systeme GmbH*.

The used chemicals which have not been synthesized were purchased at *Acros Organics BVBA*, *Alfa Aeser GmbH & Co KG*, *Fluorochem Ltd.*, *J&K Scientific Ltd.*, *Merck KGaA*, *Macherey-Nagel GmbH & Co. KG*, *Sigma-Aldrich Chemie GmbH* and *VWR* and have been used without further purification. The solvents ethanol and tetrahydrofuran (THF) (spectroscopic grade) were purchased from *Merck* and *Sigma-Aldrich* (Germany), respectively. Milli-Q-water was obtained from a *Millipore water purification system*. Carboxylated 8 µm-sized polystyrene particles (PSP) were obtained from *Kisker Biotech GmbH* (Germany).

Further purification of the compounds was performed by flash column chromatography (silica gel M60 pore size 0.040-0.063 nm) of the company *Macherey-Nagel*. The crude product was adsorbed on Celite®545 of the company *Carl Roth GmbH*, placed on the suspended silica gel and purified with a positive pressure of 2 bar. Distilled solvent mixtures of *n*-hexane, acetone and methanol have been used as eluents.

The control of reaction progress was done via thin layer chromatography with silica coated aluminium plates F_{254}, of the company *Macherey-Nagel GmbH & Co. KG.*

The melting points have been measured with *Melting Point B-540* of the company *Büchi* according to the protocol of *Kofler*.[1]

^{1}H,^{13}C and DEPT 135-spectra have been measured at 298 K on an *Avance III - 300* and an *Avance III - 600* of the company *Bruker*. Chemical shifts in the ^{1}H and ^{13}C NMR are reported in ppm relative to deuterated solvents such as acetone-d_6 (δ_H 2.05, δ_C 29.84, δ_C 206.26) with CS_2 (δ_C 192.28) and dmso-d_6 (δ_H 2.50, δ_C 39.51).[2] The multiplicity is abbreviated as followed: s = singulet; d = doublet; t = triplet; td = triplet of doublet; dd = doublet of doublet; dt = doublet of triplet, dq = doublet of quartet; pd = pentet von doublet m = multiplet. The assignment of primary carbon centers (CH), secondary carbon centers (CH_2), tertiary carbon centers (CH_3) and quaternary carbon centers (C_{quat}) were made by using DEPT-135 spectra.

All mass spectrometry experiments have been performed by the department for mass spectrometry of the University of Düsseldorf (HHUCeMSA). EI mass spectra have been measured with Triple-Quadrupol-spectrometer TSQ 7000 of the company *Finnigan MAT*. MALDI spectra have been measured with a *MALDI/TOF UltrafleXtreme* of the company *Bruker Daltronik*.

IR spectra were recorded with neat compounds under attenuated total reflection (ATR) with *IRAffinity-1* of the company *Shimadzu* and the intensities were characterized as strong (s), middle (m) and weak (w).

The elementary analyses have been measured with *Perkin Elmer Series II Analyser 2400* or *Vario Micro Cube* of the company *Analysensysteme GmbH* at the microanalytical laboratory of the institute for Pharmaceutical and Medicinal Chemistry of the University Düsseldorf.

UV/Vis spectra of the dye solutions were measured with a *Lambda 19* spectrometer from *Perkin Elmer*. The emission spectra of the dye solutions and the solid compounds were recorded with a *Hitachi F-7000* spectrofluorometer using the emission correction curve provided by the instrument manufacturer. Emission spectra were not corrected for the wavelength-dependent spectral responsivity of the fluorometer. All solution spectra were recorded with dyes dissolved in spectroscopic grade solvents at 298 K using 1 cm-quartz cuvettes from Hellma GmbH. The molar extinction coefficients of dye solutions of known dye concentration were determined by five point regression line.

The fluorescence spectra of solutions and aggregates of selected dyes **3d**, **3j**, **3s**, **3ac** and **3ag** in solution and in the solid state were also measured with a calibrated spectrofluorometer from Edinburgh Instruments (FLS 920) at BAM, equipped with a xenon lamp, Czerny−Turner double monochromators, a reference channel, and Glan−Thompson polarizers. The polarizers were set to 0° and 54.7° in the excitation and emission channel, respectively (magic angle conditions). This instrument was also used for recording the fluorescence excitation and emission spectra of the dye-loaded PSP. The fluorescence excitation and emission spectra were measured with spectral bandwidths of 10 and 6 nm in excitation and emission, respectively, an integration time of 0.2 s, and a step width of 1 nm using 1cm-quartz cells from Hellma GmbH. The scan range was 400−750 nm, with three repetitive scans being performed for each sample. All spectra were subsequently corrected for the wavelength-dependent spectral responsivity of the fluorometer's detection channel determined with a calibrated spectral radiance transfer standard and a while standard.

The photoluminescence quantum yields (Φ_f) were determined absolutely with a calibrated integrating sphere setup from Hamamatsu (Quantaurus-QY C11347-11). All Φ_f measurements were performed at 25 °C using special 10 mm × 10 mm long neck quartz cuvettes from Hamamatsu. With this setup, Φ_f values ≥ 0.01 can be reliably measured.

The fluorescence decay kinetics providing the fluorescence lifetimes (τ) of the dyes, dye aggregates, and dye-loaded particles were recorded with the calibrated fluorometer Edinburgh Instruments (FLS 920) equipped with a EPLED (ex 375 ± 10 nm) and a fast multichannel plate photomultiplier (MCP-PMT) as detector. All samples were excited at the corresponding absorption maximum and the emission was always detected at the emission

maximum with a spectral bandwidth of the emission monochromator of 10 nm, a 4096-channel setting, and time ranges of 5, 20, and 50 ns. With this setup, τ values $\geq$ 0.2 ns can be reliably measured. The measured fluorescence decay kinetics were evaluated using the reconvolution procedure of the FAST program (Edinburgh Instruments). This procedure considers the measured instrument response function determined with a non-emissive scattering LUDOX solution (silica particle dispersion) which can influence the fluorescence decays. All photoluminescence decay profiles could be satisfactorily analyzed with mono-, bi- or tri-exponential fits with reduced $\chi 2$ values between 0.8 and 1.2. From the multiexponential decays, subsequently, the intensity-weighted average lifetimes were calculated and provided.

2 Overview of synthesized aroyl-*S,N*-ketene acetals

Table S1: Synthesis of aroyl-*S,N*-ketene acetals **3**.

Entry	Acid chloride **1**	Benzothiazolium salt **2**	Aroyl-*S,N*-ketene acetal **3** (yield [%])
1[(a)]	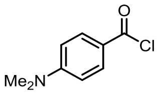 **1a**	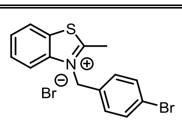 **2b**	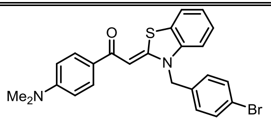 **3a** (65)
2[(a)]	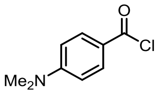 **1a**	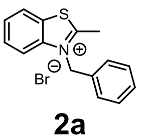 **2a**	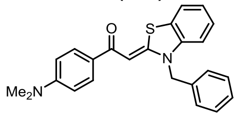 **3b** (52)
3	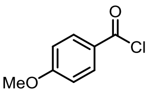 **1b**	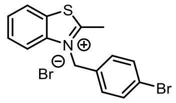 **2b**	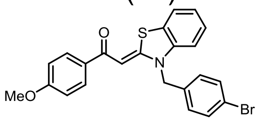 **3c** (65)
4	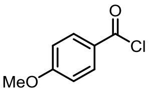 **1b**	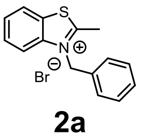 **2a**	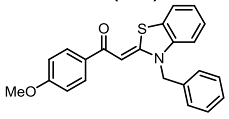 **3d** (52)
5	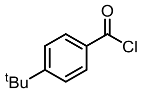 **1c**	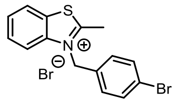 **2b**	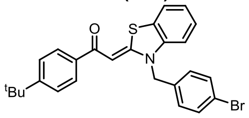 **3e** (48)
6	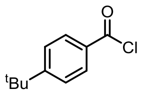 **1c**	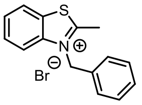 **2a**	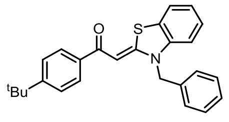 **3f** (65)
7	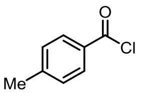 **1d**	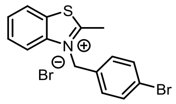 **2b**	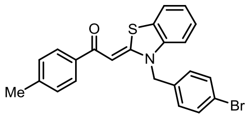 **3g** (72)
8	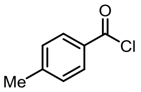 **1d**	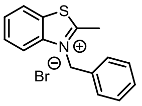 **2a**	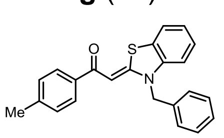 **3h** (80)
9	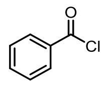 **1e**	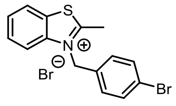 **2b**	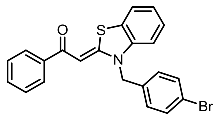 **3i** (65)

(a): Diisopropylethylamine was used as a base instead of triethylamine and only 1,4-dioxane as solvent.

Table S1: Synthesis of aroyl-*S,N*-ketene acetals **3**.

Entry	Acid chloride **1**	Benzothiazolium salt **2**	Aroyl-*S,N*-ketene acetal **3** (yield [%])
10	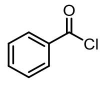 **1e**	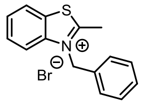 **2a**	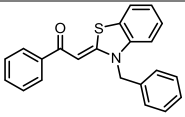 **3j** (48)
11	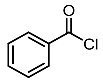 **1e**	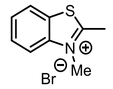 **2c**	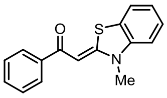 **3k** (94)
12	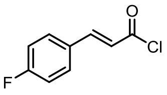 **1f**	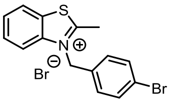 **2b**	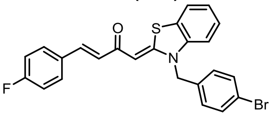 **3l** (22)
13	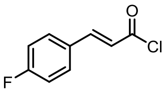 **1f**	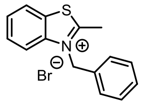 **2a**	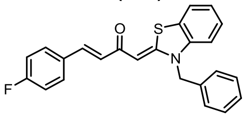 **3m** (31)
14	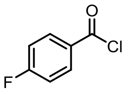 **1g**	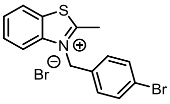 **2b**	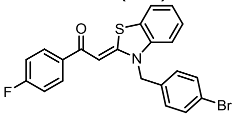 **3n** (69)
15	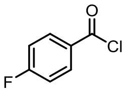 **1g**	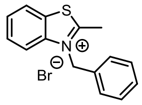 **2a**	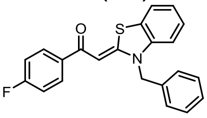 **3o** (85)
16	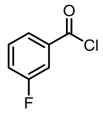 **1h**	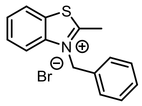 **2a**	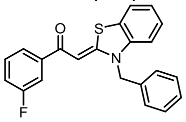 **3p** (65)
17	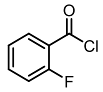 **1i**	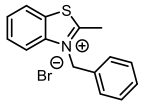 **2a**	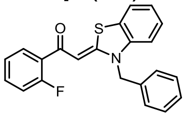 **3q** (49)
18	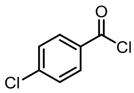 **1j**	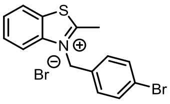 **2b**	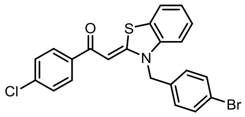 **3r** (83)

(a): Diisopropylethylamine was used as a base instead of triethylamine and only 1,4-dioxane as solvent.

Table S1: Synthesis of aroyl-*S,N*-ketene acetals **3**.

Entry	Acid chloride **1**	Benzothiazolium salt **2**	Aroyl-*S,N*-ketene acetal **3** (yield [%])
19	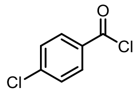 **1j**	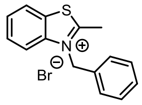 **2a**	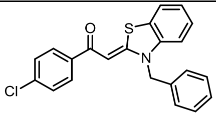 **3s** (88)
20	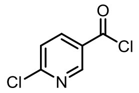 **1k**	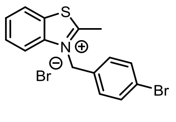 **2b**	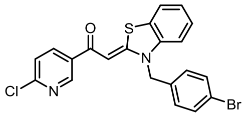 **3t** (27)
21	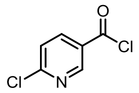 **1k**	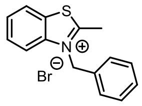 **2a**	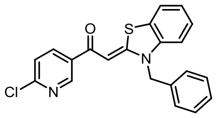 **3u** (27)
22	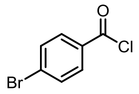 **1l**	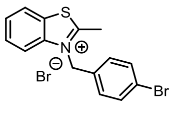 **2b**	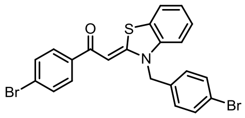 **3v** (55)
23	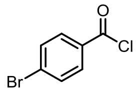 **1l**	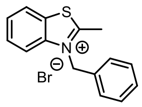 **2a**	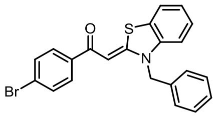 **3w** (88)
24	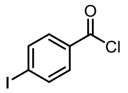 **1m**	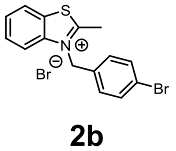 **2b**	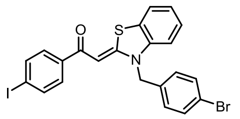 **3x** (59)
25	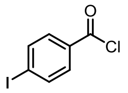 **1m**	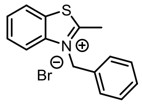 **2a**	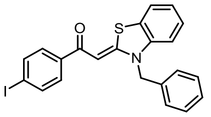 **3y** (65
26	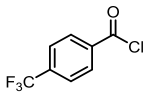 **1n**	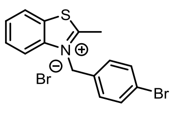 **2b**	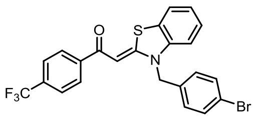 **3z** (72)

(a): Diisopropylethylamine was used as a base instead of triethylamine and only 1,4-dioxane as solvent.

Table S1: Synthesis of aroyl-*S,N*-ketene acetals **3**.

Entry	Acid chloride<br>**1**	Benzothiazolium salt<br>**2**	Aroyl-*S,N*-ketene acetal<br>**3** (yield [%])
27	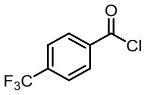<br>**1n**	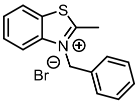<br>**2a**	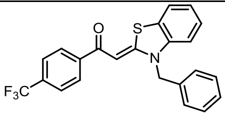<br>**3aa** (50)
28	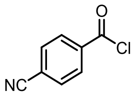<br>**1o**	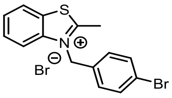<br>**2b**	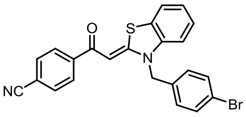<br>**3ab** (51)
29	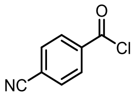<br>**1o**	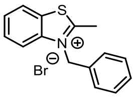<br>**2a**	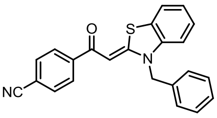<br>**3ac** (66)
30	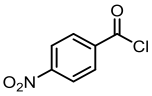<br>**1p**	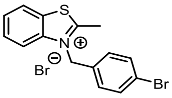<br>**2b**	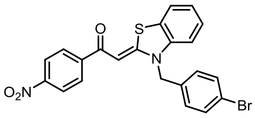<br>**3ad** (44)
31	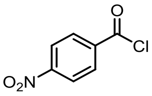<br>**1p**	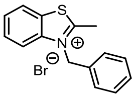<br>**2a**	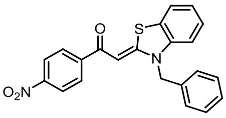<br>**3ae** (73)
32	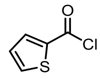<br>**1q**	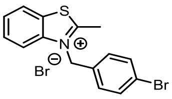<br>**2b**	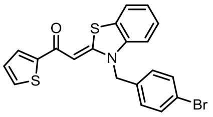<br>**3af** (72)
33	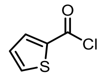<br>**1q**	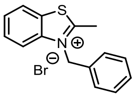<br>**2a**	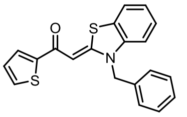<br>**3ag** (87)
34	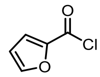<br>**1r**	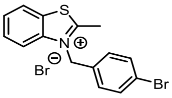<br>**2b**	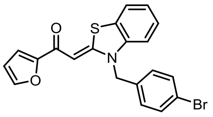<br>**3ah** (77)
35	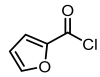<br>**1r**	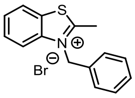<br>**2a**	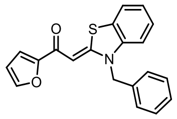<br>**3ai** (95)

(a): Diisopropylethylamine was used as a base instead of triethylamine and only 1,4-dioxane as solvent.

3 Overview of photophysical properties of aroyl-*S,N*-ketene acetals 3

Table S2: Photophysical properties of aroyl-*S,N*-ketene acetal **3**.

Entry	Aroyl-*S,N*-ketene acetal	$\lambda_{max(abs.)}$ solution [nm] (ε [L·mol^{-1}·cm^{-1}])[a]	$\lambda_{max(em.)}$ solid state [nm][b]	solid state[c]	aggregate[d]
1	**3a**	404 (57700)	604	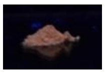	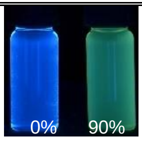
2	**3b**	404 (60300)	534	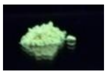	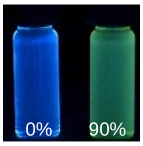
3	**3c**	383 (42800)	447	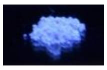	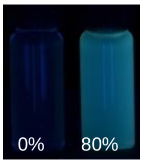
4	**3d**	384 (43500)	439<br>465<br>497	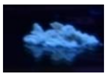	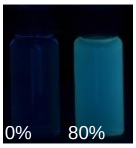
5	**3e**	383 (44700)	500	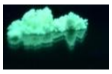	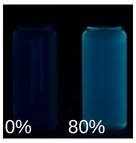
6	**3f**	383 (42700)	489	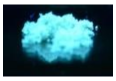	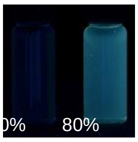

[a]: measured in ethanol, T = 298K, c = 10^{-5} M, [b]: T = 298 K, λ_{exc} = $\lambda_{abs,max}$, [c]: pictures taken under UV-light (λ_{exc} = 365 nm), [d]: photograph of aggregates taken in ethanol/water-mixtures.

Table S2: Photophysical properties of aroyl-*S,N*-ketene acetal **3**.

Entry	Aroyl-*S,N*-ketene acetal	$\lambda_{max(abs.)}$ solution [nm] (ε [L·mol^{-1}·cm^{-1}])[a]	$\lambda_{max(em.)}$ solid state [nm][b]	solid state[c]	aggregate[d]
7	**3g**	382 (40300)	536	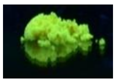	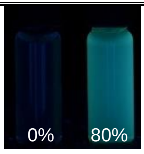
8	**3h**	382 (40000)	500	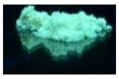	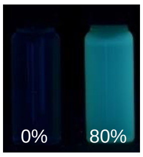
9	**3i**	376 (58400)	500<br>520 (sh)	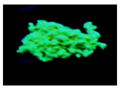	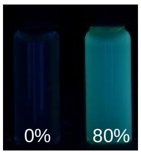
10	**3j**	376 (39100)	477<br>500 (sh)	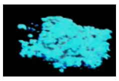	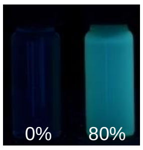
11	**3k**	382 (41400)	459<br>528	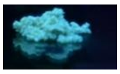	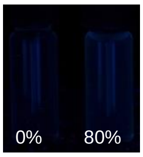
12	**3l**	410 (41400)	-	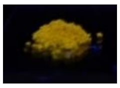	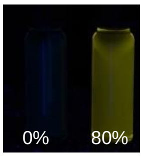
13	**3m**	411 (11000)	-	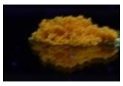	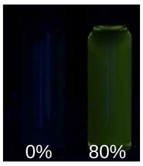
14	**3n**	381 (41100)	500<br>552	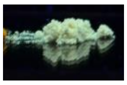	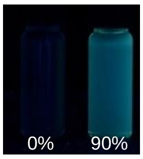
15	**3o**	381 (47200)	441<br>496	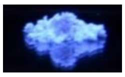	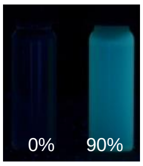

[a]: measured in ethanol, T = 298K, $c = 10^{-5}$ M; [b]: T = 298 K, $\lambda_{exc} = \lambda_{abs,max}$; [c]: pictures taken under UV-light (λ_{exc} = 365 nm); [d]: photographs of aggregates taken in ethanol/water-mixtures.

Table S2: Photophysical properties of aroyl-*S,N*-ketene acetal **3**.

Entry	Aroyl-*S,N*-ketene acetal	$\lambda_{max(abs.)}$ solution [nm] (ε [L·mol^{-1}·cm^{-1}])[a]	$\lambda_{max(em.)}$ solid state [nm][b]	solid state[c]	aggregate[d]
16	**3p**	387 (74700)	487	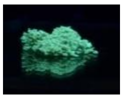	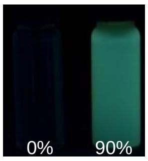
17	**3q**	383 (85400)	467	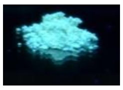	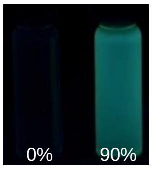
18	**3r**	386 (39000)	545	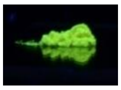	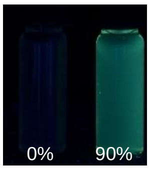
19	**3s**	387 (40000)	471<br>499	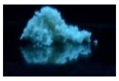	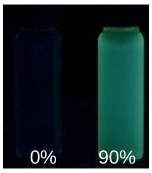
20	**3t**	391 (38400)	-	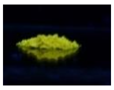	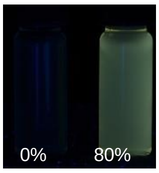
21	**3u**	392 (34800)	-	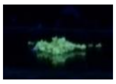	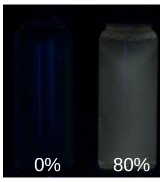
22	**3v**	387 (36800)	550	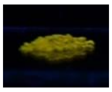	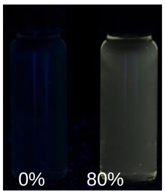
23	**3w**	388 (38400)	500	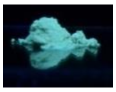	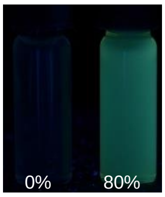

[a]: measured in ethanol, T = 298K, c = 10^{-5} M; [b]: T = 298 K, λ_{exc} = $\lambda_{abs,max}$; [c]: pictures taken under UV-light (λ_{exc} = 365 nm); [d]: photographs of aggregates taken in ethanol/water-mixtures.

Table S2: Photophysical properties of aroyl-*S,N*-ketene acetal **3**.

Entry	Aroyl-*S,N*-ketene acetal	$\lambda_{max(abs.)}$ solution [nm] (ε [L·mol^{-1}·cm^{-1}])[a]	$\lambda_{max(em.)}$ solid state [nm][b]	solid state[c]	aggregate[d]
24	**3x**	389 (70100)	491	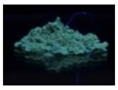	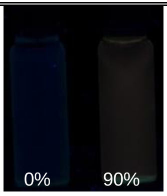
25	**3y**	389 (88600)	472<br>546<br>589<br>589	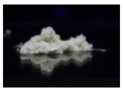	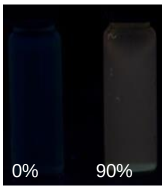
26	**3z**	390 (36600)	515	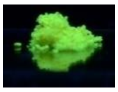	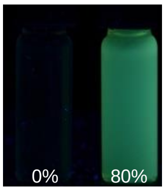
27	**3aa**	391 (31800)	489	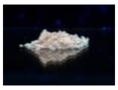	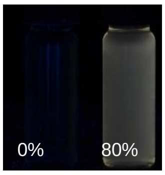
28	**3ab**	397 (31500)	495 (sh)<br>552<br>597	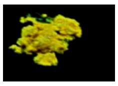	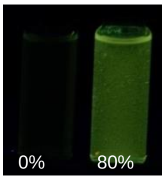
29	**3ac**	397 (43600)	516<br>593	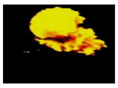	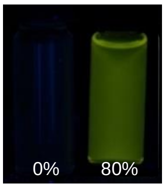
30	**3ad**	413 (12000)	627	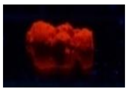	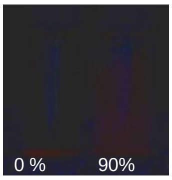
31	**3ae**	413 (22200)	646	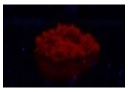	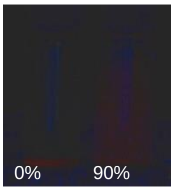

[a]: measured in ethanol, T = 298K, c = 10^{-5} M; [b]: T = 298 K, λ_{exc} = $\lambda_{abs,max}$; [c]: pictures taken under UV-light (λ_{exc} = 365 nm); [d]: photographs of aggregates taken in ethanol/water-mixtures.

Table S2: Photophysical properties of aroyl-*S,N*-ketene acetal **3**.

Entry	Aroyl-*S,N*-ketene acetal	$\lambda_{max(abs.)}$ solution [nm] (ε [L·mol^{-1}·cm^{-1}])[a]	$\lambda_{max(em.)}$ solid state [nm][b]	solid state[c]	aggregate[d]
32	**3af**	391 (38800)	522	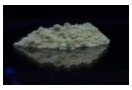	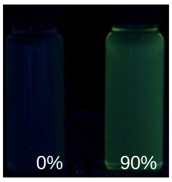
33	**3ag**	392 (39200)	499	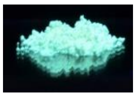	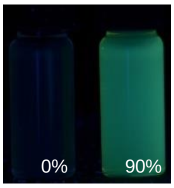
34	**3ah**	398 (58800)	478	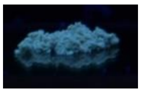	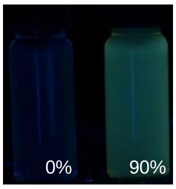
35	**3ai**	399 (43900)	447<br>507	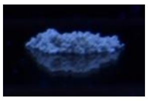	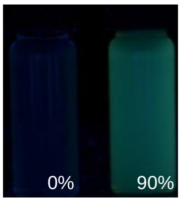

[a]: measured in ethanol, T = 298K, c = 10^{-5} M; [b]: T = 298 K, λ_{exc} = $\lambda_{abs,max}$; [c]: pictures taken under UV-light (λ_{exc} = 365 nm); [d]: photographs of aggregates taken in ethanol/water-mixtures.

Relative quantum yield of aroyl-*S,N*-ketene acetal **3a** and **3b**, determined with coumarin 343 as a standard in ethanol (Φ_F = 0.63): Φ_F = 0.07.

4 Syntheses

4.1 Synthesis of starting materials

4.1.1 General procedure (GPI) Synthesis of benzothiazoliumbromides 2[3]

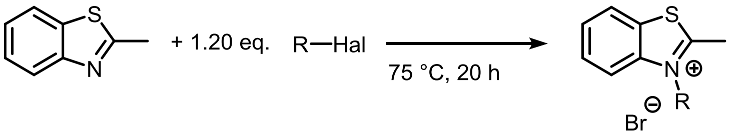

2

2-Methylbenzothiazole (1.00 equiv) and the corresponding halide were placed in a round-bottom flask. The reaction mixture was stirred at 75 °C for 20 h, until the solution was completely hardened. The formed solid was filtrated via a Buechner funnel, washed with diethyl ether and dried under vacuo.

Table S3: Experimental details for the synthesis of benzothiazoliumbromides **2**.[3]

Entry	2-Methylbenzothiazole	Halide	Yield of product **2**
**1**	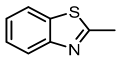 7.45 g (50.0 mmol)	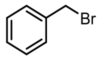 10.2 g (60.0 mmol)	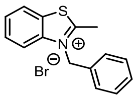 12.4 g (78%) of **2a**
**2**	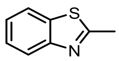 3.73 g (25.0 mmol)	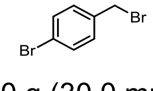 7.50 g (30.0 mmol)	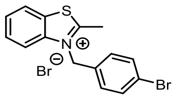 9.85 g (99%) of **2b**
**3**	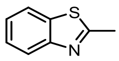 1.49 g (10.0 mmol)	MeI 4.24 g (30.0 mmol)	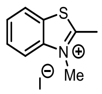 2.52 g (87%) of **2c**

4.1.1.1 Spectroscopic data

3-Benzyl-2-methylbenzo[*d*]thiazol-3-iumbromide (2a)[3]

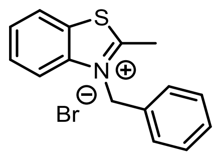

$C_{15}H_{14}BrNS$

[319.00]

The synthesis was performed according to **GPI** to give 12.4 g (39.0 mmol, 78%) of the desired product **2a** as a pink solid.

Mp: 200 °C.

R_f (*n*-hexane/acetone 4:1): 0.10.

^{1}H NMR (300 MHz, dmso-d$_6$): δ 3.30 (s, 3 H), 6.14 (s, 2 H), 7.33-7.40 (m, 5 H), 7.80 (pd, ^3J = 7.5 Hz, ^4J = 1.9 Hz, 2 H), 8.23 (dd, ^3J = 7.8 Hz, ^4J = 1.4 Hz, 1 H), 8.56 (dd, ^3J = 7.4 Hz, ^4J = 1.8 Hz, 1 H).

^{13}C NMR (75 MHz, dmso-d$_6$): δ 17.5 (CH_3), 51.9 (CH_2), 117.1 (CH), 127.0 (CH), 128.3 (CH), 128.5 (CH), 129.1 (CH), 129.2 (C_{quat}), 129.5 (CH), 132.8 (C_{quat}), 140.8 (C_{quat}), 178.3 (C_{quat}).

EI + MS (70 eV, *m/z* (%)): 240 (15), 239 ($[C_{15}H_{14}NS]^+$, 67), 238 (65), 224 ($[C_{14}H_{10}NS]^+$, 14), 162 ($[C_9H_8NS]^+$, 13), 148 ($[C_8H_6NS]^+$, 32), 104 ($[C_7H_6N]^+$, 12), 91 ($[C_7H_7]^+$, 100), 65 ($[C_5H_5]^+$, 23).

IR $\tilde{\nu}$ [cm^{-1}]: 648 (m), 689 (w), 706 (m), 729 (m), 748 (s), 764 (s), 808 (w), 860 (w), 943 (w), 1001 (w), 1024 (w), 1061 (w), 1082 (w), 1126 (w), 1148 (w), 1157 (w), 1180 (w) 1200 (w), 1242 (w), 1285 (w), 1314 (w), 1331 (w), 1339 (w), 1368 (w), 1418 (w), 1435 (w), 1441 (m), 1456 (m), 1495 (w), 1514 (w), 2251 (w), 2675 (w), 2725 (w), 2820 (w), 2874 (w), 2901 (w), 2938 (w), 3001 (w), 3055 (w).

Anal calcd for $C_{15}H_{14}BrNS$ [319.0]: C 56.26, H 4.41, N 4.37, S 10.01; Found: C 56.53, H 4.29, N 4.37, S 10.10.

3-(4-Bromobenzyl)-2-methylbenzo[*d*]thiazol-3-iumbromide (2b)

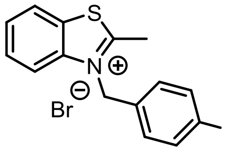

$C_{15}H_{13}Br_2NS$

[396.91]

The synthesis was performed according to **GPI** to give 9.85 g (24.8 mmol, 99%) of the desired product **2b** as a pink solid.

Mp: 237 °C.

R_f (*n*-hexane/acetone 4:1): 0.10.

^{1}H NMR (300 MHz, dmso-d$_6$): δ 3.25 (s, 3 H), 6.07 (s, 2 H), 7.29-7.32 (m, 2 H), 7.58-7.61 (m, 2 H), 7.82 (pd, ^3J = 7.5 Hz, ^4J = 1.9 Hz, 2 H), 8.18 (dd, ^3J = 7.5 Hz, ^4J = 1.5 Hz, 1 H), 8.50 (dd, ^1J = 7.4 Hz, ^2J = 1.8 Hz, 1 H).

^{13}C NMR (75 MHz, dmso-d$_6$): δ 17.4 (CH_3), 51.3 (CH_2), 117.0 (CH), 121.8 (C_{quat}), 124.9 (CH), 128.2 (CH), 129.3 (CH), 129.4 (CH), 129.5 (C_{quat}), 131.9 (CH), 132.2 (C_{quat}), 140.9 (C_{quat}), 178.7 (C_{quat}).

EI + MS (70 eV, *m/z* (%)): 320 ([$C_{15}H_{13}{}^{81}BrNS^+$, 13), 319 ([$C_{15}H_{12}{}^{81}BrNS]^+$, 69), 318 ([$C_{15}H_{13}{}^{79}BrNS^+$, 57), 317 ([$C_{15}H_{12}{}^{79}BrNS]^+$, 68), 316 (51), 238 ([$C_{15}H_{13}NS]^+$, 13), 236 (11), 223 ([$C_{14}H_9NS]^+$, 12), 171 ([$C_7H_6{}^{81}Br]^+$, 95), 169 ([$C_7H_6{}^{79}Br]^+$, 100), 162 ([$C_9H_8NS]^+$, 23), 149 ([$C_8H_6NS]^+$, 15), 148 ([$C_8H_6NS]^+$, 34), 124 (13), 119 (18), 118 (21), 108 (12), 104 ([$C_7H_6N]^+$, 12), 90 ([$C_7H_6]^+$, 43), 89 (36), 82 (14), 63 (12).

IR $\tilde{\nu}$ [cm^{-1}]: 702 (m), 718 (m), 733 (m), 762 (m), 775 (s), 785 (m), 820 (m), 870 (w), 924 (w), 1001 (m), 1011 (m), 1070 (m), 1090 (w), 1117 (w), 1157 (w), 1198 (m) 1231 (w), 1244 (w), 1285 (w), 1337 (m), 1354 (w), 1375 (m), 1406 (m), 1418 (m), 1431 (m), 1460 (m), 1489 (m), 1504 (m), 1574 (w), 1593 (w), 2388 (w), 2727 (w), 2795 (w), 2822 (w), 2974 (w), 2999 (w), 3042 (w), 3069 (w), 3118 (w).

Anal calcd for $C_{15}H_{13}Br_2NS$ [397.0]: C 45.14, H 3.28, N 3.51, S 8.03; Found: C 45.20, H 3.31, N 3.37, S 8.32.

2,3-Dimethylbenzo[*d*]thiazol-3-iumiodide (2c)[4]

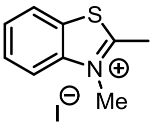

$C_9H_{10}INS$

[290.31]

The synthesis was performed according to **GPI** to give 2.52 g (8.67 mmol, 87%) of the desired product **3c** as a colourless solid.

Mp: 230 °C.

^{1}H NMR (500 MHz, dmso-d$_6$): δ 3.20 (s, 3 H), 4.22 (s, 3 H), 6.14 (s, 2 H), 7.80 (dt, ^3J = 7.8 Hz, ^4J = 0.9 Hz, 1 H), 7.88 (dt ^3J = 7.8 Hz, ^4J = 1.2 Hz, 2 H), 8.29 (d, ^3J = 8.3 Hz, 1 H), 8.47 (d, ^3J = 8.2 Hz, 1 H).

^{13}C NMR (125 MHz, dmso-d$_6$): δ 17.3 (CH_3), 36.1 (CH_3), 116.6 (CH), 124.3 (CH), 127.9 (CH), 128.5 (C_{quat}), 129.1 (CH), 141.4 (C_{quat}), 177.0 (C_{quat}).

ESI-MS (*m/z*): 164.2 ($C_9H_{10}NS^+$).

4.2 Synthesis and analytical data of aroyl-*S,N*-ketene acetals 3

4.3 General procedure (GPII) for the synthesis of aroyl-*S,N*-ketene acetals 3

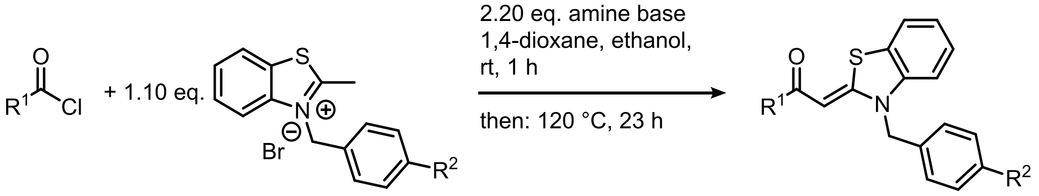

1 **2** **3**

Acid chloride **4** (1.00 equiv, 1.00 mmol) and 1.10 equiv (1.10 mmol) of 4 benzylbenzothiazoliumbromide **3** were placed in a sintered, dry screw-cap *Schlenk*-tube under nitrogen atmosphere and dissolved in 5 mL dry 1,4-dioxane and 2 mL ethanol. 2.20 equiv (2.20 mmol) amine base was added to the reaction mixture and the solution was stirred for 1 h at room temperature. Thereafter, the reaction mixture was stirred at 120 °C (oil bath) for 23 h. The crude product was absorbed onto Celite® and purified by flash chromatography on silica gel (*n*-hexane/acetone). The product was suspended in *n*-hexane, the sediment was filtrated and dried under vacuo.

Table S4: Experimental details for the synthesis of aroyl-*S,N*-ketene acetals **3**.

Entry	Acid chloride **1** [mg] ([mmol])	Benzylbenzo-thiazoliumbromide **2** [mg] ([mmol])	Yield of product **3** [mg] (%)
1[(a)]	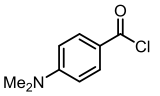 183 (1.00) of **1a**	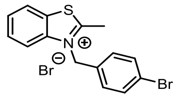 431 (1.10) of **2b**	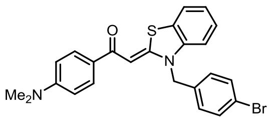 317 (68) of **3a**
2[(a)]	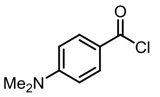 183 (1.00) of **1a**	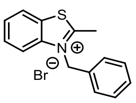 351 (1.10 mmol) of **2a**	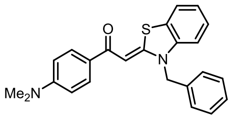 278 (72) of **3b**
3	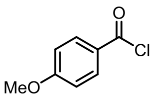 170 (1.00) of **1b**	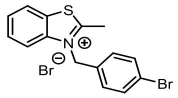 431 (1.10) of **2b**	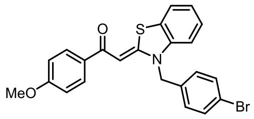 309 (69) of **3c**
4	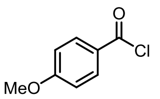 170 (1.00) of **1b**	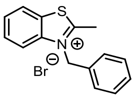 351 (1.10) of **2a**	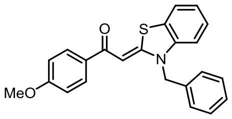 192 (52) of **3d**
5	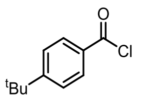 197 (1.00) of **1c**	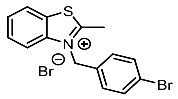 431 (1.10) of **2b**	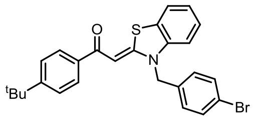 228 (48) of **3e**
6	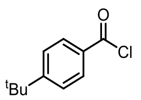 197 (1.00) of **1c**	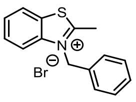 351 (1.10) of **2a**	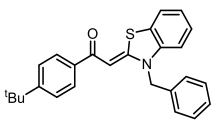 258 (65) of **3f**
7	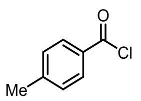 155 (1.00) of **1d**	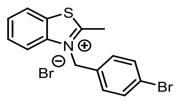 431 (1.10) of **2b**	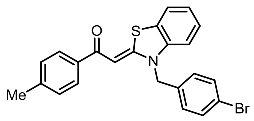 315 (72) of **3g**
8	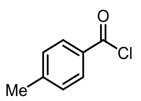 155 (1.00) of **1d**	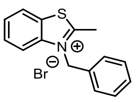 351 (1.10) of **2a**	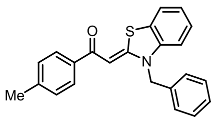 287 (80) of **3h**

(a): Diisopropylethylamine was used as a base instead of triethylamine and solely 1,4-dioxane as a solvent.

Table S4: Experimental details for the synthesis of aroyl-*S,N*-ketene acetals **3**.

Entry	Acid chloride **1** [mg] ([mmol])	Benzylbenzo-thiazoliumbromide **2** [mg] ([mmol])	Yield of product **3** [mg] ([%])
9	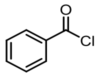 140 (1.00) of **1e**	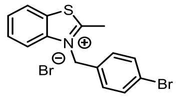 431 (1.10) of **2b**	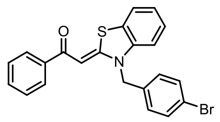 273 (65) of **3i**
10	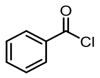 1400 (10.0) of **1e**	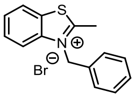 3510 (11.0) of **2a**	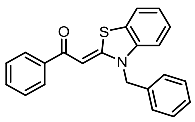 1980 (58) of **3j**
11	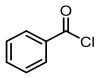 140 (1.00) of **1e**	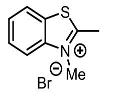 315 (1.10) of **2c**	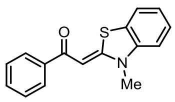 250 (94) of **3k**
12	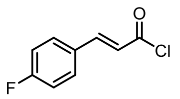 184 (1.00) of **1f**	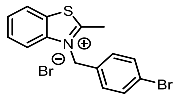 431 (1.10) of **2b**	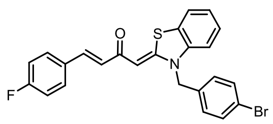 100 (22) of **3l**
13	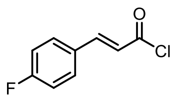 184 (1.00) of **1f**	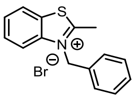 351 (1.10) of **2a**	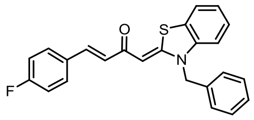 121(31) of **3m**
14	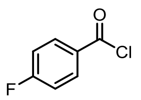 158 (1.00) of **1g**	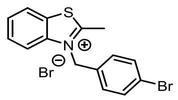 431 (1.10) of **2b**	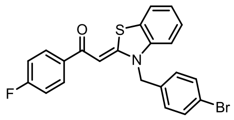 304 (69) of **3n**
15	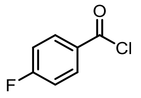 158 (1.00) of **1g**	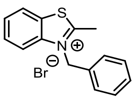 351 (1.10) of **2a**	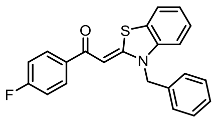 306 (85) of **3o**
16	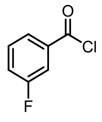 158 (1.00) of **1h**	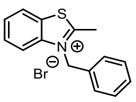 351 (1.10) of **2a**	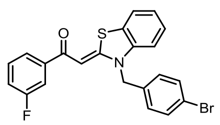 234 (65) of **3p**

(a): Diisopropylethylamine was used as a base instead of triethylamine and solely 1,4-dioxane as a solvent.

Table S4: Experimental details for the synthesis of aroyl-*S,N*-ketene acetals **3**.

Entry	Acid chloride **1** [mg] ([mmol])	Benzylbenzo-thiazoliumbromide **2** [mg] ([mmol])	Yield of product **3** [mg] ([%])
17	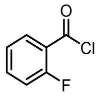 158 (1.00) of **1i**	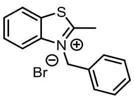 351 (1.10) of **2a**	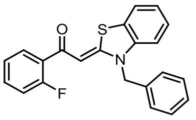 175 (49) of **3q**
18	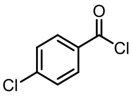 175 (1.00) of **1j**	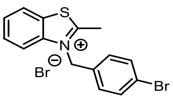 431 (1.10) of **2b**	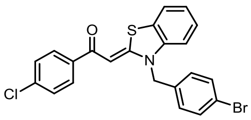 376 (83) of **3r**
19	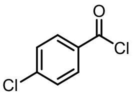 175 (1.00) of **1j**	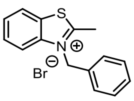 351 (1.10) of **2a**	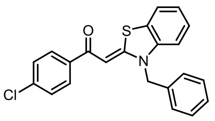 332 (88) of **3s**
20	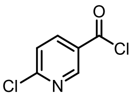 176 (1.00) of **1k**	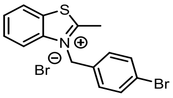 431 (1.10) of **2b**	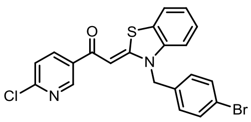 124 (27) of **3t**
21	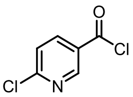 176 (1.00) of **1k**	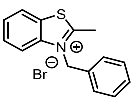 351 (1.10) of **2a**	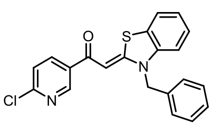 234 (27) of **3u**
22	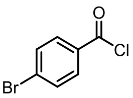 218 (1.00) of **1l**	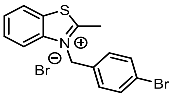 431 (1.10) of **2b**	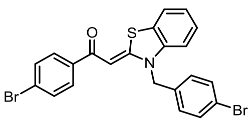 273 (55) of **3v**
23	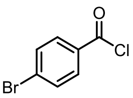 218 (1.00) of **1l**	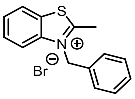 351 (1.10) of **2a**	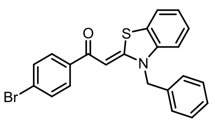 370 (88) of **3w**
24	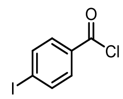 266 (1.00) of **1m**	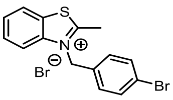 431 (1.10) of **2b**	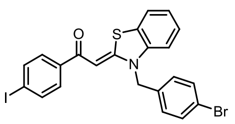 324 (59) of **3x**

(a): Diisopropylethylamine was used as a base instead of triethylamine and solely 1,4-dioxane as a solvent.

Table S4: Experimental details for the synthesis of aroyl-*S,N*-ketene acetals **3**.

Entry	Acid chloride **1** [mg] ([mmol])	Benzylbenzo-thiazoliumbromide **2** [mg] ([mmol])	Yield of product **3** [mg] ([%])
25	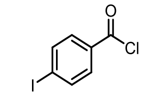 266 (1.00) of **1m**	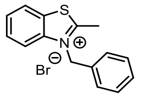 351 (1.10) of **2a**	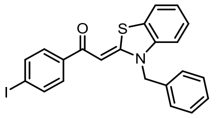 648 (65) of **3y**
26	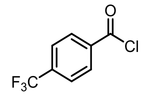 208 (1.00) of **1n**	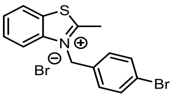 431 (1.10) of **2b**	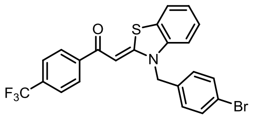 352 (72) of **3z**
27	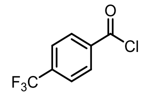 208 (1.00) of **1n**	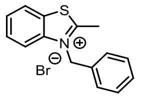 351 (1.10) of **2a**	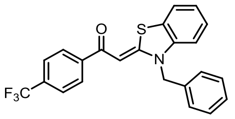 207 (50) of **3aa**
28	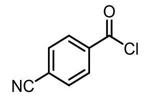 165 (1.00) of **1o**	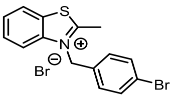 431 (1.10) of **2b**	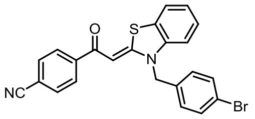 226 (51) of **3ab**
29	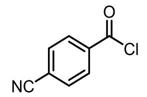 165 (1.00) of **1o**	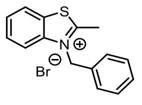 351 (1.10) of **2a**	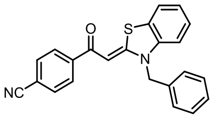 241 (66) of **3ac**
30	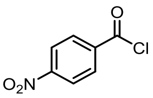 185 (1.00) of **1p**	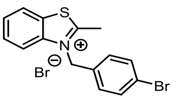 431 (1.10) of **2b**	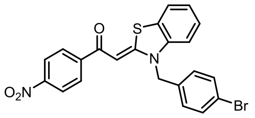 207 (44) of **3ad**
31	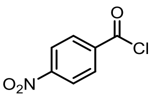 185 (1.00) of **4p**	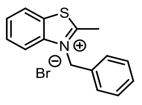 351 (1.10) of **3a**	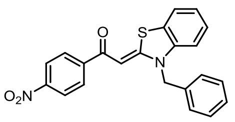 285 (73) of **3ae**
32	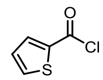 147 (1.00) of **4q**	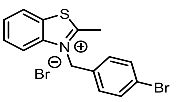 431 (1.10) of **3b**	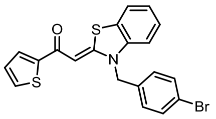 306 (72) of **3af**

(a): Diisopropylethylamine was used as a base instead of triethylamine and solely 1,4-dioxane as a solvent.

Table S4: Experimental details for the synthesis of aroyl-*S,N*-ketene acetals **3**.

Entry	Acid chloride **1** [mg] ([mmol])	Benzylbenzo-thiazoliumbromide **2** [mg] ([mmol])	Yield of product **3** [mg] ([%])
33	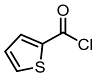 147 (1.00) of **1q**	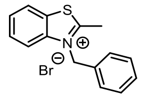 351 (1.10) of **2a**	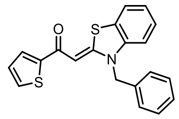 305 (87) of **3ag**
34	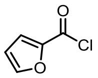 140 (1.00) of **1r**	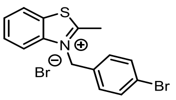 431 (1.10) of **2b**	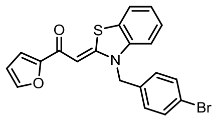 315 (77) of **3ah**
35	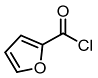 140 (1.00) of **1r**	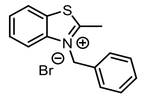 351 (1.10) of **2b**	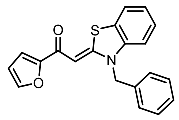 317 (95) of **3ai**

(a): Diisopropylethylamine was used as a base instead of triethylamine and solely 1,4-dioxane as a solvent.

4.3.1.1 Spectroscopic data

(*Z*)-2-(3-(4-Bromobenzyl)benzo[*d*]thiazol-2(3*H*)-ylidene)-1-(4-((dimethylamino)phenyl) ethan-1-one (3a)

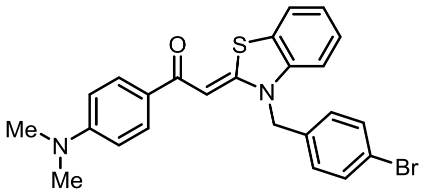

$C_{24}H_{21}BrN_2OS$

[464.06]

The synthesis was performed according to **GPII** to give 317 mg (0.683 mmol, 68%) of the desired product **3a** as a yellow solid.

Mp: 178 °C.

R_f (*n*-hexane/acetone 4:1): 0.11.

^{1}H NMR (300 MHz, acetone-d_6/CS_2 5:1): δ 3.03 (s, 6 H), 5.45 (s, 2 H), 6.62-6.75 (m, 4 H), 7.13-7.32 (m, 4 H), 7.51 (d, ^3J = 8.5 Hz, 1 H), 7.64 (d, ^3J = 7.6 Hz, 1 H), 7.20-7.22 (m, 3 H).

^{13}C NMR (75 MHz, acetone-d_6/CS_2 5:1): δ 40.2 (CH_3), 40.4 (CH_3), 49.1 (CH_2), 88.0 (CH), 110.6 (CH), 111.8 (CH), 122.2 (CH), 123.0 (CH), 123.4 (C_{quat}), 127.1 (CH), 128.5 (C_{quat}), 129.5 (C_{quat}), 129.6 (CH), 132.1 (CH), 132.8 (CH), 132.9 (CH), 135.6 (C_{quat}), 140.9 (C_{quat}), 153.0 (C_{quat}), 160.6 (C_{quat}), 183.7 (C_{quat}).

MALDI-TOF (*m/z*): 467.1 ($C_{24}H_{21}{}^{81}BrN_2OS+H^+$), 465.1 ($C_{24}H_{21}{}^{79}BrN_2OS+H^+$).

IR $\tilde{\nu}$ [cm^{-1}]: 658 (w), 689 (w), 698 (w), 712 (m), 745 (s), 768 (s), 799 (m), 822 (m), 881 (m), 945 (m), 970 (m), 1009 (m), 1047 (m), 1063 (m), 1088 (w), 1125 (m), 1163 (s), 1184 (s), 1242 (m), 1294 (m), 1319 (m), 1341 (m), 1371 (m), 1406 (m), 1443 (m), 1477 (s), 1533 (m), 1545 (s), 1578 (s), 1686 (w), 1734 (w), 2533 (w), 2806 (w), 2889 (w), 2913 (w), 3049 (w), 3906 (w).

UV/Vis (C_3H_6O): λ_{max} (ε) = 404 (57700).

Anal calcd for $C_{24}H_{21}BrN_2OS$ [464.1]: C 61.94, H 4.55, N 6.02, S 6.89; Found: C 62.29, H 4.82, N 6.23, S 6.64.

(*Z*)-2-(3-Benzyl)benzo[*d*]thiazol-2(3*H*)-ylidene)-1-(4-((dimethylamino)phenyl)ethan-1-one (3b)

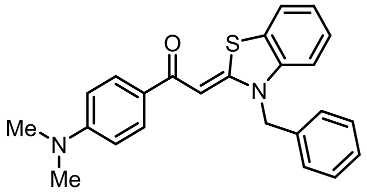

$C_{24}H_{22}N_2OS$

[386.15]

The synthesis was performed according to **GPII** to give 278 mg (0.720 mmol, 72%) of the desired product **3b** as a yellow solid.

Mp: 212 °C.

R_f (*n*-hexane/acetone 4:1): 0.26.

^{1}H NMR (300 MHz, acetone-d$_6$/CS$_2$ 5:1): δ 3.02 (s, 6 H), 5.47 (s, 2 H), 6.62-6.70 (m, 4 H), 7.14-7.38 (m, 7 H), 7.63-7.85 (m, 3 H).

^{13}C NMR (75 MHz, acetone-d$_6$/CS$_2$ 5:1): δ 40.2 (CH_3), 40.3 (CH_3), 49.7 (CH_2), 88.0 (CH), 110.8 (CH), 111.8 (CH), 122.9 (CH), 123.3 (CH), 127.1 (CH), 127.4 (CH), 128.5 (CH), 129.6 (C_{quat}), 129.7 (C_{quat}), 132.2 (CH), 136.3 (C_{quat}), 141.2 (C_{quat}), 153.1 (C_{quat}), 160.7 (C_{quat}), 183.7 (C_{quat}).

MALDI-TOF (*m/z*): 387.2 ($C_{24}H_{22}N_2OS+H^+$).

IR $\tilde{\nu}$ [cm^{-1}]: 656 (m), 692 (m), 712 (s), 733 (m), 745 (s), 766 (s), 795 (m), 814 (m), 826 (m), 874 (m), 903 (m), 930 (m), 945 (m), 968 (w), 999 (m), 1024 (m), 1047 (m), 1063 (m), 1090 (m), 1125 (m), 1169 (s), 1180 (s), 1234 (m), 1269 (m), 1294 (m), 1341 (m), 1366 (m), 1400 (m), 1425 (m), 1441 (s), 1477 (s), 1530 (m), 1545 (m), 1578 (m), 1612 (m), 1665 (w), 2552 (w), 2637 (w), 2671 (w), 2778 (w), 2812 (w), 2859 (w), 2884 (w), 3030 (w), 3059 (w).

UV/Vis (C_3H_6O): λ_{max} (ε) = 404 (60300).

Anal calcd for $C_{24}H_{22}N_2OS$ [386.2]: C 74.58, H 5.74, N 7.25, S 8.29; Found: C 74.43, H 5.89, N 7.25, S 8.42.

(*Z*)-2-(3-(4-Bromobenzyl)benzo[*d*]thiazol-2(3*H*)-ylidene)-1-(4-(methoxyphenyl)ethan-1-one (3c)

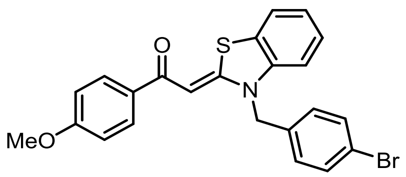

$C_{23}H_{18}BrNO_2S$

[451.02]

The synthesis was performed according to **GPII** to give 309 mg (0.685 mmol, 69%) of the desired product **3c** as a yellow solid.

Mp: 169 °C.

R_f (*n*-hexane/acetone 4:1): 0.18.

^{1}H NMR (600 MHz, acetone-d$_6$/CS$_2$ 5:1): δ 3.83 (s, 3 H), 5.56 (s, 2 H), 6.82 (s, 1 H), 6.92 (d, ^3J = 8.8 Hz, 2 H), 7.20-7.22 (m, 1 H), 7.27 (d, ^3J = 8.1 Hz, 2 H), 7.31-7.36 (m, 2 H), 7.49-7.54 (m, 2 H), 7.72 (d, ^3J = 8.0 Hz, 1 H), 7.93 (d, ^3J = 8.1 Hz, 2 H).

^{13}C NMR (150 MHz, acetone-d$_6$/CS$_2$ 5:1): δ 48.2 (CH_2), 54.9 (CH_3), 87.1 (CH), 110.4 (CH), 113.4 (CH), 121.1 (CH), 122.3 (CH), 122.9 (CH), 126.6 (CH), 127.1 (C_{quat}), 128.8 (CH), 129.0 (CH), 131.9 (CH), 132.3 (C_{quat}), 135.0 (C_{quat}), 140.0 (C_{quat}), 161.0 (C_{quat}), 162.0 (C_{quat}), 183.0 (C_{quat}).

MALDI-TOF (*m/z*): 454.1 ($C_{23}H_{18}{}^{81}BrNO_2S+H^+$), 452.1 ($C_{23}H_{18}{}^{79}BrNO_2S^+ + H^+$).

IR $\tilde{\nu}$ [cm^{-1}]: 617 (m), 708 (m), 735 (s), 764 (s), 797 (m), 843 (m), 880 (s), 1007 (m), 1024 (m), 1036 (m), 1065 (m), 1088 (m), 1113 (m), 1138 (w), 1165 (s), 1184 (m), 1227 (s), 1254 (m), 1294 (m), 1308 (m), 1329 (m), 1358 (m), 1406 (m), 1439 (m), 1456 (s), 1566 (m), 1591 (m).

UV/Vis (C_3H_6O): λ_{max} (ε) = 383 (42800).

Anal calcd for $C_{23}H_{18}BrNO_2S$ [451.0]: C 61.07, H 4.01, N 3.10, S 7.09; Found: C 61.31, H 4.11, N 2.99, S 6.96.

(*Z*)-2-(3-Benzyl)benzo[*d*]thiazol-2(3*H*)-ylidene)-1-(4-(methoxyphenyl)ethan-1-one (3d)

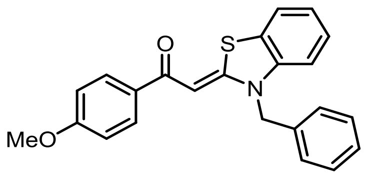

$C_{23}H_{19}NO_2S$

[373.11]

The synthesis was performed according to **GPII** to give 192 mg (0.515 mmol, 52%) of the desired product **3d** as a yellow solid.

Mp: 210 °C.

R_f (*n*-hexane/acetone 4:1): 0.21.

^{1}H NMR (300 MHz, acetone-d$_6$/CS$_2$ 5:1): δ 3.83 (s, 2 H), 5.52 (s, 2 H), 6.75 (s, 1 H), 6.90 (d, ^3J = 8.5 Hz, 2 H), 7.18-7.21 (m, 1 H), 7.27-7.36 (m, 8 H), 7.70 (d, ^3J = 7.6 Hz, 2 H), 7.88 (d, ^3J = 8.3 Hz, 2 H).

^{13}C NMR (75 MHz, acetone-d$_6$/CS$_2$ 5:1): δ 49.6 (CH_2), 55.6 (CH_3), 87.9 (CH), 111.1 (CH), 114.1 (CH), 123.0 (CH), 123.5 (CH), 127.2 (C_{quat}), 127.4 (CH), 128.1 (C_{quat}), 128.5 (CH), 129.6 (CH), 129.8 (CH), 133.2 (CH), 136.1 (C_{quat}), 137.9 (C_{quat}), 140.9 (C_{quat}), 161.7 (C_{quat}), 162.6 (C_{quat}), 183.4 (C_{quat}).

MALDI-TOF (*m/z*): 374.2 ($C_{23}H_{19}NO_2S+H^+$).

IR $\tilde{\nu}$ [cm^{-1}]: 613 (m), 658 (m), 692 (m), 718 (s), 729 (m), 745 (s), 768 (s), 793 (w), 812 (w), 820 (m), 841 (m), 849 (m), 874 (s), 907 (m), 924 (w), 970 (w), 108 (s), 1047 (m), 1065 (s), 1078 (m), 1092 (m), 1111 (m), 1132 (m), 1159 (m), 1175 (s), 1225 (s), 1254 (m), 1294 (m), 1308 (m), 1331 (m), 1352 (m), 1379 (m), 1395 (m), 1414 (m), 1437 (s), 1560 (m), 1597 (m), 2805 (w), 2832 (w), 2901 (m), 2924 (m), 2968 (m), 2986 (m), 3065 (w), 3653 (w), 3672 (w).

UV/Vis (C_3H_6O): λ_{max} (ε) = 384 (43500).

Anal calcd for $C_{23}H_{18}BrNO_2S$ [451.0]: C 73.97, H 5.13, N 3.75, S 8.59; Found: C 73.88, H 5.28, N 3.58, S 8.32.

(*Z*)-2-(3-(4-Bromobenzyl)benzo[*d*]thiazol-2(3*H*)-ylidene)-1-(4-*tert*-butylphenyl)ethan-1-one (3e)

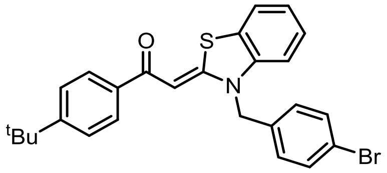

$C_{26}H_{24}BrNOS$

[477.08]

The synthesis was performed according to **GPII** to give 228 mg (0.478 mmol, 48%) of the desired product **3e** as a yellow solid.

Mp: 196 °C.

R$_f$ (*n*-hexane/acetone 4:1): 0.35.

^{1}H NMR (600 MHz, acetone-d$_6$/CS$_2$ 5:1): δ 1.34 (s, 9 H), 5.55 (s, 2 H), 6.80 (s, 1 H), 7.21-7.23 (m, 1 H), 7.27 (d, ^3J = 8.2 Hz, 2 H), 7.32-7.37 (m, 2 H), 7.43 (d, ^3J = 8.2 Hz, 2 H), 7.52 (d, ^3J = 8.5 Hz, 2 H), 7.73 (d, ^3J = 7.8 Hz, 1 H), 7.86 (d, ^3J = 7.8 Hz, 1 H).

^{13}C NMR (150 MHz, acetone-d$_6$/CS$_2$ 5:1): δ 31.6 (CH$_3$), 35.3 (C$_{quat}$), 49.1 (CH$_2$), 88.3 (CH), 111.5 (CH), 122.2 (CH), 123.2 (CH), 123.8 (CH), 125.8 (CH), 127.4 (CH), 127.9 (C$_{quat}$), 128.0 (CH), 129.5 (CH), 132.7 (CH), 135.5 (C$_{quat}$), 137.8 (C$_{quat}$), 140.7 (C$_{quat}$), 154.6 (C$_{quat}$), 161.9 (C$_{quat}$), 184.2 (C$_{quat}$).

MALDI-TOF (*m/z*): 480.0 ($C_{26}H_{24}{}^{81}BrNOS+H^+$), 478.0 ($C_{26}H_{24}{}^{79}BrNOS+H^+$).

IR $\tilde{\nu}$ [cm^{-1}]: 606 (m), 656 (m), 696 (m), 714 (s), 737 (s), 772 (s)812 (m), 843 (m), 881 (s), 1007 (m), 1020 (m), 1065 (m), 1101 (m), 1155 (m), 1180 (m), 1192 (m), 1234 (m), 1267 (m), 1296 (m), 1333 (m), 1362 (m), 1422 (m), 1433 (m), 1449 (s), 1468 (s), 1487 (s), 1514 (m), 1557 (m), 1599 (m).

UV/Vis (C_3H_6O): λ_{max} (ε) = 383 (44700).

Anal calcd for $C_{26}H_{24}BrNOS$ [477.1]: C 65.27, H 5.06, N 2.93, S 6.70; Found: C 65.56, H 5.10, N 2.89, S 6.59.

(*Z*)-2-(3-Benzyl)benzo[*d*]thiazol-2(3*H*)-ylidene)-1-(4-*tert*-butylphenyl)ethan-1-one (3f)

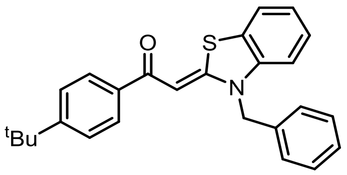

$C_{26}H_{25}NOS$

[399.17]

The synthesis was performed according to **GPII** to give 258 mg (0.647 mmol, 65%) of the desired product **3f** as a yellow solid.

Mp: 245 °C.

R_f (*n*-hexane/acetone 4:1): 0.37.

^{1}H NMR (600 MHz, acetone-d_6/CS_2 5:1): δ 1.35 (s, 9 H), 5.51 (s, 2 H), 6.73 (s, 1 H), 7.21 (t, ^3J = 7.8 Hz, 1 H), 7.27-7.30 (m, 4 H), 7.32-7.36 (m, 3 H), 7.40 (d, ^3J = 8.2 Hz, 2z H), 7.70 (d, ^3J = 7.7 Hz, 1 H), 7.81 (d, ^3J = 8.1 Hz, 2 H).

^{13}C NMR (150 MHz, acetone-d_6/CS_2 5:1): δ 31.6 (CH_3), 35.2 (C_{quat}), 49.7 (CH_2), 88.2 (CH), 111.1 (CH), 123.0 (CH), 123.6 (CH), 125.7 (CH), 127.2 (CH), 127.3 (CH), 127.8 (C_{quat}), 128.1 (CH), 128.5 (C_{quat}), 129.7 (CH), 135.9 (C_{quat}), 137.8 (C_{quat}), 140.8 (C_{quat}), 154.3 (C_{quat}), 161.9 (C_{quat}), 183.9 (C_{quat}).

MALDI-TOF (*m/z*): 400.1 ($C_{26}H_{25}NOS+H^+$).

IR $\tilde{\nu}$ [cm^{-1}]: 650 (m), 692 (m), 718 (s), 746 (s), 773 (s), 793 (m), 810 (m), 833 (m), 843 (m), 856 (m), 876 (m), 907 (m), 926 (w), 957 (w), 970 (w), 1001 (m), 1015 (m), 1045 (m), 1067 (m), 1092 (m), 1109 (m), 1157 (m), 1188 (m), 1202 (m), 1231 (m), 1267 (m), 1298 (m), 1333 (m), 1350 (m), 1379 (m), 1396 (m), 1447 (s), 1460 (s), 1545 (m), 1555 (m), 1595 (m), 2864 (w), 2903 (w), 2951 (w), 2961 (w).

UV/Vis (C_3H_6O): λ_{max} (ε) = 383 (42700).

Anal calcd for $C_{26}H_{25}NOS$ [399.2]: C 78.16, H 6.31, N 3.51, S 8.03; Found: C 78.29, H 6.43, N 3.38, S 8.02.

(*Z*)-2-(3-(4-Bromobenzyl)benzo[*d*]thiazol-2(3*H*)-ylidene)-1-(*p*-tolyl)ethan-1-one (3g)

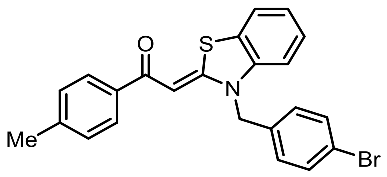

$C_{23}H_{18}BrNOS$

[435.03]

The synthesis was performed according to **GPII** to give 315 mg (0.724 mmol, 72%) of the desired product **3g** as a yellow solid.

Mp: 191 °C.

R_f (*n*-hexane/acetone 4:1): 0.40.

^{1}H NMR (600 MHz, acetone-d$_6$/CS$_2$ 5:1): δ 2.22 (s, 3 H), 5.43 (s, 2 H), 6.69 (s, 1 H), 7.06-7.13 (m, 5 H), 7.20-7.21 (m, 2 H), 7.38 (d, ^3J = 8.2 Hz, 2 H), 7.60 (d, ^3J = 7.7 Hz, 1 H), 7.71 (dd, ^3J = 7.9 Hz, ^4J = 1.5 Hz, 2 H).

^{13}C NMR (150 MHz, acetone-d$_6$/CS$_2$ 5:1): δ 21.6 (CH_3), 49.0 (CH_2), 88.2 (CH), 111.2 (CH), 122.0 (CH), 123.2 (CH), 123.8 (CH), 127.5 (CH), 128.0 (C_{quat}), 128.1 (CH), 129.5 (CH), 129.6 (CH), 132.8 (CH), 135.6 (C_{quat}), 137.8 (C_{quat}), 137.9 (C_{quat}), 140.7 (C_{quat}), 141.7 (C_{quat}), 162.0 (C_{quat}), 184.3 (C_{quat}).

MALDI-TOF (*m/z*): 438.1 ($C_{23}H_{18}{}^{81}BrNOS+H^+$), 436 .1 ($C_{23}H_{18}{}^{79}BrNOS+H^+$).

IR $\tilde{\nu}$ [cm^{-1}]: 617 (m), 662 (m), 679 (m), 700 (m), 719 (m), 741 (s), 750 (s), 781 (m), 802 (m), 822 (m), 881 (m), 920 (w), 937 (w), 962 (w), 997 (m), 1011 (m), 1043 (m), 1069 (m), 1090 (m), 1115 (m), 11134 (m), 1153 (m), 1175 (m), 1196 (m), 1227 (m), 1263 (w), 1294 (m), 1333 (m), 1395 (m), 1449 (m), 1466 (m), 1479 (m), 1514 (m), 1557 (m), 1591 (w), 1597 (m), 2855 (w), 2914 (w), 2943 (w), 3026 (w).

UV/Vis (C_3H_6O): λ_{max} (ε) = 382 (40300).

Anal calcd for $C_{23}H_{18}BrNOS$ [435.0]: C 63.31, H 4.16, N 3.21, S 7.35; Found: C 63.82, H 4.37, N 3.05, S 7.06.

(Z)-2-(3-Benzyl)benzo[d]thiazol-2(3H)-ylidene)-1-(p-tolyl)ethan-1-one (3h)

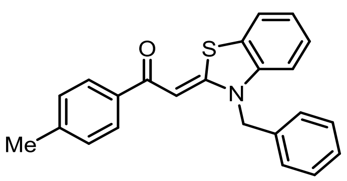

$C_{23}H_{19}NOS$

[357.12]

The synthesis was performed according to **GPII** to give 287 mg (0.804 mmol, 80%) of the desired product **3h** as a yellow solid.

Mp: 176 °C.

R_f (n-hexane/acetone 4:1): 0.22.

^{1}H NMR (600 MHz, acetone-d_6/CS_2 5:1): δ 2.36 (s, 3 H), 5.56 (s, 2 H), 6.82 (s, 1 H), 7.19-7.36 (m, 10 H), 7.73 (d, ^3J = 7.8 Hz, 1 H), 7.84 (d, ^3J = 7.8 Hz, 2 H).

^{13}C NMR (150 MHz, acetone-d_6/CS_2 5:1): δ 21.6 (CH_3), 49.7 (CH_2), 88.2 (CH), 111.3 (CH), 123.1 (CH), 123.7 (CH), 127.4 (CH), 128.0 (C_{quat}), 128.5 (CH), 129.6 (CH), 129.7 (CH), 132.6 (CH), 136.2 (C_{quat}), 137.9 (C_{quat}), 141.0 (C_{quat}), 141.6 (C_{quat}), 162.1 (C_{quat}), 184.2 (C_{quat}).

MALDI-TOF (m/z): 358.2 ($C_{23}H_{19}NOS+H^+$).

IR $\tilde{\nu}$ [cm^{-1}]: 615 (w), 657 (w), 681 (w), 694 (m), 735 (m), 752 (s), 822 (w), 876 (m), 908 (m), 970 (w), 1022 (w), 1067 (m), 1092 (w), 1115 (w), 1136 (w), 1155 (w), 1177 (m), 1198 (m), 1234 (m), 1265 (w), 1296 (w), 1331 (m), 1395 (w), 1406 (w), 1441 (m), 1466 (m), 1477 (m), 1512 (w), 1560 (m), 1595 (w), 2879 (w), 2990 (w), 3057 (w), 3906 (w).

UV/Vis (C_3H_6O): λ_{max} (ε) = 382 (40000).

Anal calcd for $C_{23}H_{19}NOS$ [357.1]: C 77.28, H 5.36, N 3.92, S 8.97; Found: C 77.55, H 5.46, N 3.82, S 8.67.

(*Z*)-2-(3-(4-Bromobenzyl)benzo[*d*]thiazol-2(3*H*)-ylidene)-1-phenylethan-1-one (3i)

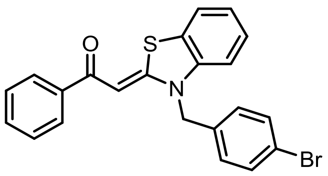

$C_{22}H_{16}BrNOS$

[421.01]

The synthesis was performed according to **GPII** to give 273 mg (0.648 mmol, 65%) of the desired product **3i** as a yellow solid.

Mp: 176 °C (decomposition).

R*f* (*n*-hexane/acetone 4:1): 0.18.

^{1}H NMR (300 MHz, acetone-d$_6$): δ 5.57 (s, 2 H), 6.83 (s, 1 H), 7.22-7.27 (m, 3 H), 7.32-7.43 (m, 5 H), 7.51 (d, ^3J = 8.3 Hz, 2 H), 7.73 (d, ^3J = 8.3 Hz, 1 H), 7.93 (d, ^3J = 8.1 Hz, 2 H).

^{13}C NMR (75 MHz, acetone-d$_6$): δ 49.1 (CH_2), 88.2 (CH), 111.2 (CH), 122.2 (C_{quat}), 123.2 (CH), 123.8 (CH), 127.5 (CH), 127.9 (CH), 128.0 (C_{quat}), 128.9 (CH), 129.5 (CH), 131.5 (CH), 132.7 (CH), 135.4 (C_{quat}), 140.4 (C_{quat}), 140.7 (C_{quat}), 162.3 (C_{quat}), 184.3 (C_{quat}).

EI + MS (70 eV, *m/z* (%)): 423 ($[C_{22}H_{16}{}^{81}BrNOS]^+$, 30), 421 ($[C_{22}H_{16}{}^{79}BrNOS]^+$, 30), 406 ($[C_{22}H_{15}{}^{81}BrNS]^+$, 13), 404 ($[C_{22}H_{15}{}^{79}BrNS]^+$, 12), 318 ($[C_{15}H_{11}{}^{81}BrNS]^+$, 25), 316 ($[C_{15}H_{11}{}^{79}BrNS]^+$, 27), 237 (19), 236 ($[C_{15}H_{10}N^{32}S]^+$, 28), 225 (11), 224 (40), 223 ($[C_{14}H_9NS]^+$, 49), 171 ($[C_7H_6{}^{81}Br]^+$, 61), 169 ($[C_7H_6{}^{81}Br]^+$, 62), 105 ($[C_7H_6O]^+$, 100), 90 ($[C_7H_6]^+$, 30), 89 ($[C_7H_5]^+$, 21), 77 ($[C_6H_5]^+$, 29).

IR $\tilde{\nu}$ [cm^{-1}]: 629 (w), 652 (m), 677 (m). 702 (m), 714 (s), 739 (m), 772 (w), 789 (m), 810 (m), 830 (m), 843 (w), 878 (m), 920 (w), 935 (w), 953 (w), 1001 (m), 1045 (m), 1059 (m), 1090 (m), 1111 (w), 1134 (w),1153 (m), 1177 (m), 1198 (m), 1227 (m), 1261 (w), 1275 (w), 1292 (m), 1306 (m), 1341 (m), 1396 (m), 1433 (m), 1449 (m), 1481 (s), 1555 (w), 1566 (m), 1601 (m).

UV/Vis (C_3H_6O): λ_{max} (ε) = 376 (58300).

Anal calcd for $C_{22}H_{16}BrNOS$ [421.1]: C 62.57, H 3.82, N 3.32, S 7.59; Found: C 62.54, H 3.71, N 3.15, S 7.46.

(*Z*)-2-(3-Benzylbenzo[*d*]thiazol-2(3*H*)-ylidene)-1-phenylethan-1-one (3j)

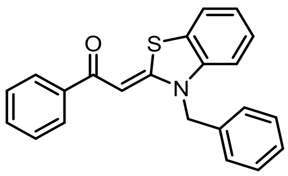

$C_{22}H_{17}NOS$

[343.10]

The synthesis was performed according to **GPII** to give 1.98 g (5.77 mmol, 58%) of the desired product **3j** as a yellow solid.

Mp: 161 °C (decomposition).

R_f (*n*-hexane/acetone 4:1): 0.26.

^{1}H NMR (300 MHz, acetone-d$_6$/CS$_2$ 5:1): δ 5.59 (s, 2 H), 6.88 (s, 1 H), 7.20-7.52 (m, 11 H), 7.76 (d, ^3J = 8.3 Hz, 1 H), 7.96 (dd, ^3J = 8.0 Hz, ^4J = 1.4 Hz, 2 H).

^{13}C NMR (75 MHz, acetone-d$_6$/CS$_2$ 5:1): δ 49.8 (CH_2), 88.3 (CH), 111.6 (CH), 123.3 (CH), 123.9 (CH), 127.5 (CH), 127.6 (CH), 128.0 (C_{quat}), 128.1 (CH), 128.6 (CH), 129.1 (CH), 129.8 (CH), 131.6 (CH), 136.3 (C_{quat}), 140.6 (C_{quat}), 141.0 (C_{quat}), 162.6 (C_{quat}), 184.5 (C_{quat}).

MALDI-TOF (*m/z*): 344.1 ($C_{22}H_{17}NOS+H^+$).

IR $\tilde{\nu}$ [cm^{-1}]: 662 (m), 671 (m), 700 (m), 716 (s), 731 (s), 743 (s), 800 (m), 812 (m), 841 (m), 874 (m), 910 (m), 926 (m), 964 (m), 976 (m), 999 (m), 1022 (m), 1032 (m), 1061 (m), 1096 (m), 1179 (m), 1198 (s), 1227 (m), 1252 (m), 1292 (m), 1300 (m), 1344 (m), 1395 (m), 1418 (m), 1431 (s), 1447 (s), 1468 (s), 1477 (s), 1558 (s), 1593 (m).

UV/Vis (C_3H_6O): λ_{max} (ε) = 377 (39100).

Anal calcd for $C_{22}H_{17}NOS$ [343.1]: C 76.94, H 4.99, N 4.08, S 9.33; Found: C 77.21, H 5.09, N 4.04, S 9.13.

(*Z*)-2-(3-Methyl)benzo[*d*]thiazol-2(3*H*)-ylidene)-1-phenylethan-1-one (3k)[5]

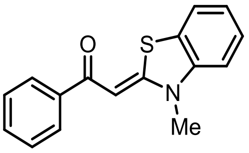

$C_{16}H_{13}NOS$

[267.07]

The synthesis was performed according to **GPII** to give 250 mg (0.936 mmol, 94%) of the desired product **3k** as a yellow solid.

Mp: 178 °C.

R_f (*n*-hexane/acetone 4:1): 0.27.

^{1}H NMR (600 MHz, acetone-d$_6$/CS$_2$ 5:1): δ 3.79 (s), 6.78 (s, 1 H), 7.19-7.22 (m, 1 H), 7.38-7.48 (m, 6 H), 8.05 (d, ^3J = 6.9 Hz, 2 H).

^{13}C NMR (150 MHz, acetone-d$_6$/CS$_2$ 5:1): δ 33.1 (CH_3), 87.7 (CH), 111.1 (CH), 123.0 (CH), 123.4 (CH), 127.3 (CH), 127.8 (C_{quat}), 128.0 (CH), 129.0 (C_{quat}), 131.4 (CH), 140.7 (C_{quar}), 141.2 (C_{quat}), 162.5 (C_{quat}), 184.1 (C_{quat}).

MALDI-TOF (*m/z*): 268.2 ($C_{16}H_{13}NOS+H^+$).

IR $\tilde{\nu}$ [cm^{-1}]: 617 (m), 664 (m), 687 (s), 718 (s), 746 (m), 799 (m), 847 (m), 885 (m), 926 (m), 970 (m), 989 (m), 999 (m), 1022 (m), 1038 (m), 1061 (m), 1086 (m), 1101 (m), 1134 (m), 1153 (m), 1179 (m), 1217 (m), 1260 (m), 1288 (m), 1323 (m), 1346 (m), 1393 (m), 1418 (m), 1441 (m), 1456 (s), 1476 (s), 1564 (m), 1591 (m), 1722 (w), 1938 (w), 2479 (w), 2509 (w), 2540 (w), 2590 (w), 2679 (w), 2733 (w), 2791 (w), 2818 (w), 2855 (w), 2926 (w), 2957 (w), 3038 (w), 3080 (w), 3157 (w).

UV/Vis (C_3H_6O): λ_{max} (ε) = 382 (41400).

Anal calcd for $C_{16}H_{13}NOS$ [267.1]: C 71.88, H 4.90, N 5.24, S 11.99; Found: C 71.59, H 4.84, N 5.23, S 12.12.

(1*Z*)(3*E*)-2-(3-(4-Bromobenzyl)benzo[*d*]thiazol-2(3*H*)-ylidene)-1-(4-fluorophenyl)but-3-en-2-one (3l)

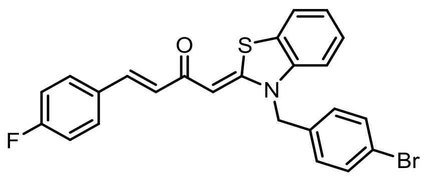

$C_{24}H_{17}BrFNOS$

[465.02]

The synthesis was performed according to **GPII** to give 100 mg (0.215 mmol, 22%) of the desired product **3l** as an orange solid.

Mp: 198 °C.

R_f (*n*-hexane/acetone 4:1): 0.18.

^{1}H NMR (300 MHz, acetone-d_6/CS_2 5:1): δ 5.46 (s, 2 H), 6.83 (d, ^3J = 7.2 Hz, 1 H), 7.08-7.16 (m, 2 H), 7.20-7.25 (m, 3 H), 7.28-7.39 (m, 2 H), 7.45 (s, 1 H), 7.50-7.55 (m, 2 H), 7.58-7.65 (m, 2 H), 7.73 (d, ^3J = 7.9 Hz, 1 H).

^{13}C NMR (75 MHz, acetone-d_6/CS_2 5:1): δ 49.2 (CH_2), 92.8 (CH), 111.3 (CH), 116.3 (CH), 116.6 (CH), 122.3 (C_{quat}), 123.3 (CH), 123.9 (CH), 127.5 (CH), 128.3 (C_{quat}), 129.2 (CH), 129.5 (CH), 130.5 (CH), 130.6 (CH), 132.8 (CH), 133.5 (C_{quat}), 135.4 (C_{quat}), 137.1 (C_{quat}), 140.7 (C_{quat}), 162.4 (C_{quat}), 165.7 (C_{quat}), 182.7 (C_{quat}).

MALDI-TOF (*m/z*): 468.0 ($C_{24}H_{17}{}^{81}BrFNOS+H^+$), 466.0 ($C_{24}H_{17}{}^{79}BrFNOS+H^+$).

IR $\tilde{\nu}$ [cm^{-1}]: 743 (s), 802 (m), 826 (s), 970 (m), 1011 (m), 1070 (m), 1128 (m), 1136 (m), 1159 (m), 1229 (m), 1267 (m), 1329 (m), 1342 (m), 1400 (m), 1460 (s), 1508 (m), 1582 (m), 2887 (m), 3676 (m), 3784 (m), 3854 (m).

UV/Vis (C_3H_6O): λ_{max} (ε) = 410 (41400).

Anal calcd for $C_{24}H_{17}BrFNOS$ [465.0]: C 61.81, H 3.67, N 3.00, S 6.88; Found: C 61.55, H 3.82, N 2.90, S 6.63.

(1Z)(3E)-2-(3-Benzyl)benzo[d]thiazol-2(3H)-ylidene)-1-(4-fluorophenyl)but-3-en-2-one (3m)

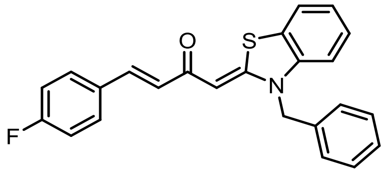

$C_{24}H_{18}FNOS$

[387.11]

The synthesis was performed according to **GPII** to give 121 mg (0.313 mmol, 31%) of the desired product **3m** as an orange solid.

Mp: 133 °C.

R_f (*n*-hexane/acetone 4:1): 0.28.

^{1}H NMR (300 MHz, acetone-d$_6$/CS$_2$ 5:1): δ 5.48 (s, 2 H), 6.31 (s, 1 H), 6.85 (d, ^3J = 7.2 Hz, 1 H), 7.09-7.16 (m, 2 H), 7.21-7.26 (m, 3 H), 7.32-7.37 (m, 2 H), 7.46 (s, 1 H), 7.51-7.55 (m, 2 H), 7.61-7.65 (m, 3 H), 7.74 (d, ^3J = 7.9 Hz, 1 H).

^{13}C NMR (75 MHz, acetone-d$_6$/CS$_2$ 5:1): δ 49.2 (CH_2), 92.8 (CH), 111.3 (CH), 116.3 (CH), 116.6 (CH), 122.3 (C_{quat}), 123.3 (CH), 123.9 (CH), 127.5 (CH), 128.3 (C_{quat}), 129.2 (CH), 129.5 (CH), 130.5 (CH), 130.6 (CH), 132.8 (CH), 133.5 (C_{quat}), 135.4 (C_{quat}), 137.1 (C_{quat}), 140.7 (C_{quat}), 162.4 (C_{quat}), 165.7 (C_{quat}), 182.7 (C_{quat}).

MALDI-TOF (*m/z*): 388.1 ($C_{24}H_{18}FNOS+H^+$).

IR $\tilde{\nu}$ [cm^{-1}]: 637 (m), 696 (s), 721 (m), 729 (s), 743 (s), 785 (m), 827 (s), 864 (w), 935 (m), 982 (m), 1015 (m), 1024 (m), 1043 (m), 1072 (m), 1094 (m), 1126 (m), 1157 (s), 1179 (m), 1229 (s), 1267 (m), 1287 (m), 1342 (m), 1395 (m), 1435 (m), 1450 (s), 1466 (s), 1477 (s), 1506 (s), 1574 (m), 1599 (m), 1628 (m), 1688 (m), 1697 (m), 2901 (w), 2922 (w), 2965 (w), 2986 (w), 3061 (w).

UV/Vis (C_3H_6O): λ_{max} (ε) = 411 (11000).

Anal calcd for $C_{24}H_{18}FNOS$ [387.1]: C 74.39, H 4.68, N 3.61, S 8.28; Found: C 74.14, H 4.96, N 3.72, S 8.10.

(Z)-2-(3-(4-Bromobenzyl)benzo[d]thiazol-2(3H)-ylidene)-1-(4-fluoro)phenylethan-1-one (3n)

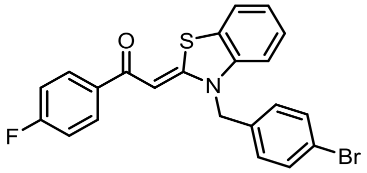

$C_{22}H_{15}BrFNOS$

[439.00]

The synthesis was performed according to **GPII** to give 304 mg (0.692 mmol, 69%) of the desired product **3n** as a yellow solid.

Mp: 181 °C.

R_f (*n*-hexane/acetone 4:1): 0.30.

^{1}H NMR (600 MHz, acetone-d$_6$/CS$_2$ 5:1): δ 5.58 (s, 2 H), 6.84 (s, 1 H), 7.11 (t, ^3J = 8.2 Hz, 2 H), 7.22-7.27 (m, 3 H), 7.33-7.37 (m, 2 H), 7.52 (d, ^3J = 8.2 Hz, 2 H), 7.79 (d, ^3J = 8.2 Hz, 1 H), 7.99-8.02 (m, 2 H).

^{13}C NMR (150 MHz, acetone-d$_6$/CS$_2$ 5:1): δ 49.1 (CH_2), 87.9 (CH), 111.3 (CH), 115.6 (CH), 115.7 (CH), 122.2 (C_{quat}), 123.2 (CH), 123.9 (CH), 127.5 (CH), 127.9 (C_{quat}), 129.5 (CH), 130.4 (CH), 132.7 (CH), 135.4 (C_{quat}), 136.8 (C_{quat}), 140.7 (C_{quat}), 162.5 (C_{quat}), 164.2 (C_{quat}), 165.9 (C_{quat}), 182.9 (C_{quat}).

MALDI-TOF (*m/z*): 442.0 ($C_{22}H_{15}{}^{81}BrFNOS+H^+$), 440.0 ($C_{22}H_{15}{}^{79}BrFNOS+H^+$).

IR $\tilde{\nu}$ [cm^{-1}]: 615 (m), 664 (m), 679 (m), 700 (m), 710 (m), 721 (m), 743 (s), 764 (s), 787 (m), 797 (m), 816 (m), 829 (m), 835 (m), 851 (m), 881 (m), 922 8w), 945 (w), 974 (w), 1009 (m), 1043 (m), 1063 (m), 1134 (m), 1152 (m), 1186 (m), 1196 (m), 1223 (m), 1265 (w), 1290 (m), 1329 (m), 1342 (m), 1398 (m), 1445 (s), 1468 (s), 1479 (s), 1510 (m), 1566 (m), 1578 (m), 1603 (m), 1892 (w), 2947 (w), 3026 (w).

UV/Vis (C_3H_6O): λ_{max} (ε) = 381 (41100).

Anal calcd for $C_{22}H_{15}BrFNOS$ [439.0]: C 60.01, H 3.43, N 3.18, S 7.28; Found: C 60.33, H 3.38, N 3.12, S 7.44.

(*Z*)-2-(3-Benzyl)benzo[*d*]thiazol-2(3*H*)-ylidene)-1-(4-fluoro)phenylethan-1-one (3o)

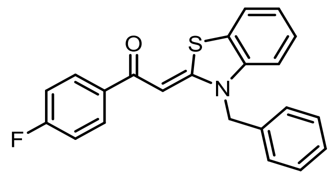

$C_{22}H_{16}FNOS$

[361.09]

The synthesis was performed according to **GPII** to give 306 mg (0.847 mmol, 85%) of the desired product **3o** as a yellow solid.

Mp: 155 °C.

R_f (*n*-hexane/acetone 4:1): 0.29.

^{1}H NMR (600 MHz, acetone-d_6/CS_2 5:1): δ 5.57 (s, 2 H), 6.83 (s, 1 H), 7.11 (t, ^3J = 8.2 Hz, 2 H), 7.21-7.24 (m, 1 H), 7.26-7.30 (m, 3 H), 7.32-7.36 (m, 4 H), 7.73 (d, ^3J = 8.2 Hz, 1 H), 7.98-8.02 (m, 2 H).

^{13}C NMR (150 MHz, acetone-d_6/CS_2 5:1): δ 49.7 (CH_2), 87.9 (CH), 111.4 (CH), 115.6 (CH), 115.7 (CH), 123.1 (C_{quat}), 123.8 (CH), 127.4 (CH), 127.9 (C_{quat}), 128.5 (CH), 129.7 (CH), 130.3 (CH), 130.4 (CH), 132.7 (C_{quat}), 136.0 (C_{quat}), 136.9 (C_{quat}), 140.8 (C_{quat}), 162.6 (C_{quat}), 164.2 (C_{quat}), 165.9 (C_{quat}), 182.8 (C_{quat}).

MALDI-TOF (*m/z*): 362.1 ($C_{22}H_{16}FNOS+H^+$).

IR $\tilde{\nu}$ [cm^{-1}]: 610 (m), 654 (m), 689 (m), 710 (m), 743 (s), 768 (s), 820 (w), 854 (m), 880 (m), 903 (m), 1003 (w), 1026 (m), 1043 (m), 1067 (m), 1090 (m), 1101 (w), 1138 (m), 1150 (s), 1196 (m), 1219 (m), 1225 (m), 1269 (w), 1304 (m), 1331 (m), 1377 (w), 1414 (m), 1449 (s), 1474 (s), 1578 (m), 1595 (m), 2359 (w), 3030 (w), 3061 (w).

UV/Vis (C_3H_6O): λ_{max} (ε) = 381 (47200).

Anal calcd for $C_{22}H_{16}FNOS$ [439.0]: C 73.11, H 4.46, N 3.88, S 8.87; Found: C 73.22, H 4.46, N 3.80, S 8.87.

(*Z*)-2-(3-Benzyl)benzo[*d*]thiazol-2(3*H*)-ylidene)-1-(3-fluoro)phenylethan-1-one (3p)

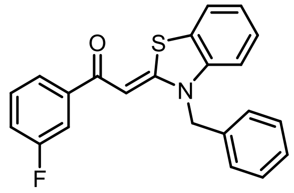

$C_{22}H_{16}FNOS$

[361.09]

The synthesis was performed according to **GPII** to give 234 mg (0.648 mmol, 65%) of the desired product **3p** as a yellow solid.

Mp: 143 °C.

R_f (*n*-hexane/acetone 4:1): 0.43.

^{1}H NMR (600 MHz, acetone-d_6/CS_2 5:1): δ 5.60 (s, 2 H), 6.86 (s, 1 H), 7.11 (tdd, ^3J = 8.3 Hz, ^4J = 2.7 Hz, ^5J = 1.0 Hz, 1 H), 7.23-7.26 (m, 1 H), 7.27-7.31 (m, 3 H), 7.33-7.37 (m, 4 H), 7.41-7.43 (m, 1 H), 7.66 (d, ^3J = 10.1 Hz, 1 H), 7.76 (d, ^3J = 7.9 Hz, 2 H).

^{13}C NMR (150 MHz, acetone-d_6/CS_2 5:1): δ 49.7 (CH_2), 88.1 (CH), 111.6 (CH), 114.4 (CH), 118.1 (CH), 123.1 (C_{quat}), 123.8 (CH), 127.4 (CH), 127.5 (CH), 127.9 (C_{quat}), 128.5 (CH), 129.7 (CH), 130.3 (CH), 130.7 (C_{quat}), 132.7 (C_{quat}), 135.9 (C_{quat}), 136.0 (C_{quat}), 143.0 (C_{quat}), 162.8 (C_{quat}), 163.2 (C_{quat}), 164.4 (C_{quat}), 182.5 (C_{quat}).

MALDI-TOF (*m/z*): 363.1 ($C_{22}H_{16}FNOS$+2 H^+), 362.1 ($C_{22}H_{16}FNOS$+H^+).

IR $\tilde{\nu}$ [cm^{-1}]: 642 (w), 673 (m), 694 (m), 739 (s), 752 (s), 795 (m), 839 (s), 876 (m), 903 (w), 934 (m), 968 (w), 995 (w), 1024 (m), 1045 (m), 1057 (m), 1076 (w), 1088 (w), 1109 (w), 1150 (m), 1175 (s), 1213 (m), 1238 (m), 1271 (m), 1300 (m), 1329 (m), 1341 (m), 1423 (s), 1441 (s), 1466 (s), 1493 (s), 1562 (m), 1572 (m), 1603 (w), 2208 (w), 3030 (w), 3063 (w).

UV/Vis (C_3H_6O): λ_{max} (ε) = 387 (74700).

Anal calcd for $C_{22}H_{16}FNOS$ [439.0]: C 73.11, H 4.46, N 3.88, S 8.87; Found: C 73.10, H 4.55, N 3.83, S 9.03.

(*Z*)-2-(3-Benzyl)benzo[*d*]thiazol-2(3*H*)-ylidene)-1-(2-fluoro)phenylethan-1-one (3q)

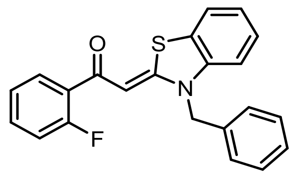

$C_{22}H_{16}FNOS$

[361.09]

The synthesis was performed according to **GPII** to give 175 mg (0.485 mmol, 49%) of the desired product **3q** as a yellow solid.

Mp: 152 °C (decomposition).

R_f (*n*-hexane/acetone 4:1): 0.42.

^{1}H NMR (300 MHz, acetone-d$_6$/CS$_2$ 5:1): δ 5.51 (s, 2 H), 6.70 (s, 1 H), 7.07-7.14 (m, 1 H), 7.20-7.38 (m, 7 H), 7.40-7.48 (m, 3 H), 7.77 (d, ^3J = 7.7 Hz, 1 H), 7.89 (td, ^3J = 7.8 Hz, ^3J = 2.0 Hz, 1 H).

^{13}C NMR (75 MHz, acetone-d$_6$/CS$_2$ 5:1): δ 49.7 (CH_2), 92.8 (CH), 111.6 (CH), 116.7 (CH), 117.0 (CH), 123.3 (C_{quat}), 124.0 (CH), 125.0 (CH), 125.1 (CH), 127.5 (CH), 127.6 (CH), 128.1 (C_{quat}), 128.7 (CH), 129.8 (CH), 131.7 (CH), 131.8 (C_{quat}), 132.9 (C_{quat}), 135.9 (C_{quat}), 159.8 (C_{quat}), 162.4 (C_{quat}), 163.1 (C_{quat}), 180.6 (C_{quat}).

MALDI-TOF (*m/z*): 362.1 ($C_{22}H_{16}FNOS+H^+$).

IR $\tilde{\nu}$ [cm^{-1}]: 658 (m), 683 (w), 704 (m, 719 (m), 739 (s), 762 (s), 826 (m), 880 (m), 920 (w9, 1022 (m), 1040 (w), 1084 (w), 1109 (m), 1152 (m), 1190 (m), 1204 (m), 1217 (w), 1261 (m), 1300 (m), 1312 (m), 1333 (m), 1352 (m), 1373 (w), 1410 (m), 1429 (m), 1439 (m), 1468 (s), 1558 (m), 1591 (m), 3647 (w), 3674 (w), 3688 (w), 3836 (w).

UV/Vis (C_3H_6O): λ_{max} (ε) = 383 (85400).

Anal calcd for $C_{22}H_{16}FNOS$ [439.0]: C 73.11, H 4.46, N 3.88, S 8.87; Found: C 72.87, H 4.51, N 3.83, S 8.66.

(*Z*)-2-(3-(4-Bromobenzyl)benzo[*d*]thiazol-2(3*H*)-ylidene)-1-(4-chloro)phenylethan-1-one (3r)

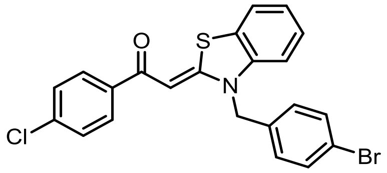

$C_{22}H_{15}BrClNOS$

[454.97]

The synthesis was performed according to **GPII** to give 376 mg (0.826 mmol, 83%) of the desired product **3r** as a yellow solid.

Mp: 215 °C.

R$_f$ (*n*-hexane/acetone 4:1): 0.22.

^{1}H NMR (300 MHz, acetone-d$_6$/CS$_2$ 5:1): δ 5.59 (s, 2 H), 6.86 (s, 1 H), 7.22-7.29 (m, 3 H), 7.34-7.43 (m, 4 H), 7.52 (d, ^3J = 8.5 Hz, 2 H), 7.76 (d, ^3J = 7.7 Hz, 1 H), 7.93-7.98 (m, 2 H).

^{13}C NMR (75 MHz, acetone-d$_6$/CS$_2$ 5:1): δ 49.3 (CH$_2$), 88.1 (CH), 111.5 (CH), 122.3 (C$_{quat}$), 123.3 (CH), 124.0 (CH), 127.6 (CH), 128.1 (C$_{quat}$), 129.1 (CH), 129.6 (CH), 129.7 (CH), 132.9 (CH), 135.5 (C$_{quat}$), 137.3 (C$_{quat}$), 139.2 (C$_{quat}$), 140.8 (C$_{quat}$), 162.9 (C$_{quat}$), 183.0 (C$_{quat}$).

MALDI-TOF (*m/z*): 460.0 ($C_{22}H_{15}{}^{81}Br^{37}ClNOS+H^+$), 458.0 ($C_{22}H_{15}{}^{81}Br^{35}ClNOS+H^+$, $C_{22}H_{15}{}^{79}Br^{37}ClNOS+H^+$), 456.0 ($C_{22}H_{15}{}^{79}Br^{35}ClNOS+H^+$).

IR $\tilde{\nu}$ [cm^{-1}]: 629 (m), 660 (m), 673 (m), 689 (m), 696 (m), 708 (m), 719 (m), 739 (s), 754 (s), 789 (m), 800 (m), 818 (m), 837 (m), 880 (m), 908 (w), 924 (w), 961 (w), 997 (m), 1009 (m), 1022 (m), 1043 (m), 1069 (m), 1090 (m), 1134 (m), 1155 (m), 1173 (m), 1196 (m), 1231 (m), 1263 (m), 1290 (m), 1308 (m), 1331 (m), 1389 (m), 1433 (m), 1449 (s), 1466 (s), 1477 (m), 1497 (m), 1560 (m), 1589 (m), 2886 (w), 2945 (w), 2988 (w).

UV/Vis (C_3H_6O): λ_{max} (ε) = 386 (39000).

Anal calcd for $C_{22}H_{15}BrClNOS$ [455.0]: C 57.85, H 3.31, N 3.07, S 7.02; Found: C 57.71, H 3.42, N 3.03, S 6.89.

(*Z*)-2-(3-Benzyl)benzo[*d*]thiazol-2(3*H*)-ylidene)-1-(4-chloro)phenylethan-1-one (3s)

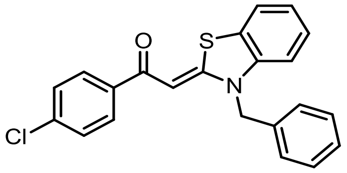

$C_{22}H_{16}ClNOS$

[377.06]

The synthesis was performed according to **GPII** to give 332 mg (0.881 mmol, 88%) of the desired product **3s** as a yellow solid.

Mp: 199 °C.

R_f (*n*-hexane/acetone 4:1): 0.29.

^{1}H NMR (300 MHz, acetone-d_6/CS_2 5:1): δ 5.59 (s, 2 H), 6.87 (s, 1 H), 7.20-7.45 (m, 11 H), 7.76 (d, ^3J = 7.5 Hz, 1 H), 7.93-7.98 (m, 2 H).

^{13}C NMR (75 MHz, acetone-d_6/CS_2 5:1): δ 49.9 (CH_2), 88.1 (CH), 111.6 (CH), 123.2 (CH), 123.9 (CH), 127.5 (CH), 128.1 (C_{quat}), 129.1 (CH), 129.7 (CH), 129.8 (CH), 136.1 (C_{quat}), 137.2 (C_{quat}), 139.3 (C_{quat}), 141.0 (C_{quat}), 163.0 (C_{quat}), 182.9 (C_{quat}).

MALDI-TOF (*m/z*): 380.1 ($C_{22}H_{16}{}^{37}ClNOS+H^+$), 378.1 ($C_{22}H_{16}{}^{35}ClNOS+H^+$).

IR $\tilde{\nu}$ [cm^{-1}]: 644 (m), 669 (w), 698 (s), 731 (s), 800 (w), 841 (m), 876 (m), 903 (m), 947 (w), 970 (w), 1013 (m), 1022 (m), 1042 (m), 1067 (m), 1092 (m), 1107 (w), 1136 (w), 1161 (m), 1169 (m), 1198 (s), 1229 (m), 1265 (m), 1304 (m), 1329 (m), 1339 (m), 1362 (m), 1389 (m), 1406 (m), 1445 (s), 1560 (m), 1589 (m), 2560 (w), 2967 (w), 3034 (w), 3057 (w).

UV/Vis (C_3H_6O): λ_{max} (ε) = 387 (40000).

Anal calcd for $C_{22}H_{16}ClNOS$ [377.1]: C 69.92, H 4.27, N 3.71, S 8.49; Found: C 70.16, H 4.22, N 3.63, S 8.64.

(*Z*)-2-(3-(4-Bromobenzyl)benzo[*d*]thiazol-2(3*H*)-yliden)-1-(6-chloropyridine-3-yl)phenyl ethan-1-one (3t)

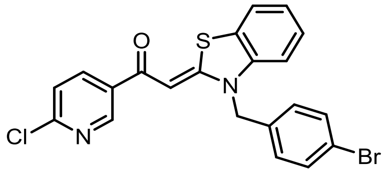

$C_{21}H_{14}BrClN_2OS$

[455.97]

The synthesis was performed according to **GPII** to give 124 mg (0.272 mmol, 27%) of the desired product **3t** as a yellow solid.

Mp: 222 °C.

R_f (*n*-hexane/acetone 4:1): 0.21.

^{1}H NMR (300 MHz, acetone-d$_6$/CS$_2$ 5:1): δ 5.63 (s, 2 H), 6.91 (s, 1 H), 7.25-7.30 (m, 3 H), 7.39-7.54 (m, 4 H), 7.79 (d, ^3J = 7.8 Hz, 1 H), 8.28 (dd, ^3J = 8.3 Hz, ^{4}J = 2.5 Hz, 1 H), 8.90 (d, ^3J = 2.5 Hz, 1 H).

^{13}C NMR (75 MHz, acetone-d$_6$/CS$_2$ 5:1): δ 49.3 (CH$_2$), 88.2 (CH), 111.8 (CH), 122.4 (C$_{quat}$), 123.4 (CH), 124.3 (CH), 124.7 (CH), 127.8 (CH), 128.0 (C$_{quat}$), 129.6 (CH), 129.7 (CH), 132.9 (CH), 134.7 (C$_{quat}$), 135.3 (C$_{quat}$), 138.5 (CH), 140.6 (C$_{quat}$), 149.7 (CH), 153.7 (C$_{quat}$), 163.4 (C$_{quat}$), 181.2 (C$_{quat}$).

MALDI-TOF (*m/z*): 461.0 ($C_{21}H_{14}{}^{81}Br^{37}ClN_2OS+H^+$), 459.0 ($C_{21}H_{14}{}^{81}Br^{35}ClN_2OS+H^+$, $C_{21}H_{14}{}^{79}Br^{37}ClN_2OS+H^+$), 457.0 ($C_{21}H_{14}{}^{79}Br^{35}ClN_2OS+H^+$).

IR $\tilde{\nu}$ [cm^{-1}]: 660 (m), 691 (m), 719 (m), 741 (s), 760 (s), 791 (m), 820 (m), 835 (m), 881 (s), 997 (m), 1043 (m), 1069 (m), 1109 (m), 1198 (m), 1233 (m), 1267 (m), 1296 (m), 1339 (m), 1362 (m), 1396 (m), 1447 (s), 1468 (m), 1489 (s), 1551 (m).

UV/Vis (C_3H_6O): λ_{max} (ε) = 391 (38400).

Anal calcd for $C_{21}H_{14}BrClN_2OS$ [455.0]: C 55.10, H 3.08, N 6.12, S 7.00; Found: C 55.37, H 3.15, N 5.95, S 7.01.

(*Z*)-2-(3-Benzyl)benzo[*d*]thiazol-2(3*H*)-ylidene)-1-(6-chloropyridine-3-yl)phenylethan-1-one (3u)

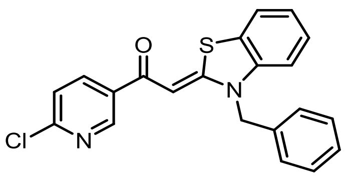

$C_{21}H_{15}ClN_2OS$

[378.06]

The synthesis was performed according to **GPII** to give 100 mg (0.265 mmol, 27%) of the desired product **3u** as a yellow solid.

Mp: 243 °C.

R_f (*n*-hexane/acetone 4:1): 0.25.

^{1}H NMR (600 MHz, acetone-d$_6$/CS$_2$ 5:1): δ 5.65 (s, 2 H), 6.94 (s, 1 H), 7.24-7.37 (m, 6 H), 7.40-7.48 (m, 3 H), 7.82 (d, ^3J = 7.6 Hz, 1 H), 8.30 (dd, ^3J = 8.4 Hz, ^4J = 2.3 Hz, 1 H), 8.92 (d, ^3J = 2.5 Hz, 1 H).

^{13}C NMR (150 MHz, acetone-d$_6$/CS$_2$ 5:1): δ 49.8 (CH$_2$), 88.1 (CH), 111.9 (CH), 123.4 (CH), 124.2 (CH), 124.7 (CH), 127.5 (CH), 127.7 (CH), 128.6 (CH), 129.8 (CH), 134.8 (C_{quat}), 135.3 (C_{quat}), 138.5 (CH), 140.8 (C_{quat}), 149.7 (CH), 153.5 (C_{quat}), 163.5 (C_{quat}), 180.9 (C_{quat}).

MALDI-TOF (*m/z*): 381.1 ($C_{21}H_{15}{}^{37}ClN_2OS+H^+$), 379.1 ($C_{21}H_{15}{}^{35}ClN_2OS+H^+$).

IR $\tilde{\nu}$ [cm^{-1}]: 631 (m), 644 (m), 692 (m), 716 (m), 737 (m), 804 (w), 837 (m), 878)m), 908 (w), 934 (w), 984 (w), 1020 (m), 1043 (m), 1070 (w), 1099 (s), 1123 (w), 1163 (w), 1196 (m), 1227 (m), 1265 (w), 1288 (m), 1310 (m), 1331 (m), 1360 (m), 1389 (m), 1400 (m), 1443 (s), 1470 (s), 1481 (s), 1551 (m), 1578 (m), 1591 (m), 3032 (w), 3096 (w).

UV/Vis (C_3H_6O): λ_{max} (ε) = 392 (34800).

Anal calcd for $C_{21}H_{15}ClN_2OS$ [378.0]: C 66.57, H 3.99, N 7.39, S 8.46; Found: C 66.67, H 4.10, N 7.11, S 8.75.

(*Z*)-2-(3-(4-Bromobenzyl)benzo[*d*]thiazol-2(3*H*)-ylidene)-1-(4-bromophenyl)ethan-1-one (3v)

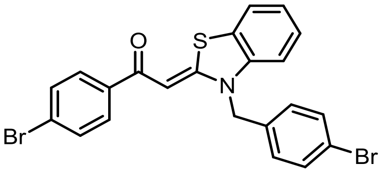

$C_{22}H_{15}Br_2NOS$

[500.92]

The synthesis was performed according to **GPII** to give 273 mg (0.545 mmol, 55%) of the desired product **3v** as a yellow solid.

Mp: 199 °C.

R_f (*n*-hexane/acetone 4:1): 0.30.

^{1}H NMR (500 MHz, acetone-d$_6$/CS$_2$ 5:1): δ 5.59 (s, 2 H), 6.86 (s, 1 H), 7.24-7.28 (m, 3 H), 7.35-7.41 (m, 2 H), 7.48-7.52 (m, 2 H), 7.53-7.58 (m, 2 H), 7.77 (d, ^3J = 7.9 Hz, 1 H), 7.89 (d, ^3J = 8.5 Hz, 2 H).

^{13}C NMR (125 MHz, acetone-d$_6$/CS$_2$ 5:1): δ 49.2 (CH$_2$), 88.0 (CH), 111.5 (CH), 122.2 (CH), 123.3 (CH), 124.1 (CH), 125.8 (CH), 127.6 (CH), 128.0 (C_{quat}), 129.6 (CH), 129.9 (CH), 132.2 (CH), 132.8 (CH), 135.5 (C_{quat}), 137.3 (C_{quat}), 138.2 (C_{quat}), 139.6 (C_{quat}), 140.7 (C_{quat}), 162.9 (C_{quat}), 183.1 (C_{quat}).

MALDI-TOF (*m/z*): 503.9 ($C_{22}H_{15}{}^{81}Br_2NOS+H^+$), 501.9 ($C_{22}H_{15}{}^{81}Br^{79}BrNOS+H^+$), 499.9 ($C_{22}H_{15}{}^{79}Br_2NOS+H^+$).

IR $\tilde{\nu}$ [cm^{-1}]: 658 (m), 673 (m), 689 (m), 706 (m), 739 (s), 752 (s), 789 (m), 800 (m), 816 (m), 837 (m), 880 (s), 924 (w), 937 (w), 962 (w), 995 (w), 1009 (m), 1022 (m), 1043 (m), 1070 (s), 1090 (m), 1105 (w), 1130 (w), 1153 (w), 1175 (m), 1196 (m), 1232 (m), 1263 (w), 1277 (w), 1290 (m), 1304 (w), 1333 (m), 1385 (m), 1396 (m), 1450 (s), 1466 (s), 1495 (m), 1557 (m), 1589 (m), 2382 (w), 2945 (w), 2984 (w), 3065 (w), 2084 (w).

UV/Vis (C_3H_6O): λ_{max} (ε) = 387 (36800).

Anal calcd for $C_{22}H_{15}Br_2NOS$ [500.9]: C 52.72, H 3.02, N 2.79, S 6.40; Found: C 52.92, H 3.15, N 2.61, S 6.52.

(*Z*)-2-(3-Benzyl)benzo[*d*]thiazol-2(3*H*)-ylidene)-1-(4-bromophenyl)ethan-1-one (3w)

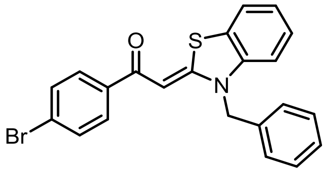

$C_{22}H_{16}BrNOS$

[421.01]

The synthesis was performed according to **GPII** to give 370 mg (0.879 mmol, 88%) of the desired product **3w** as a yellow solid.

Mp: 217 °C.

R_f (*n*-hexane/acetone 4:1): 0.20.

^{1}H NMR (600 MHz, acetone-d_6/CS_2 5:1): δ 5.60 (s, 2 H), 6.86 (s, 1 H), 7.23-7.39 (m, 8 H), 7.57 (d, ^3J = 8.6 Hz, 2 H), 7.77 (d, ^3J = 7.7 Hz, 1 H), 7.89 (d, ^3J = 8.5 Hz, 2 H).

^{13}C NMR (150 MHz, acetone-d_6/CS_2 5:1): δ 49.7 (CH_2), 88.0 (CH), 111.5 (CH), 123.2 (CH), 123.9 (CH), 124.1 (CH), 125.8 (CH), 127.4 (CH), 127.5 (CH), 128.0 (C_{quat}), 128.5 (C_{quat}), 129.7 (CH), 129.8 (CH), 132.2 (CH), 132.8 (CH), 136.0 (C_{quat}), 139.5 (C_{quat}), 140.7 (C_{quat}), 162.9 (C_{quat}), 182.8 (C_{quat}).

MALDI-TOF (*m/z*): 424.1 ($C_{22}H_{16}{}^{81}BrNOS+H^+$), 422.1 ($C_{22}H_{16}{}^{79}Br_2NOS+H^+$).

IR $\tilde{\nu}$ [cm^{-1}]: 650 (m), 675 (m), 689 (s), 714 (m), 748 (s), 795 (w), 827 (w), 837 (m), 870 (s), 899 (m), 939 (w), 999 (m), 1009 (m), 1026 (m), 1047 (m), 1070 (m), 1092 (m), 1136 w), 1177 (m), 1192 (m), 1227 (m), 1272 (m), 1294 (m), 1308 (m), 1327 (m), 1340 (m), 1354 (m), 1385 (m), 1449 (s), 1474 (s), 1491 (s), 1558 (m), 1587 (m), 2594 (w), 2886 (w), 2999 (w), 3059 (w).

UV/Vis (C_3H_6O): λ_{max} (ε) = 388 (38400).

Anal calcd for $C_{22}H_{16}BrNOS$ [421.0]: C 62.56, H 3.82, N 3.32, S 7.59; Found: C 62.86, H 3.90, N 3.21, S 7.80.

(*Z*)-2-(3-(4-Bromobenzyl)benzo[*d*]thiazol-2(3*H*)-ylidene)-1-(4-iodophenyl)ethan-1-one (3x)

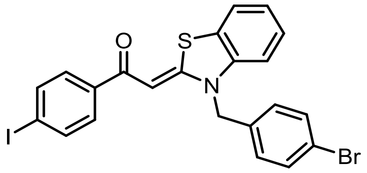

$C_{22}H_{15}BrINOS$

[546.91]

The synthesis was performed according to **GPII** to give 324 mg (0.592 mmol, 59%) of the desired product **3x** as a yellow solid.

Mp: 236 °C.

R_f (*n*-hexane/acetone 4:1): 0.43.

^{1}H NMR (600 MHz, acetone-d$_6$/CS$_2$ 5:1): δ 5.51 (s, 2 H), 6.73 (s, 1 H), 7.20-7.23 (m, 3 H), 7.26-7.27 (m, 2 H), 7.32-7.35 (m, 2 H), 7.47-7.49 (m, 2 H), 7.66-7.68 (m, 2 H), 7.70 (d, ^3J = 7.9 Hz, 1 H), 7.72-7.74 (m, 2 H).

^{13}C NMR (150 MHz, acetone-d$_6$/CS$_2$ 5:1): δ 49.1 (CH$_2$), 87.8 (CH), 98.7 (C$_{quat}$), 111.1 (CH), 122.4 (CH), 123.2 (CH), 123.9 (CH), 127.4 (CH), 128.0 (CH), 129.2 (CH), 129.7 (CH), 132.7 (CH), 134.9 (C$_{quat}$), 138.0 (C$_{quat}$), 139.7 (C$_{quat}$), 140.4 (C$_{quat}$), 162.5 (C$_{quat}$), 182.8 (C$_{quat}$).

MALDI-TOF (*m/z*): 549.9 ($C_{22}H_{15}{}^{81}BrOS+H^+$), 547.9 ($C_{22}H_{15}{}^{79}BrINOS+H^+$).

IR $\tilde{\nu}$ [cm^{-1}]: 658 (m), 675 (m), 692 (m), 704 (m), 719 (s), 739 (s), 748 (s), 793 (m), 808 (m), 835 (m), 878 (s), 920 (m), 941 (m), 953 (m), 1003 (m), 1022 (m), 1047 (m), 1067 (m), 1088 (m), 1107 (m), 1136 (m), 1152 (m), 1180 (m), 1194 (m), 1223 (m), 1261 (m), 1290 (m), 1304 (m), 1329 (m), 1375 (m), 1395 (m), 1433 (m), 1449 (m), 1487 (m), 1580 (m), 2380 (w), 2882 (w), 3090 (w), 2198 (w).

UV/Vis (C_3H_6O): λ_{max} (ε) = 389 (70100).

Anal calcd for $C_{22}H_{15}BrINOS$ [546.9]: C 48.20, H 2.76, N 2.55, S 5.85; Found: C 47.94, H 2.92, N 2.64, S 5.68.

(*Z*)-2-(3-Benzyl)benzo[*d*]thiazol-2(3*H*)-ylidene)-1-(4-iodophenyl)ethan-1-one (3y)

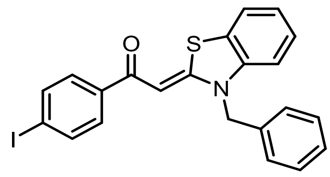

$C_{22}H_{16}INOS$

[469.00]

The synthesis was performed according to **GPII** to give 304 mg (0.879 mmol, 65%) of the desired product **3y** as a yellow solid.

Mp: 238°C.

R_f (*n*-hexane/acetone 4:1): 0.37.

^{1}H NMR (600 MHz, acetone-d_6/CS_2 5:1): δ 5.58 (s, 2 H), 6.84 (s, 1 H), 7.23-7.37 (m, 8 H), 7.71-7.78 (m, 5 H).

^{13}C NMR (150 MHz, acetone-d_6/CS_2 5:1): δ 49.8 (CH_2), 87.9 (CH), 98.3 (C_{quat}), 111.5 (CH), 123.2 (CH), 123.9 (CH), 125.8 (CH), 127.4 (CH), 127.5 (CH), 128.0 (C_{quat}), 128.6 (C_{quat}), 129.7 (CH), 129.8 (CH), 136.0 (CH), 138.2 (C_{quat}), 140.0 (C_{quat}), 140.8 (C_{quat}), 162.9 (C_{quat}), 183.1 (C_{quat}).

MALDI-TOF (*m/z*): 470.0 ($C_{22}H_{16}INOS+H^+$).

IR $\tilde{\nu}$ [cm^{-1}]: 689 (m), 710 (m), 748 (s), 835 (m), 870 (s), 901 (m), 1005 (s), 1026 (m), 1047 (s), 1067 (s), 1082 (m), 1138 (m), 1179 (m), 1190 (m), 1231 (m), 1271 (m), 1292 (m), 1308 (m), 1327 (m), 1341 (m), 1350 (m), 1383 (m), 1408 (m), 1449 (s), 1474 (s), 1489 (s), 1549 (m), 1582 (m), 2886 (m), 2901 (m), 2972 (m), 3059 (m), 3653 (m), 3676 (m).

UV/Vis (C_3H_6O): λ_{max} (ε) = 389 (88600).

Anal calcd for $C_{22}H_{16}BrNOS$ [469.0]: C 56.30, H 3.44, N 2.98, S 6.83; Found: C 56.25, H 3.31, N 2.93, S 6.81.

(Z)-2-(3-(4-Bromobenzyl)benzo[*d*]thiazol-2(3*H*)-ylidene)-1-(4-(trifluoromethyl)-phenyl)ethan-1-one (3z)[6]

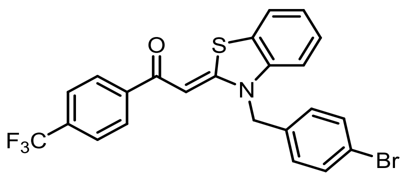

$C_{23}H_{15}BrF_3NOS$

[489.00]

The synthesis was performed according to **GPII** to give 352 mg (0.720 mmol, 72%) of the desired product **3z** as a yellow solid.

Mp: 209 °C.

R_f (*n*-hexane/acetone 4:1): 0.26.

^{1}H NMR (600 MHz, acetone-d_6/CS_2 5:1): δ 5.63 (s, 2 H), 6.94 (s, 1 H), 7.26-7.28 (m, 3 H), 7.39-7.40 (m, 2 H), 7.51-7.53 (m, 2 H), 7.72 (d, ^3J = 8.1 Hz, 2 H), 7.79 (d, ^3J = 8.1 Hz, 1 H), 8.14 (d, ^3J = 8.1 Hz, 2 H).

^{13}C NMR (150 MHz, acetone-d_6/CS_2 5:1): δ 49.2 (CH_2), 88.3 (CH), 111.7 (CH), 122.2 (CH), 123.4 (CH), 124.2 (CH), 125.9 (CH), 126.0 (CF_3), 127.7 (CH), 127.9 (CH), 128.5 (C_{quat}), 129.5 (CH), 132.2 (CH), 132.4 (C_{quat}), 132.8 (CH), 135.4 (C_{quat}), 137.3 (C_{quat}), 138.2 (C_{quat}), 139.6 (C_{quat}), 143.8 (C_{quat}), 162.9 (C_{quat}), 183.1 (C_{quat}).

MALDI-TOF (*m/z*): 492.1 ($C_{23}H_{15}{}^{81}BrF_3NOS+H^+$), 490.1 ($C_{23}H_{15}{}^{79}BrF_3NOS^{+}+H^+$).

IR $\tilde{\nu}$ [cm^{-1}]: 629 (m), 656 (m), 702 (s), 745 (s), 770 (s), 791 (m), 800 (m), 818 (m), 831 (m), 851 (s), 881 (m), 930 (m), 939 (m), 957 (m), 966 (m), 997 (m), 1011 (s), 1024 (m), 1043 (m), 1063 (s), 1090 (m), 1105 (s), 1115 (s), 1157 (m), 1186 (m), 1198 (m), 1227 (m), 1265 (m), 1296 (m), 1308 (m), 1321 (m), 1396 (m), 1433, M), 1452 (m), 1468 (m), 1485 (s), 1514 (m), 1564 (m), 1603 (w), 2372 (w), 2637 (w), 2889 (w), 2955 (w), 3065 (w).

UV/Vis (C_3H_6O): λ_{max} (ε) = 390 (36600).

Anal calcd for $C_{23}H_{15}BrF_3NOS$ [489.0]: C 56.34, H 3.08, N 2.86, S 6.54; Found: C 56.57, H 3.17, N 2.78, S 6.63.

(*Z*)-2-(3-Benzyl)benzo[*d*]thiazol-2(3*H*)-ylidene)-1-(4-(trifluoromethyl)phenyl)ethan-1-one (3aa)[6]

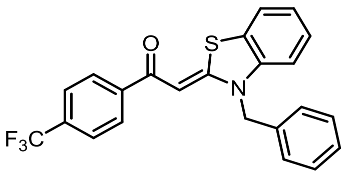

$C_{23}H_{16}F_3NOS$

[411.09]

The synthesis was performed according to **GPII** to give 207 mg (0.504 mmol, 50%) of the desired product **3aa** as a yellow solid.

Mp: 165 °C.

R_f (*n*-hexane/acetone 4:1): 0.25.

^{1}H NMR (500 MHz, acetone-d$_6$/CS$_2$ 5:1): δ 5.60 (s, 2 H), 6.94 (s, 1 H), 7.21-7.44 (m, 8 H), 7.73 (d, ^3J = 8.2 Hz, 2 H), 7.80 (d, ^3J = 7.8 Hz, 1 H), 8.14 (d, ^3J = 7.9 Hz, 2 H).

^{13}C NMR (125 MHz, acetone-d$_6$/CS$_2$ 5:1): δ 49.7 (CH_2), 88.1 (CH), 111.6 (CH), 122.2 (CH), 123.1 (CH), 123.2 (CH), 125.9 (CH), 125.7 (CF_3), 127.7 (CH), 127.9 (CH), 128.3 (C_{quat}), 129.6 (CH), 132.2 (CH), 132.4 (C_{quat}), 132.8 (CH), 135.4 (C_{quat}), 137.3 (C_{quat}), 138.2 (C_{quat}), 139.6 (C_{quat}), 143.8 (C_{quat}), 162.9 (C_{quat}), 183.1 (C_{quat}).

MALDI-TOF (*m/z*): 412.1 ($C_{23}H_{16}F_3NOS+H^+$).

IR $\tilde{\nu}$ [cm^{-1}]: 613 (w), 637 (m), 667 (m), 675 (m), 698 (s), 710 (s), 739 (s), 750 (m), 768 (s), 802 (w), 814 (w), 831 (m), 862 (s), 876 (m), 914 (m), 934 (w), 951 (w), 964 (m), 1003 (m), 1015 (s), 1026 (m), 1064 (s), 1090 (m), 1105 (s), 1165 (s), 1223 (m), 1265 (m), 1308 (s), 1323 (s), 1354 (m), 1391 (m), 1406 (m), 1454 (s), 1468 (s), 1512 (m), 1564 (m), 1582 (w), 1601 (w), 1651 (w), 1661 (m), 2382 (w), 2733 (w), 2930 (w), 3015 (w), 3030 (w), 3061 (w), 3088 (w), 3127(w).

UV/Vis (C_3H_6O): λ_{max} (ε) = 391 (31800).

Anal calcd for $C_{23}H_{16}F_3NOS$ [411.1]: C 67.14, H 3.92, N 3.40, S 7.79; Found: C 67.44, H 3.96, N 3.38, S 8.09.

(*Z*)-4-(2-(3-(4-Bromobenzyl)benzo[*d*]thiazol-2(3*H*)-ylidene)acetyl)benzonitrile (3ab)

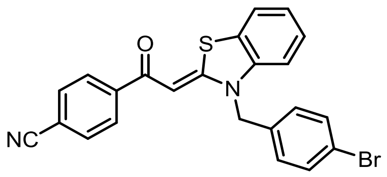

$C_{23}H_{15}BrN_2OS$

[446.01]

The synthesis was performed according to **GPII** to give 251 mg (0.563 mmol, 56%) of the desired product **3ab** as an orange solid.

Mp: 250 °C (decomposition).

R_f (*n*-hexane/acetone 4:1): 0.20.

^{1}H NMR (300 MHz, dmso-d$_6$): δ 5.67 (s, 2 H), 7.04 (s, 1 H), 7.16-7.27 (m, 3 H), 7.36-7.52 (m, 5 H), 7.85-7.91 (m, 2 H), 8.13 (d, ^3J = 8.2 Hz, 2 H).

^{13}C NMR (75 MHz, dmso-d$_6$): δ 47.8 (CH_2), 87.7 (CH), 111.4 (CH), 113.0 (C_{quat}), 118.6 (C_{quat}), 120.7 (CH), 122.8 (CH), 123.4 (C_{quat}), 126.1 (CH), 127.0 (CH), 127.7 (CH), 128.9 (CH), 131.7 (CH), 134.9 (C_{quat}), 139.3 (C_{quat}), 142.6 (C_{quat}), 162.5 (C_{quat}), 181.0 (C_{quat}).

MALDI-TOF (*m/z*): 448.1 ($C_{23}H_{15}{}^{81}BrN_2OS+H^+$), 446.1 ($C_{23}H_{15}{}^{79}BrN_2OS+H^+$).

IR $\tilde{\nu}$ [cm^{-1}]: 644 (m), 662 (m), 681 (w), 716 (m), 750 (s), 766 (s), 791 (w), 818 (m), 841 (m), 856 (m), 881 (s), 934 (w), 1009 (m), 1045 (w), 1069 (m), 1092 (m), 1111 (w), 1134 (w), 1161 (w), 1174 (w), 1198 (m), 1229 (m), 1267 (w), 1290 (w), 1327 (m), 1337 (m), 1395 (m), 1447 (s), 1472 (s), 1501 (m), 1555 (m), 1594 (m), 2226 (m).

UV/Vis (C_3H_6O): λ_{max} (ε) = 397(31500).

Anal calcd for $C_{23}H_{15}BrN_2OS$ [446.0]: C 61.75, H 3.38, N 6.26, S 7.17; Found: C 61.46, H 3.29, N 6.01, S 7.13.

(*Z*)-4-(2-(3-Benzylbenzo[*d*]thiazol-2(3*H*)-ylidene)acetyl)benzonitrile (3ac)

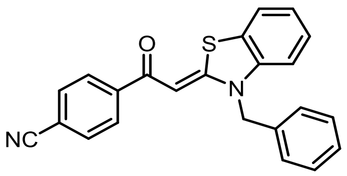

$C_{23}H_{16}N_2OS$
[368.10]

The synthesis was performed according to **GPII** to give 226 mg (0.655 mmol, 66%) of the desired product **3ac** as an orange solid.

Mp: 198 °C (decomposition).

R_f (*n*-hexane/acetone 4:1): 0.08.

^{1}H NMR (300 MHz, acetone-d_6/CS_2 5:1): δ 5.65 (s, 2 H), 6.96 (s, 1 H), 7.23-7.47 (m, 8 H), 7.76-7.82 (m, 3 H), 8.12 (d, ^3J = 8.4 Hz, 2 H).

^{13}C NMR (75 MHz, acetone-d_6/CS_2 5:1): δ 49.9 (CH_2), 88.5 (CH), 112.0 (CH), 114.7 (C_{quat}), 119.1 (C_{quat}), 123.5 (CH), 124.3 (CH), 127.6 (CH), 127.8 (CH), 128.0 (C_{quat}), 128.7 (CH), 129.2 (CH), 129.9 (CH), 133.0 (CH), 136.3 (C_{quat}), 140.9 (C_{quat}), 144.2 (C_{quat}), 163.8 (C_{quat}), 182.2 (C_{quat}).

EI + MS (70 eV, *m/z* (%)): 348 ($[C_{23}H_{16}N_2OS]^+$, 16), 257 (13), 238 ($[C_{15}H_{12}NS]^+$, 21), 224 ($[C_{14}H_{10}NS]^+$, 17), 215 (12), 214 ($[C_{12}H_8NOS]^+$, 18), 136 ($[C_7H_6NS]^+$, 13), 91 ($[C_7H_6]^+$, 100), 65 ($[C_5H_5]^+$, 11).

IR $\tilde{\nu}$ [cm^{-1}]: 613 (w), 638 (m), 665 (w), 677 (m), 698 (s), 723 (m), 743 (s), 781 (w), 812 (w), 831 (w), 847 (m), 874 (m), 914 (w), 932 (w), 972 (w), 1003 (w), 1018 (m), 1042 (w), 1067 (w), 1088 (w), 1113 (w), 1132 (w), 1157 (w), 1177 (m), 1192 (m), 1223 (m), 1261 (w), 1283 (w), 1296 (w), 1317 (w), 1329 (m), 1354 (w), 1406 (m), 1452 (s), 1508 (m), 1555 (w), 1595 (w), 1665 (w), 2222 (w), 3030 (w), 3063 (w).

UV/Vis (C_3H_6O): λ_{max} (ε) = 397 (43600).

Anal calcd for $C_{23}H_{16}N_2OS$ [368.1]: C 74.98, H 4.28, N 7.60, S 8.70; Found: C 74.92, H 4.43, N 7.34, S 8.65.

(*Z*)-2-(3-(4-Bromobenzyl)benzo[*d*]thiazol-2(3*H*)-ylidene)-1-(4-nitrophenyl)ethan-1-one (3ad)

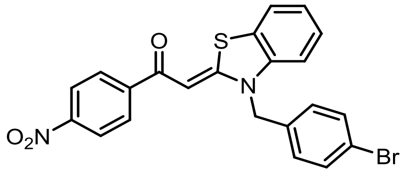

$C_{22}H_{15}BrN_2O_3S$

[466.00]

The synthesis was performed according to **GPII** to give 207 mg (0.444 mmol, 44%) of the desired product **3ad** as an orange solid.

Mp: 232 °C.

R_f (*n*-hexane/acetone 4:1): 0.22.

^{1}H NMR (500 MHz, dmso-d$_6$): δ 5.71 (s, 2 H), 7.10 (s, 1 H), 7.22 (d, ^3J = 8.1 Hz, 2 H), 7.30 (t, ^3J = 7.5 Hz, 1 H), 7.44 (t, ^3J = 7.5 Hz, 1 H), 7.50-7.56 (m, 3 H), 7.92 (d, ^3J = 7.5 Hz, 1 H), 8.22-8.31 (m, 4 H).

^{13}C NMR (125 MHz, dmso-d$_6$): δ 47.8 (CH_2), 87.9 (CH), 111.4 (CH), 122.8 (CH), 123.4 (CH), 123.5 (CH), 126.3 (CH), 127.0 (CH), 128.2 (C_{quat}), 128.8 (CH), 131.7 (C_{quat}), 134.7 (C_{quat}), 139.3 (C_{quat}), 144.4 (C_{quat}), 148.6 (C_{quat}), 162.5 (C_{quat}), 180.5 (C_{quat}).

MALDI-TOF (*m/z*): 469.1 ($C_{22}H_{15}{}^{81}BrN_2O_3S+H^+$), 467.1 ($C_{22}H_{15}{}^{79}BrN_2O_3S+H^+$).

IR $\tilde{\nu}$ [cm^{-1}]: 654 (m), 691 (m), 704 (m), 723 (s), 731 (s), 745 (s), 777 (m), 789 (m), 816 (m), 835 (m), 851 (s), 883 (m), 934 (m), 964 (w), 995 (m), 1011 (m), 1022 (m), 1040 (m), 1069 (s), 1092 (m), 1109 (m), 1132 (m), 1157 (m), 1180 (m), 1198 (m), 1227 (m), 1269 (m), 1294 (m), 1306 (m), 1321 (s), 1337 (s), 1375 (m), 1396 (m), 1433 (m), 1460 (s), 1481 (s), 1497 (s), 1574 (m), 1446 (w), 2851 (w), 2901 (w), 2914 (w), 2959, 3065 (w), 3082 (w), 3103 (w).

UV/Vis (C_3H_6O): λ_{max} (ε) = 413 (12000).

Anal calcd for $C_{22}H_{15}BrN_2O_3S$ [466.0]: C 56.54, H 3.24, N 5.99, S 6.86; Found: C 56.82, H 3.23, N 5.77, S 6.80.

(*Z*)-2-(3-Benzyl)benzo[*d*]thiazol-2(3*H*)-ylidene)-1-(4-nitrophenyl)ethan-1-one (3e)

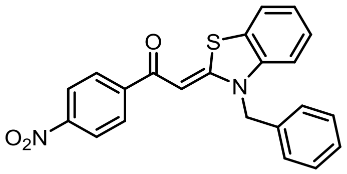

$C_{22}H_{16}N_2O_3S$

[388.09]

The synthesis was performed according to **GPII** to give 285 mg (0.734 mmol, 73%) of the desired product **3e** as an orange solid.

Mp: 266 °C.

R_f (*n*-hexane/acetone 4:1): 0.12.

^{1}H NMR (600 MHz, dmso-d$_6$): δ 5.74 (s, 2 H), 7.12 (s, 1 H), 7.26-7.36 (m, 6 H), 7.43 (t, ^3J = 7.5 Hz, 1 H), 7.53(d, ^3J = 8.1 Hz, 1 H), 7.92 (d, ^3J = 8.1 Hz, 1 H), 8.22-8.30 (m, 4 H).

^{13}C NMR (150 MHz, dmso-d$_6$): δ 48.5 (CH_2), 88.0 (CH), 111.6 (CH), 122.8 (CH), 123.5 (CH), 123.6 (CH), 126.1 (CH), 126.7 (CH), 127.1 (CH), 127.6 (C_{quat}), 128.3 (C_{quat}), 128.9 (C_{quat}), 135.4 (C_{quat}), 139.5 (C_{quat}), 144.4 (C_{quat}), 148.6 (C_{quat}), 162.7 (C_{quat}), 180.5 (C_{quat}).

MALDI-TOF (*m/z*): 389.1 ($C_{22}H_{16}N_2O_3S+H^+$).

IR $\tilde{\nu}$ [cm^{-1}]: 619 (w), 633 (w), 646 (m), 667 (m), 692 (m), 718 (s),777 (m), 800 (w), 818 (w), 847 (s), 866 (m), 880 (m), 914 (w), 970 (w), 984 (w), 1009 (m), 1018 (m), 1038 (m), 1057 (m), 1067 (m), 1088 (m), 1109 (m), 1132 (m), 1155 (m), 1173 (m), 1190 (m), 1223 (m), 1261 (m), 1279 (m), 1323 (m), 1339 (s), 1358 (w), 1400 (m), 1433 (m), 1449 (s), 1464 (s), 1477 (s), 1493 (s), 1512 (s), 1574 (m), 2376 (w), 2442 (w), 2886 (w), 2901 (w), 2924 (w), 2972 (w), 290 (w), 3053 (w).

UV/Vis (C_3H_6O): λ_{max} (ε) = 413 (22200).

Anal calcd for $C_{22}H_{16}N_2O_3S$ [388.1]: C 68.02, H 4.15, N 7.21, S 8.25; Found: C 56.82, H 3.23, N 5.77, S 6.80.

(Z)-2-(3-(4-Bromobenzyl)benzo[d]thiazol-2(3H)-ylidene)-1-(thiophen-2-yl)ethan-1-one (3af)

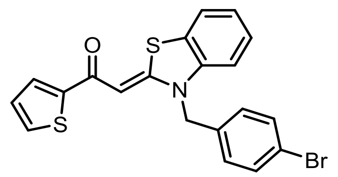

$C_{20}H_{14}BrNOS_2$
[426.97]

The synthesis was performed according to **GPII** to give 306 mg (0.717 mmol, 72%) of the desired product **3af** as a yellow solid.

Mp: 210 °C.

R_f (*n*-hexane/acetone 4:1): 0.11.

^{1}H NMR (300 MHz, acetone-d_6/CS_2 5:1): δ 5.50 (s, 2 H), 6.66 (s, 1 H), 7.04-7.06 (m, 1 H), 7.20-7.35 (m, 5 H), 7.58 (dd, ^3J = 5.0 Hz, ^4J = 1.0 Hz, 1 H), 7.66 (dd, ^3J = 3.6 Hz, ^4J = 1.0 Hz, 1 H), 7.73 (d, ^3J = 7.7 Hz, 1 H).

^{13}C NMR (75 MHz, acetone-d_6/CS_2 5:1): δ 49.0 (CH_2), 88.1 (CH), 111.2 (CH), 122.3 (CH), 123.1 (CH), 123.8 (CH), 127.4 (CH), 128.0 (C_{quat}), 128.3 (CH), 128.4 (C_{quat}), 129.4 (CH), 131.0 (CH), 132.7 (CH), 136.1 (C_{quat}), 140.6 (C_{quat}), 148.1 (C_{quat}), 161.5 (C_{quat}), 177.8 (C_{quat}).

MALDI-TOF (*m/z*): 429.9 ($C_{20}H_{14}{}^{81}BrNOS_2+H^+$), 427.9 ($C_{20}H_{14}{}^{79}BrNOS_2+H^+$).

IR $\tilde{\nu}$ [cm^{-1}]: 640 (m), 658 (m), 702 (s), 743 (s), 797 (s), 820 (m), 833 (s), 847 (s), 870 (m), 895 (w), 953 (w), 984 (w), 1007 (m), 1022 (m), 1036 (m), 1061 (m), 1088 (m), 1113 (m), 1138 (m), 1163 (m), 1194 (m), 1234 (s), 1261 (m), 1292 (m), 1321 (m), 1331 (m), 1346 (m), 1405 (m), 1435 (s), 1464 (s), 1574 (m), 1585 (m), 2714 (w), 3024 (w), 3061 (w).

UV/Vis (C_3H_6O): λ_{max} (ε) = 391 (25500).

Anal calcd for $C_{20}H_{14}BrNOS_2$ [427.0]: C 56.08, H 3.29, N 3.27, S 14.97; Found: C 55.98, H 3.13, N 3.19, S 14.76.

(*Z*)-2-(3-Benzyl)benzo[*d*]thiazol-2(3*H*)-ylidene)-1-(thiophen-2-yl)ethan-1-one (3ag)

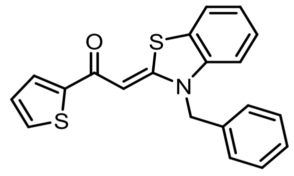

$C_{20}H_{15}NOS_2$

[349.06]

The synthesis was performed according to **GPII** to give 305 mg (0.874 mmol, 87%) of the desired product **3ag** as a yellow solid.

Mp: 190 °C.

R_f (*n*-hexane/acetone 4:1): 0.16.

^{1}H NMR (600 MHz, acetone-d$_6$/CS$_2$ 5:1): δ 5.54 (s, 2 H), 6.74 (s, 1 H), 7.06-7.07 (m, 1 H), 7.18-7.25 (m, 1 H), 7.27-7.40 (m, 7 H), 7.51 (d, ^3J = 7.7 Hz, 2 H), 7.55 (dd, ^3J = 5.0 Hz, ^4J = 1.0 Hz, 1 H), 7.61 (dd, ^3J = 3.7 Hz, ^4J = 1.0 Hz, 1 H), 7.70 (d, ^3J = 7.7 Hz, 1 H).

^{13}C NMR (150 MHz, acetone-d$_6$/CS$_2$ 5:1): δ 49.6 (CH$_2$), 88.1 (CH), 111.5 (CH), 123.1 (CH), 123.8 (CH), 127.5 (CH), 127.9 (C$_{quat}$), 128.3 (CH), 128.5 (CH), 129.7 (CH), 131.1 (CH), 136.1 (C$_{quat}$), 139.6 (C$_{quat}$), 148.3 (C$_{quat}$), 161.8 (C$_{quat}$), 178.0 (C$_{quat}$).

MALDI-TOF (*m/z*): 350.0 ($C_{20}H_{15}NOS_2+H^+$).

IR $\tilde{\nu}$ [cm^{-1}]: 635 (m), 658 (m), 689 (m), 704 (s), 716 (s), 725 (s), 748 (s), 793 (m), 802 (m), 816 (m), 831 (m), 864 (m), 918 (w), 961 (w), 968 (w), 1005 (m), 1022 (m), 1040 (m), 1063 (m), 1088 (m), 1115 (w), 1140 (w), 1161 (m), 1188 (m), 1219 (m), 1231 (m), 1263 (w), 1298 (m), 1312 (m), 1331 (m), 1348 (m), 1400 (m), 1456 (s), 1470 (s), 1572 (m), 2793 (w), 2901 (w), 3009 (w), 3038 (w), 3069 (w).

UV/Vis (C_3H_6O): λ_{max} (ε) = 392 (39200).

Anal calcd for $C_{20}H_{15}NOS_2$ [427.0]: C 68.74, H 4.33, N 4.01, S 18.35; Found: C 68.45, H 4.26, N 3.91, S 18.12.

(*Z*)-2-(3-(4-Bromobenzyl)benzo[*d*]thiazol-2(3*H*)-ylidene)-1-(furan-2-yl)ethan-1-one (3ah)

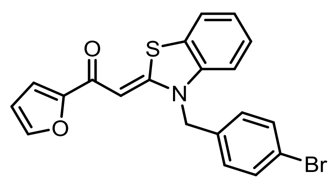

$C_{20}H_{14}BrNO_2S$

[410.99]

The synthesis was performed according to **GPII** to give 315 mg (0.766 mmol, 77%) of the desired product **3ah** as a yellow solid.

Mp: 201 °C.

R_f (*n*-hexane/acetone 4:1): 0.12.

^{1}H NMR (600 MHz, acetone-d$_6$/CS$_2$ 5:1): δ 5.53 (s, 2 H), 6.53-6.54 (m, 1 H), 6.63 (s, 1 H), 7.04 (d, ^3J = 3.4 Hz, 1 H), 7.24-7.26 (m, 3 H), 7.35-7.38 (m, 2 H), 7.54 (d, ^3J = 8.3 Hz, 2 H), 7.60 (s, 1 H), 7.76 (d, ^3J = 7.6 Hz, 1 H).

^{13}C NMR (150 MHz, acetone-d$_6$/CS$_2$ 5:1): δ 49.1 (CH$_2$), 88.1 (CH), 111.4 (CH), 112.7 (CH), 113.3 (CH), 122.1 (CH), 123.2 (CH), 124.0 (CH), 127.6 (CH), 127.8 (C$_{quat}$), 129.5 (CH), 132.8 (CH), 135.4 (C$_{quat}$), 140.6 (C$_{quat}$), 145.0 (CH), 155.5 (C$_{quat}$), 161.7 (C$_{quat}$), 174.7 (C$_{quat}$).

MALDI-TOF (*m/z*): 414.0 ($C_{20}H_{14}{}^{81}BrNO_2S+H^+$), 412.0 ($C_{20}H_{14}{}^{79}BrNO_2S+H^+$).

IR $\tilde{\nu}$ [cm^{-1}]: 656 (m), 698 (m), 710 (m), 735 (s), 756 (s), 785 (m), 799 (m), 816 (m), 829 (m), 856 (s), 918 (w), 959 (w), 991 (w), 1007 (s), 1026, 1047 (m), 1070 (m), 1103 (m), 1140 (w), 1155 (m), 1200 (m), 1219 (m), 1236 (m), 1263 (w), 1292 (m), 1333 (m), 1377 (m), 1391 (m), 1404 (m), 1452 (m), 1470 (s), 1497 (s), 1572 (m), 1597 (m), 2860 (w), 2887 (w), 2961 (w), 2990 (w).

UV/Vis (C_3H_6O): λ_{max} (ε) = 398 (58800).

Anal calcd for $C_{20}H_{14}BrNO_2S$ [411.0]: C 58.26, H 3.42, N 3.40, S 7.78; Found: C 58.42, H 3.40, N 3.27, S 7.78.

(*Z*)-2-(3-Benzyl)benzo[*d*]thiazol-2(3*H*)-ylidene)-1-(furan-2-yl)ethan-1-one (3ai)

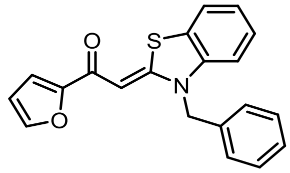

$C_{20}H_{15}NO_2S$

[333.08]

The synthesis was performed according to **GPII** to give 317 mg (0.952 mmol, 95%) of the desired product **3ai** as a yellow solid.

Mp: 177 °C.

R_f (*n*-hexane/acetone 4:1): 0.22.

^{1}H NMR (600 MHz, acetone-d$_6$/CS$_2$ 5:1): δ 5.54 (s, 2 H), 6.53-6.54 (m, 1 H), 6.66 (s, 1 H), 7.03 (d, ^3J = 3.5 Hz, 1 H), 7.23-7.25 (m, 1 H), 7.28-7.30 (m, 3 H), 7.35-7.38 (m, 4 H), 7.60 (s, 1 H), 7.77 (d, ^3J = 7.7 Hz, 1 H).

^{13}C NMR (150 MHz, acetone-d$_6$/CS$_2$ 5:1): δ 49.7 (CH_2), 87.9 (CH), 111.6 (CH), 112.7 (CH), 113.2 (CH), 122.1 (CH), 123.2 (CH), 123.9 (CH), 127.4 (CH), 127.6 (CH), 127.8 (C_{quat}), 127.9 (C_{quat}), 128.6 (CH), 129.8 (CH), 136.0 (C_{quat}), 140.9 (C_{quat}), 145.0 (CH), 155.6 (C_{quat}), 161.9 (C_{quat}), 174.7 (C_{quat}).

MALDI-TOF (*m/z*): 334.2 ($C_{20}H_{15}NO_2S+H^+$).

IR $\tilde{\nu}$ [cm^{-1}]: 615 (w), 638 (m), 667 (w), 696 (m), 710 (m), 746 (s), 795 (w), 833 (w), 847 (s), 899 (m), 934 (w), 980 (w), 1007 (m), 1040 (w), 1065 (m), 1103 (m), 1140 (w), 1157 (m), 1207 (m), 1240 (m), 1263 (w), 1296 (m), 1327 (m), 1369 (m), 1398 (w), 1418 (m), 1429 (m), 1450 (s), 1489 (s), 1558 (m), 1574 (s), 1595 (m), 2359 (w), 2961 (w), 3028 (w), 3059 (w).

UV/Vis (C_3H_6O): λ_{max} (ε) = 399 (43900).

Anal calcd for $C_{20}H_{15}NO_2S$ [333.1]: C 72.05, H 4.53, N 4.20, S 9.62; Found: C 71.95, H 4.60, N 4.09, S 9.65.

5 NMR spectra

^{1}H NMR-spectrum of 3-(4-Bromobenzyl)-2-methylbenzo[*d*]thiazol-3-iumbromide (2b) (dmso-d_6, 300 MHz, 293 K)

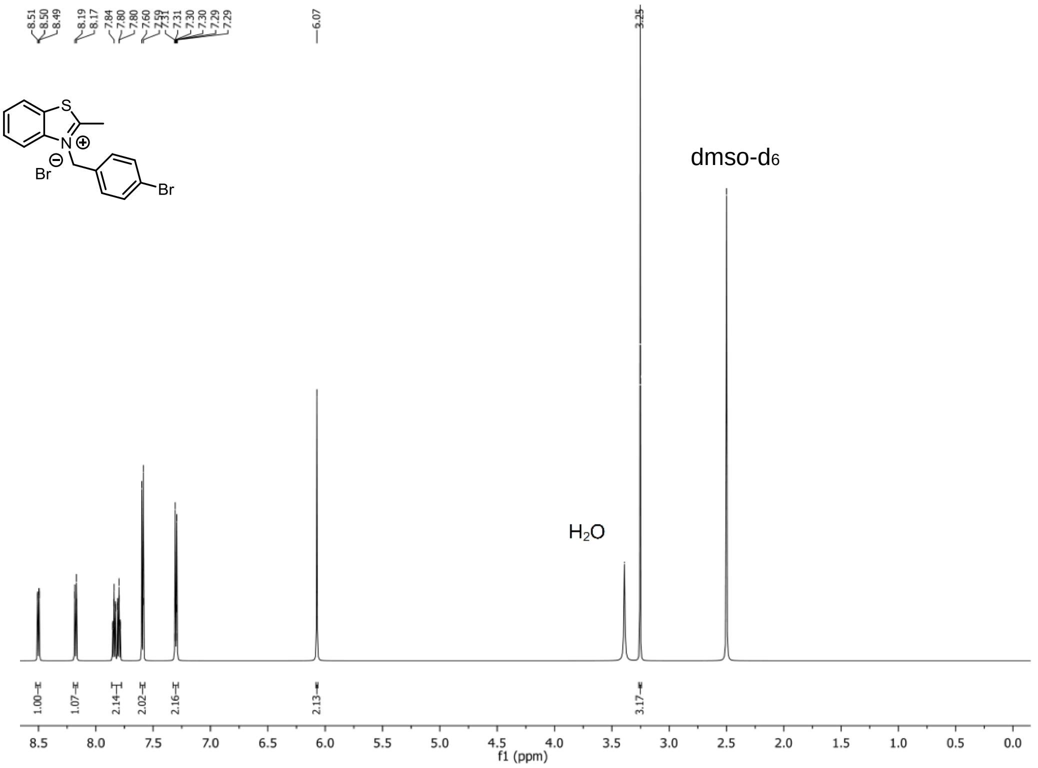

^{13}C NMR-spectrum of 3-(4-Bromobenzyl)-2-methylbenzo[*d*]thiazol-3-iumbromide (2b) (dmso-d_6, 75 MHz, 293 K)

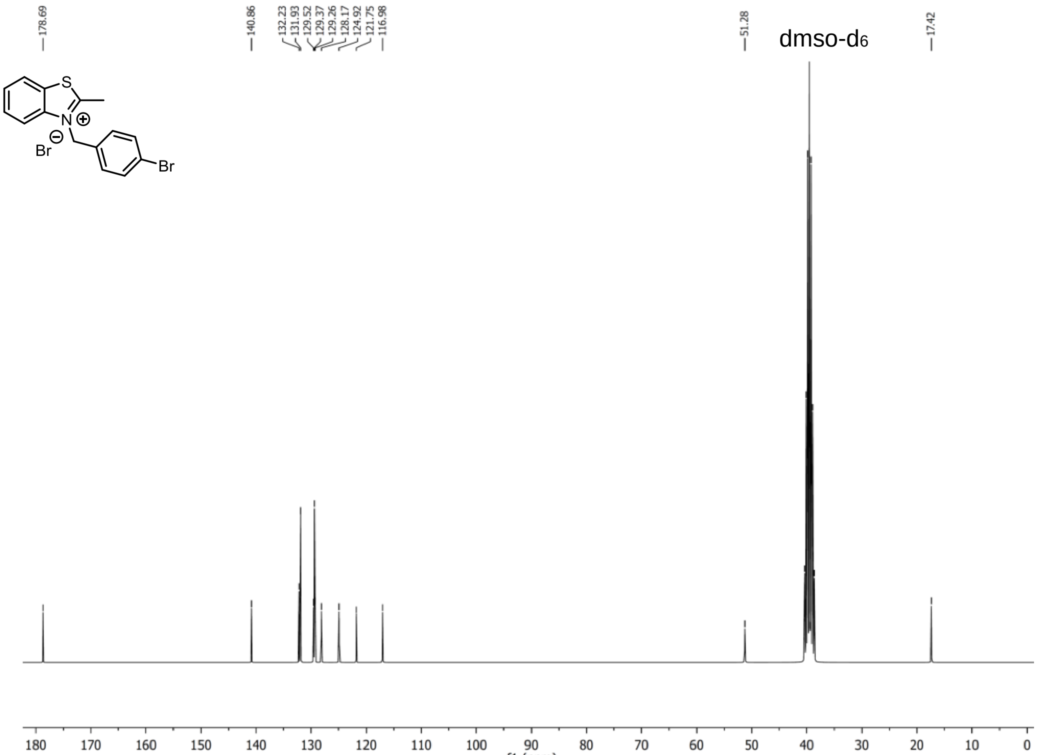

^{1}H NMR-spectrum of (*Z*)-2-(3-(4-Bromobenzyl)benzo[*d*]thiazol-2(3*H*)-ylidene)-1-(4-dimethylaminophenyl)ethan-1-one (3a) (acetone-d_6/CS_2 5:1, 300 MHz, 293 K)

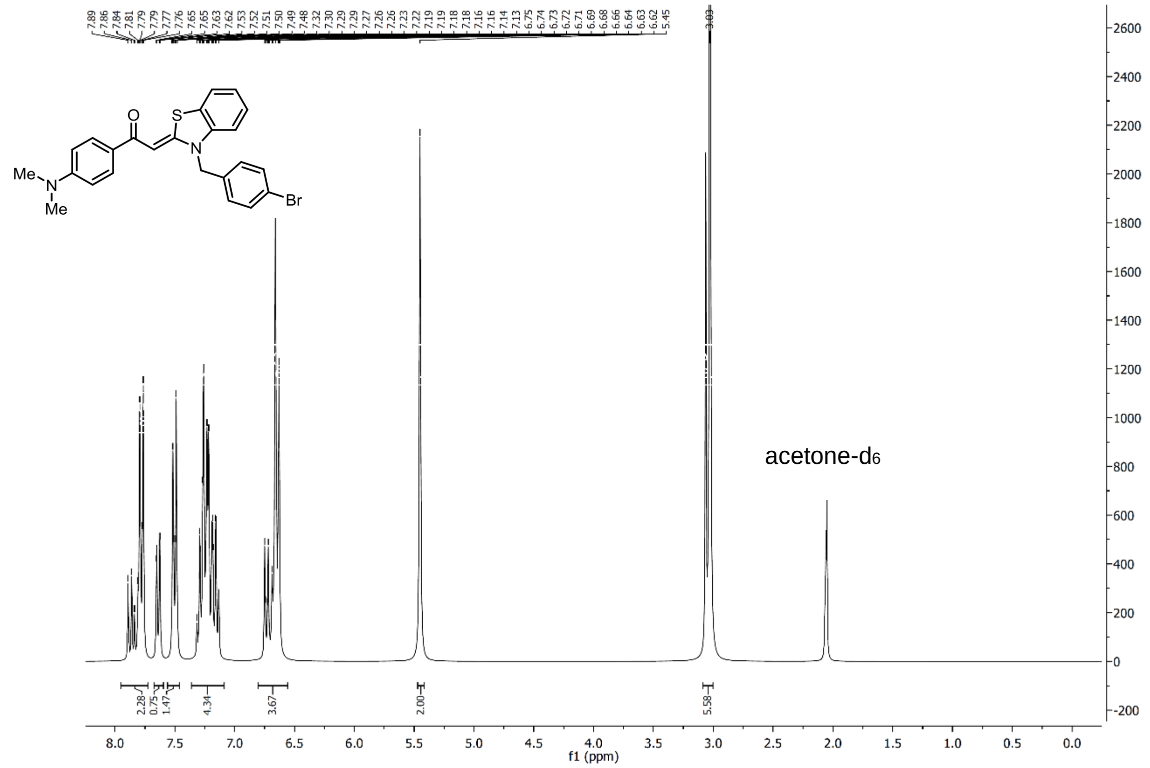

^{13}C NMR-spectrum of (*Z*)-2-(3-(4-Bromobenzyl)benzo[*d*]thiazol-2(3*H*)-ylidene)-1-(4-dimethylaminophenyl)ethan-1-one (3a) (acetone-d_6/CS_2 5:1, 75 MHz, 293 K)

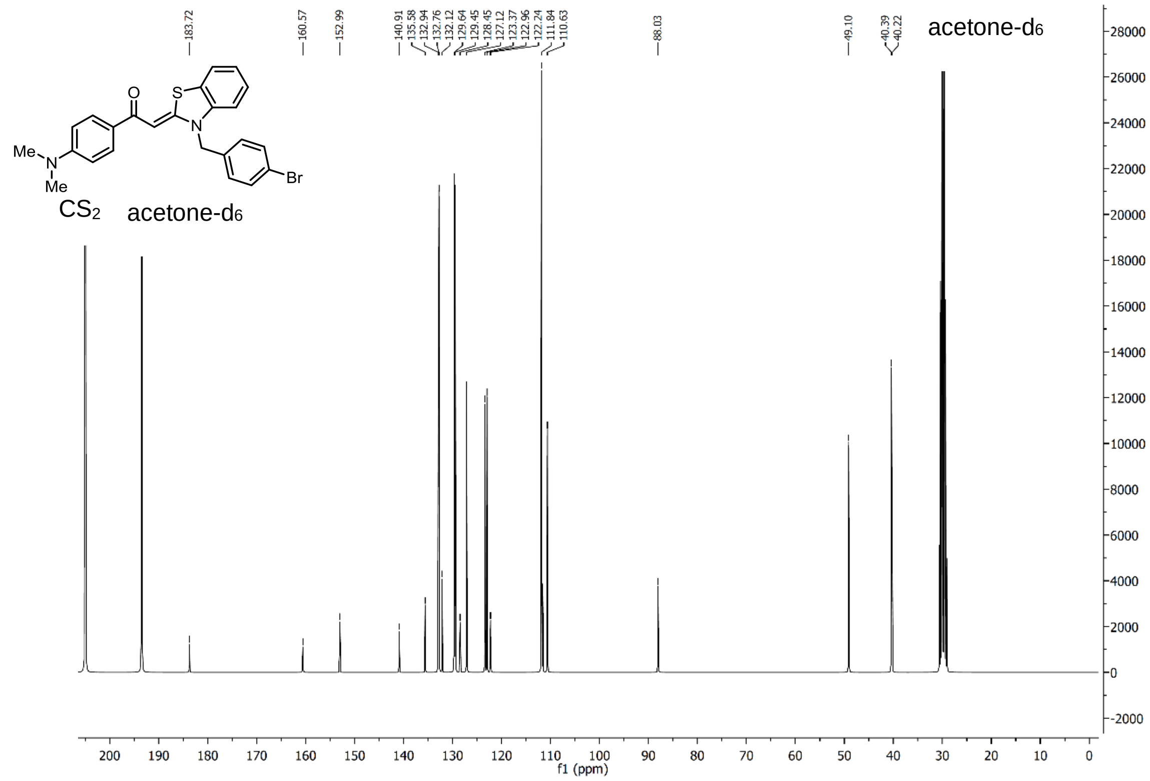

^{1}H NMR-spectrum of (*Z*)-2-(3-Benzyl)benzo[*d*]thiazol-2(3*H*)-ylidene)-1-(4-dimethylaminophenyl)ethan-1-one (3b) (acetone-d_6/CS_2 5:1, 300 MHz, 293 K)

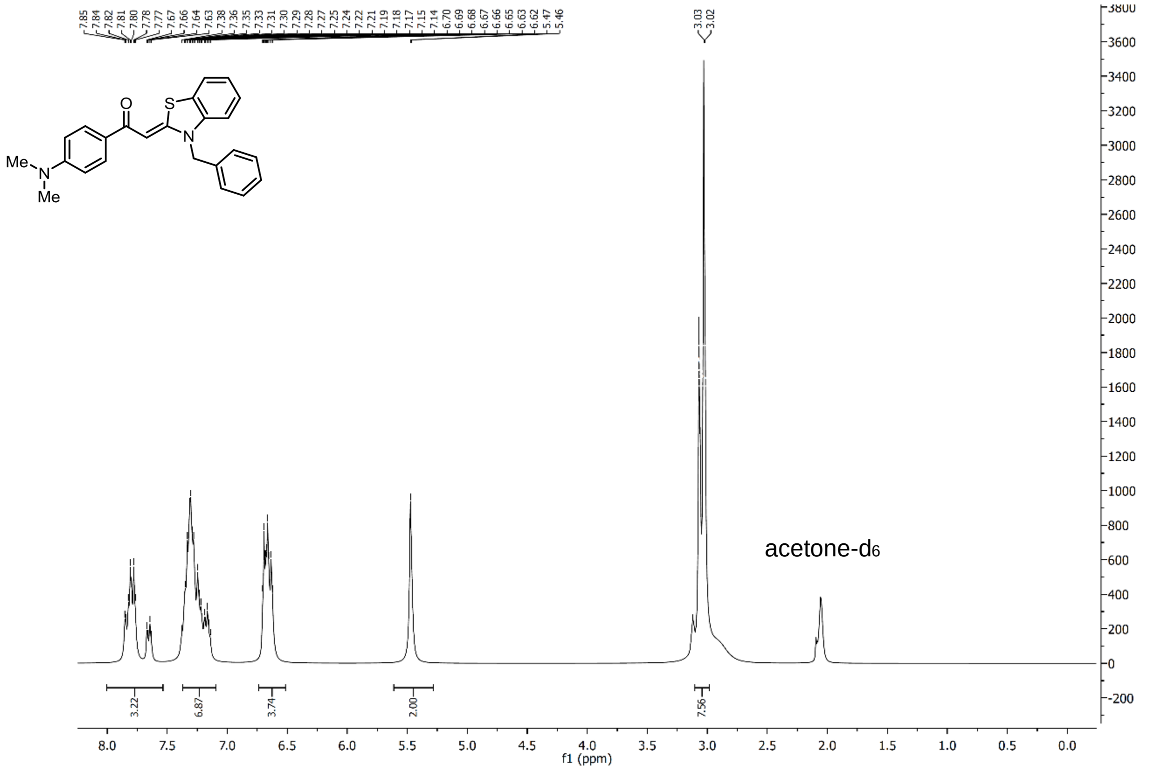

^{13}C NMR-spectrum von (*Z*)-2-(3-Benzyl)benzo[*d*]thiazol-2(3*H*)-ylidene)-1-(4-dimethylaminophenyl)ethan-1-one (3b) (acetone-d_6/CS_2 5:1, 75 MHz, 293 K)

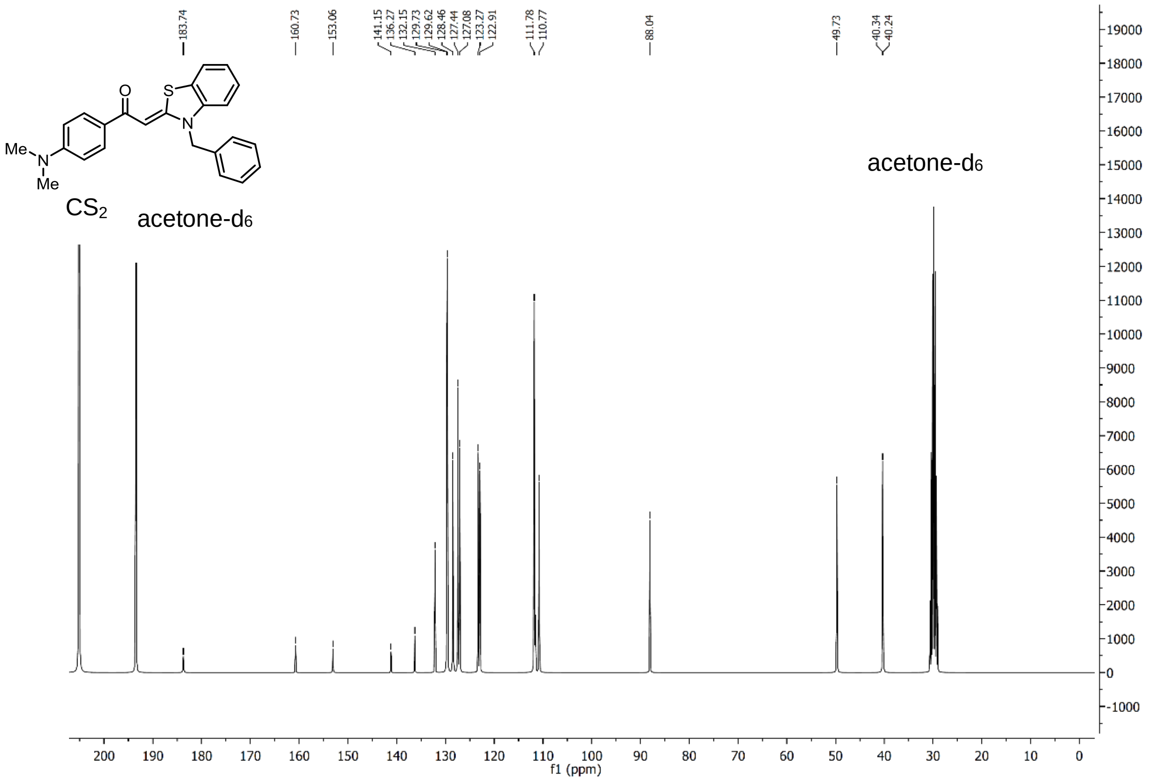

^{1}H NMR-spectrum of (*Z*)-2-(3-(4-Bromobenzyl)benzo[*d*]thiazol-2(3*H*)-ylidene)-1-(4-methoxyphenyl)ethan-1-one (3c) (acetone-d_6/CS_2 5:1, 600 MHz, 293 K)

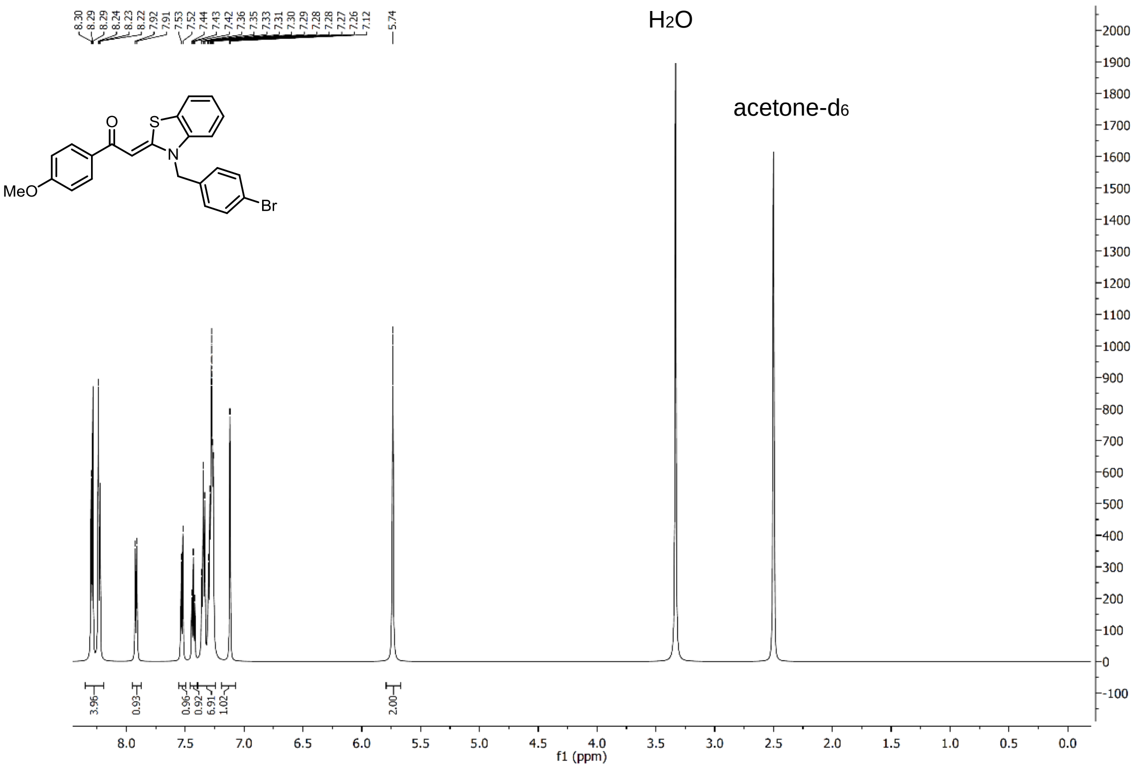

^{13}C NMR-spectrum of (*Z*)-2-(3-(4-Bromobenzyl)benzo[*d*]thiazol-2(3*H*)-ylidene)-1-(4-methoxyphenyl)ethan-1-one (3c) (acetone-d_6/CS_2 5:1, 150 MHz, 293 K)

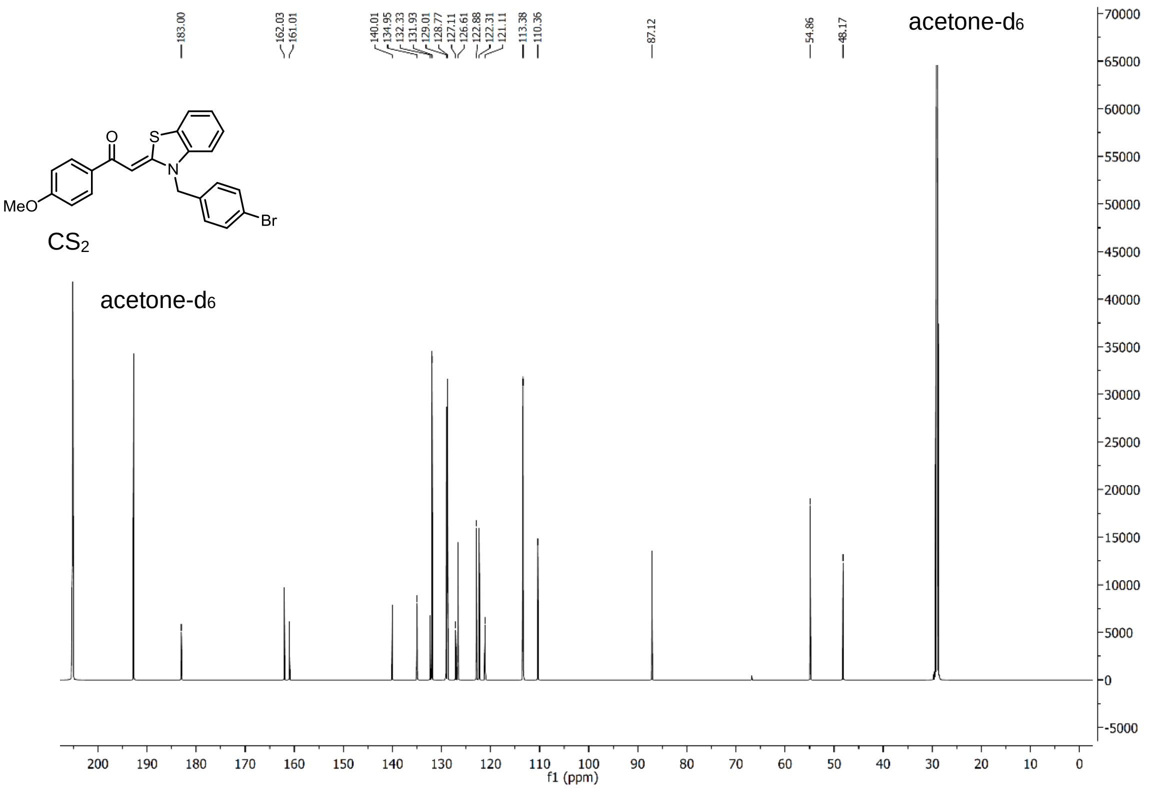

^{1}H NMR-spectrum of (*Z*)-2-(3-Benzyl)benzo[*d*]thiazol-2(3*H*)-ylidene)-1-(4-methoxyphenyl)ethan-1-one (3d) (acetone-d_6/CS_2 5:1, 600 MHz, 293 K)

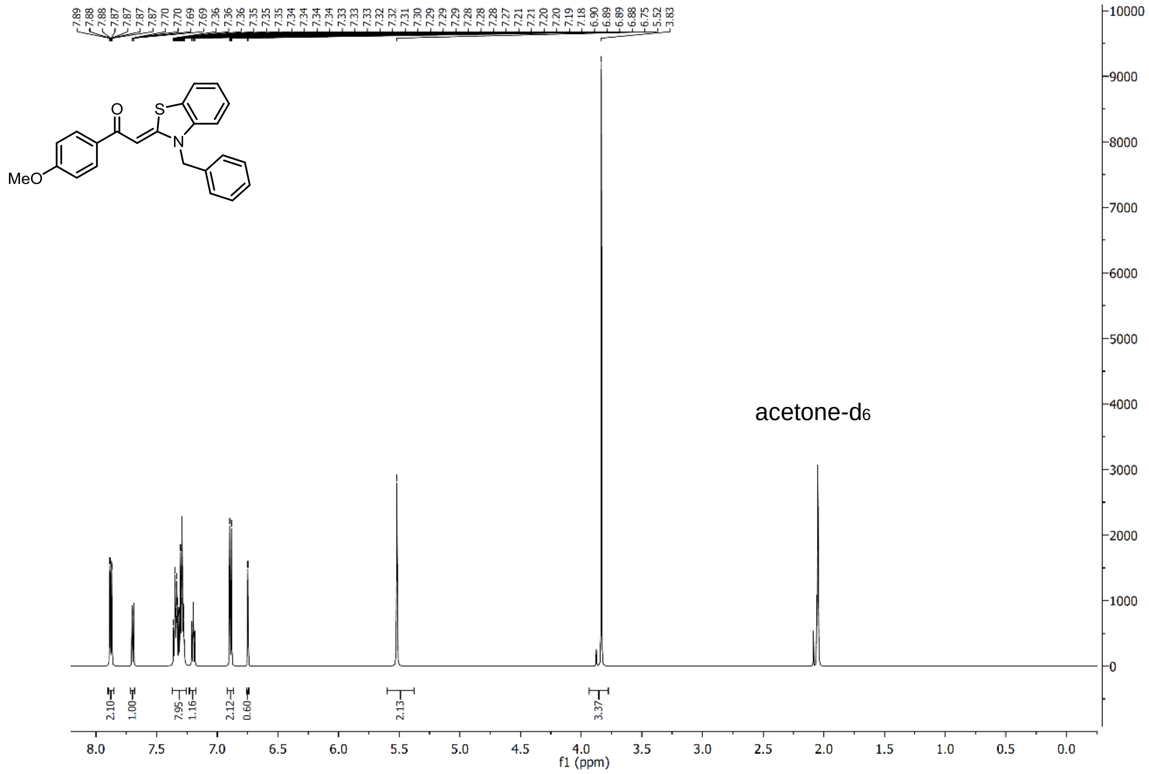

^{13}C NMR-spectrum of (*Z*)-2-(3-Benzyl)benzo[*d*]thiazol-2(3*H*)-ylidene)-1-(4-methoxyphenyl)ethan-1-one (3d) (acetone-d_6/CS_2 5:1, 150 MHz, 293 K)

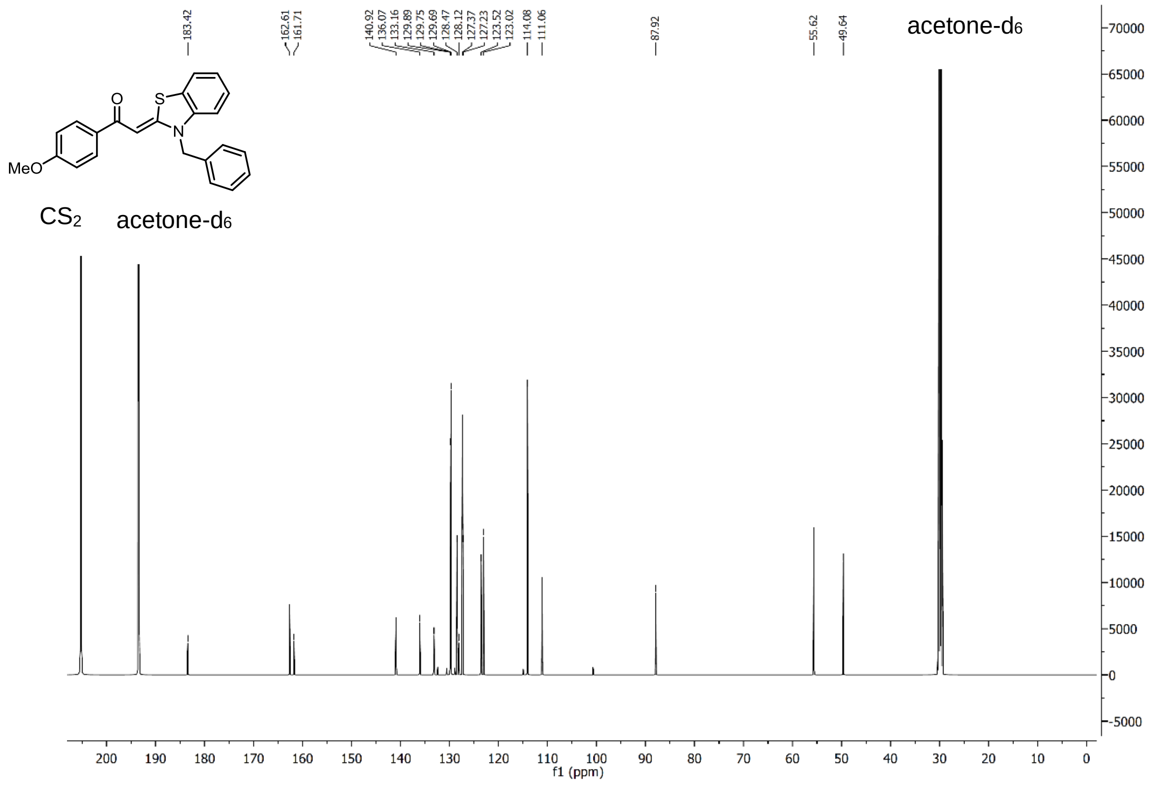

^{1}H NMR-spectrum of (*Z*)-2-(3-(4-Bromobenzyl)benzo[*d*]thiazol-2(3*H*)-ylidene)-1-(4-*tert*-butylphenyl)ethan-1-one (3e) (acetone-d_6/CS_2 5:1, 600 MHz, 293 K)

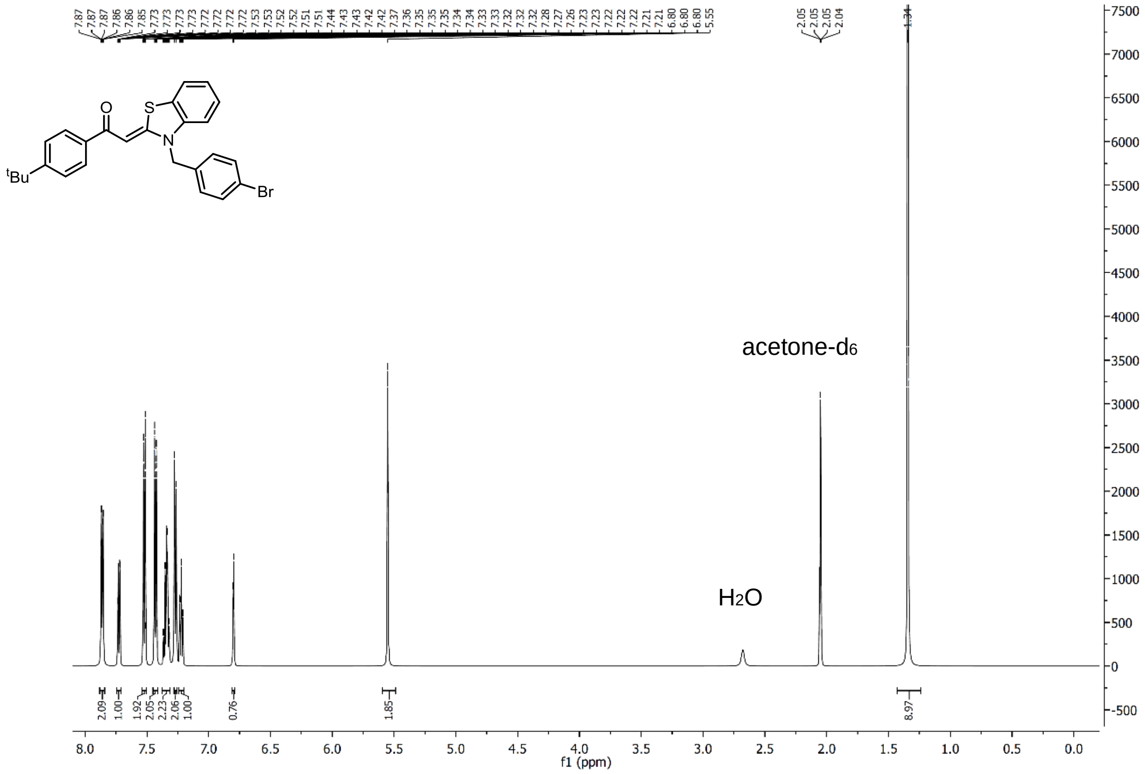

^{13}C NMR-spectrum of (*Z*)-2-(3-(4-Bromobenzyl)benzo[*d*]thiazol-2(3*H*)-ylidene)-1-(4-*tert*-butylphenyl)ethan-1-one (3e) (acetone-d_6/CS_2 5:1, 150 MHz, 293 K)

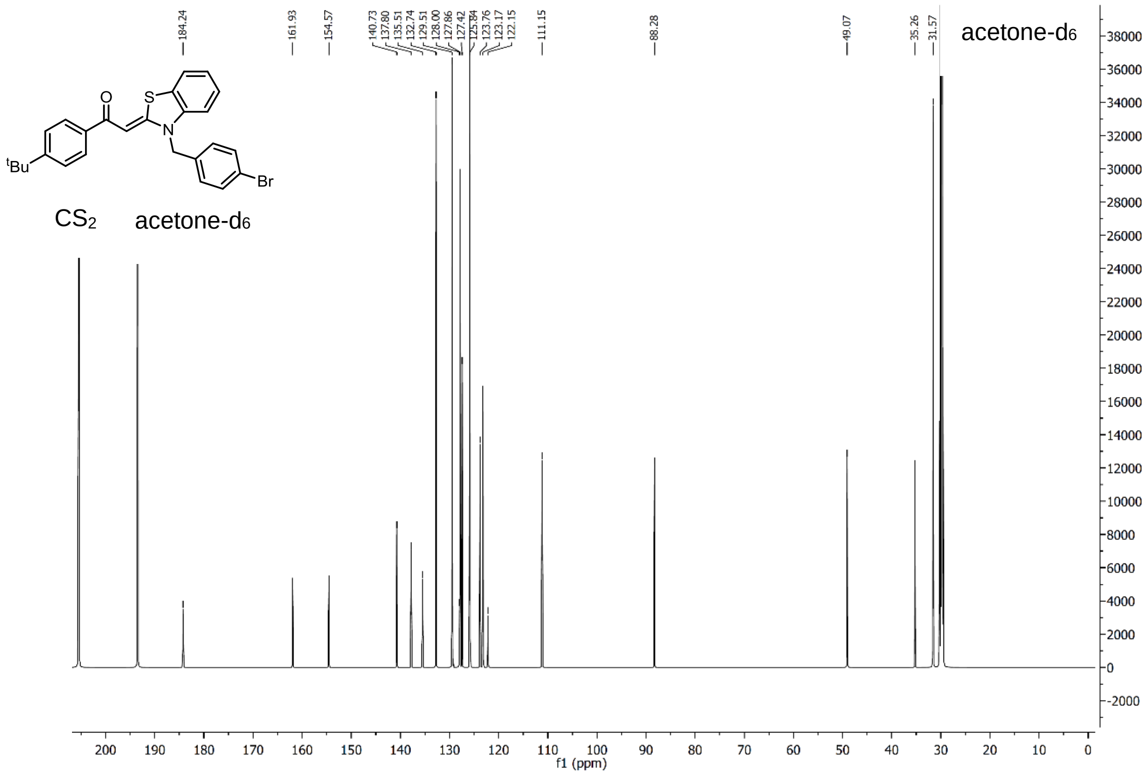

^{1}H NMR-spectrum of (*Z*)-2-(3-Benzyl)benzo[*d*]thiazol-2(3*H*)-ylidene)-1-(4-*tert*-butylphenyl)ethan-1-one (3f) (acetone-d_6/CS_2 5:1, 600 MHz, 293 K)

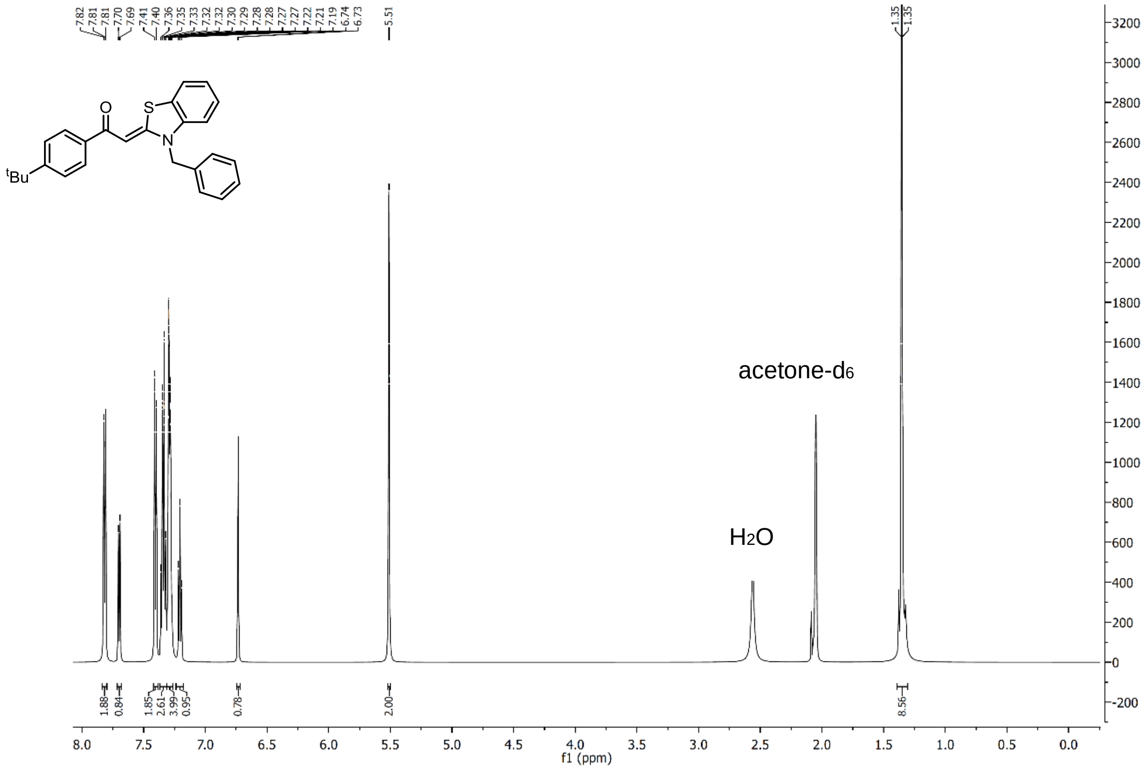

^{13}C NMR-spectrum of (*Z*)-2-(3-Benzyl)benzo[*d*]thiazol-2(3*H*)-ylidene)-1-(4-*tert*-butylphenyl)ethan-1-one (3f) (acetone-d_6/CS_2 5:1, 150 MHz, 293 K)

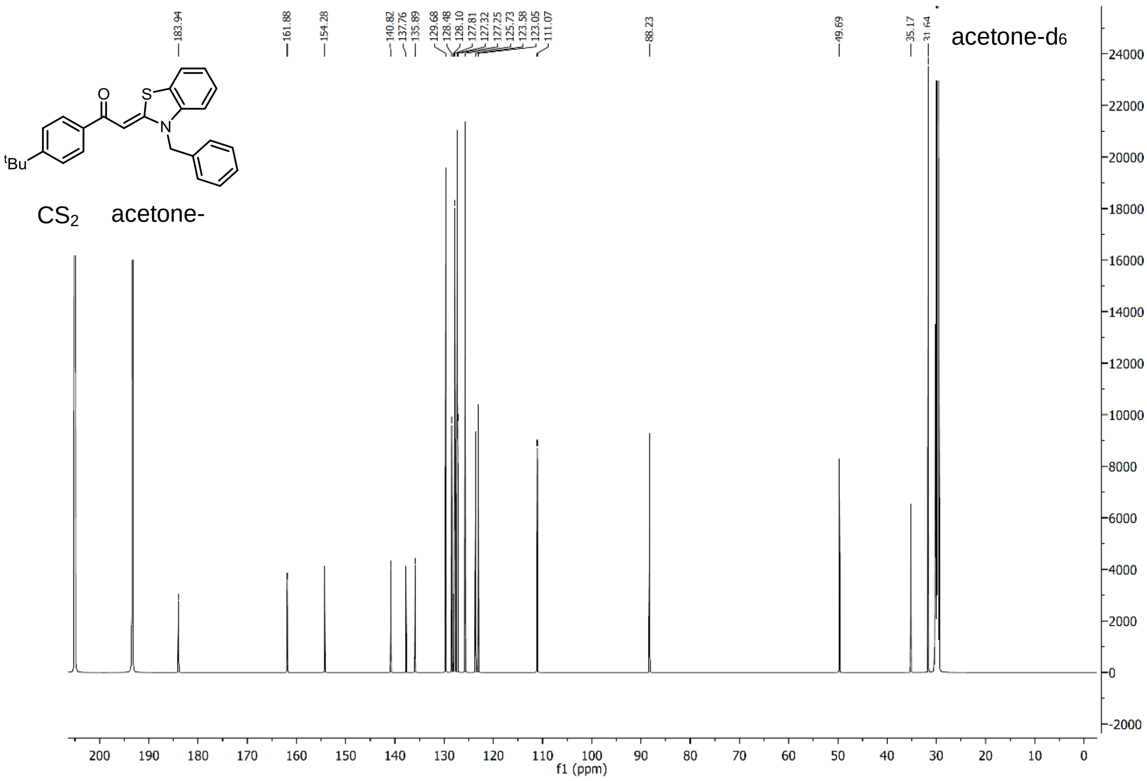

^{1}H NMR-spectrum of (*Z*)-2-(3-(4-Bromobenzyl)benzo[*d*]thiazol-2(3*H*)-ylidene)-1-(*p*-tolyl)ethan-1-one (3g) (acetone-d_6/CS_2 5:1, 600 MHz, 293 K)

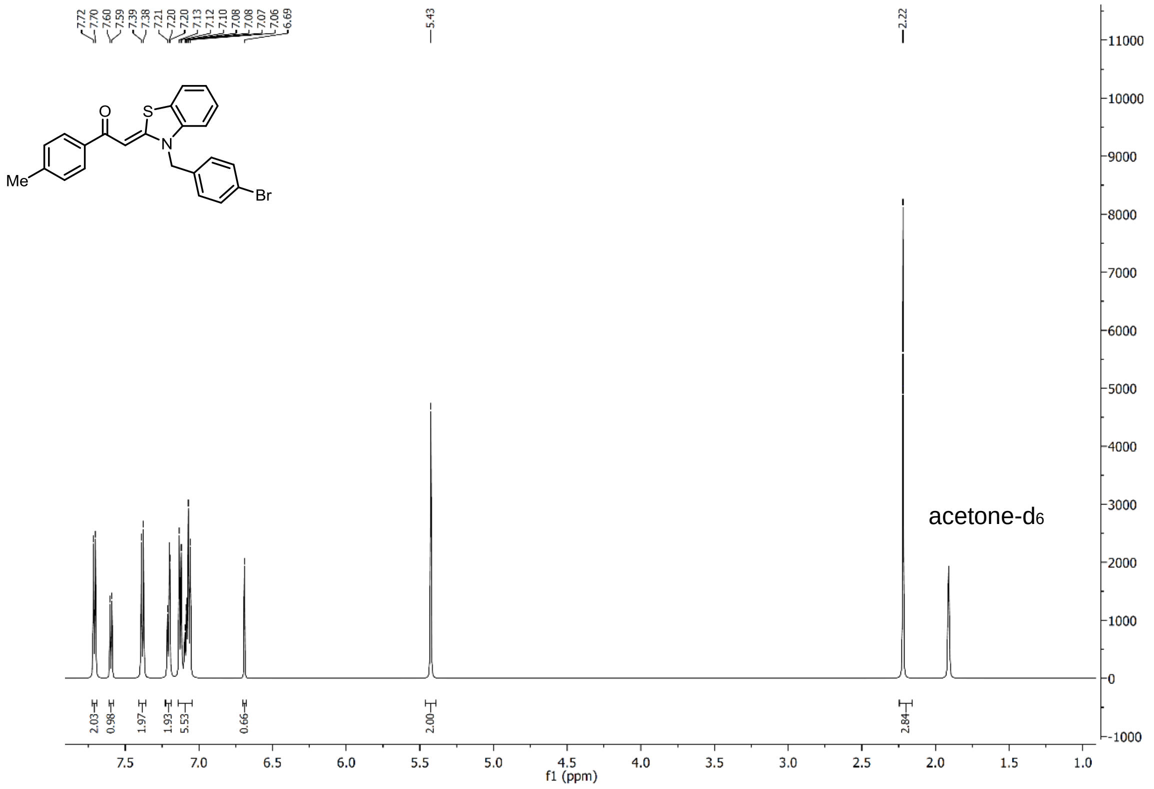

^{13}C NMR-spectrum of (*Z*)-2-(3-(4-Bromobenzyl)benzo[*d*]thiazol-2(3*H*)-ylidene)-1-(*p*-tolyl)ethan-1-one (3g) (acetone-d_6/CS_2 5:1, 150 MHz, 293 K)

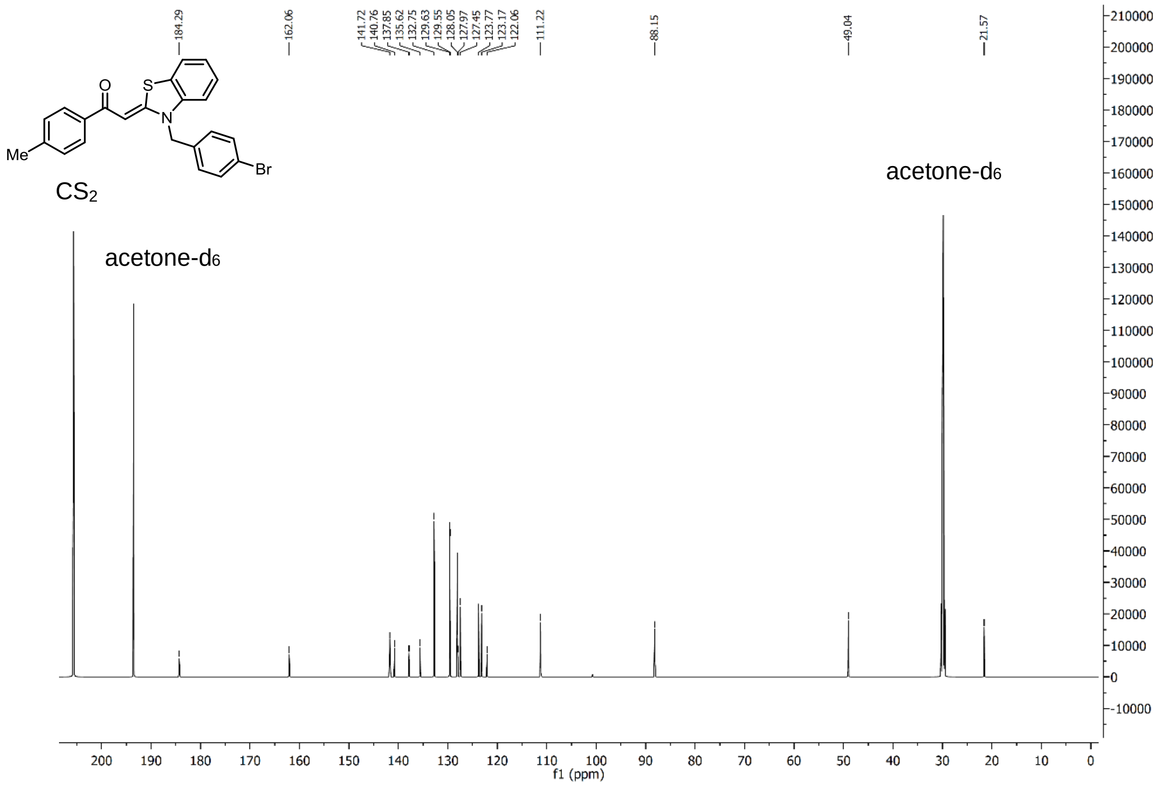

^{1}H NMR-spectrum of (*Z*)-2-(3-Benzyl)benzo[*d*]thiazol-2(3*H*)-ylidene)-1-(*p*-tolyl)ethan-1-one (3h) (acetone-d_6/CS_2 5:1, 600 MHz, 293 K)

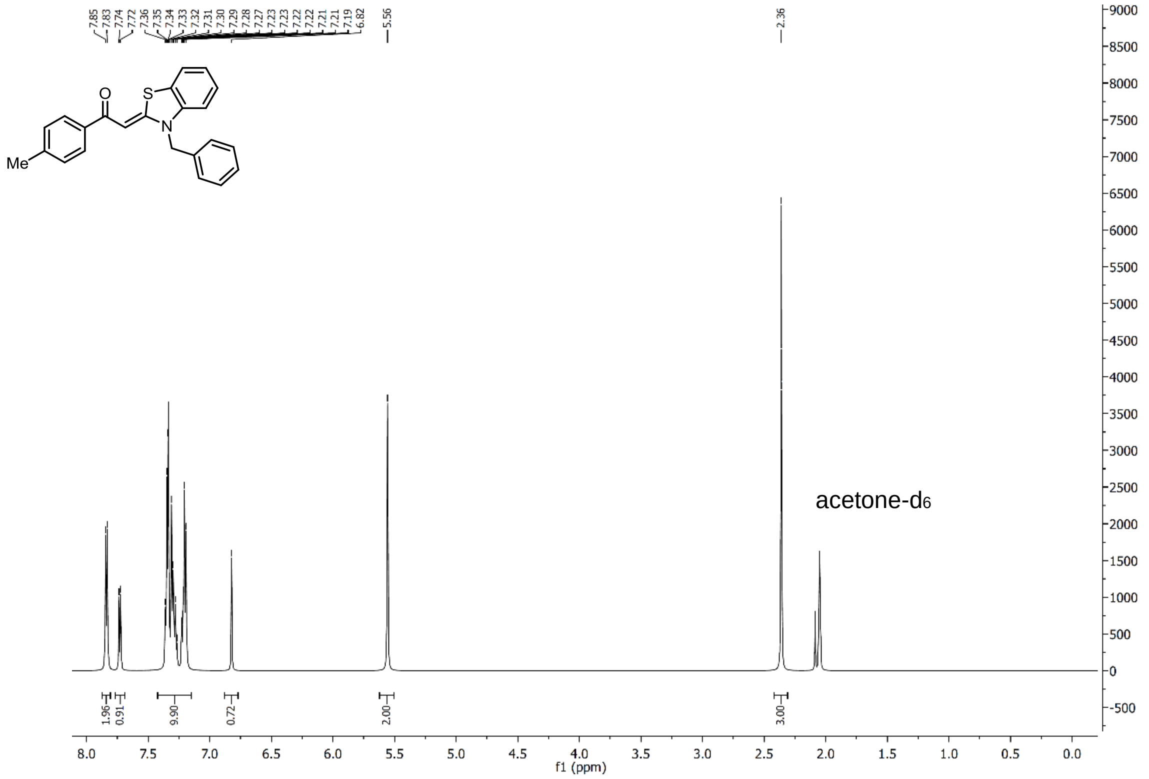

^{13}C NMR-spectrum of (*Z*)-2-(3-Benzyl)benzo[*d*]thiazol-2(3*H*)-ylidene)-1-(*p*-tolyl)ethan-1-one (3h) (acetone-d_6/CS_2 5:1, 150 MHz, 293 K)

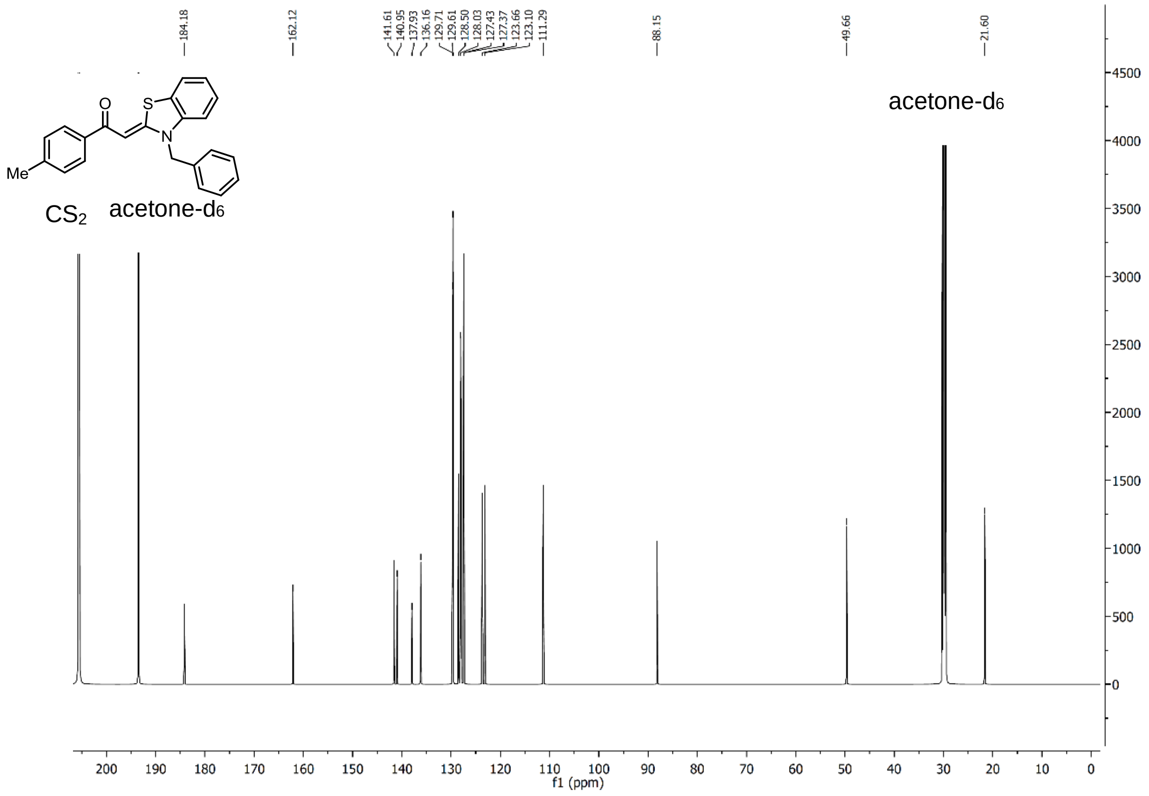

^{1}H NMR-spectrum of (*Z*)-2-(3-(4-Bromobenzyl)benzo[*d*]thiazol-2(3*H*)-ylidene)-1-phenylethan-1-one (3i) (acetone-d_6/CS_2 5:1, 300 MHz, 293 K)

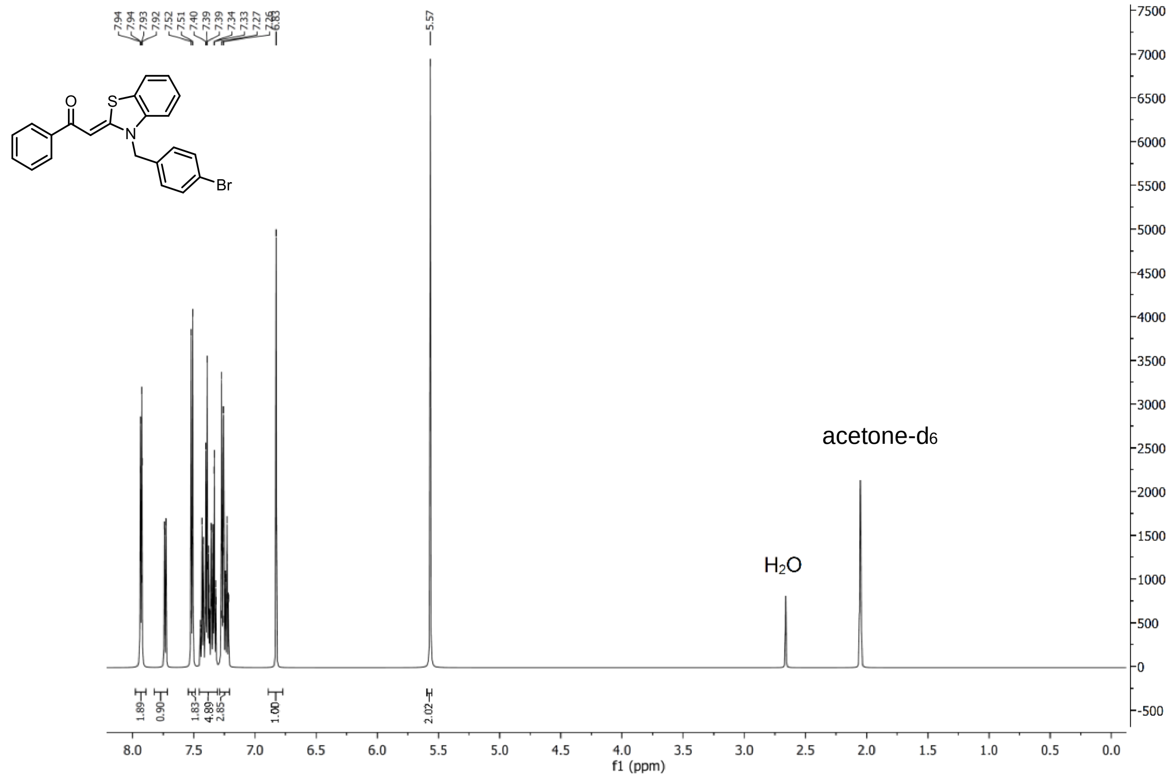

^{13}C NMR-spectrum of (*Z*)-2-(3-(4-Bromobenzyl)benzo[*d*]thiazol-2(3*H*)-ylidene)-1-phenylethan-1-one (3i) (acetone-d_6/CS_2 5:1, 75 MHz, 293 K)

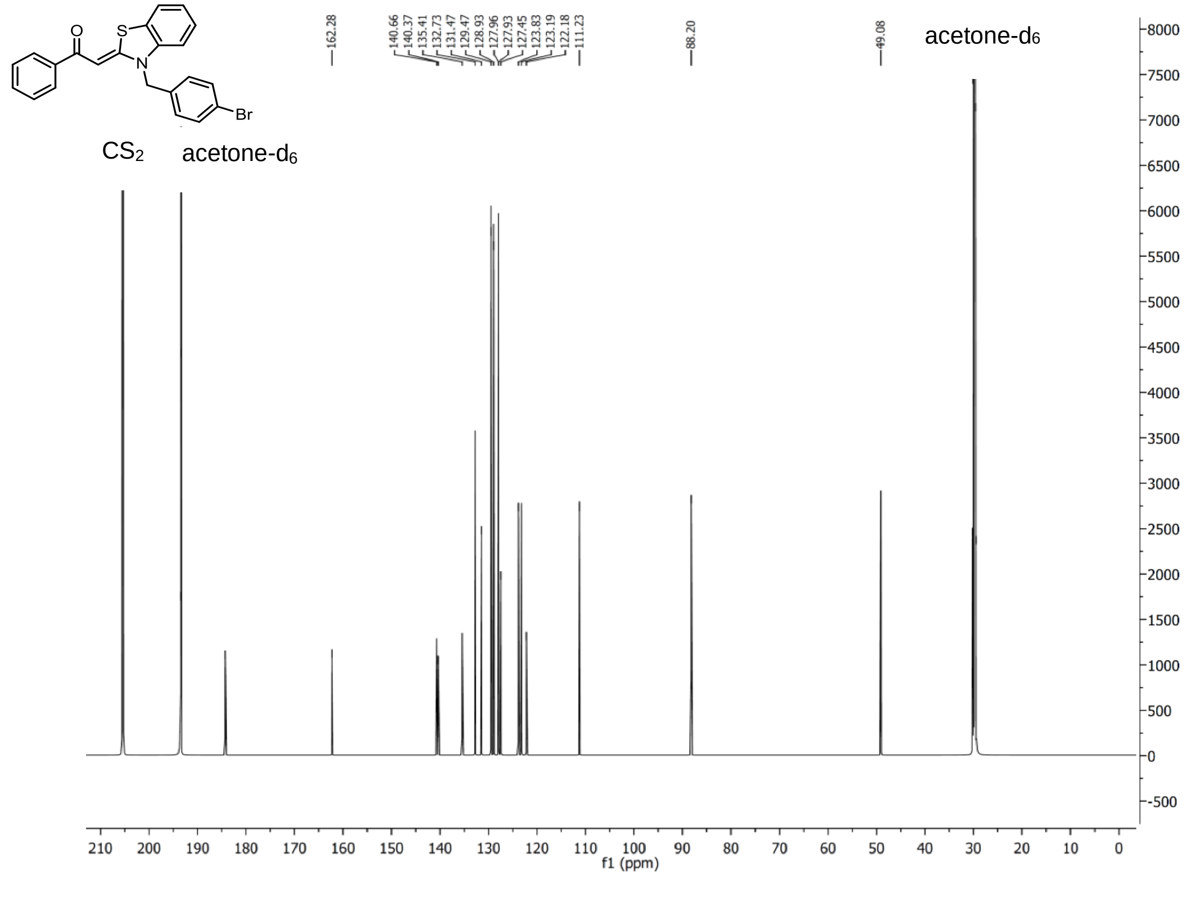

^{1}H NMR-spectrum von (*Z*)-2-(3-Benzylbenzo[*d*]thiazol-2 (3*H*)-ylidene)-1-phenylethan-1-one (3j) (acetone-d_6/CS_2 5:1, 300 MHz, 293 K)

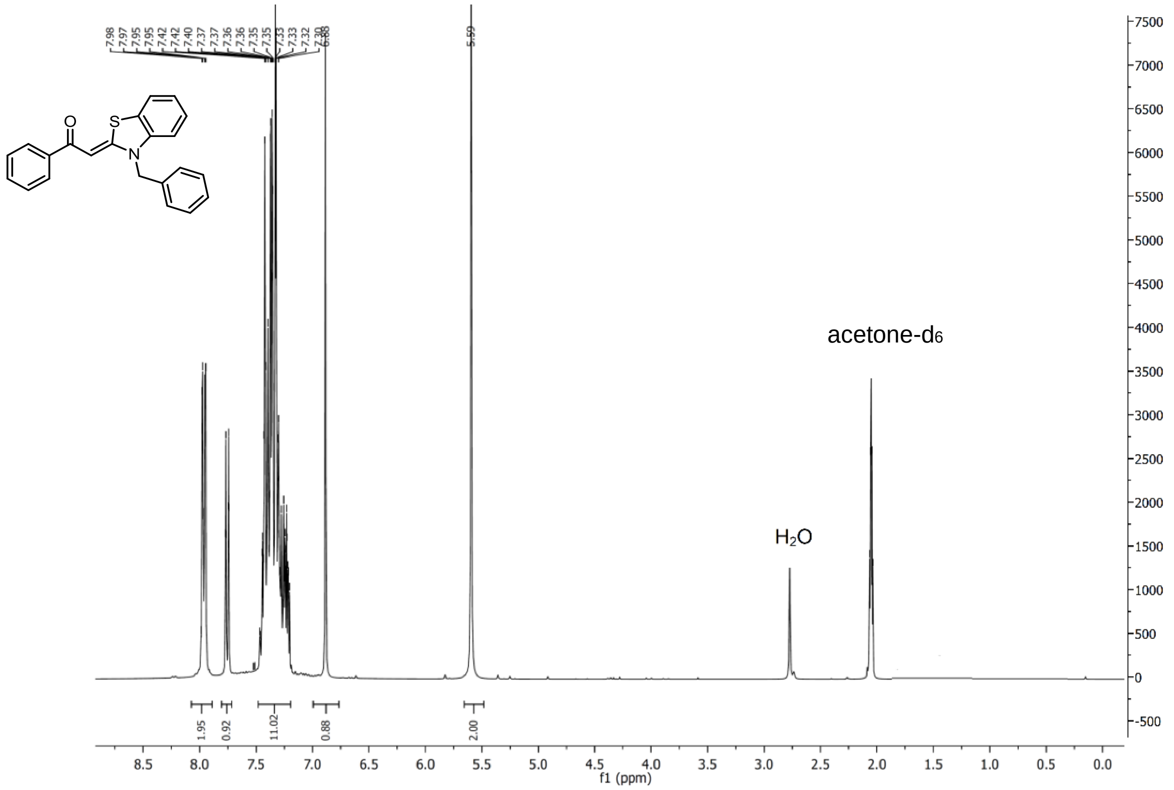

^{13}C NMR-spectrum von (*Z*)-2-(3-Benzylbenzo[*d*]thiazol-2(3*H*)-ylidene)-1-phenylethan-1-one (3j) (acetone-d_6/CS_2 5:1, 75 MHz, 293 K)

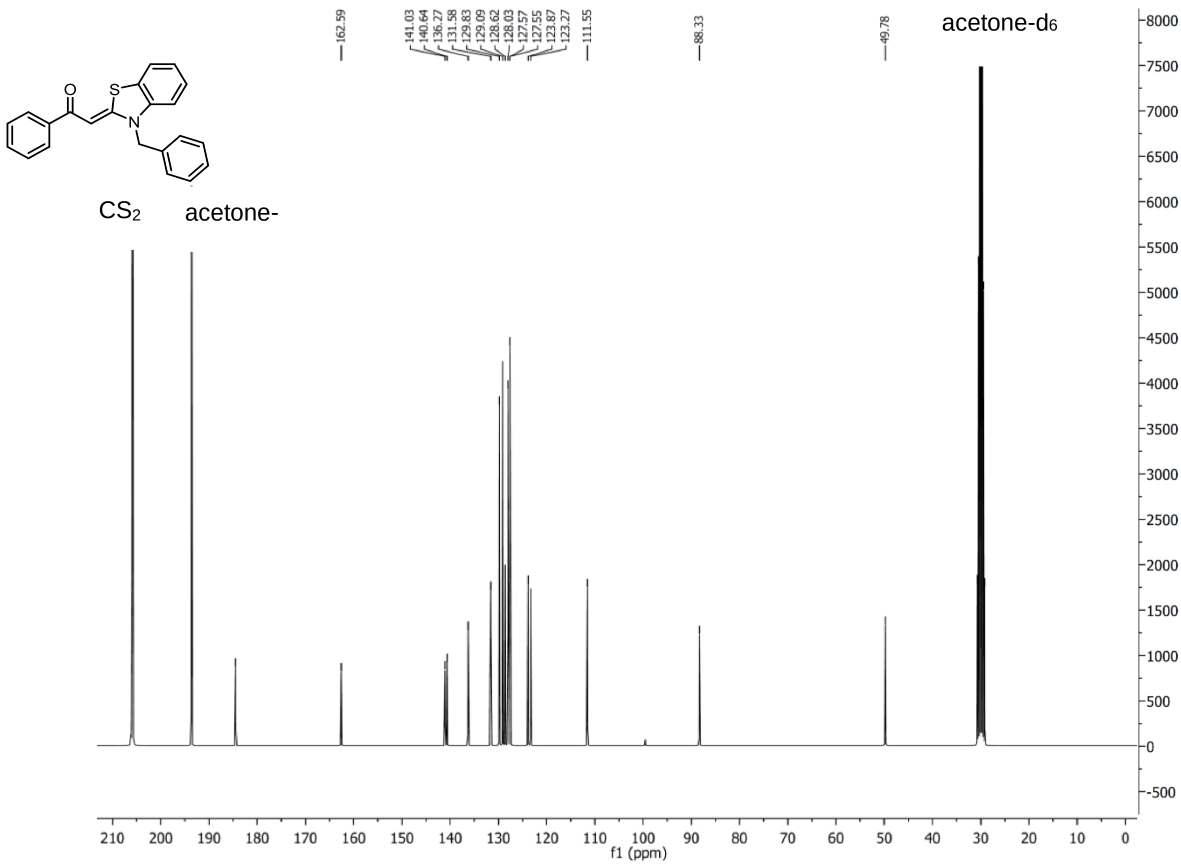

^{1}H NMR-spectrum of *(Z)*-2-(3-Methylbenzo[*d*]thiazol-2(3*H*)-yliden-1-phenylethan-1-one (3k) (acetone-d_6/CS_2 5:1, 600 MHz, 293 K)

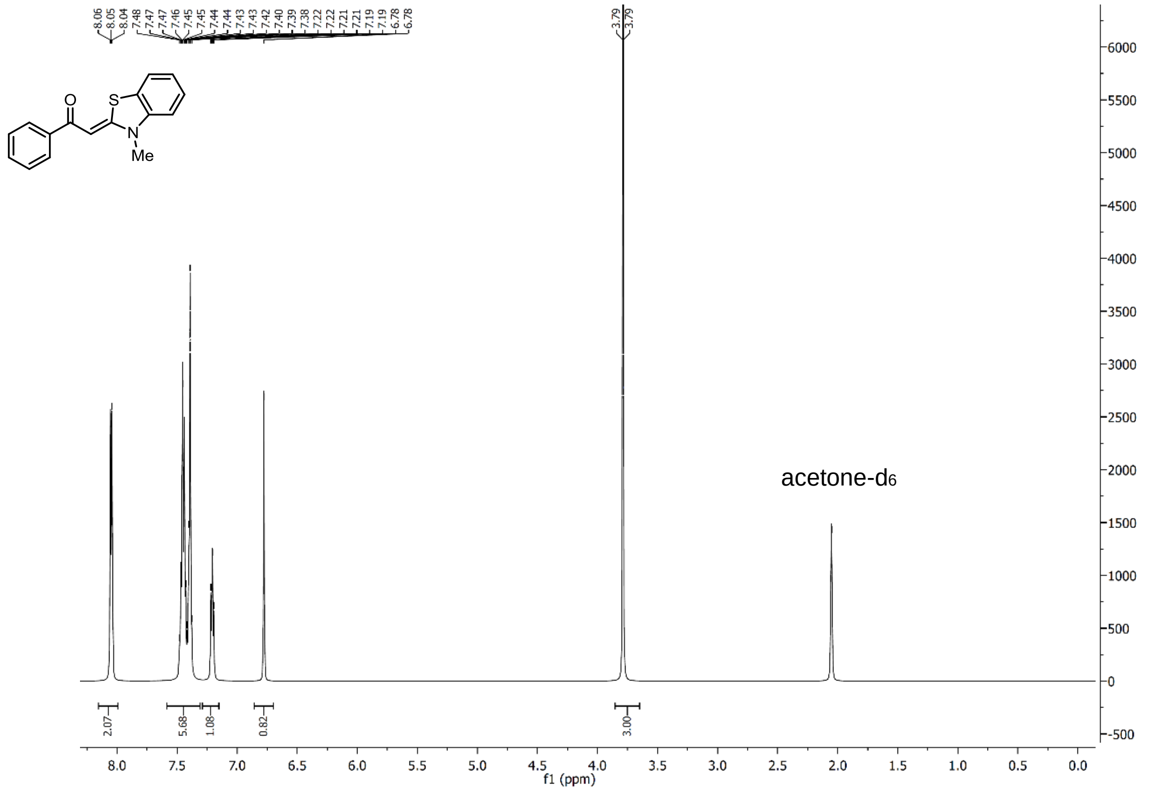

^{13}C NMR-spectrum of *(Z)*-2-(3-Methylbenzo[*d*]thiazol-2(3*H*)-ylidene-1-phenylethan-1-one (3k) (acetone-d_6/CS_2 5:1, 150 MHz, 293 K)

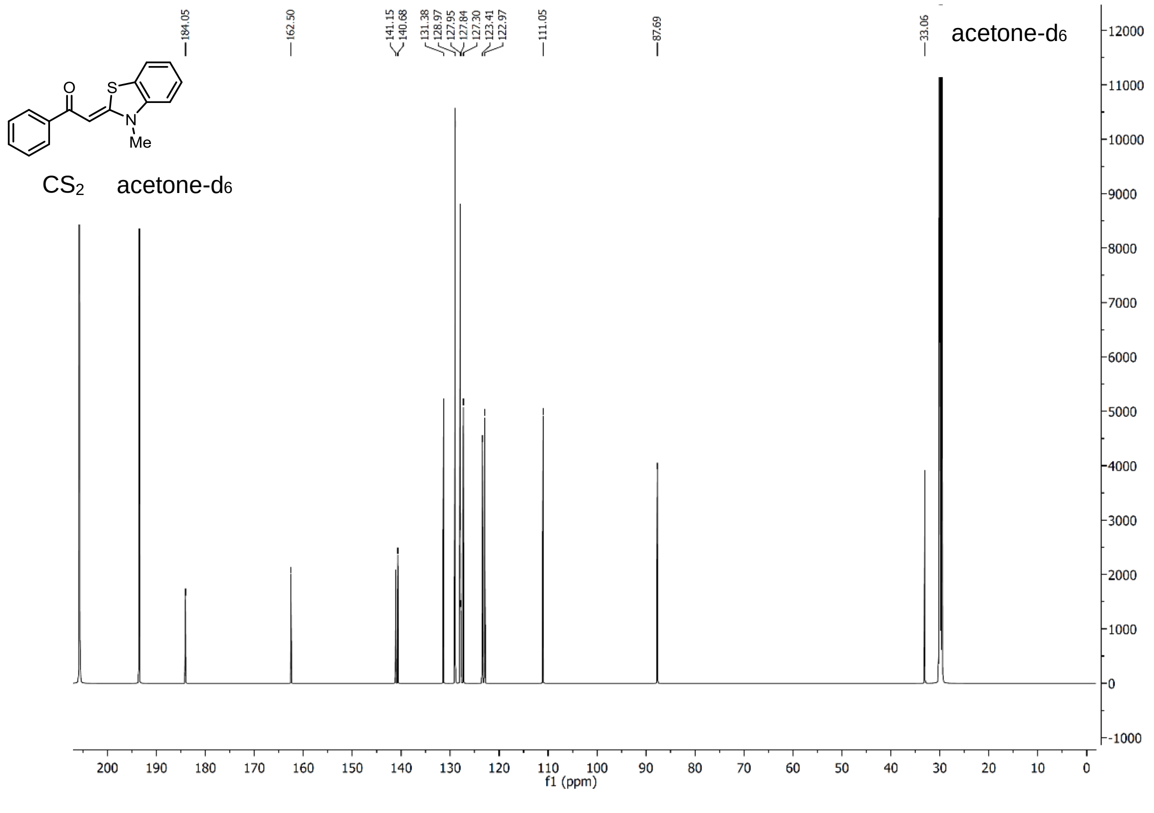

^{1}H NMR-spectrum of ((1*Z*)(3*E*)-2-(3-(4-Bromobenzyl)benzo[*d*]thiazol-2(3*H*)-ylidene)-1-(4-fluorophenyl)but-3-en-2-one (3l) (acetone-d_6/CS_2 5:1, 300 MHz, 293 K)

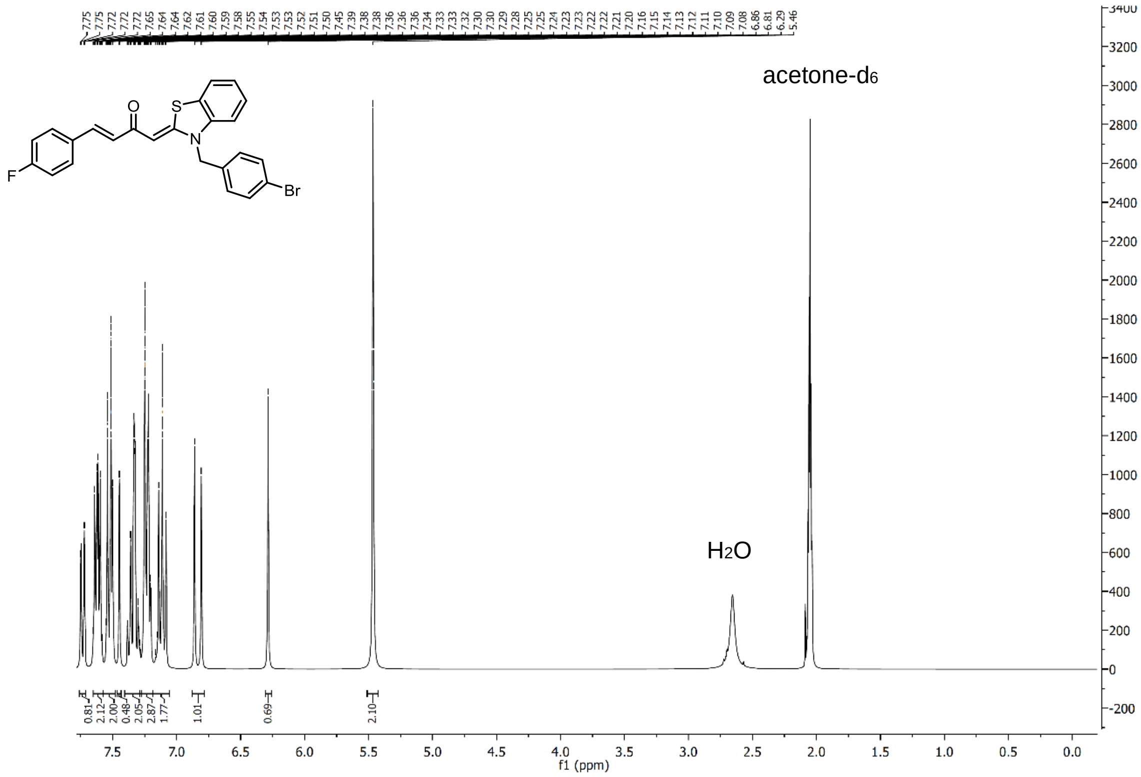

^{13}C NMR-spectrum of ((1*Z*)(3*E*)-2-(3-(4-Bromobenzyl)benzo[*d*]thiazol-2(3*H*)-ylidene)-1-(4-fluorophenyl)but-3-en-2-one (3l) (acetone-d_6/CS_2 5:1, 75 MHz, 293 K)

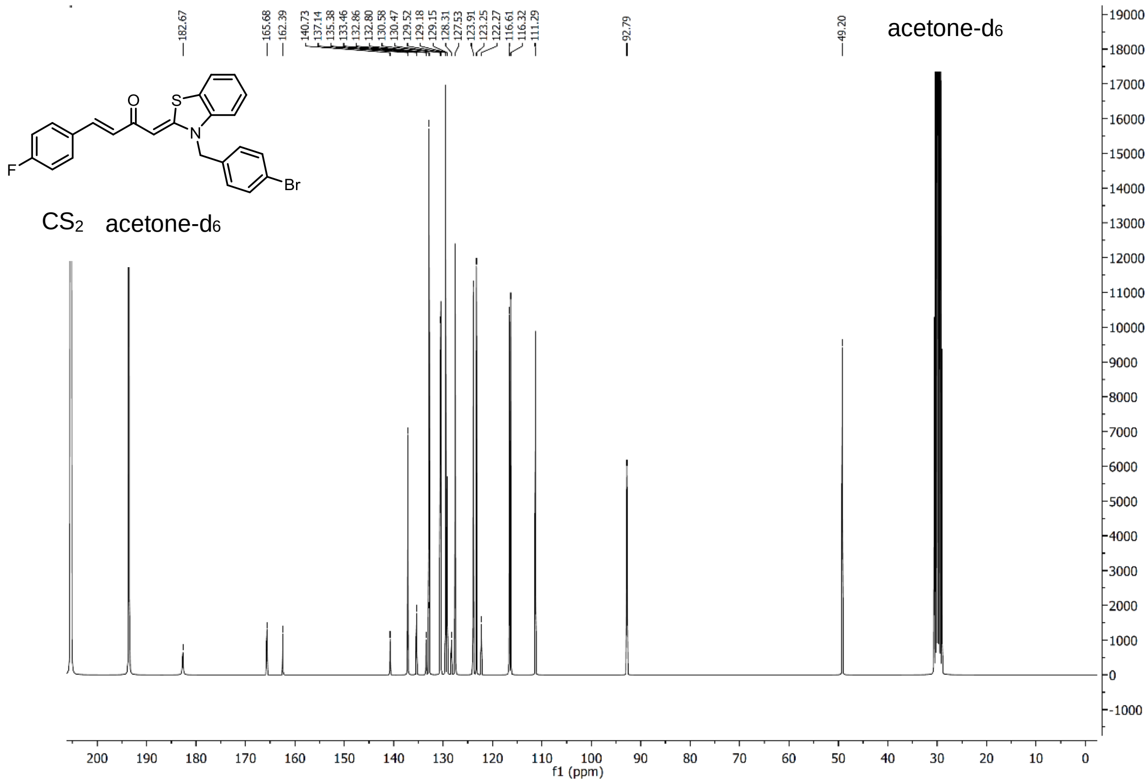

^{1}H NMR-spectrum of ((1*Z*)(3*E*)-2-(3-Benzyl)benzo[*d*]thiazol-2(3*H*)-ylidene)-1-(4-fluorophenyl)but-3-en-2-one (3m) (acetone-d_6/CS_2 5:1, 300 MHz, 293 K)

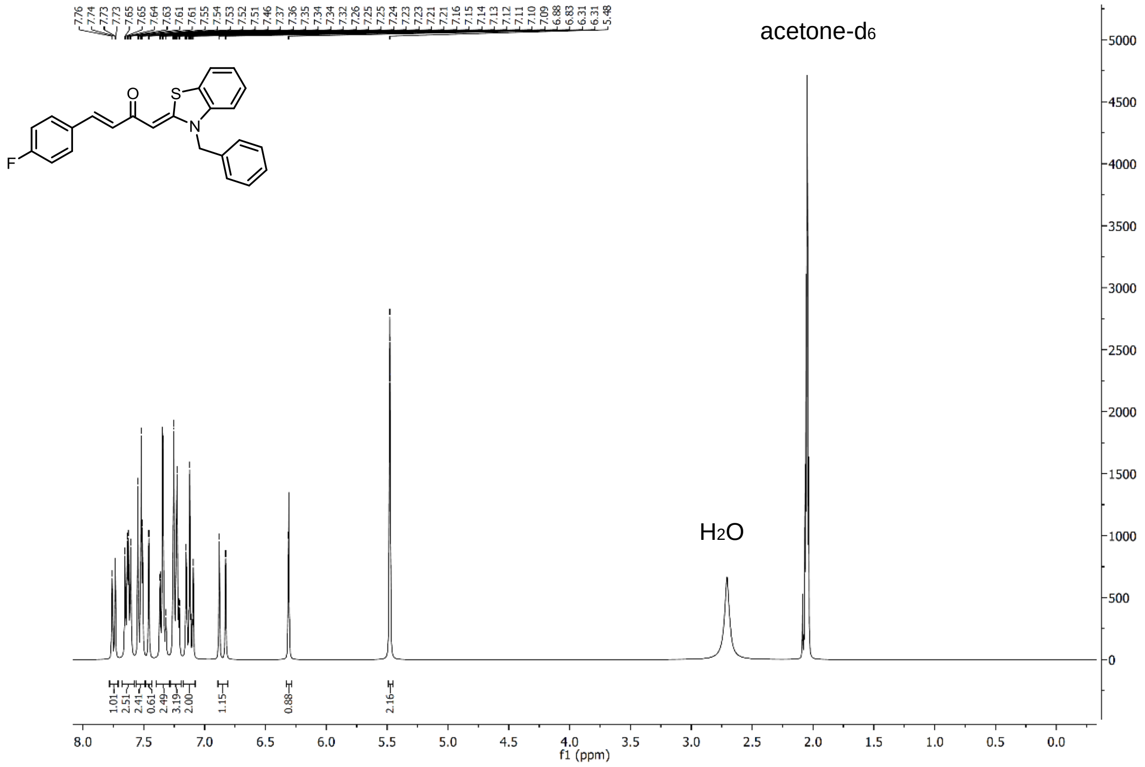

^{13}C NMR-spectrum of ((1*Z*)(3*E*)-2-(3-Benzyl)benzo[*d*]thiazol-2(3*H*)-ylidene)-1-(4-fluorophenyl)but-3-en-2-one (3m) (acetone-d_6/CS_2 5:1, 75 MHz, 293 K)

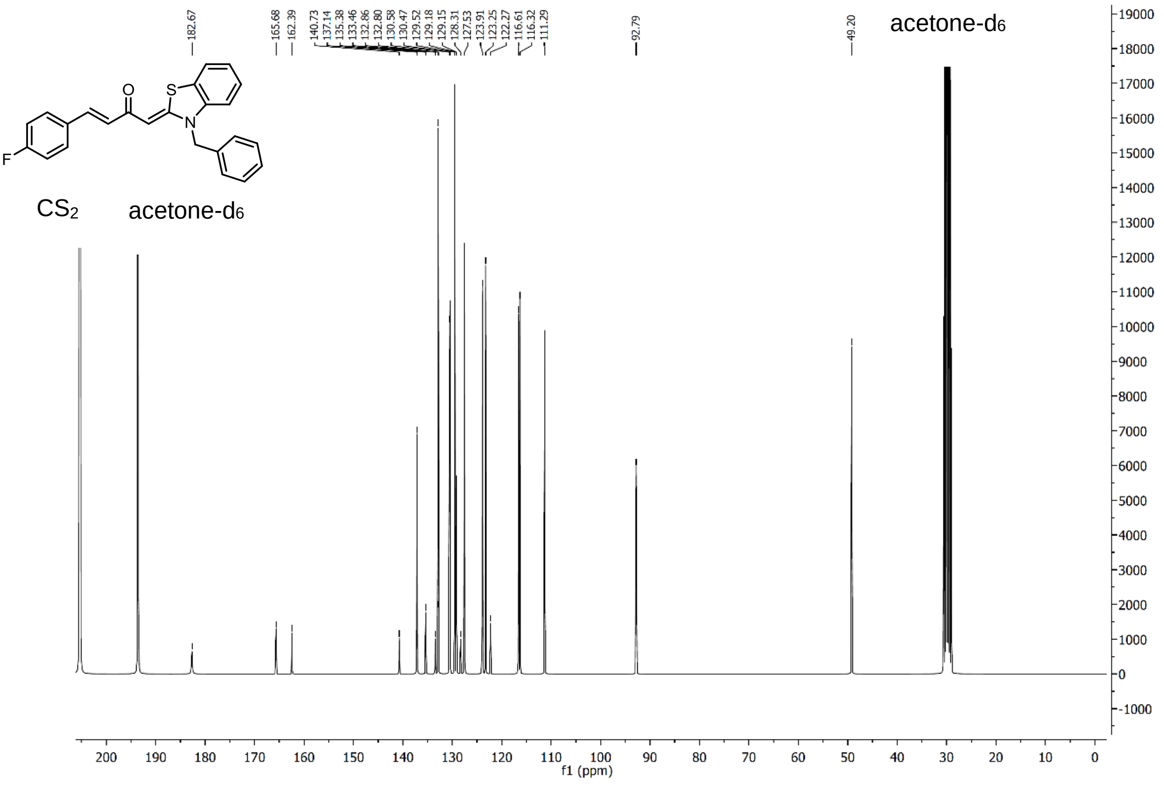

^{1}H NMR-spectrum of (*Z*)-2-(3-(4-Bromobenzyl)benzo[*d*]thiazol-2(3*H*)-ylidene)-1-(4-fluorophenyl)ethan-1-one (3n) (acetone-d_6/CS_2 5:1, 600 MHz, 293 K)

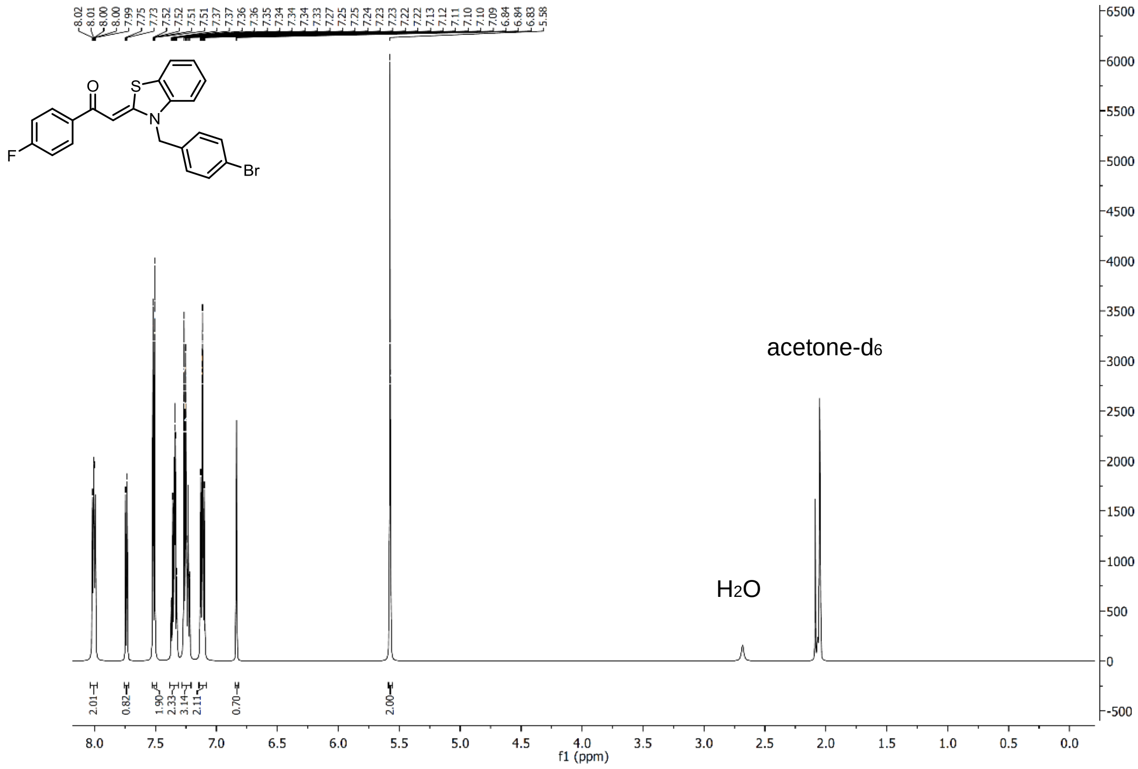

^{13}C NMR-spectrum of (*Z*)-2-(3-(4-Bromobenzyl)benzo[*d*]thiazol-2(3*H*)-ylidene)-1-(4-fluorophenyl)ethan-1-one (3n) (acetone-d_6/CS_2 5:1, 150 MHz, 293 K)

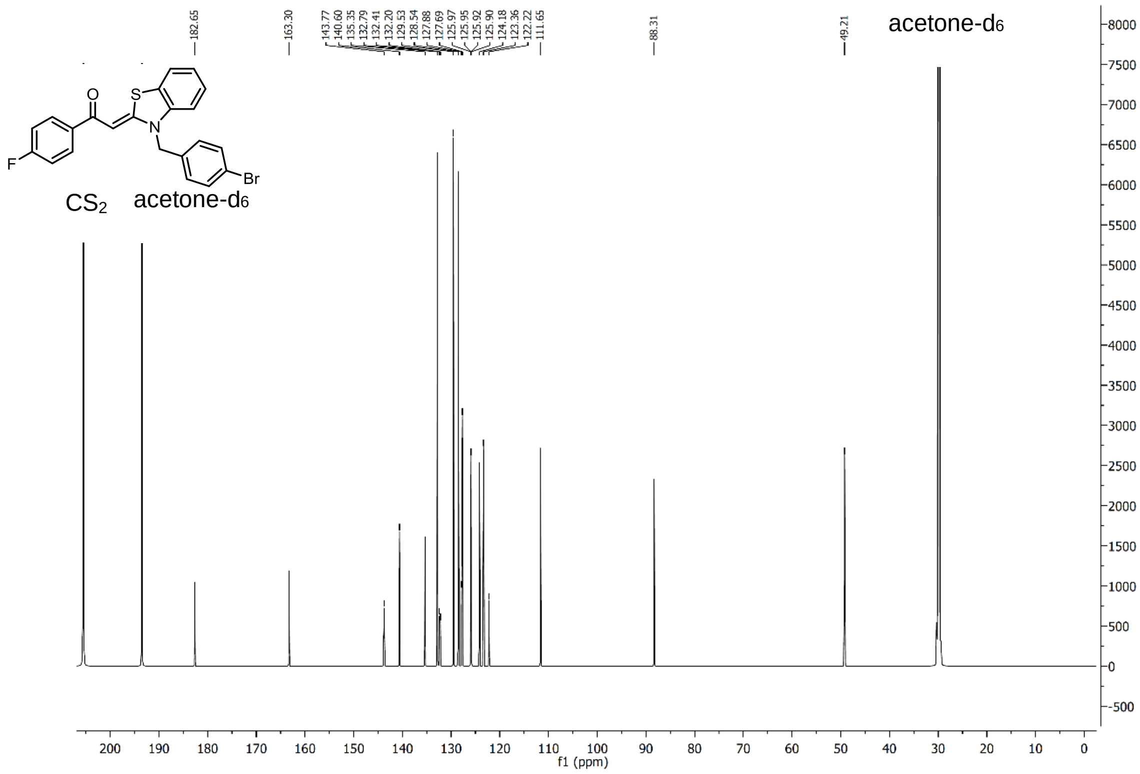

^{1}H NMR-spectrum of (*Z*)-2-(3-Benzyl)benzo[*d*]thiazol-2(3*H*)-ylidene)-1-(4-fluorophenyl)ethan-1-one (3o) (acetone-d_6/CS_2 5:1, 600 MHz, 293 K)

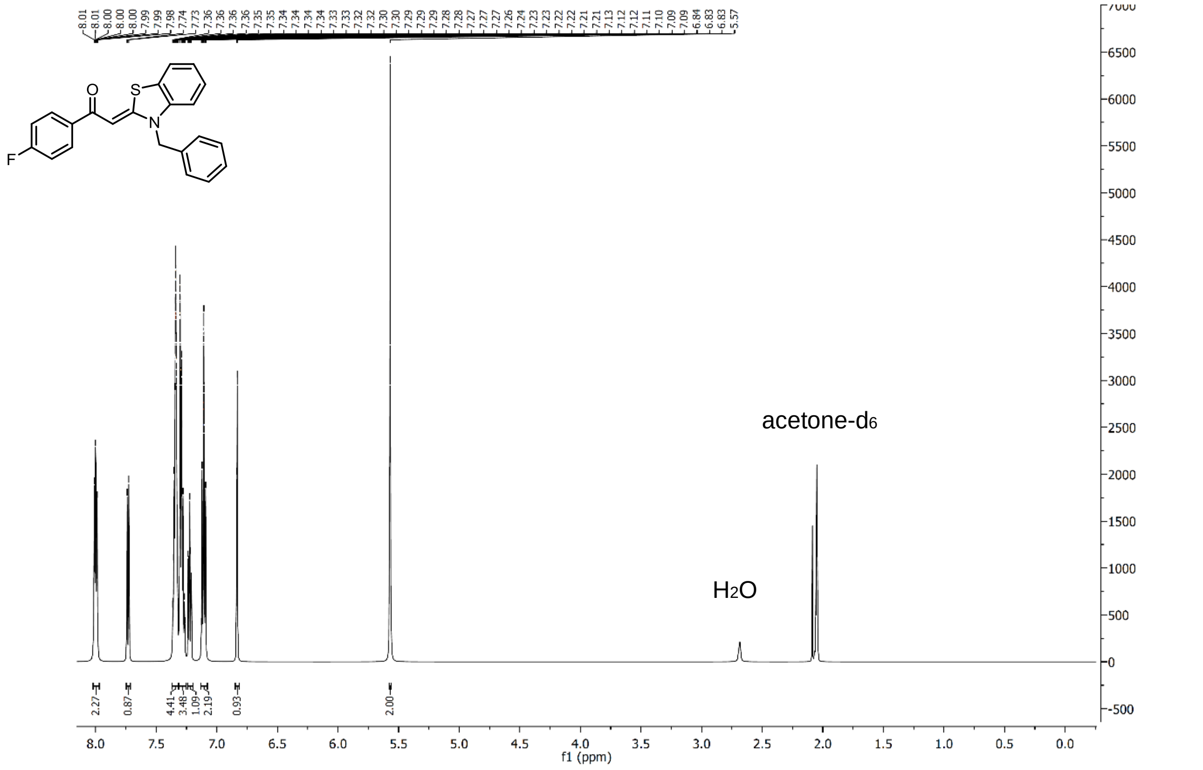

^{13}C NMR-spectrum of (*Z*)-2-(3-Benzyl)benzo[*d*]thiazol-2(3*H*)-ylidene)-1-(4-fluorophenyl)ethan-1-one (3o) (acetone-d_6/CS_2 5:1, 150 MHz, 293 K)

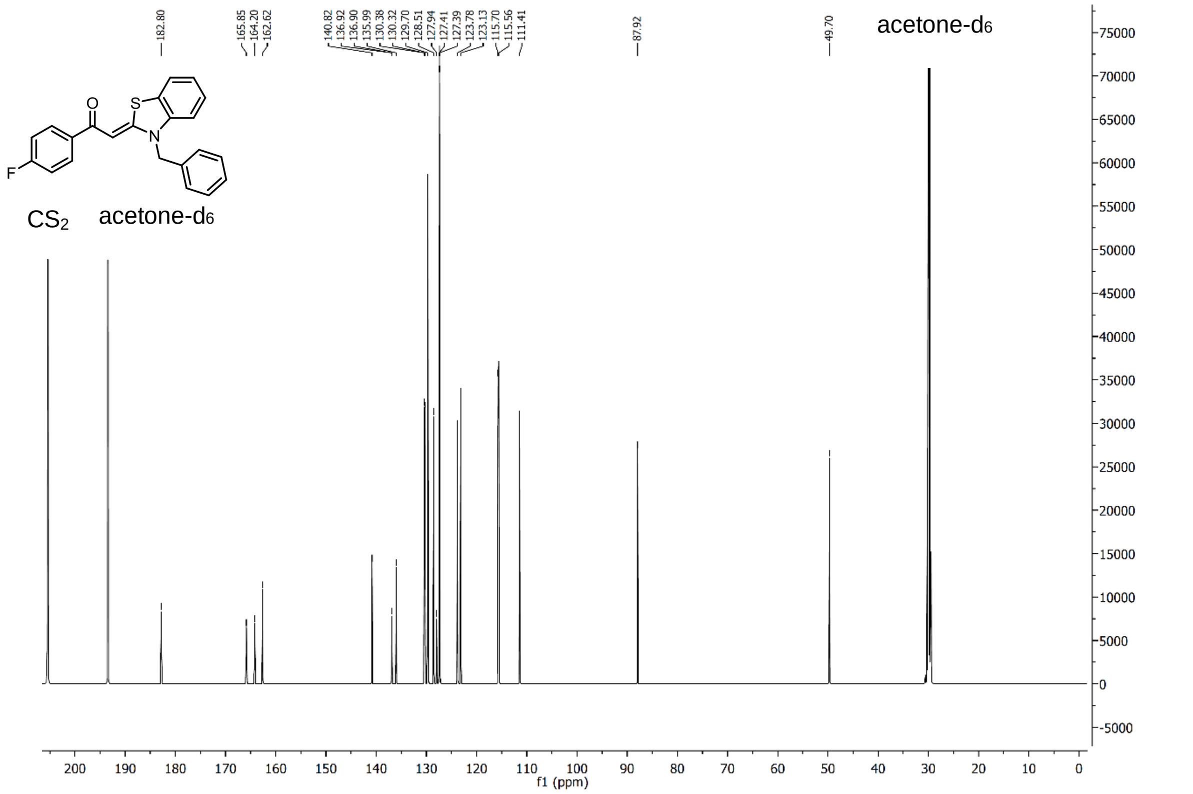

^{1}H NMR-spectrum of (*Z*)-2-(3-Benzyl)benzo[*d*]thiazol-2(3*H*)-ylidene)-1-(3-fluorophenyl)ethan-1-one (3p) (acetone-d$_6$/CS$_2$ 5:1, 600 MHz, 293 K)

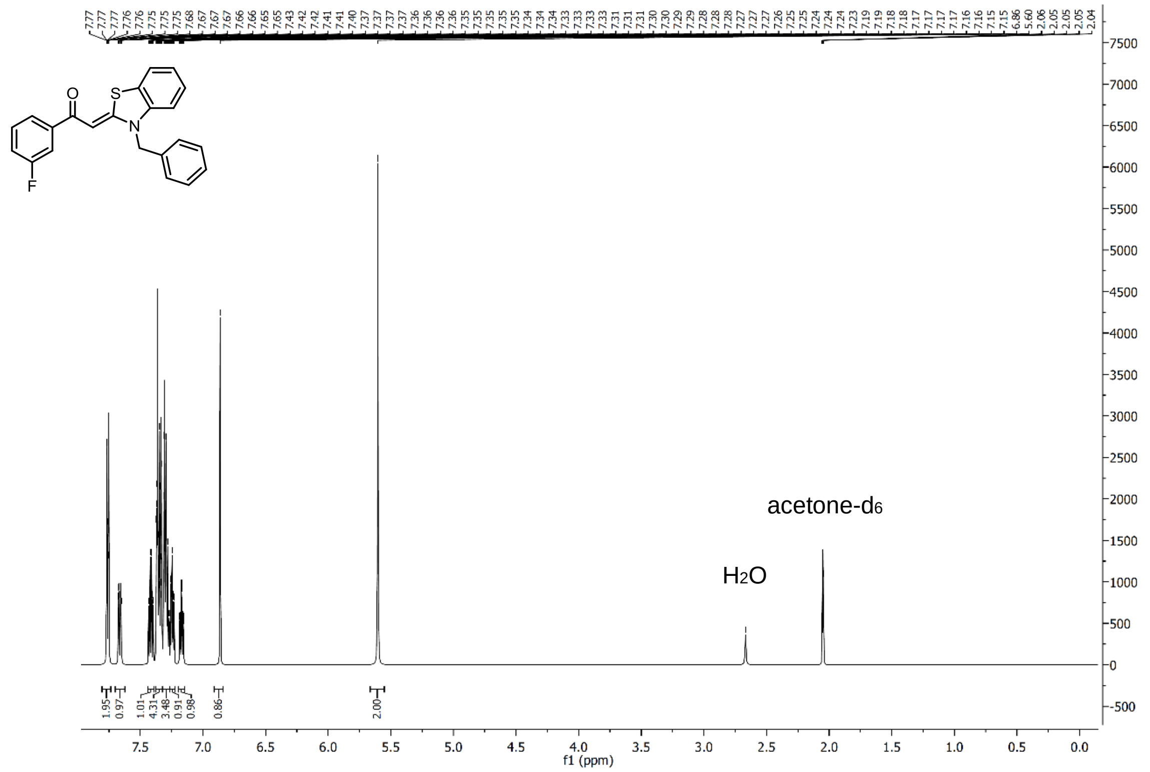

^{13}C NMR-spectrum of (*Z*)-2-(3-Benzyl)benzo[*d*]thiazol-2(3*H*)-ylidene)-1-(3-fluorophenyl)ethan-1-one (3p) (acetone-d$_6$/CS$_2$ 5:1, 150 MHz, 293 K)

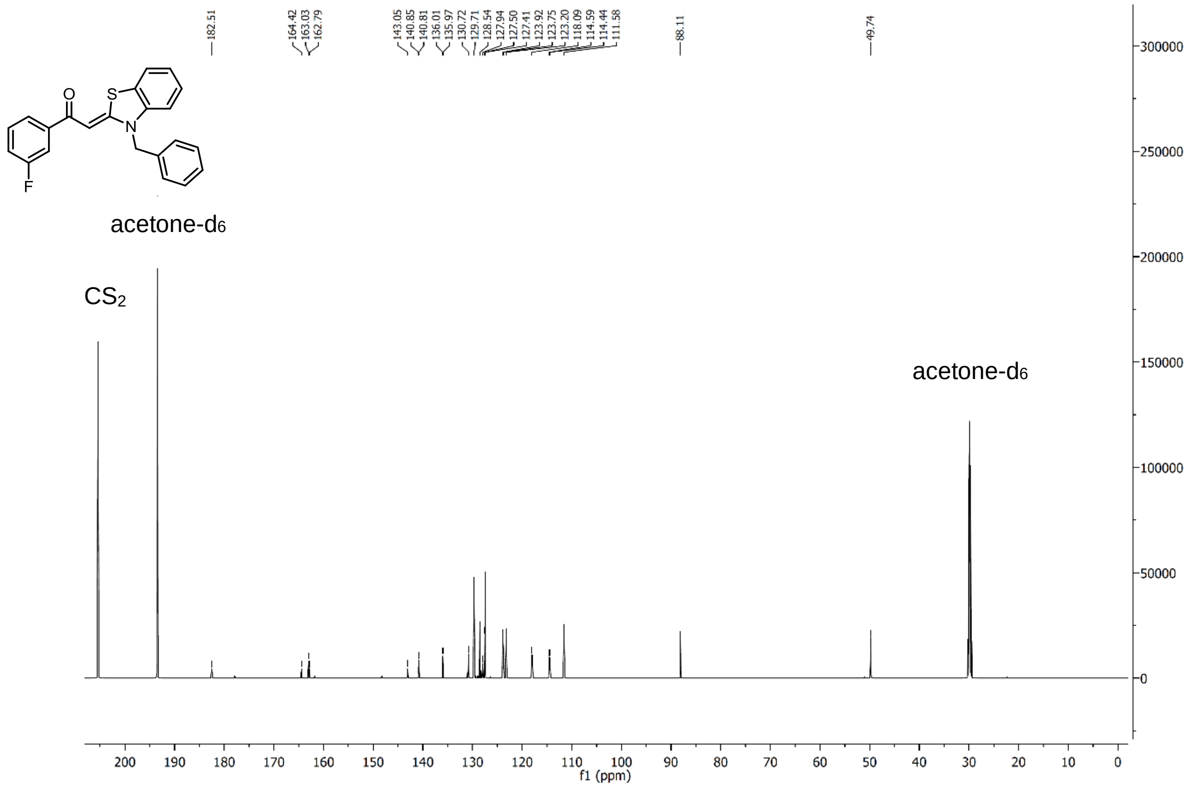

^{1}H NMR-spectrum of (*Z*)-2-(3-Benzyl)benzo[*d*]thiazol-2(3*H*)-ylidene)-1-(2-fluorophenyl)ethan-1-one (3q) (acetone-d_6/CS_2 5:1, 300 MHz, 293 K)

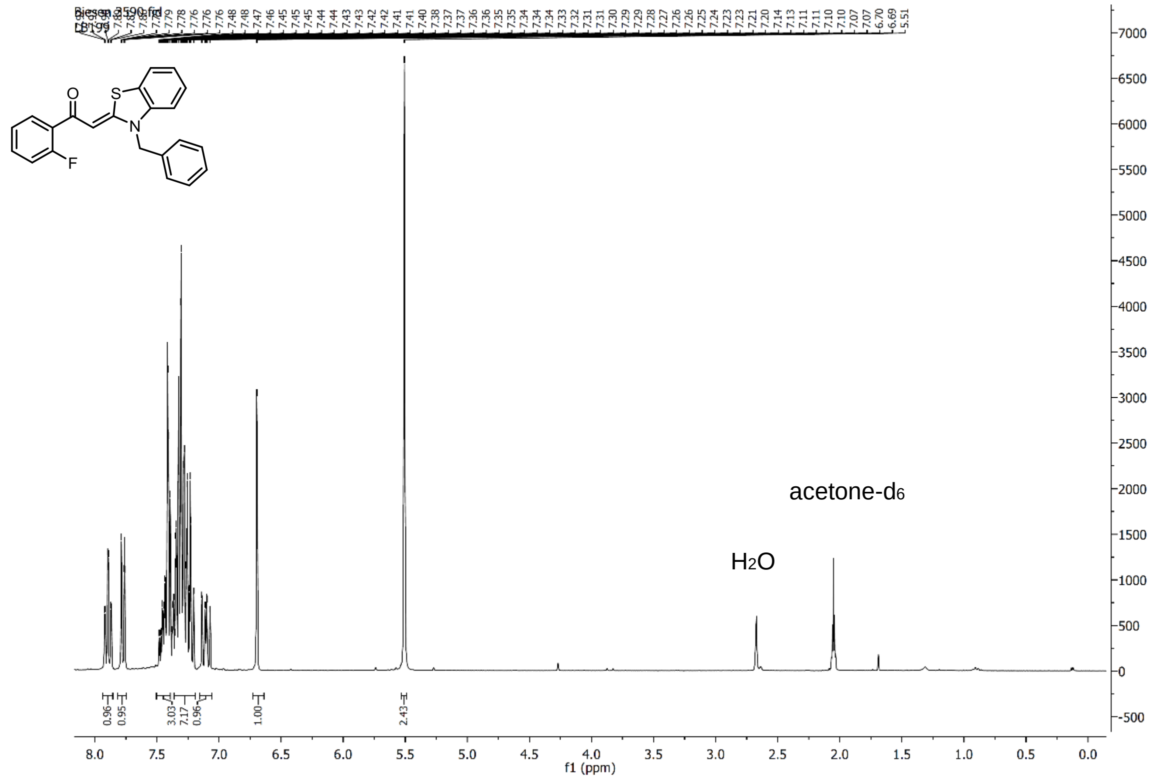

^{13}C NMR-spectrum of (*Z*)-2-(3-Benzyl)benzo[*d*]thiazol-2(3*H*)-ylidene)-1-(2-fluorophenyl)ethan-1-one (3q) (acetone-d_6/CS_2 5:1, 75 MHz, 293 K)

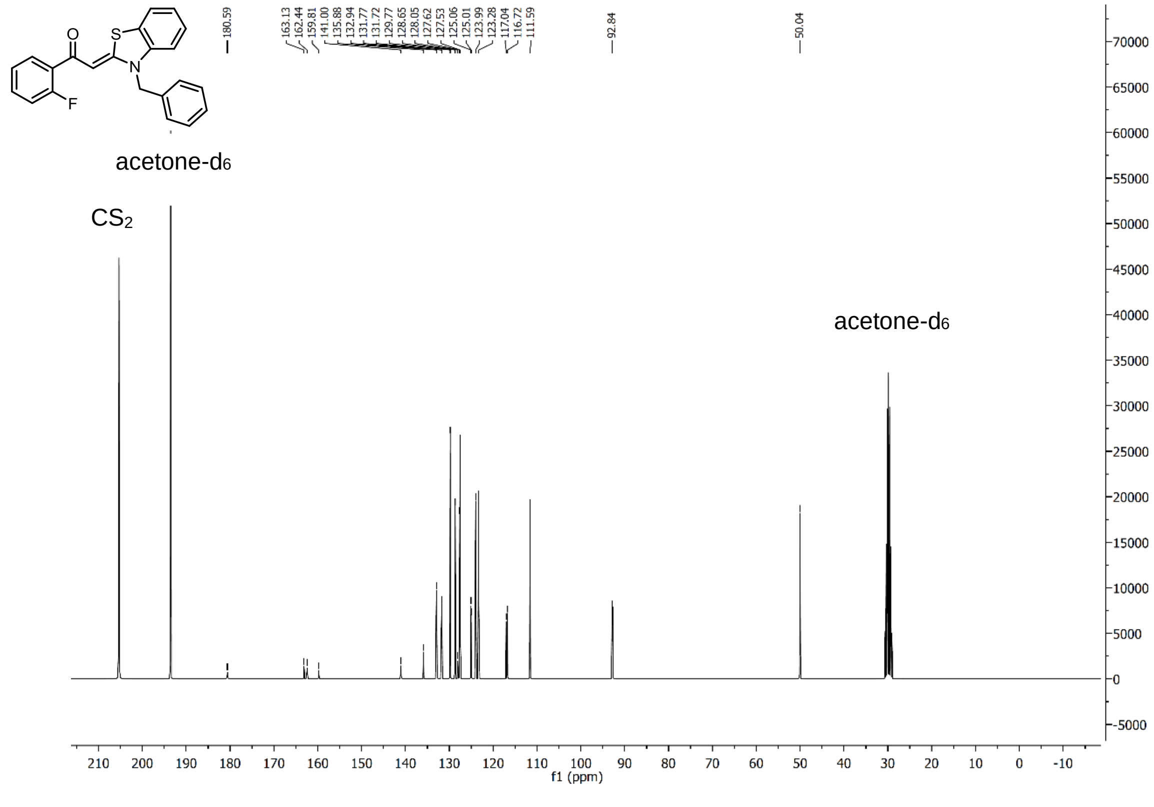

^{1}H NMR-spectrum of (*Z*)-2-(3-(4-Bromobenzyl)benzo[*d*]thiazol-2(3*H*)-ylidene)-1-(4-chlorophenyl)ethan-1-one (3r) (acetone-d_6/CS_2 5:1, 600 MHz, 293 K)

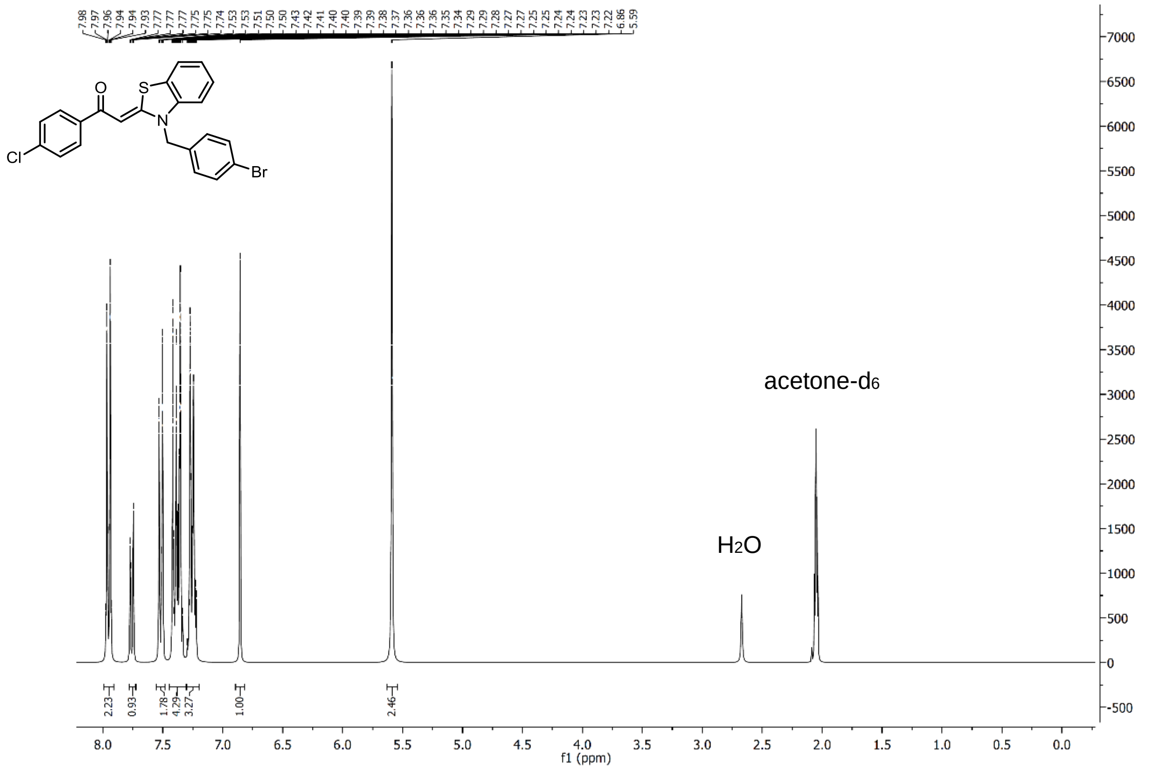

^{13}C NMR-spectrum of (*Z*)-2-(3-(4-Bromobenzyl)benzo[*d*]thiazol-2(3*H*)-ylidene)-1-(4-chlorophenyl)ethan-1-one (3r) (acetone-d_6/CS_2 5:1, 150 MHz, 293 K)

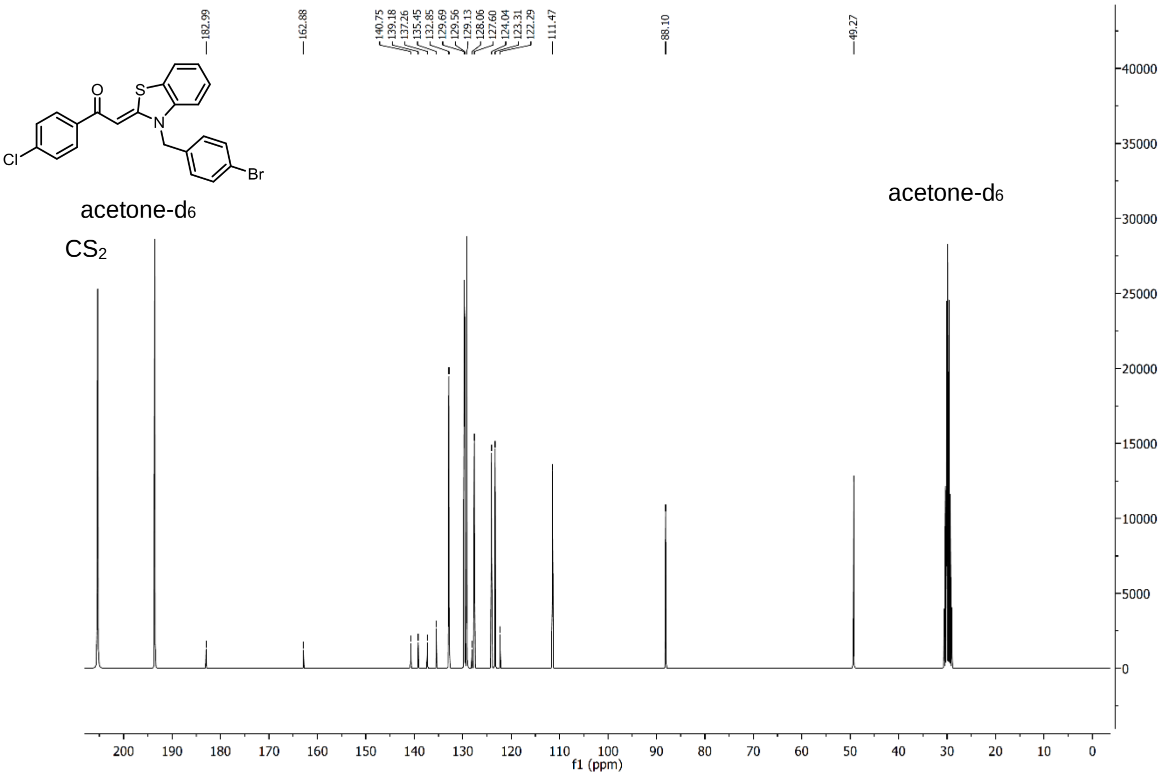

^{1}H NMR-spectrum of (*Z*)-2-(3-Benzyl)benzo[*d*]thiazol-2(3*H*)-ylidene)-1-(4-chlorophenyl)-ethan-1-one (3s) (acetone-d_6/CS_2 5:1, 300 MHz, 293 K)

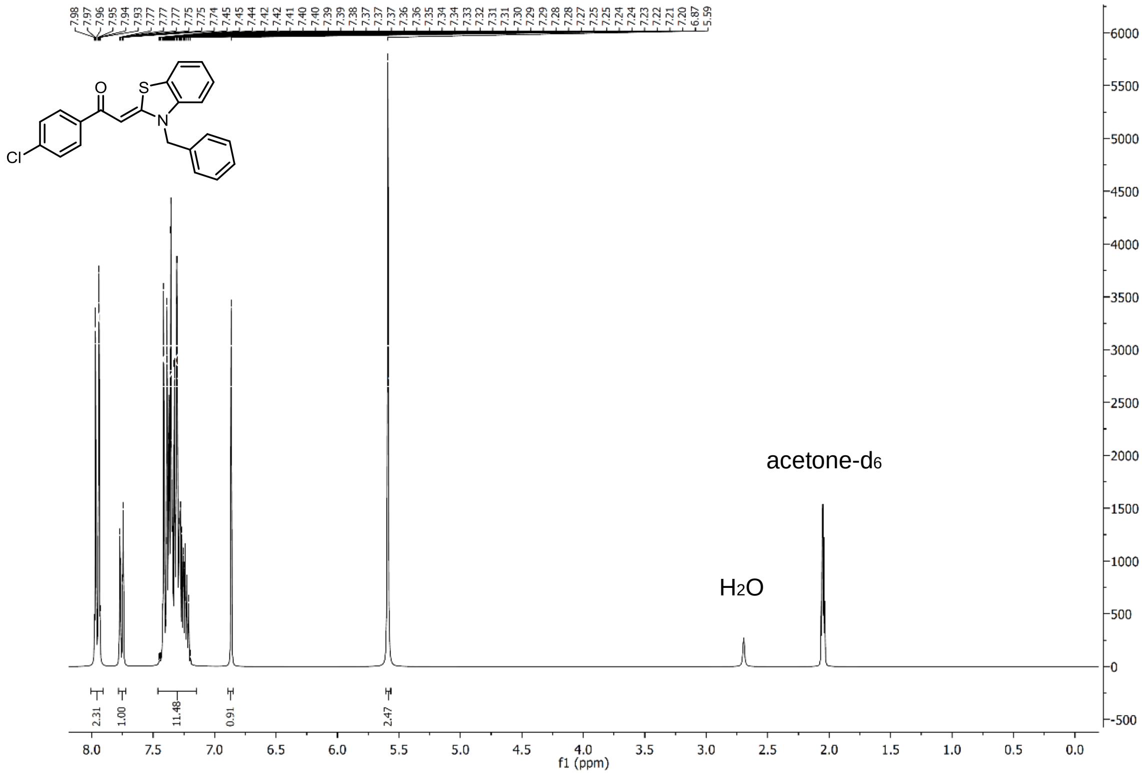

^{13}C NMR-spectrum of (*Z*)-2-(3-Benzyl)benzo[*d*]thiazol-2(3*H*)-ylidene)-1-(4-chlorophenyl)-ethan-1-one (3s) (acetone-d_6/CS_2 5:1, 75 MHz, 293 K)

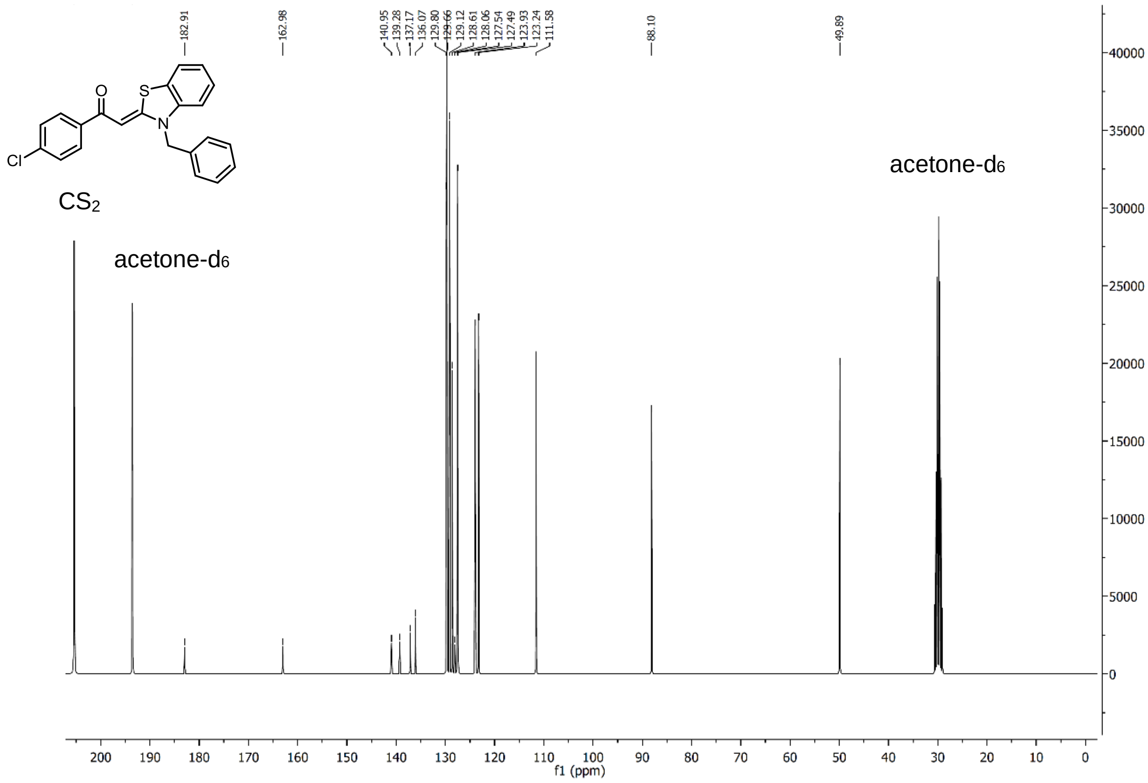

^{1}H NMR-spectrum of (*Z*)-2-(3-(4-Bromobenzyl)benzo[*d*]thiazol-2(3*H*)-ylidene)-1-(6-chloropyridine-3-yl)phenylethan-1-one (3t) (acetone-d_6/CS_2 5:1, 300 MHz, 293 K)

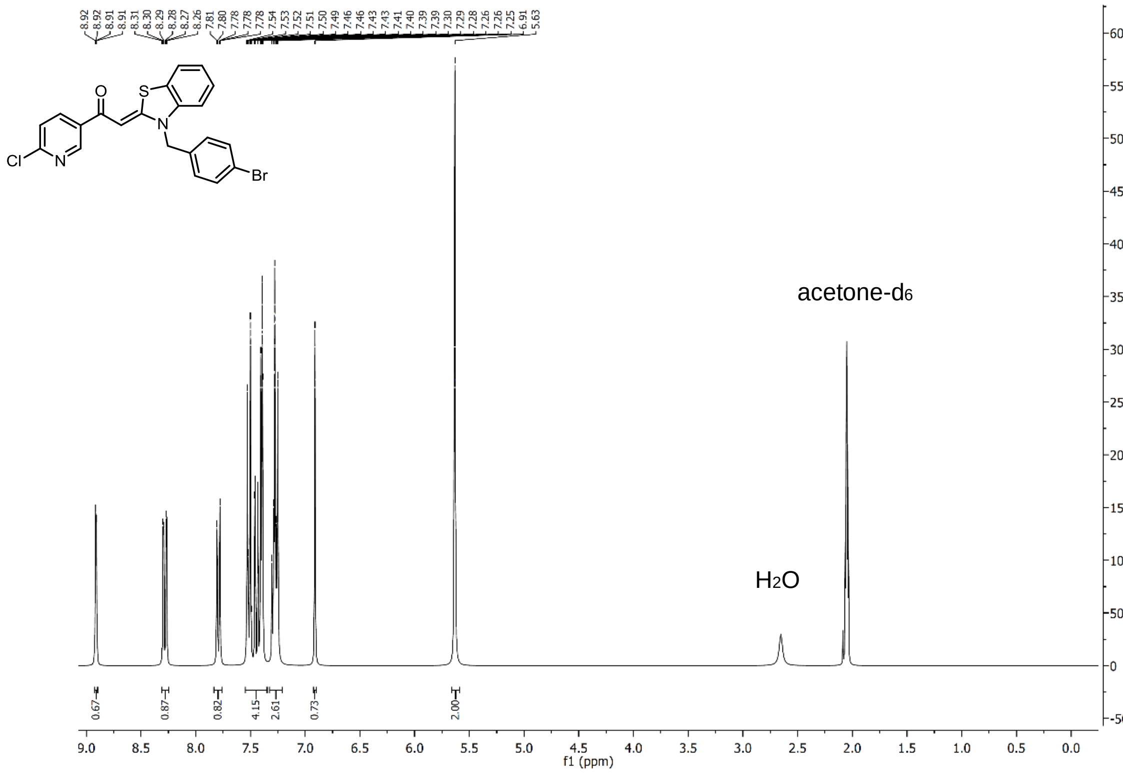

^{13}C NMR-spectrum of (*Z*)-2-(3-(4-Bromobenzyl)benzo[*d*]thiazol-2(3*H*)-ylidene)-1-(6-chloropyridine-3-yl)phenylethan-1-one (3t) (acetone-d_6/CS_2 5:1, 75 MHz, 293 K)

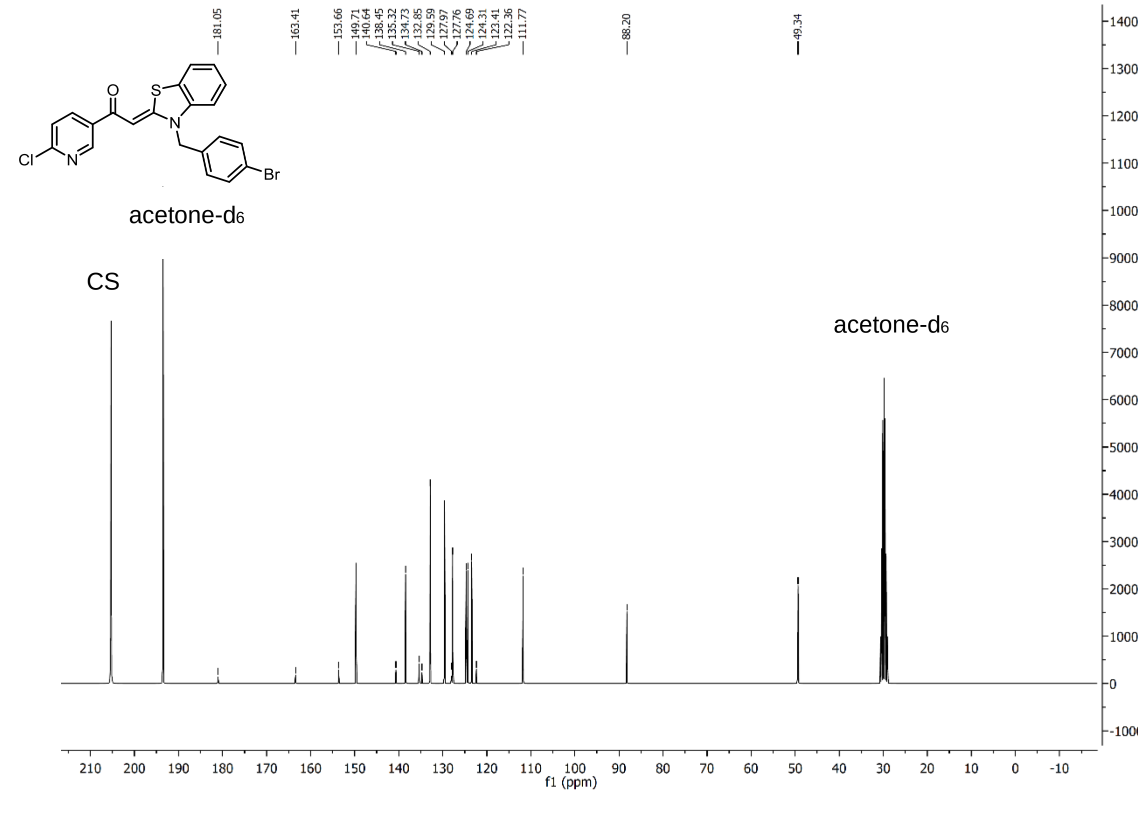

^{1}H **NMR-spectrum of (*Z*)-2-(3-Benzyl)benzo[*d*]thiazol-2(3*H*)-ylidene)-1-(6-chloropyridine-3-yl)phenylethan-1-one (3u) (acetone-d_6/CS_2 5:1, 300 MHz, 293 K)**

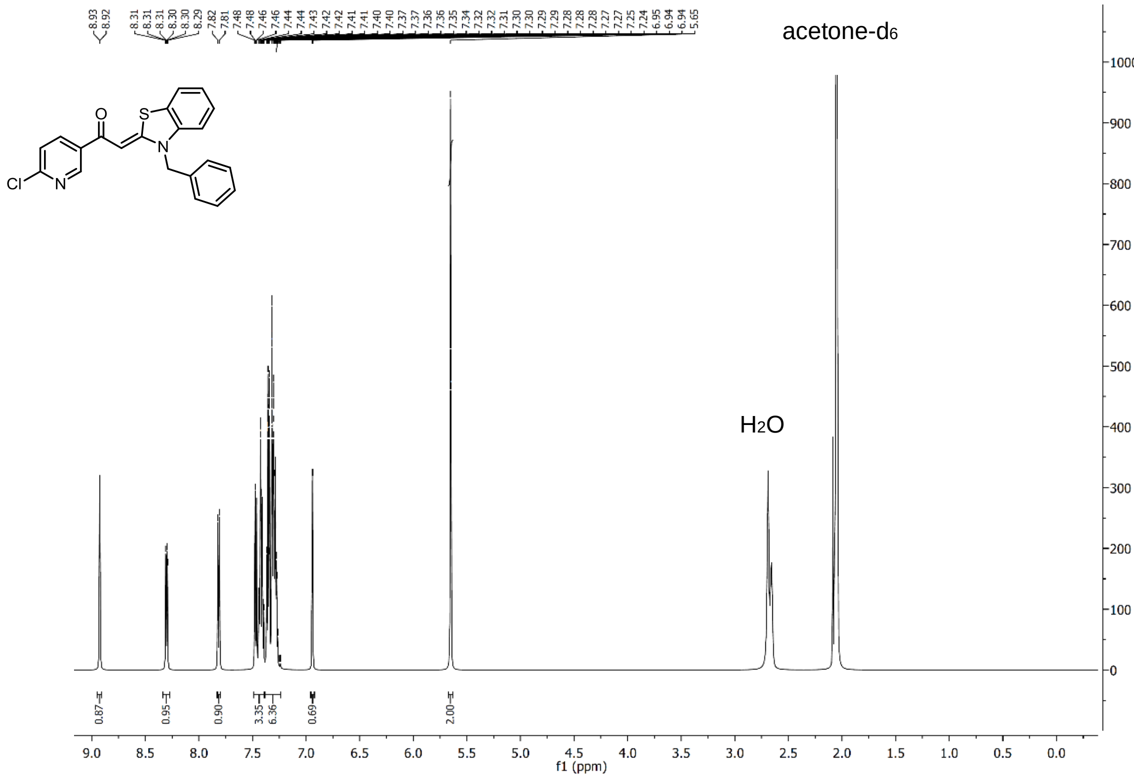

^{13}C **NMR-spectrum of (*Z*)-2-(3-Benzyl)benzo[*d*]thiazol-2(3*H*)-ylidene)-1-(6-chloropyridine-3-yl)phenylethan-1-one (3u) (acetone-d_6/CS_2 5:1, 75 MHz, 293 K)**

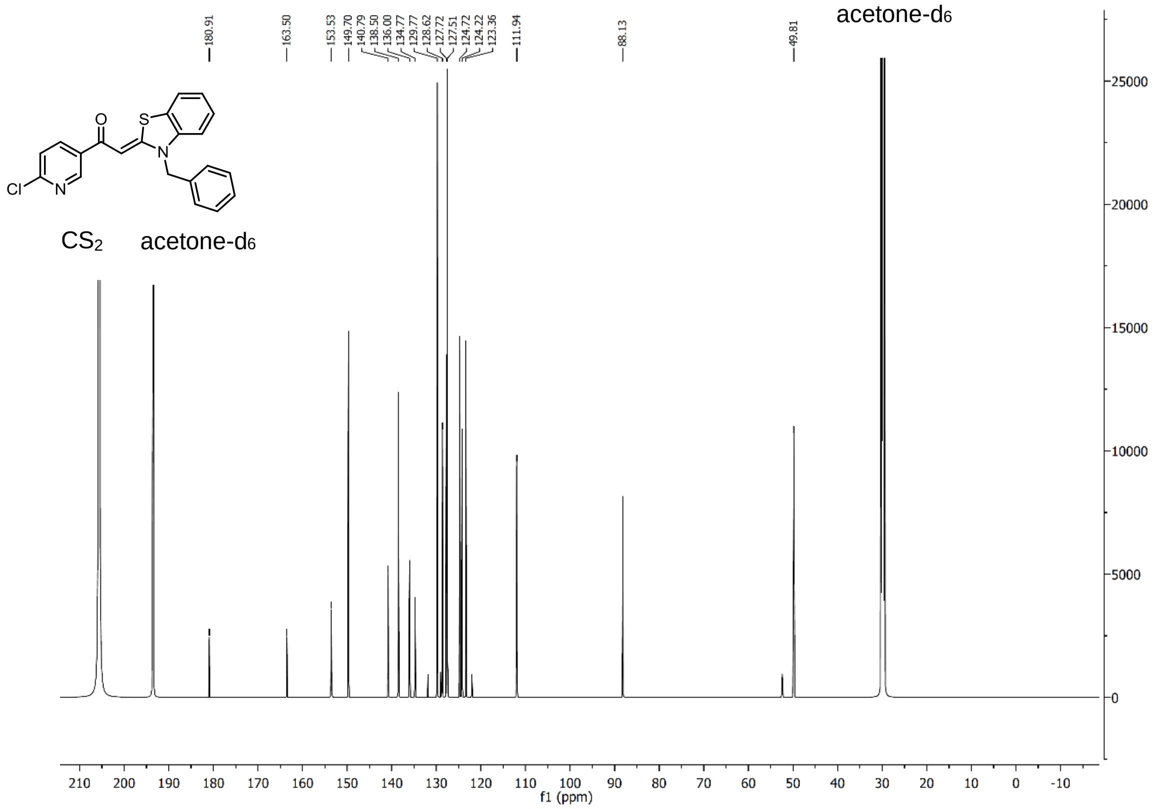

^{1}H NMR-spectrum of (*Z*)-2-(3-(4-Bromobenzyl)benzo[*d*]thiazol-2(3*H*)-ylidene)-1-(4-bromophenyl)ethan-1-one (3v) (acetone-d_6/CS_2 5:1, 500 MHz, 293 K)

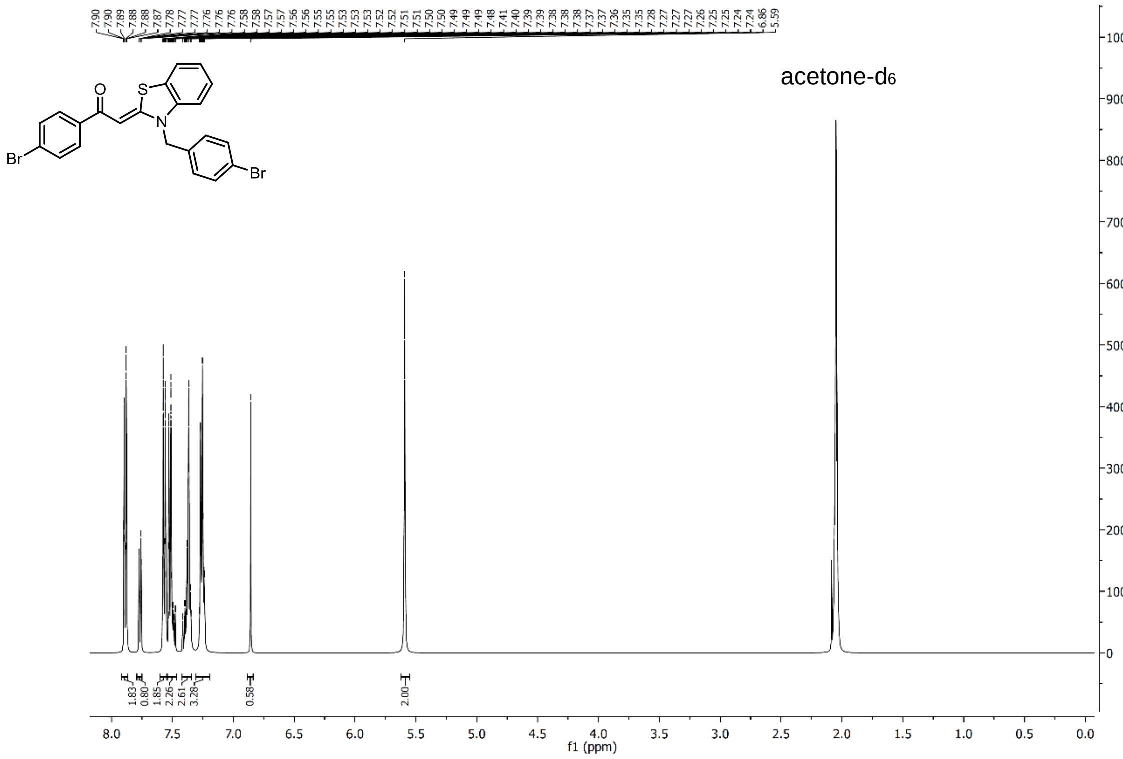

^{13}C NMR-spektrum of (*Z*)-2-(3-(4-Bromobenzyl)benzo[*d*]thiazol-2(3*H*)-ylidene)-1-(4-bromophenyl)ethan-1-one (3v) (acetone-d_6/CS_2 5:1, 125 MHz, 293 K)

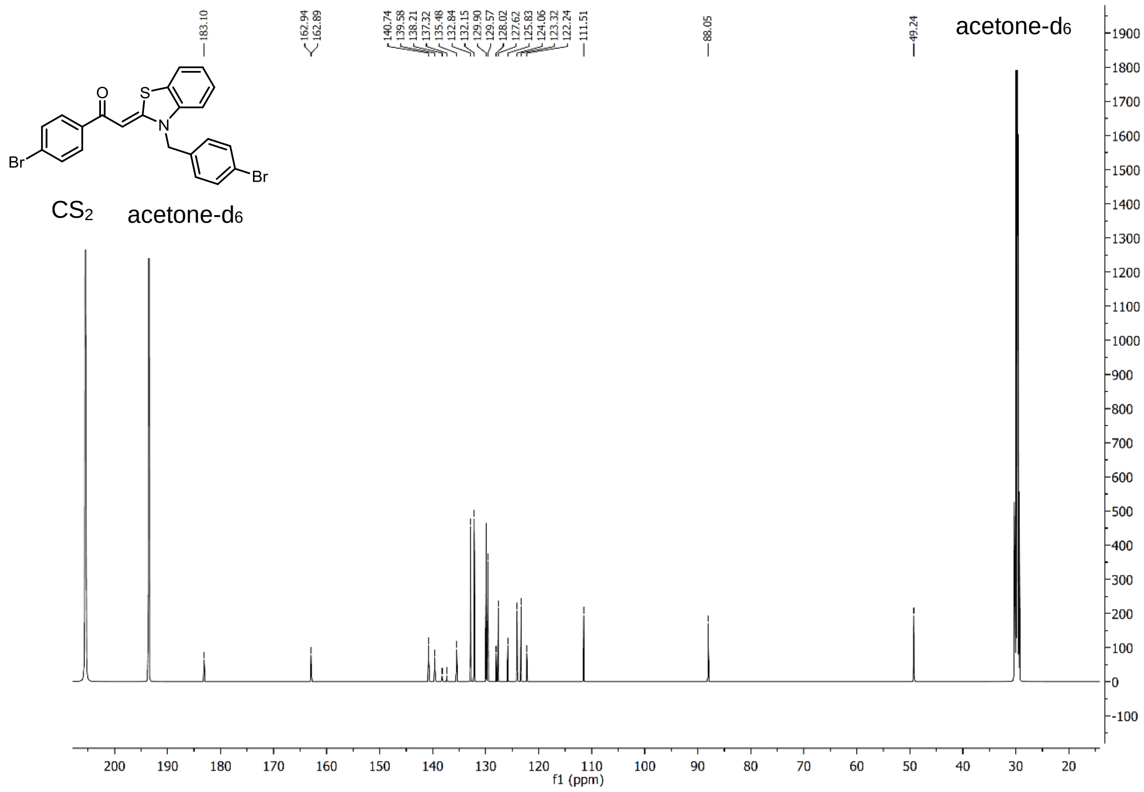

^{1}H NMR-spectrum of (*Z*)-2-(3-Benzyl)benzo[*d*]thiazol-2(3*H*)-ylidene)-1-(4-bromophenyl)-ethan-1-one (3w) (acetone-d_6/CS_2 5:1, 600 MHz, 293 K)

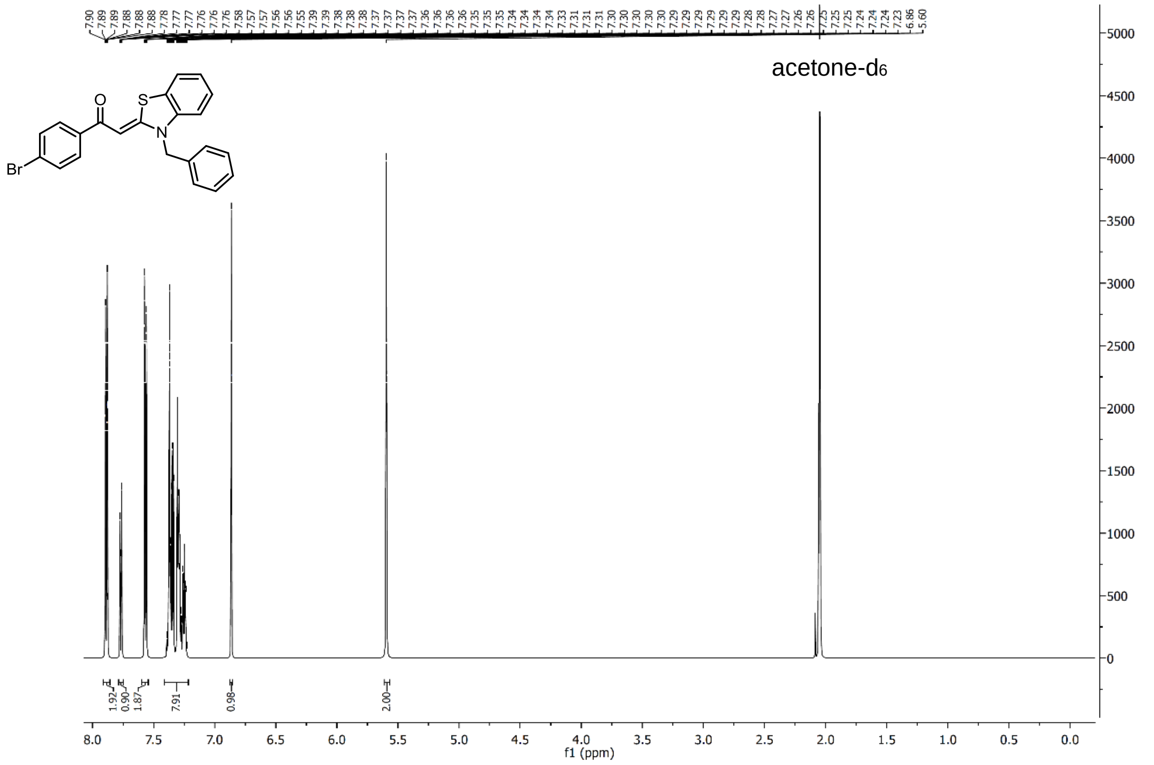

^{13}C NMR-spectrum of (*Z*)-2-(3-Benzyl)benzo[*d*]thiazol-2(3*H*)-ylidene)-1-(4-bromophenyl)-ethan-1-one (3w) (acetone-d_6/CS_2 5:1, 150 MHz, 293 K)

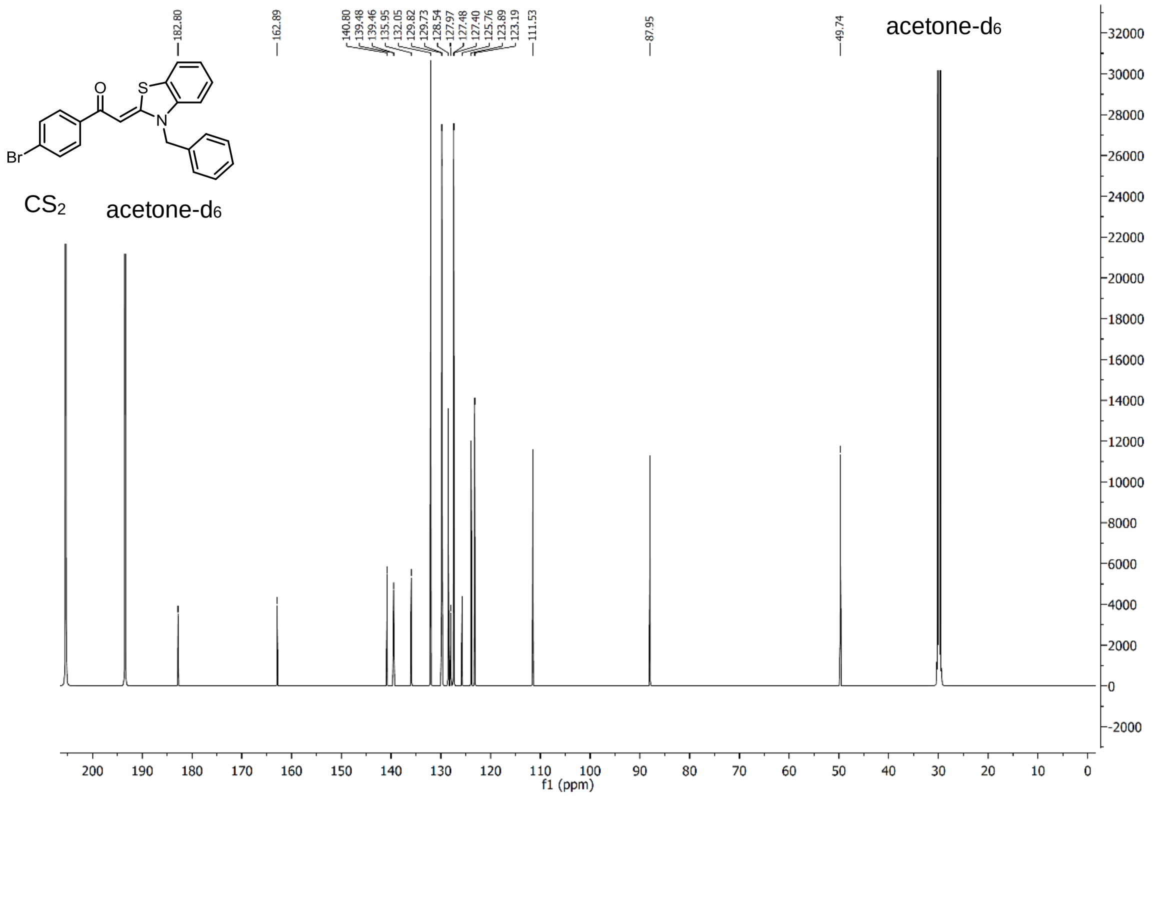

^{1}H NMR-spectrum of (*Z*)-2-(3-(4-Bromobenzyl)benzo[*d*]thiazol-2(3*H*)-ylidene)-1-(4-iodophenyl)ethan-1-one (3x) (acetone-d_6/CS_2 5:1, 600 MHz, 293 K)

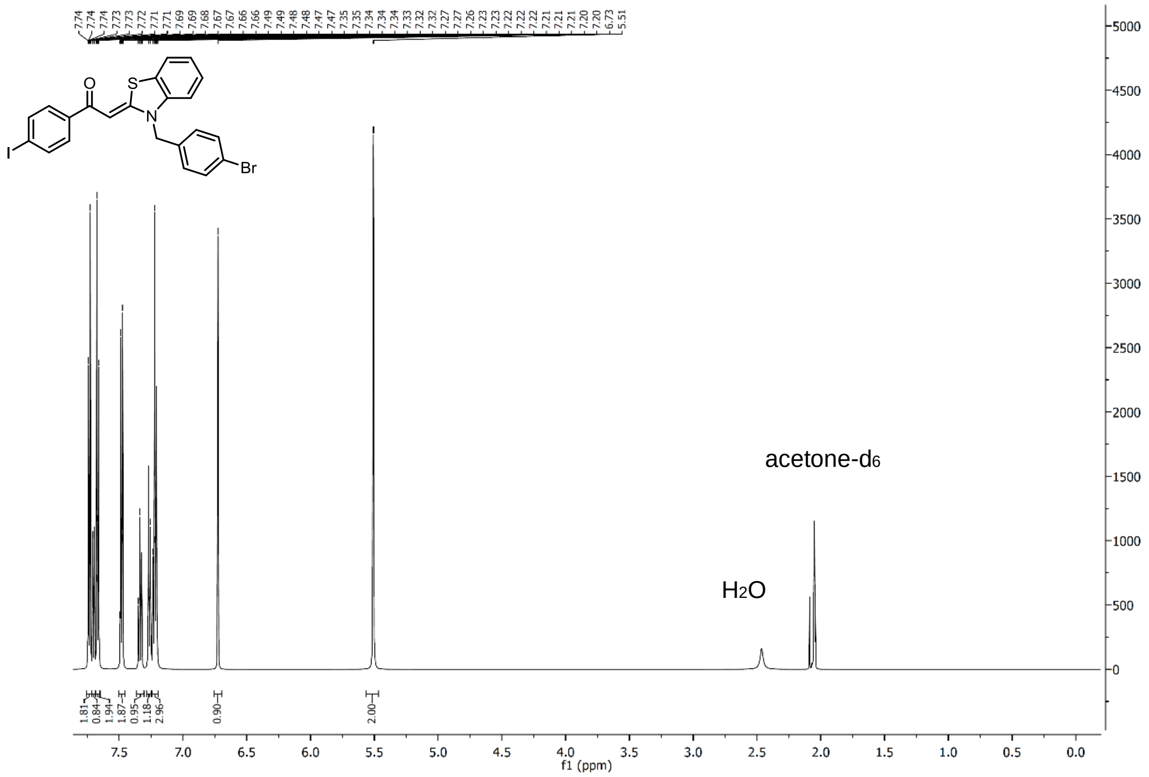

^{13}C NMR-spectrum of (*Z*)-2-(3-(4-Bromobenzyl)benzo[*d*]thiazol-2(3*H*)-ylidene)-1-(4-iodophenyl)ethan-1-one (3x) (acetone-d_6/CS_2 5:1, 150 MHz, 293 K)

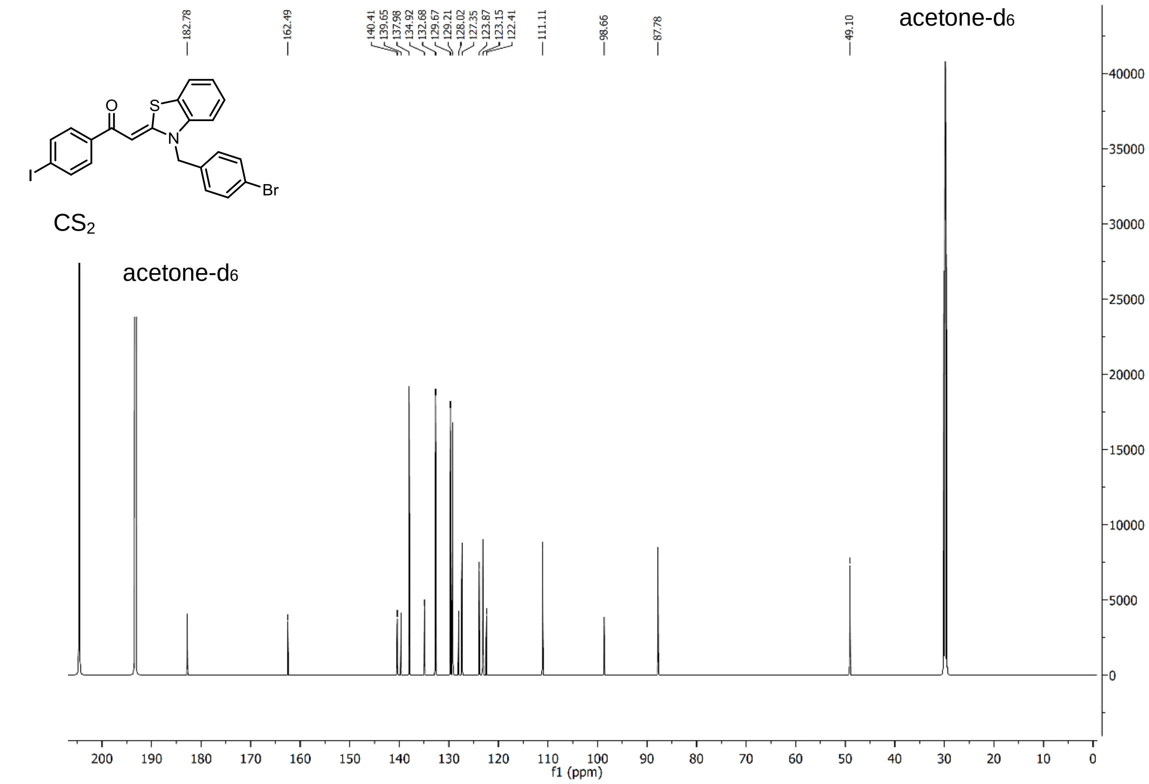

^{1}H NMR-spectrum of (*Z*)-2-(3-Benzyl)benzo[*d*]thiazol-2(3*H*)-ylidene)-1-(4-iodophenyl)-ethan-1-one (3y) (acetone-d_6/CS_2 5:1, 600 MHz, 293 K)

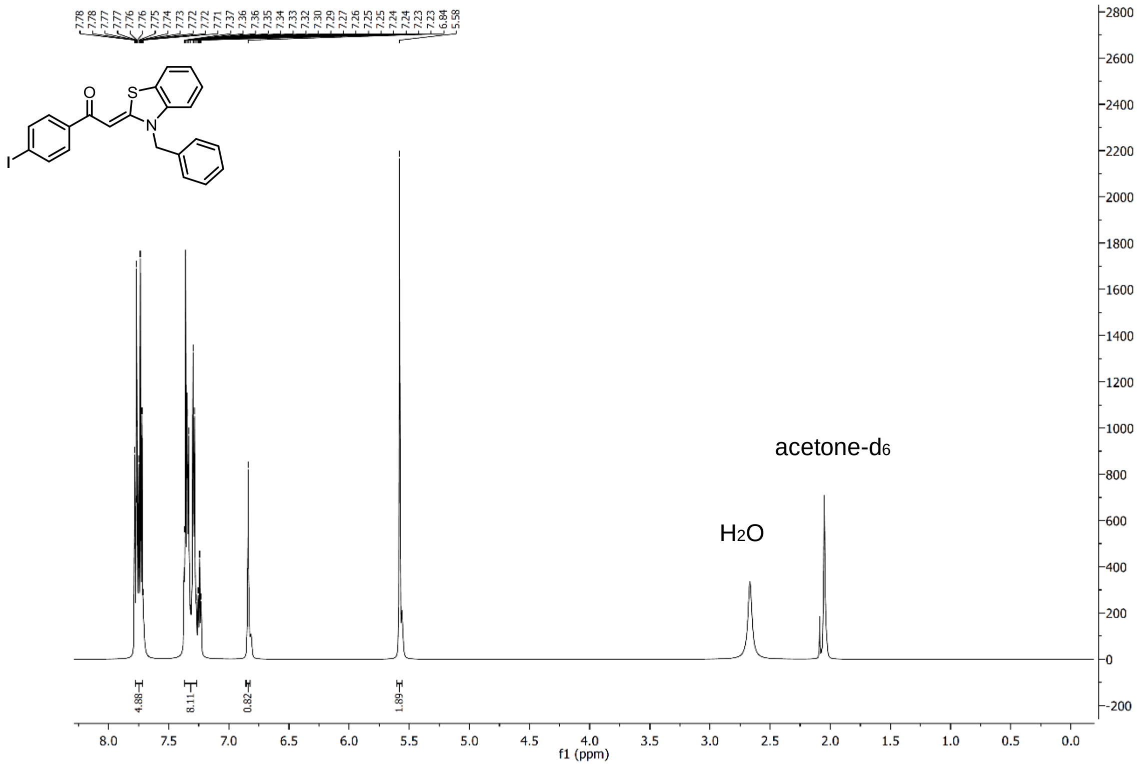

^{13}C NMR-spectrum of (*Z*)-2-(3-Benzyl)benzo[*d*]thiazol-2(3*H*)-ylidene)-1-(4-iodophenyl)-ethan-1-one (3y) (acetone-d_6/CS_2 5:1, 150 MHz, 293 K)

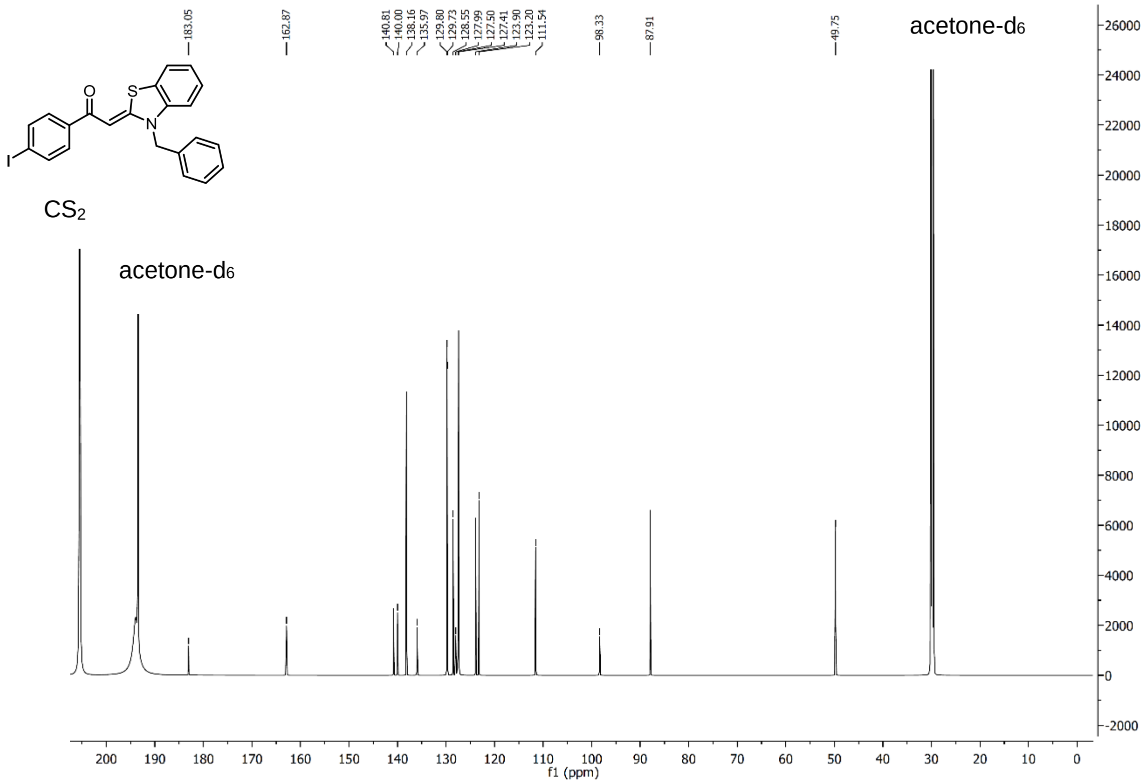

^{1}H NMR-spectrum of (*Z*)-2-(3-(4-Bromobenzyl)benzo[*d*]thiazol-2(3*H*)-ylidene)-1-(4-trifluoromethylphenyl)ethan-1-one (3z) (acetone-d_6/CS_2 5:1, 600 MHz, 293 K)

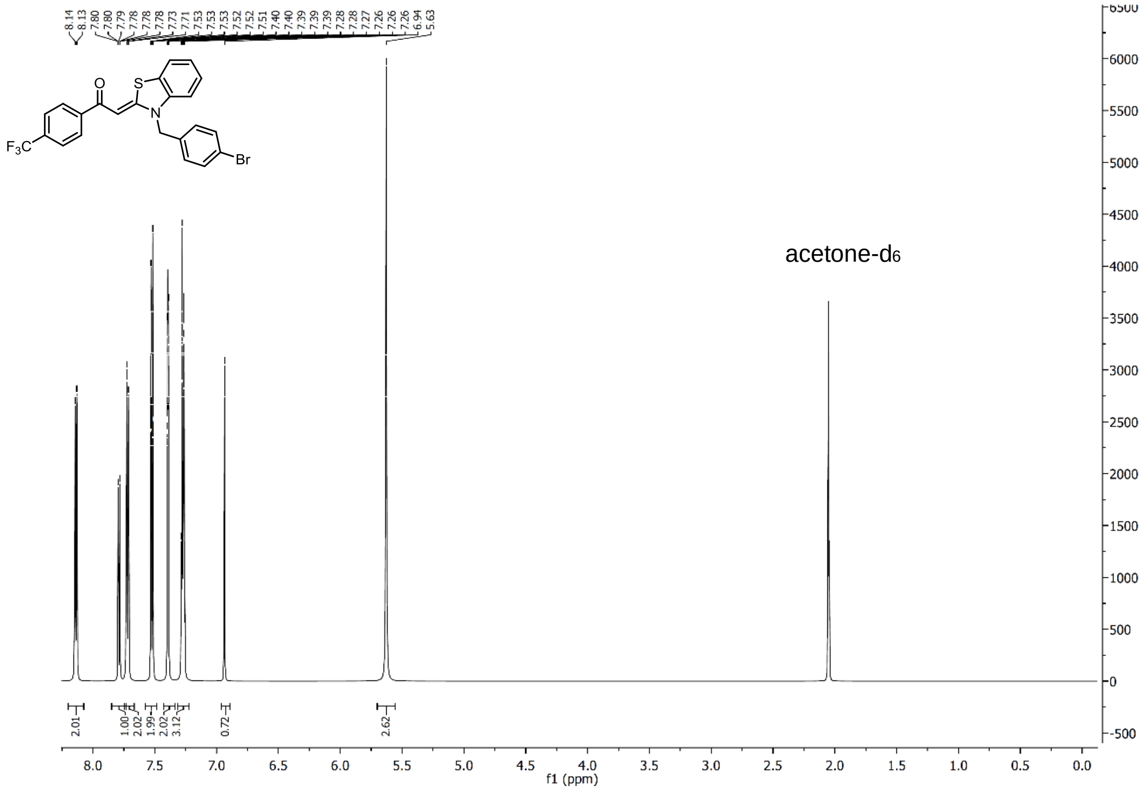

^{13}C NMR-spectrum of (*Z*)-2-(3-(4-Bromobenzyl)benzo[*d*]thiazol-2(3*H*)-ylidene)-1-(4-trifluoromethylphenyl)ethan-1-one (3z) (acetone-d_6/CS_2 5:1, 150 MHz, 293 K)

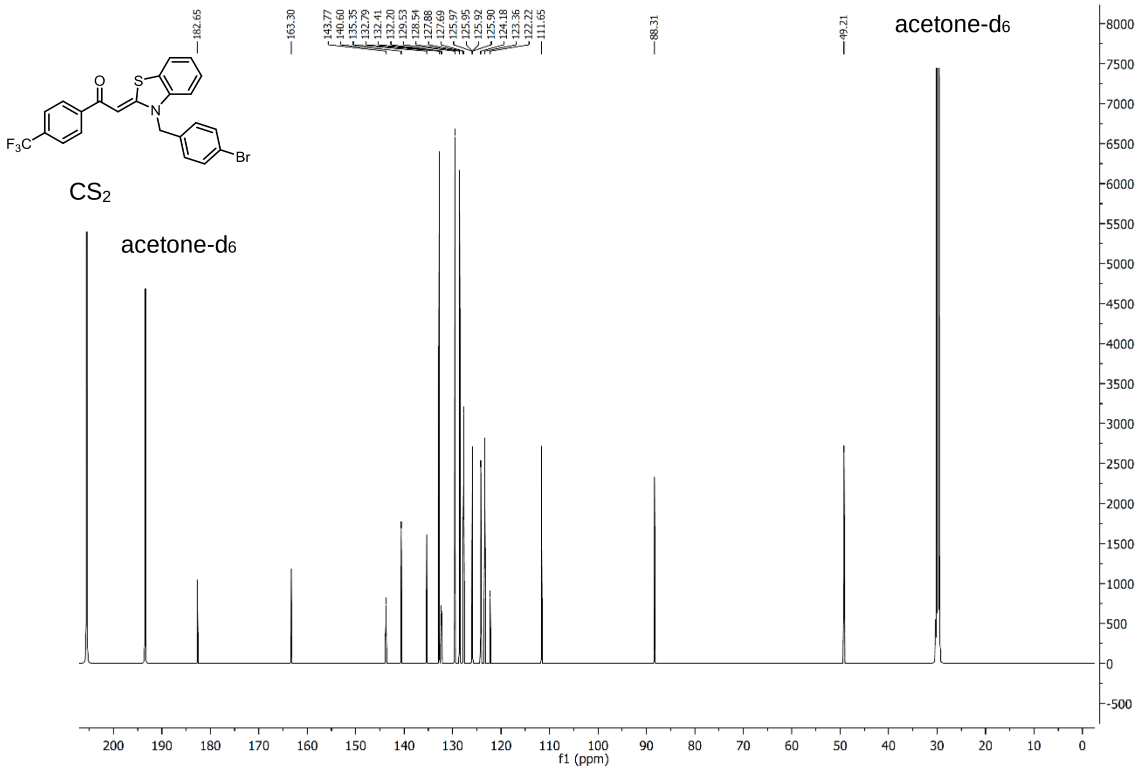

^{1}H NMR-spectrum of (*Z*)-2-(3-Benzyl)benzo[*d*]thiazol-2(3*H*)-ylidene)-1-(4-trifluoromethyl-phenyl)ethan-1-one (3aa) (acetone-d_6/CS_2 5:1, 500 MHz, 293 K)

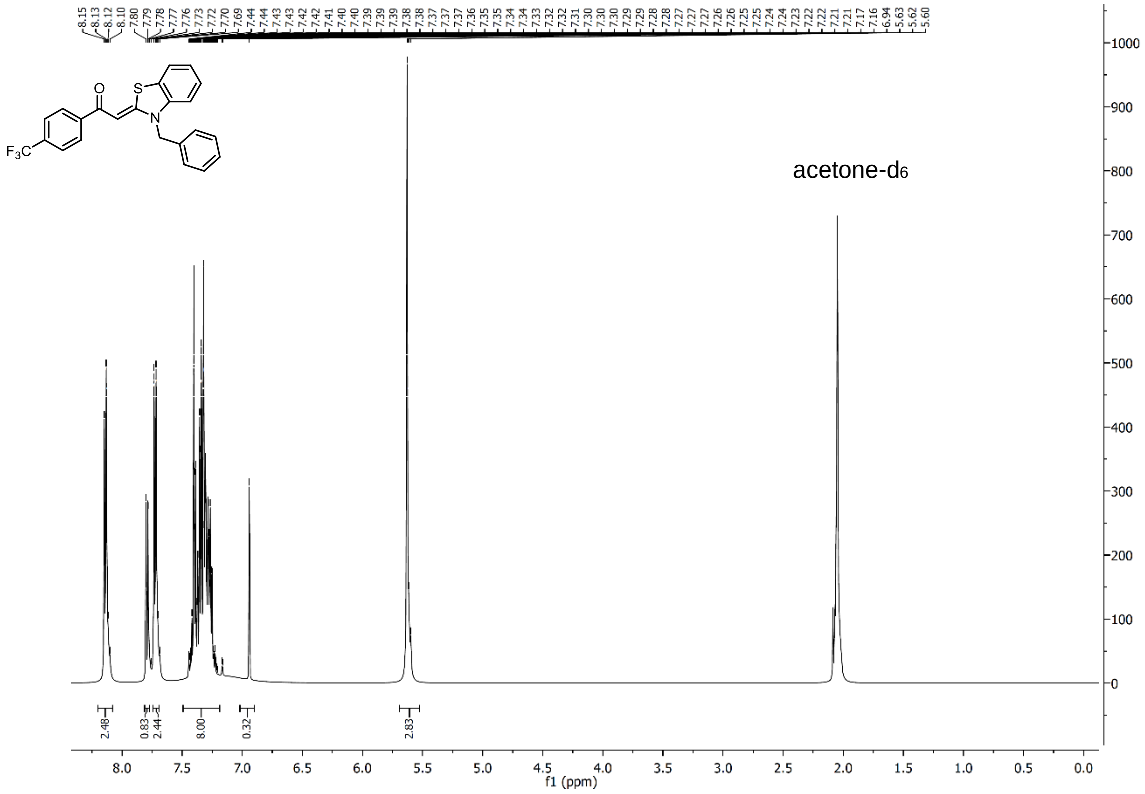

^{13}C NMR-spectrum of (*Z*)-2-(3-Benzyl)benzo[*d*]thiazol-2(3*H*)-ylidene)-1-(4-trifluoromethylphenyl)ethan-1-one (3aa) (acetone-d_6/CS_2 5:1, 125 MHz, 293 K)

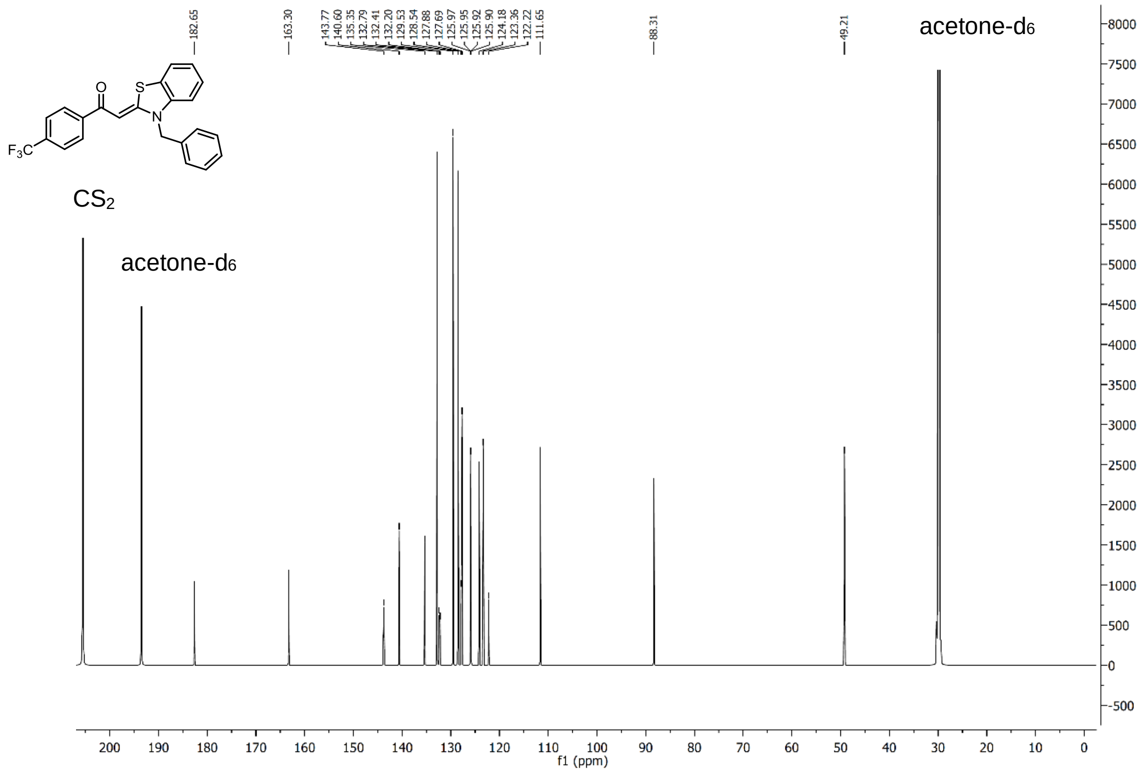

^{1}H NMR-spectrum of (*Z*)-4-(2-(3-(4-Bromobenzyl)benzo[*d*]thiazol-2(3*H*)-ylidene)acetyl)benzonitrile (3ab) (dmso-d_6, 300 MHz, 293 K)

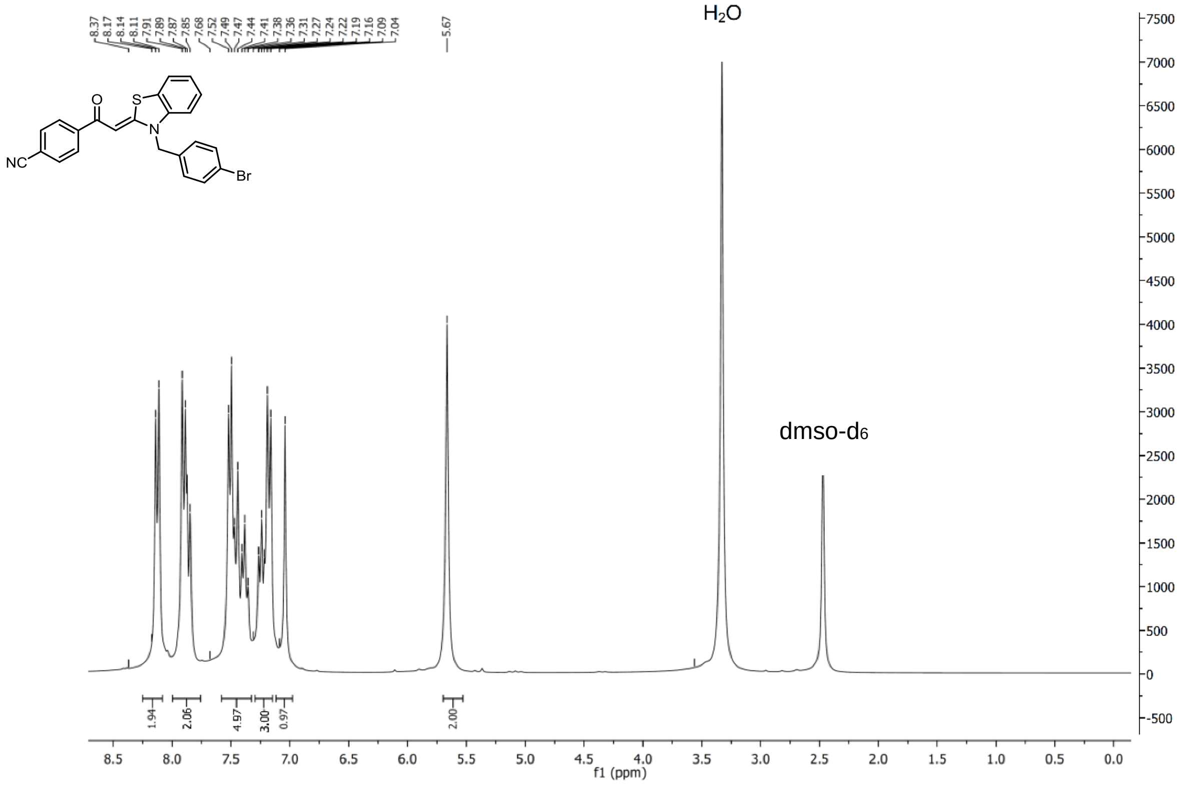

^{13}C NMR-spectrum of (*Z*)-4-(2-(3-(4-Bromobenzyl)benzo[*d*]thiazol-2(3*H*)-ylidene)acetyl)-benzonitrile (3ab) (dmso-d_6, 75 MHz, 293 K)

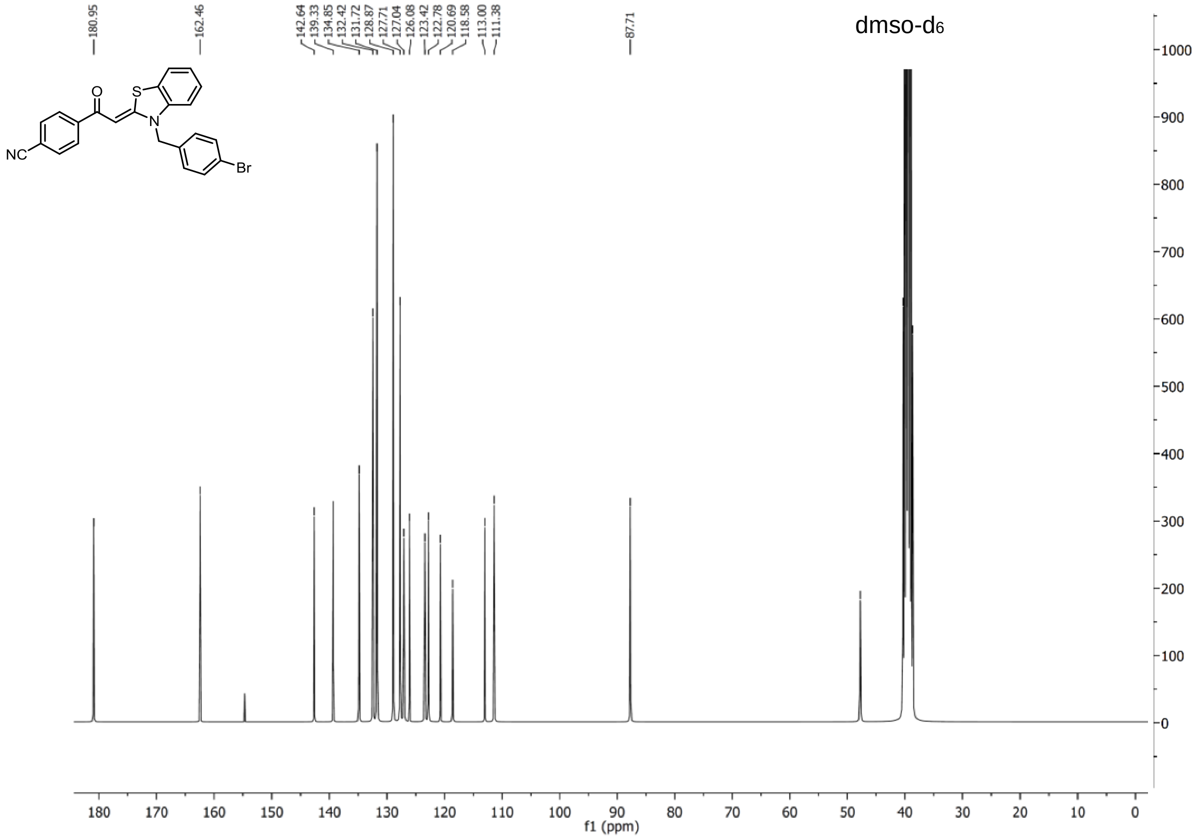

^{1}H NMR-spectrum of (*Z*)-4-(2-(3-Benzyl)benzo[*d*]thiazol-2(3*H*)-ylidene)acetyl)-benzonitrile (3ac) (acetone-d_6/CS_2 5:1, 300 MHz, 293 K)

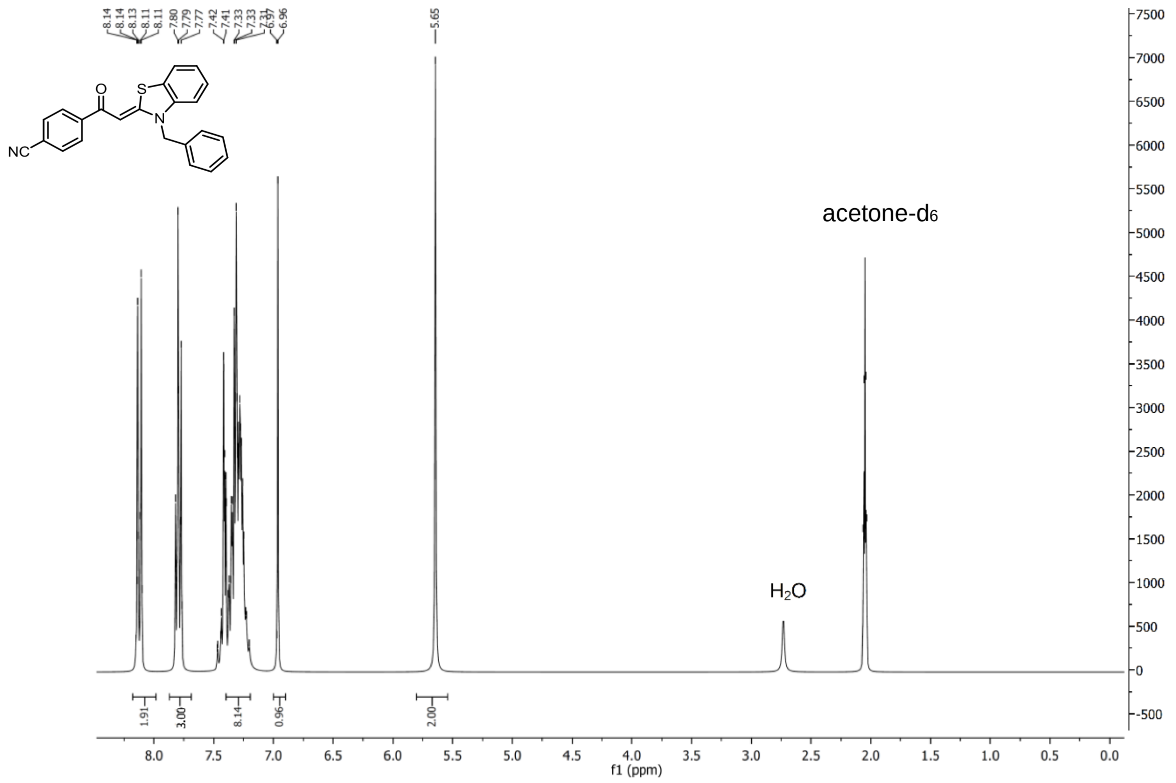

^{13}C NMR-spectrum of (*Z*)-4-(2-(3-Benzyl)benzo[*d*]thiazol-2(3*H*)-ylidene)acetyl)-benzonitrile (3ac) (acetone-d_6/CS_2 5:1, 75 MHz, 293 K)

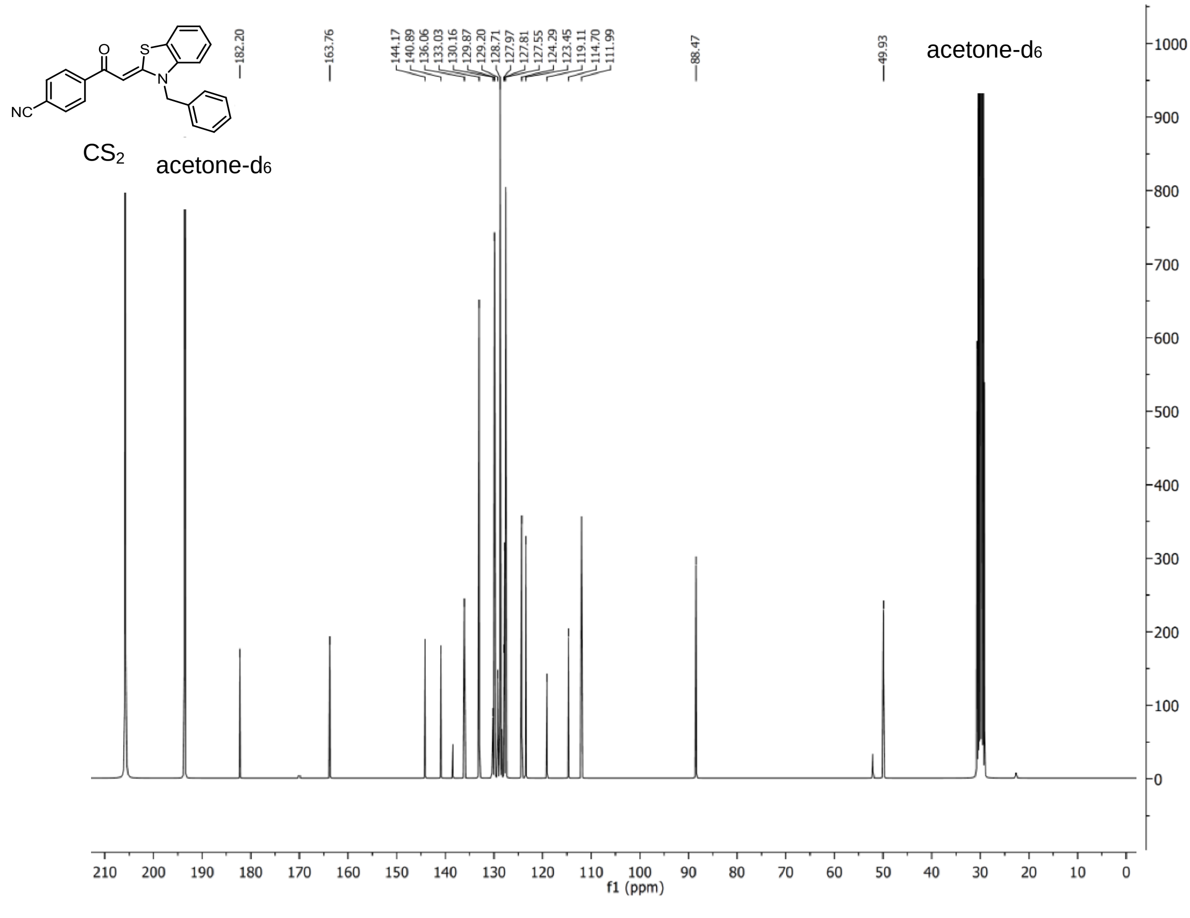

^{1}H NMR-spectrum of (*Z*)-2-(3-(4-Bromobenzyl)benzo[*d*]thiazol-2(3*H*)-ylidene)-1-(4-nitrophenyl)ethan-1-one (3ad) (dmso-d_6, 500 MHz, 293 K)

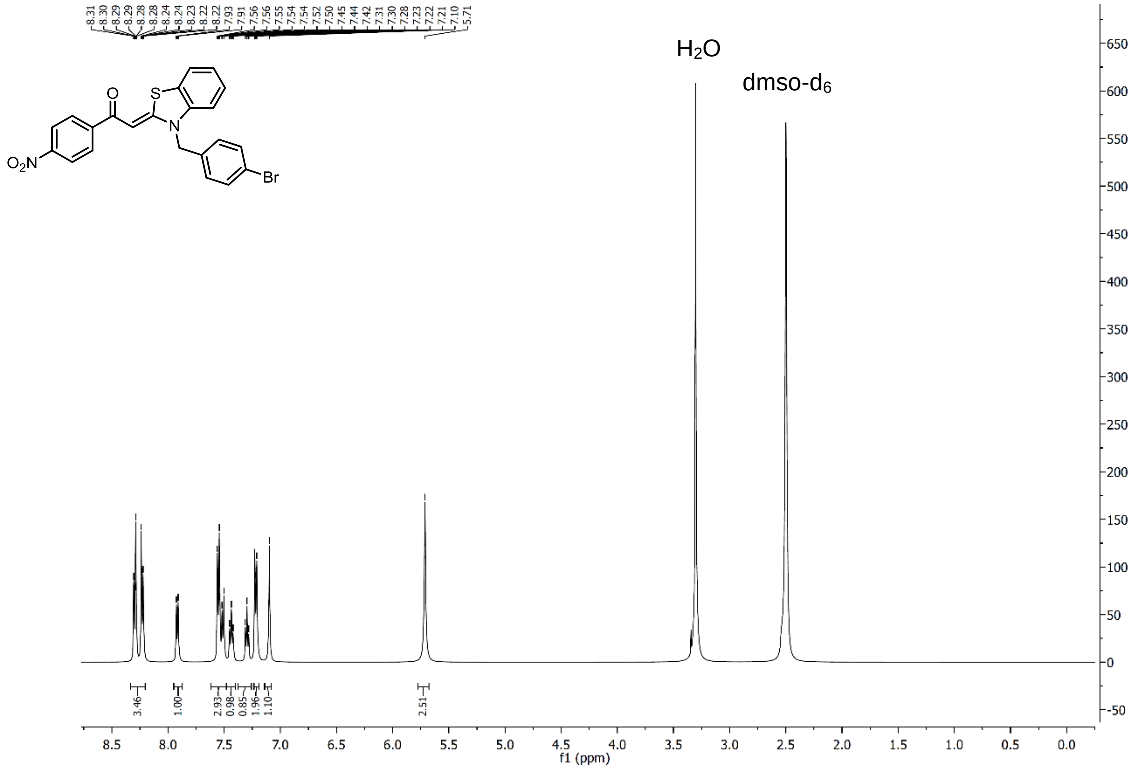

^{13}C NMR-spectrum of (*Z*)-2-(3-(4-Bromobenzyl)benzo[*d*]thiazol-2(3*H*)-ylidene)-1-(4-nitrophenyl)ethan-1-one (3ad) (dmso-d_6, 125 MHz, 293 K)

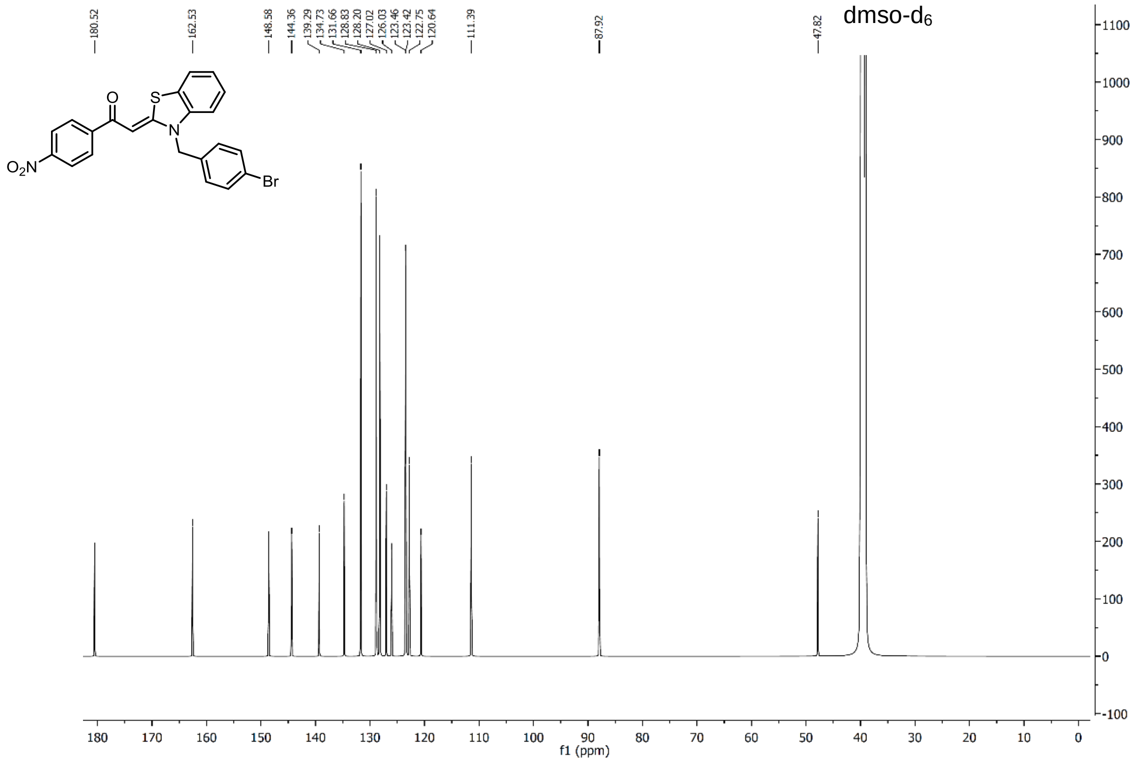

^{1}H NMR-spectrum von (*Z*)-2-(3-Benzyl)benzo[*d*]thiazol-2(3*H*)-ylidene)-1-(4-nitrophenyl)-ethan-1-one (3ae) (dmso-d_6, 600 MHz, 293 K)

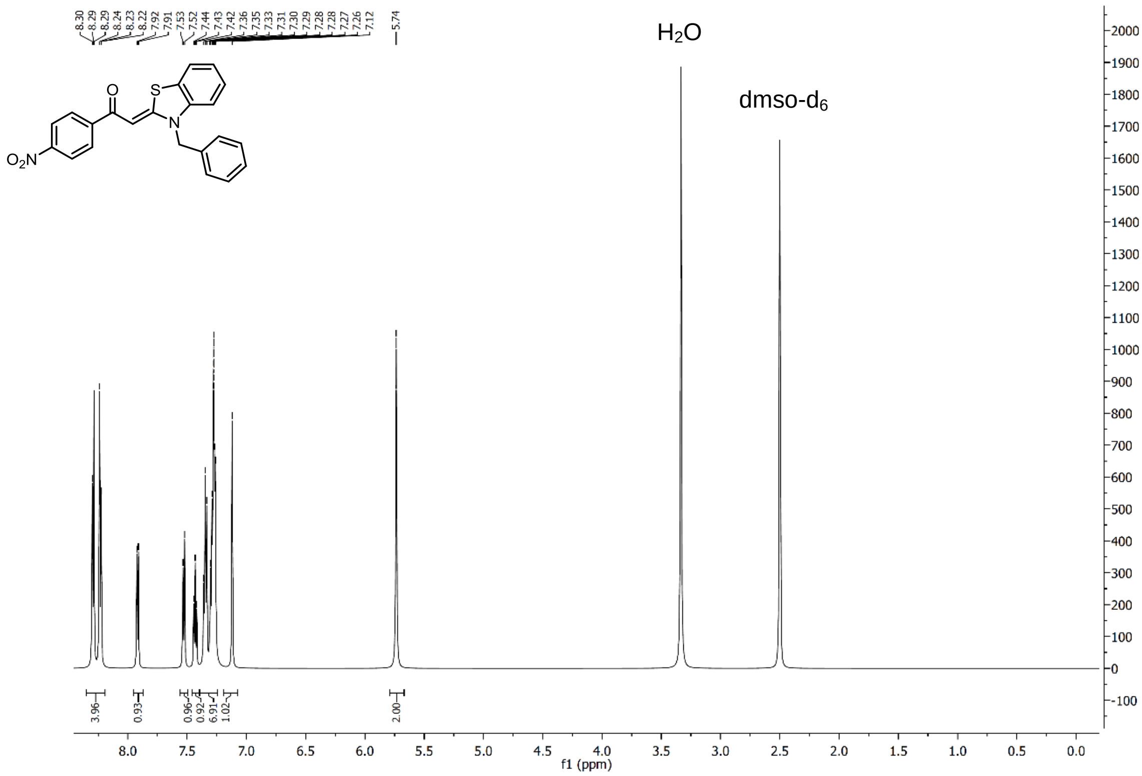

^{13}C NMR-spectrum of (*Z*)-2-(3-Benzyl)benzo[*d*]thiazol-2(3*H*)-ylidene)-1-(4-nitrophenyl)-ethan-1-one (3ae) (dmso-d_6, 150 MHz, 293 K)

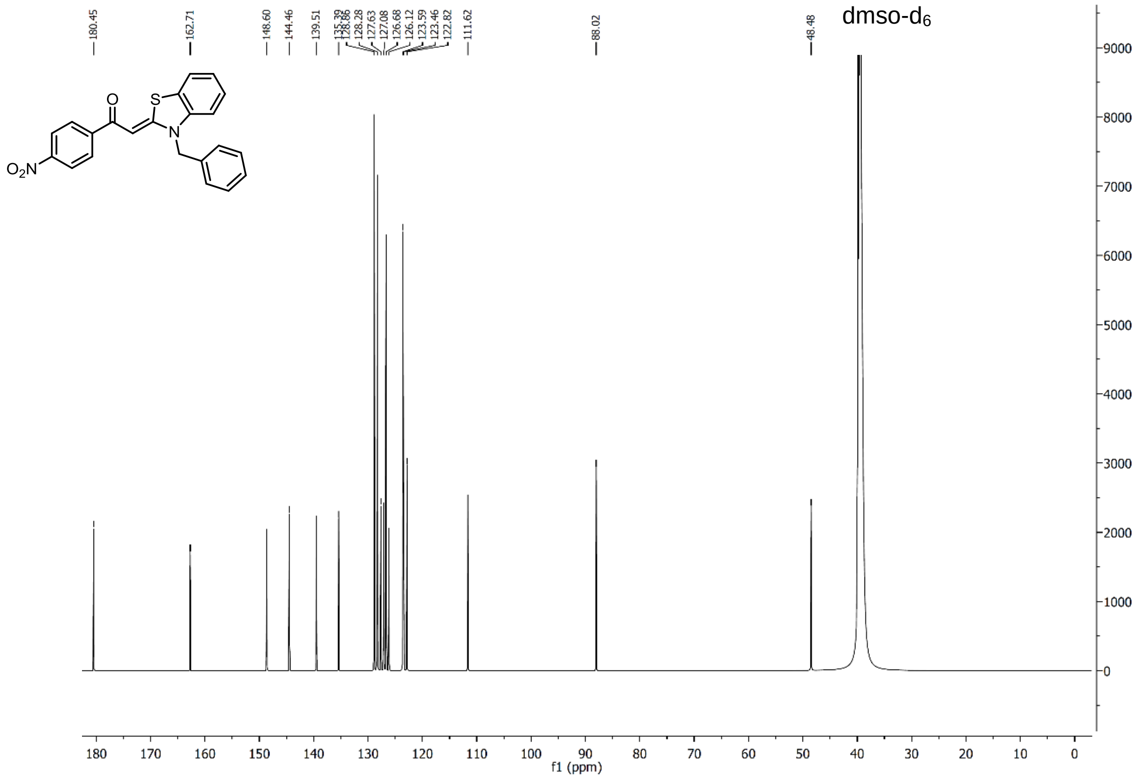

^{1}H NMR-spectrum of (*Z*)-2-(3-(4-Bromobenzyl)benzo[*d*]thiazol-2(3*H*)-ylidene)-1-(thiophen-2-yl)ethan-1-one (3af) (acetone-d_6/CS_2 5:1, 600 MHz, 293 K)

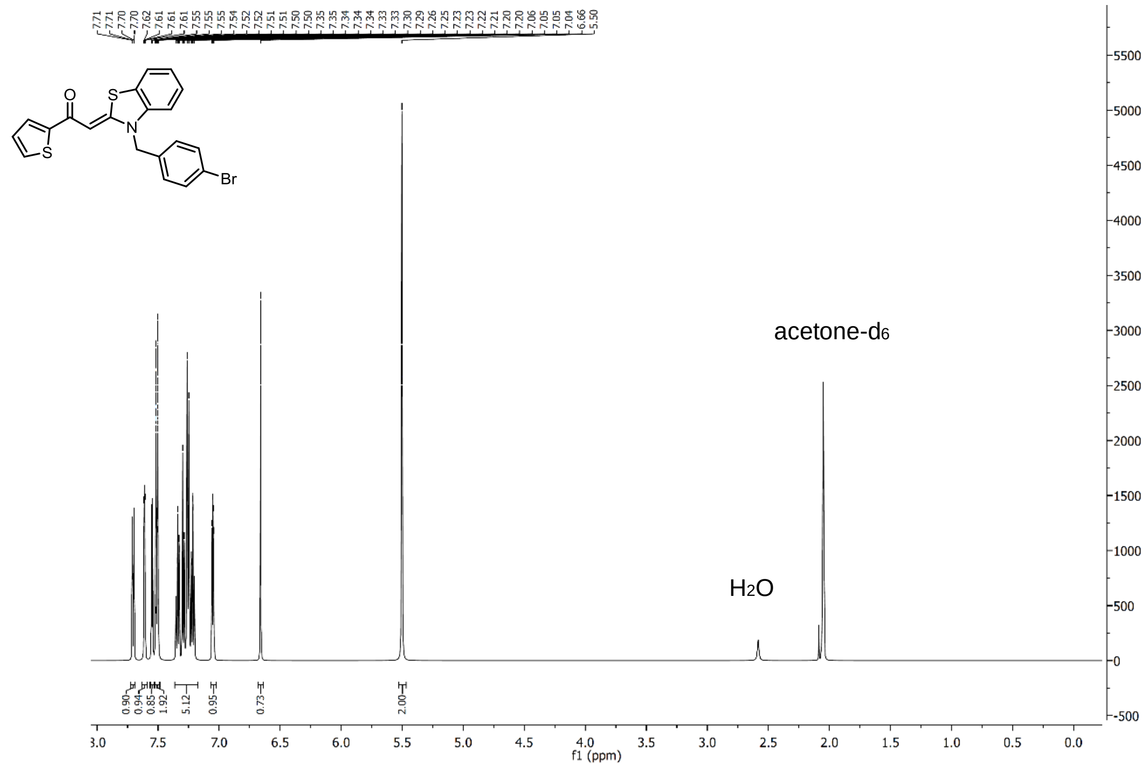

^{13}C NMR-spectrum of (*Z*)-2-(3-(4-Bromobenzyl)benzo[*d*]thiazol-2(3*H*)-ylidene)-1-(thiophen-2-yl)ethan-1-one (3af) (acetone-d_6/CS_2 5:1, 150 MHz, 293 K)

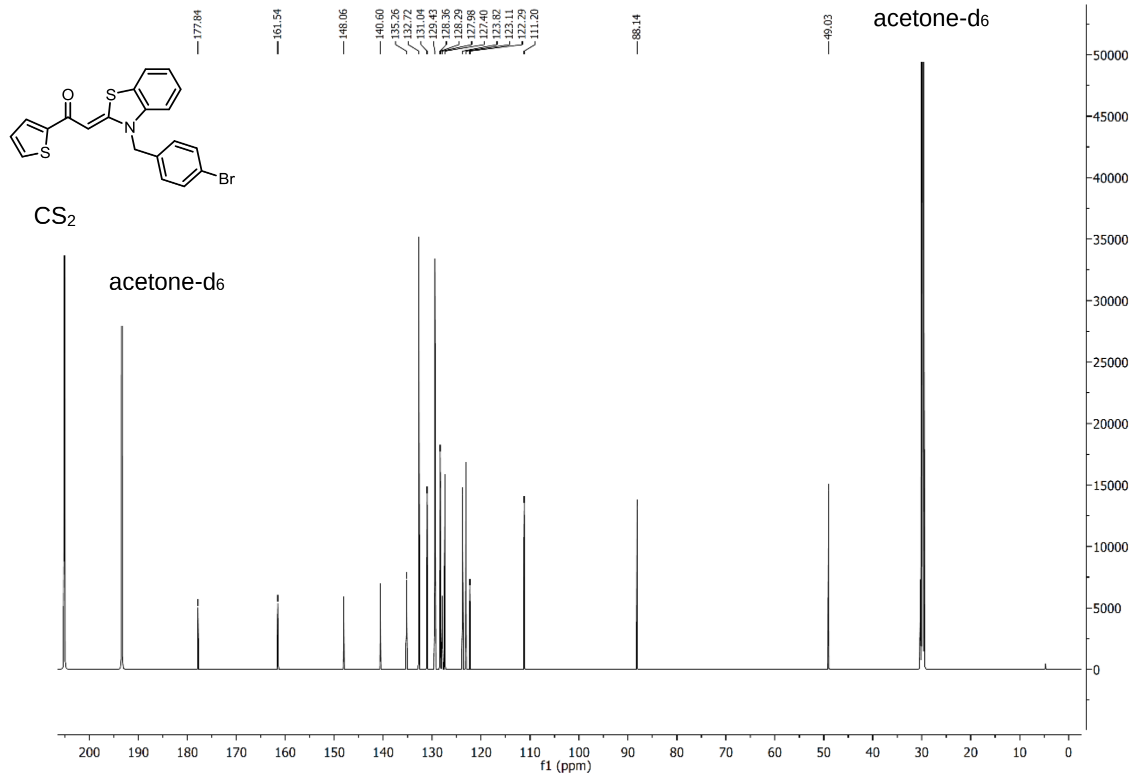

^{1}H NMR-spectrum of ((*Z*)-2-(3-Benzyl)benzo[*d*]thiazol-2(3*H*)-ylidene)-1-(thiophen-2-yl)ethan-1-one (3ag) (acetone-d_6/CS_2 5:1, 600 MHz, 293 K)

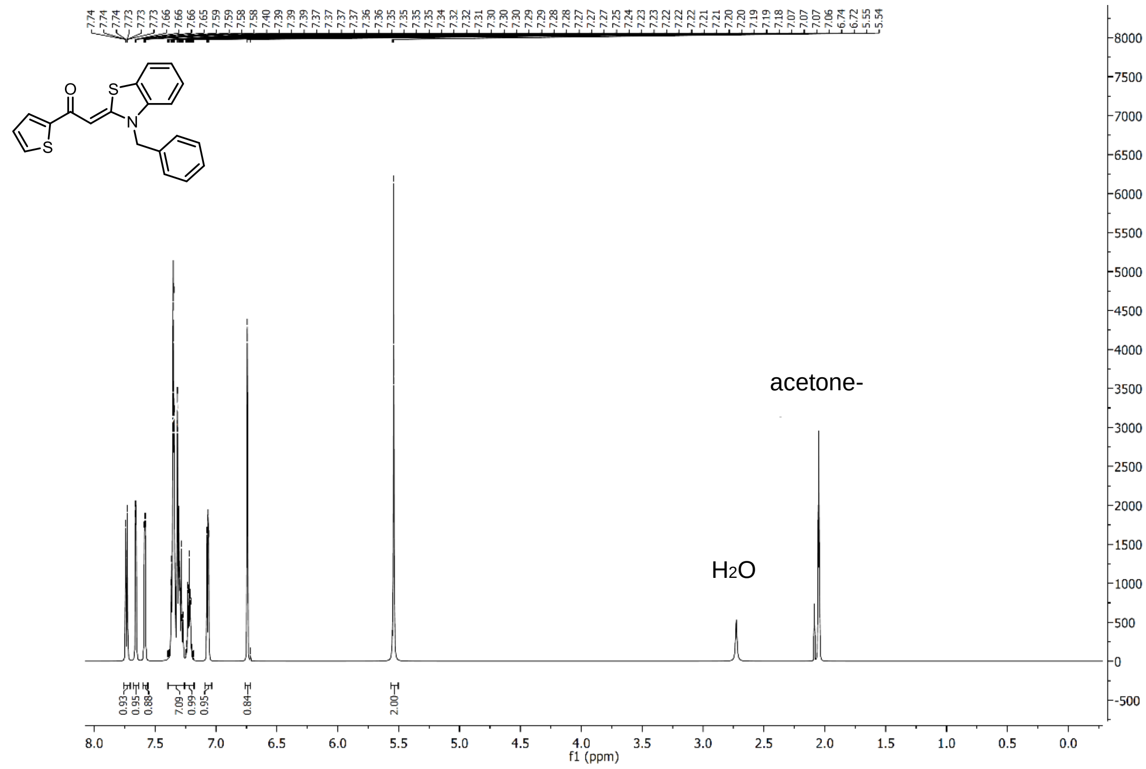

^{13}C NMR-spectrum of ((*Z*)-2-(3-Benzyl)benzo[*d*]thiazol-2(3*H*)-ylidene)-1-(thiophen-2-yl)ethan-1-one (3ag) (acetone-d_6/CS_2 5:1, 150 MHz, 293 K)

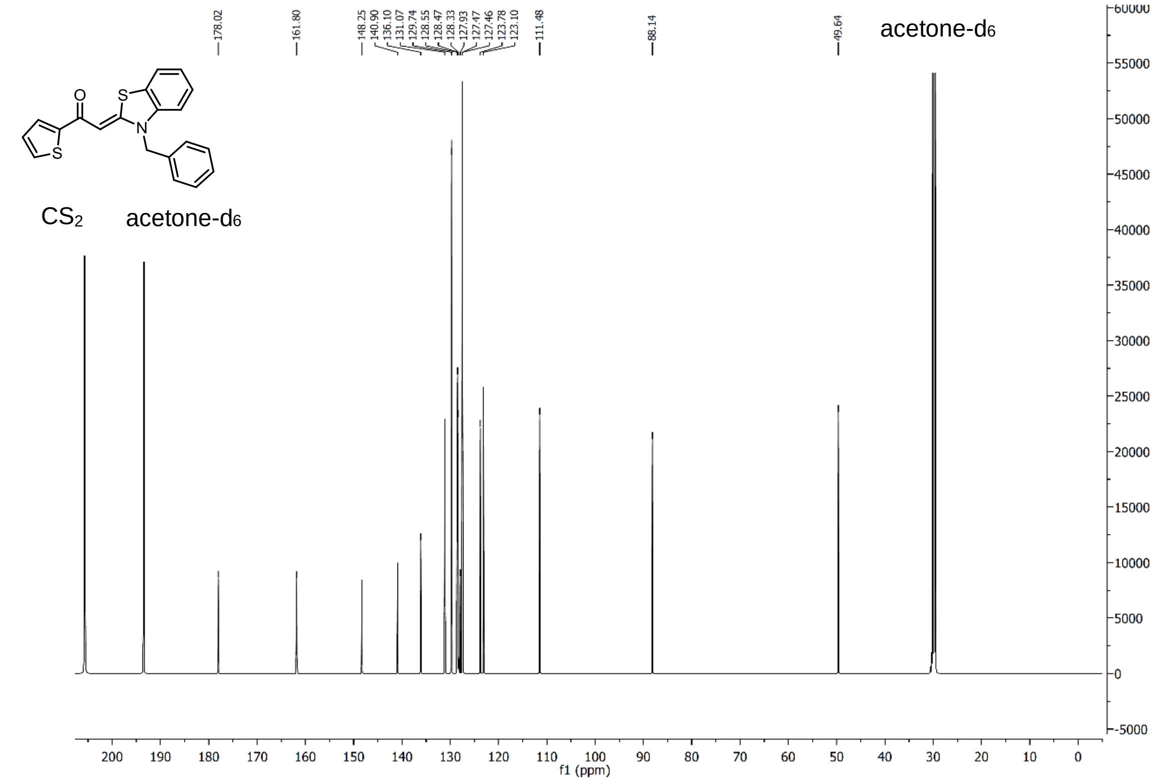

^{1}H NMR-spectrum of ((*Z*)-2-(3-(4-Bromobenzyl)benzo[*d*]thiazol-2(3*H*)-ylidene)-1-(furan-2-yl)ethan-1-one (3ah) (acetone-d_6/CS_2 5:1, 600 MHz, 293 K)

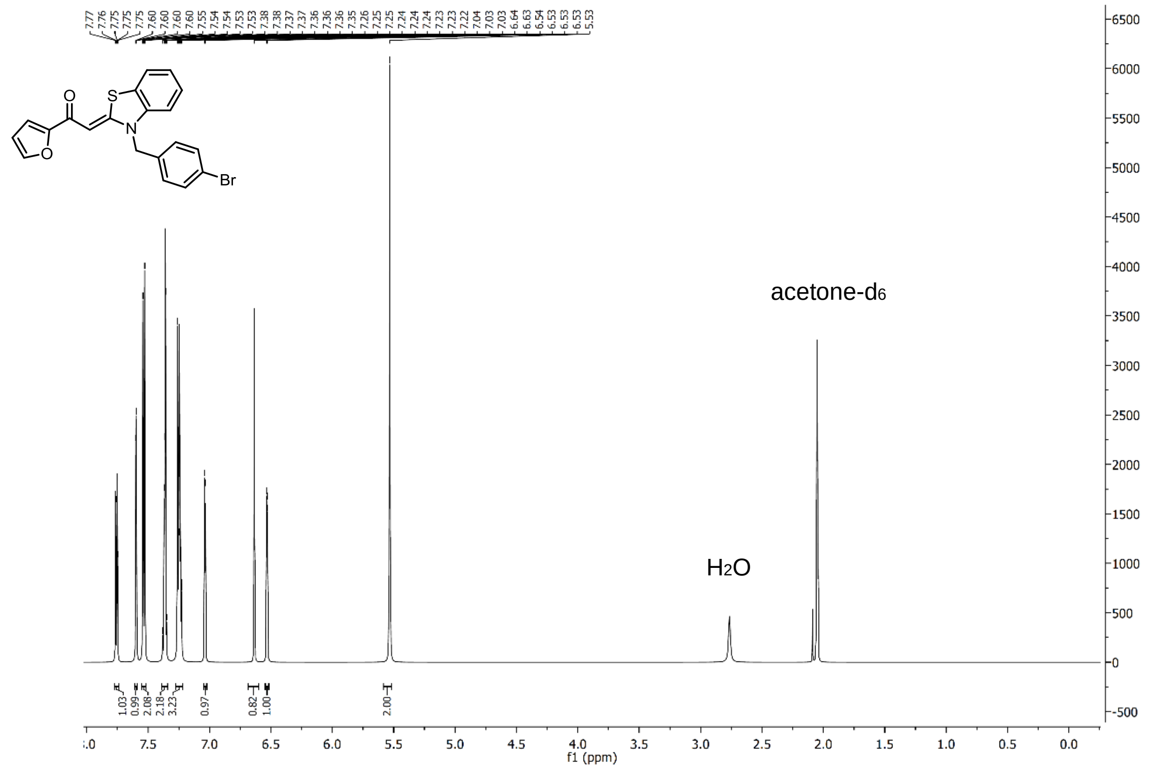

^{13}C NMR-spectrum of ((*Z*)-2-(3-(4-Bromobenzyl)benzo[*d*]thiazol-2(3*H*)-ylidene)-1-(furan-2-yl)ethan-1-one (3ah) (acetone-d_6/CS_2 5:1, 150 MHz, 293 K)

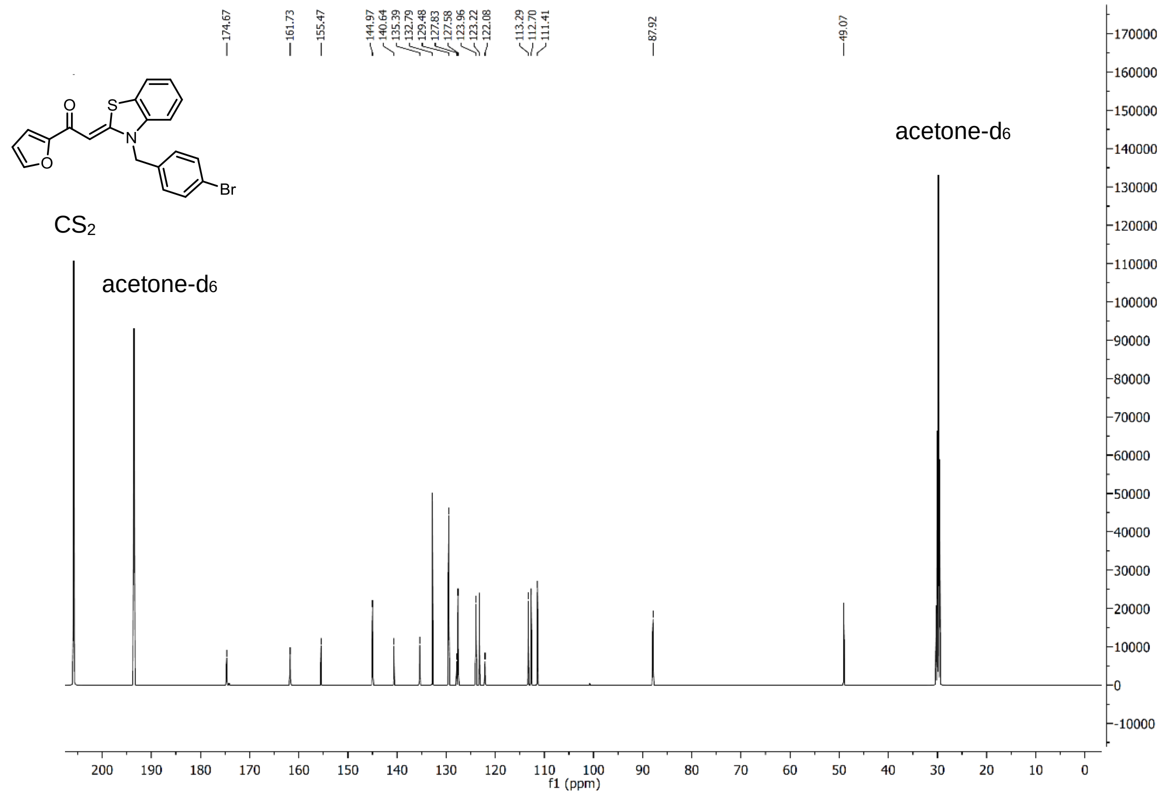

^{1}H NMR-spectrum of ((*Z*)-2-(3-Benzyl)benzo[*d*]thiazol-2(3*H*)-ylidene)-1-(furan-2-yl)ethan-1-one (3ai) (acetone-d_6/CS_2 5:1, 600 MHz, 293 K)

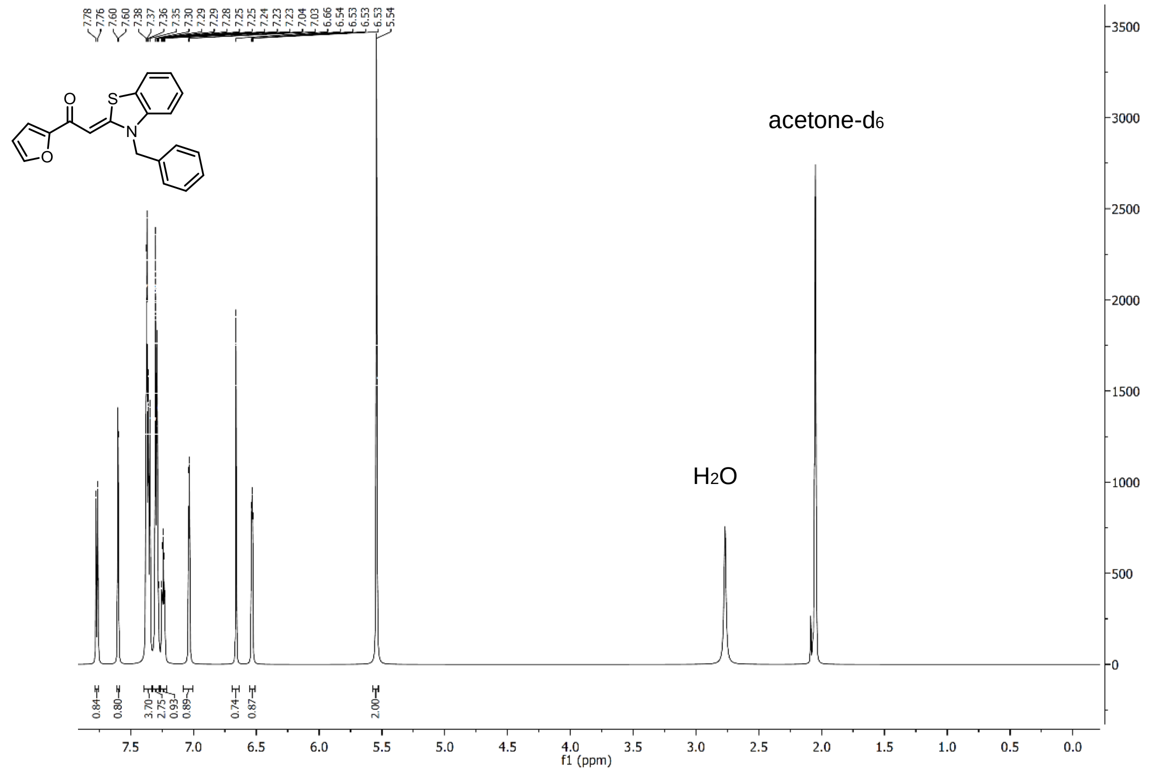

^{13}C NMR-spectrum of ((*Z*)-2-(3-Benzyl)benzo[*d*]thiazol-2(3*H*)-ylidene)-1-(furan-2-yl)ethan-1-one (3ai) (acetone-d_6/CS_2 5:1, 150 MHz, 293 K)

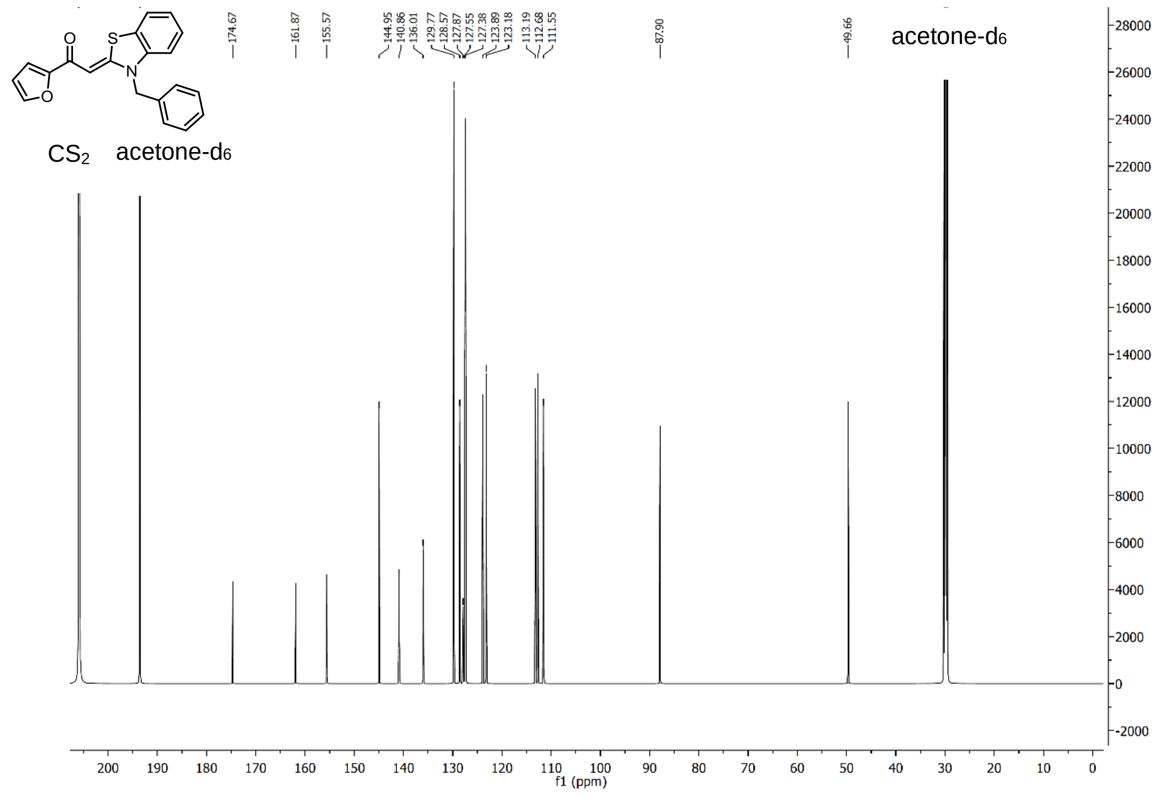

6 Absorption and emission spectra

All solution spectra were recorded in ethanol or in ethanol/water mixtures at T = 298 K, the excitation wavelengths for the AIE-titration studies and the emission spectra in solution for **3a** and **3b** were determined from the absorption maxima of this compound, the excitation wavelength for the solid state emission spectra was determined from solid state excitation spectra. The dye concentration of the solution for absorption measurements was $c = 10^{-5}$ M and the dye concentration of the ethanol/water mixtures for AIE measurements was $c = 10^{-7}$ M.

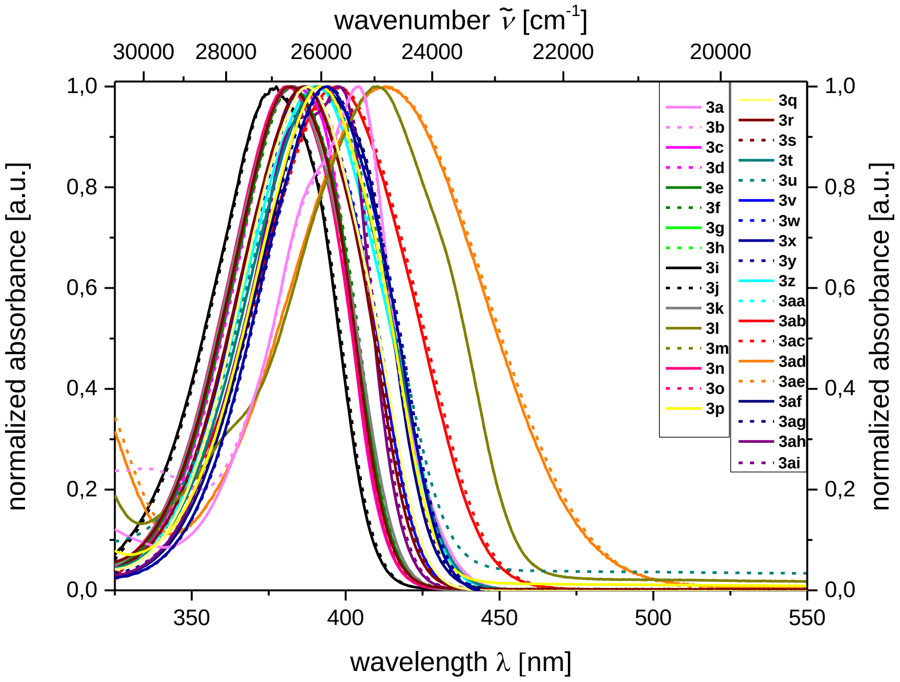

Figure S1: Normalized UV/Vis absorption bands of aroyl-*S,N*-ketene acetals **3**.

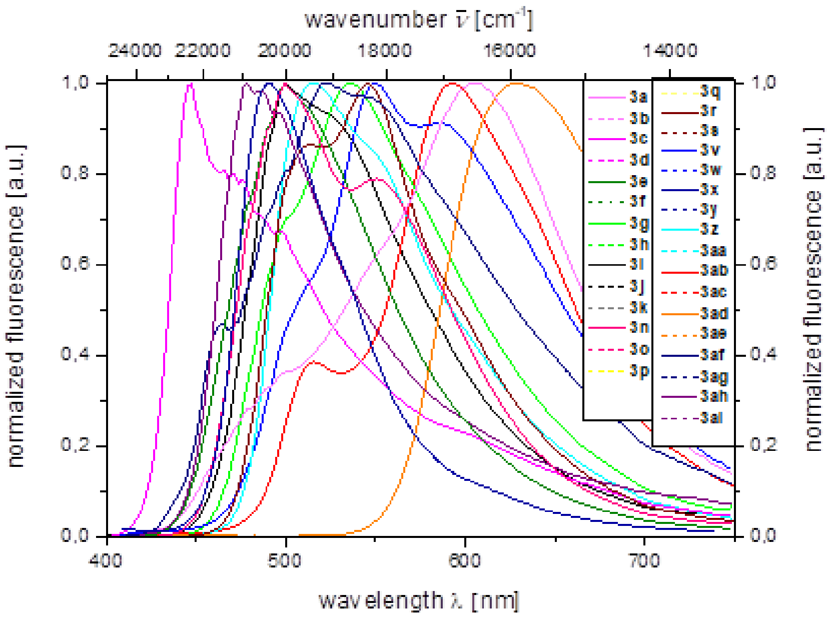

Figure S2: Normalized solid state emission bands of aroyl-*S,N*-ketene acetals **3**.

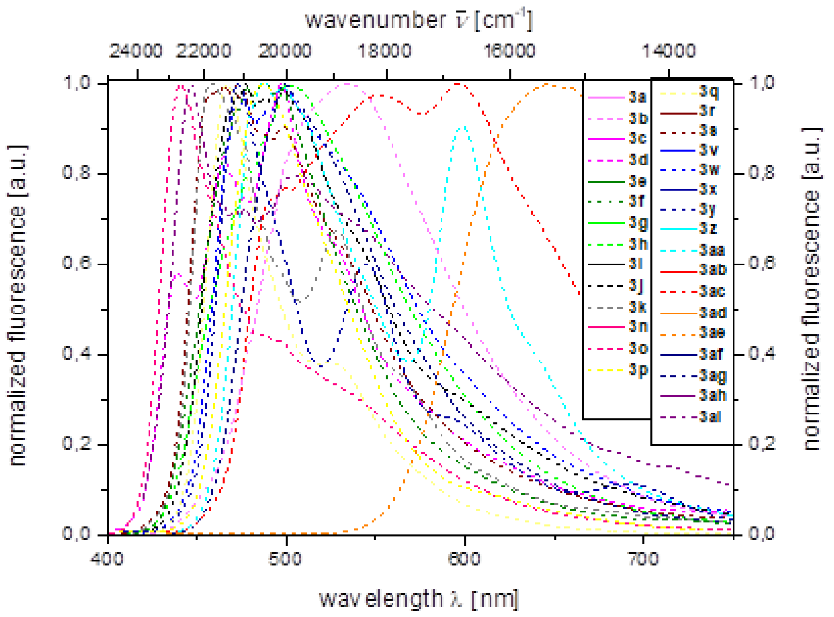

Figure S3: Normalized solid state emission bands of aroyl-*S,N*-ketene acetals **3**.

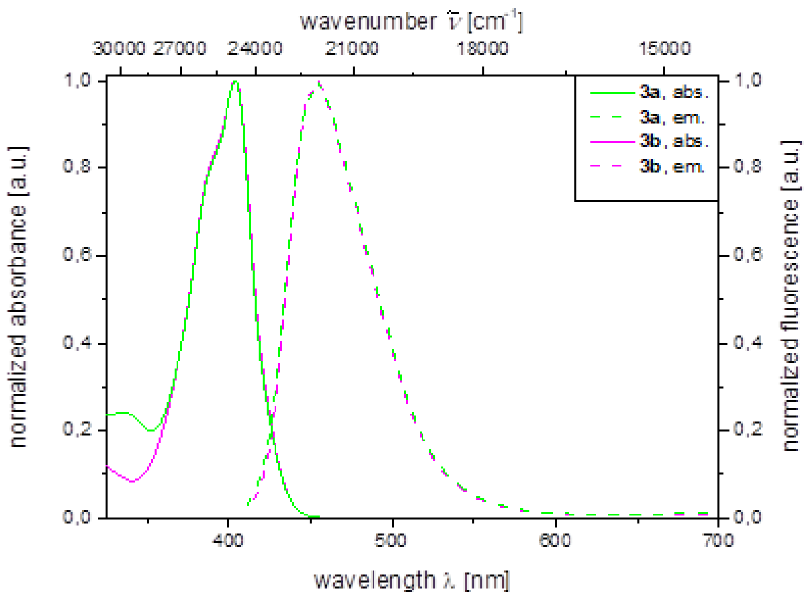

Figure S4: Normalized absorption and emission spectra of **3a** and **3b**, abs.: c = 10^{-5} M, em: c = 10^{-7} M, **3a**: λ_{exc} = 404 nm, **3b**: λ_{exc} = 404 nm.

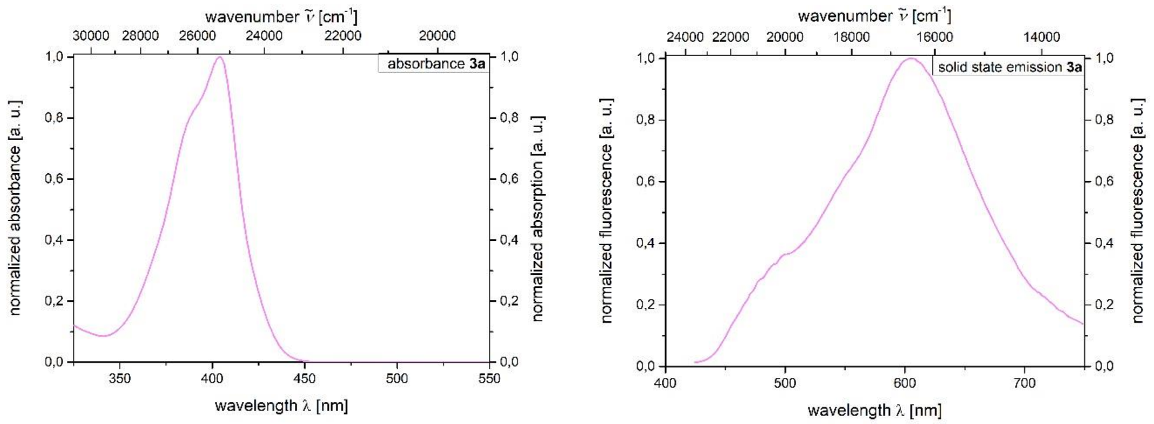

Figure S5: Absorption spectrum of compound **3a** in ethanol and solid state emission spectrum of compound **3a**

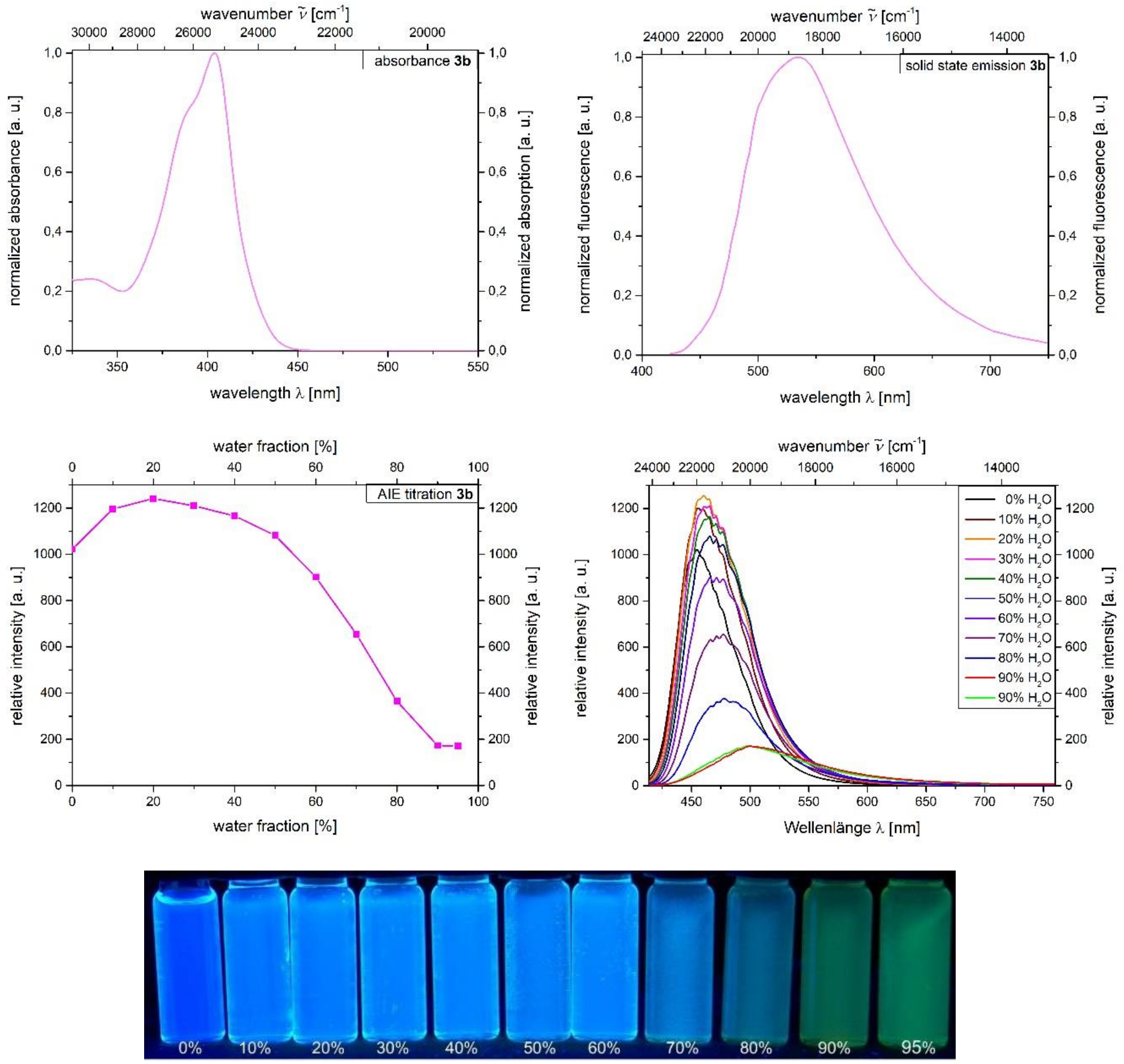

Figure S6: Absorption spectrum of compound **3b** in ethanol (top, left), solid state emission spectrum (top, right), and AIE-induced changes in emission (middle, left), AIE-related emission spectra of compound **3b** and photographs of solutions of dye **3b** in ethanol/water mixtures of increasing water content. The latter spectra were measured in ethanol/water mixtures of varying water content.

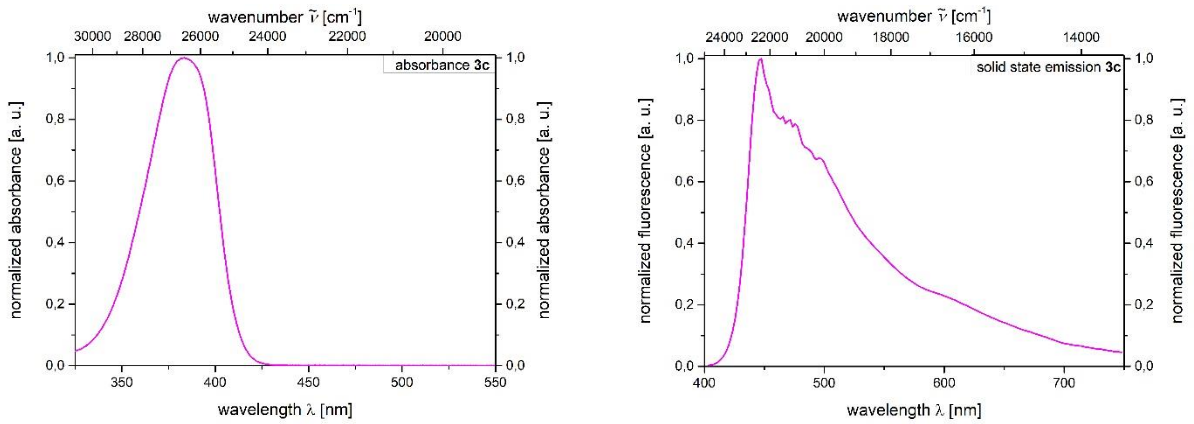

Figure S7: Absorption spectrum of compound **3c** in ethanol and solid state emission spectrum of compound **3c**

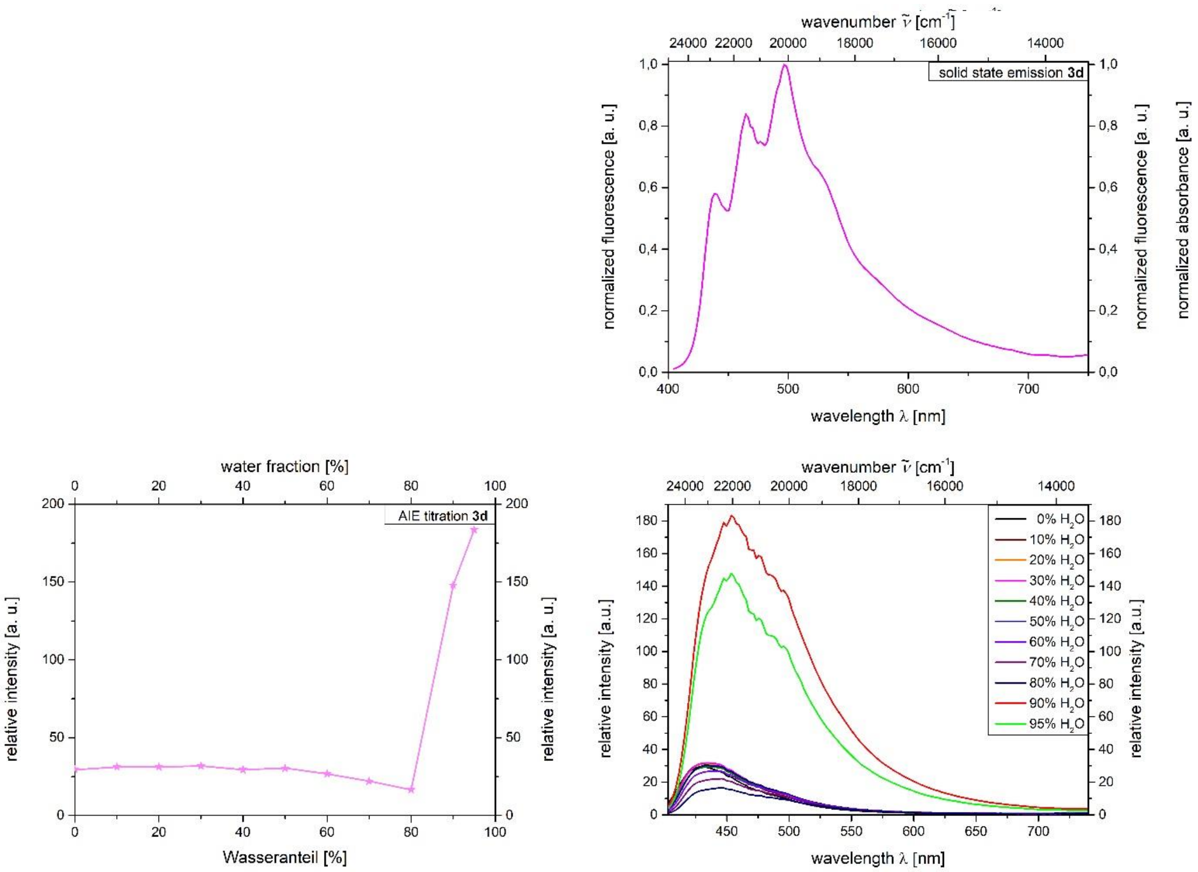

Figure S8: Absorption spectrum of compound **3d** in ethanol (top, left), solid state emission spectrum (top, right), and AIE-induced changes in emission (bottom, left) and AIE-related emission spectra of compound **3d**. The latter spectra were measured in ethanol/water mixtures of varying water content.

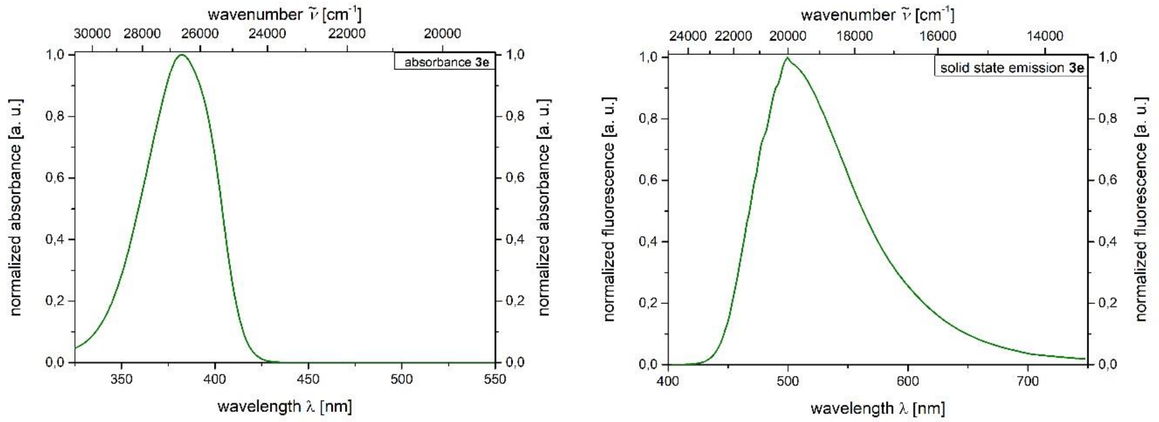

Figure S9: Absorption spectrum of compound **3e** in ethanol and solid state emission spectrum of compound **3e**

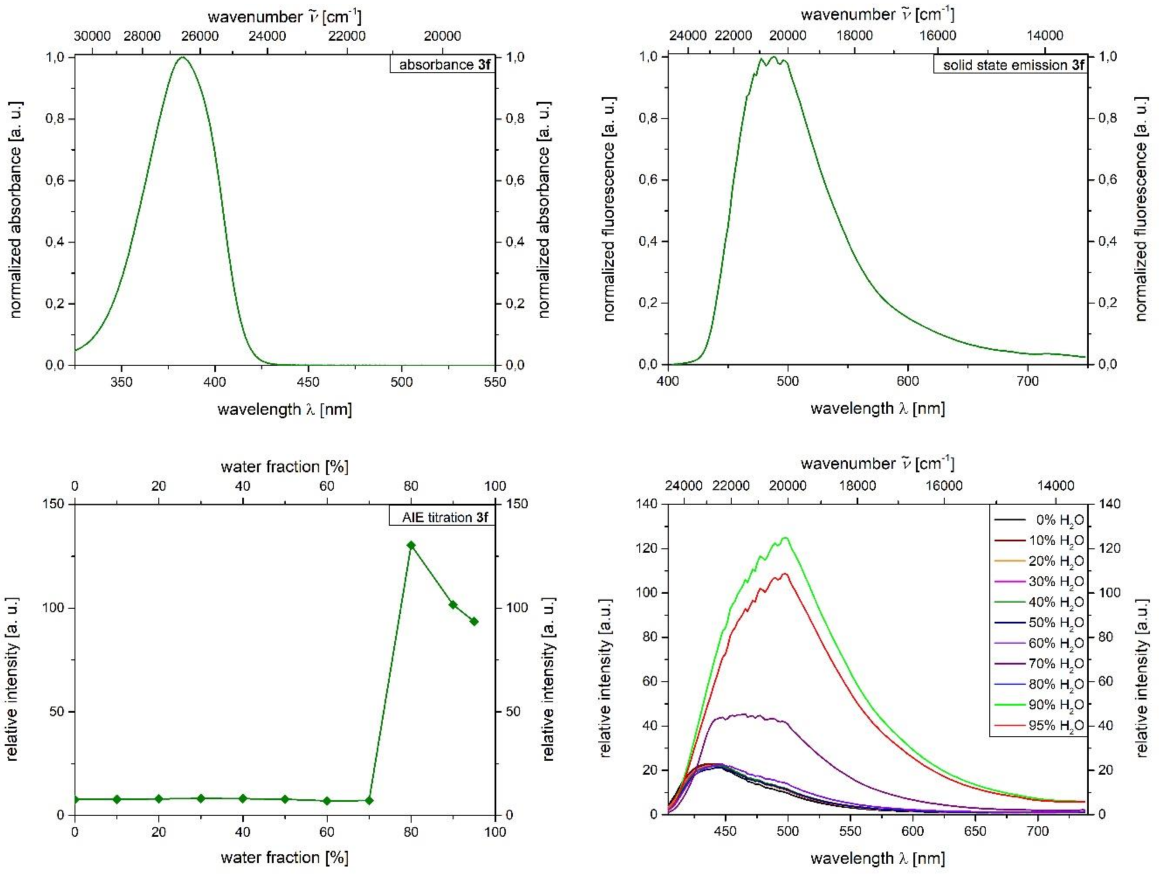

Figure S10: Absorption spectrum of compound **3f** in ethanol (top, left), solid state emission spectrum (top, right), and AIE-induced changes in emission (bottom, left) and AIE-related emission spectra of compound **3f**. The latter spectra were measured in ethanol/water mixtures of varying water content.

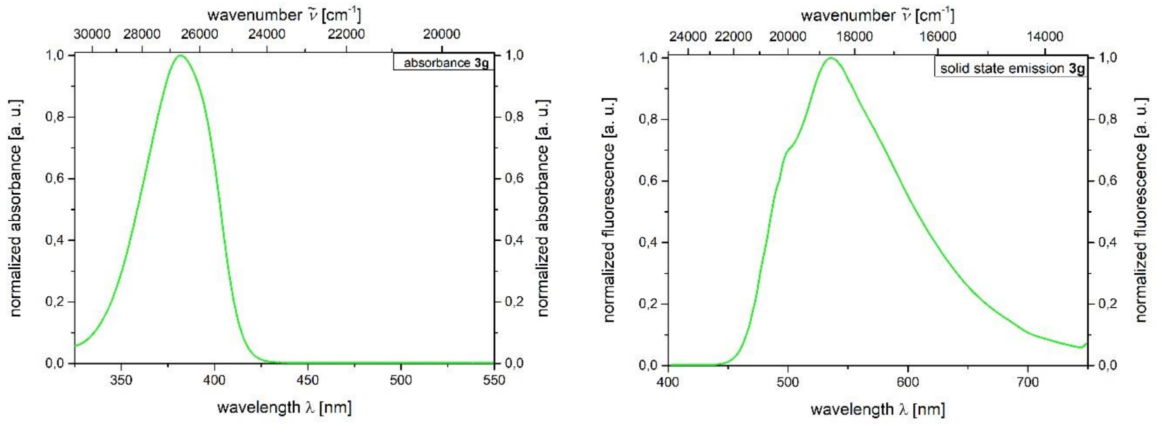

Figure S11: Absorption spectrum of compound **3e** in ethanol and solid state emission spectrum of compound **3e**

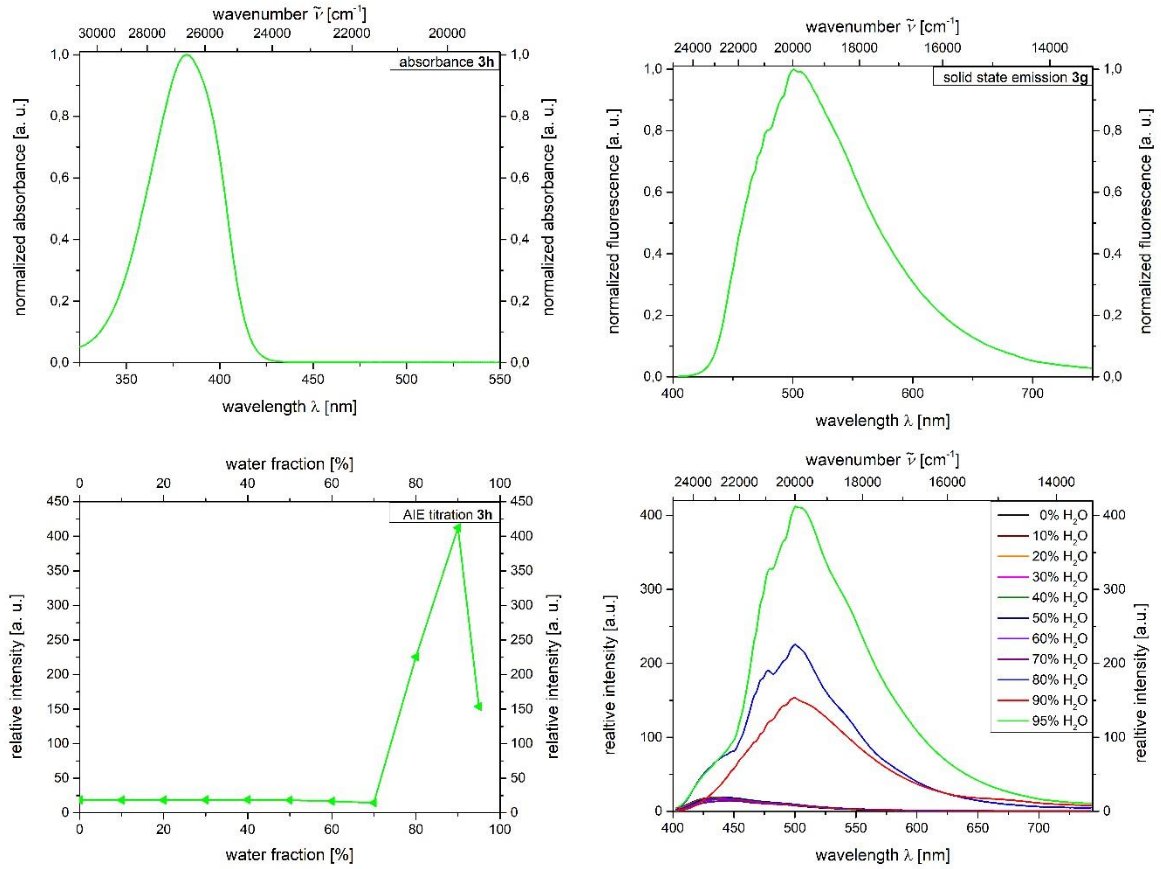

Figure S12: Absorption spectrum of compound **3h** in ethanol (top, left), solid state emission spectrum (top, right), and AIE-induced changes in emission (bottom, left) and AIE-related emission spectra of compound **3h**. The latter spectra were measured in ethanol/water mixtures of varying water content.

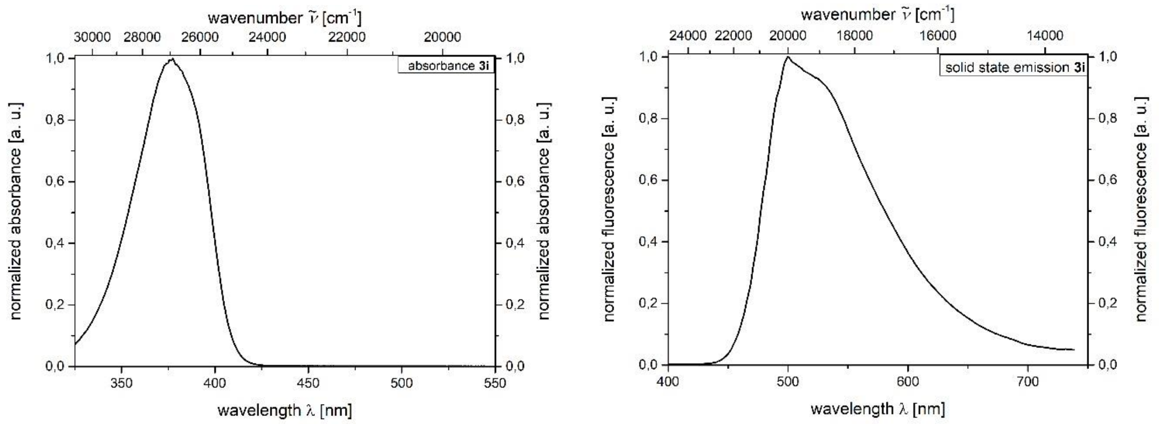

Figure S13: Absorption spectrum of compound **3i** in ethanol and solid state emission spectrum of compound **3i**

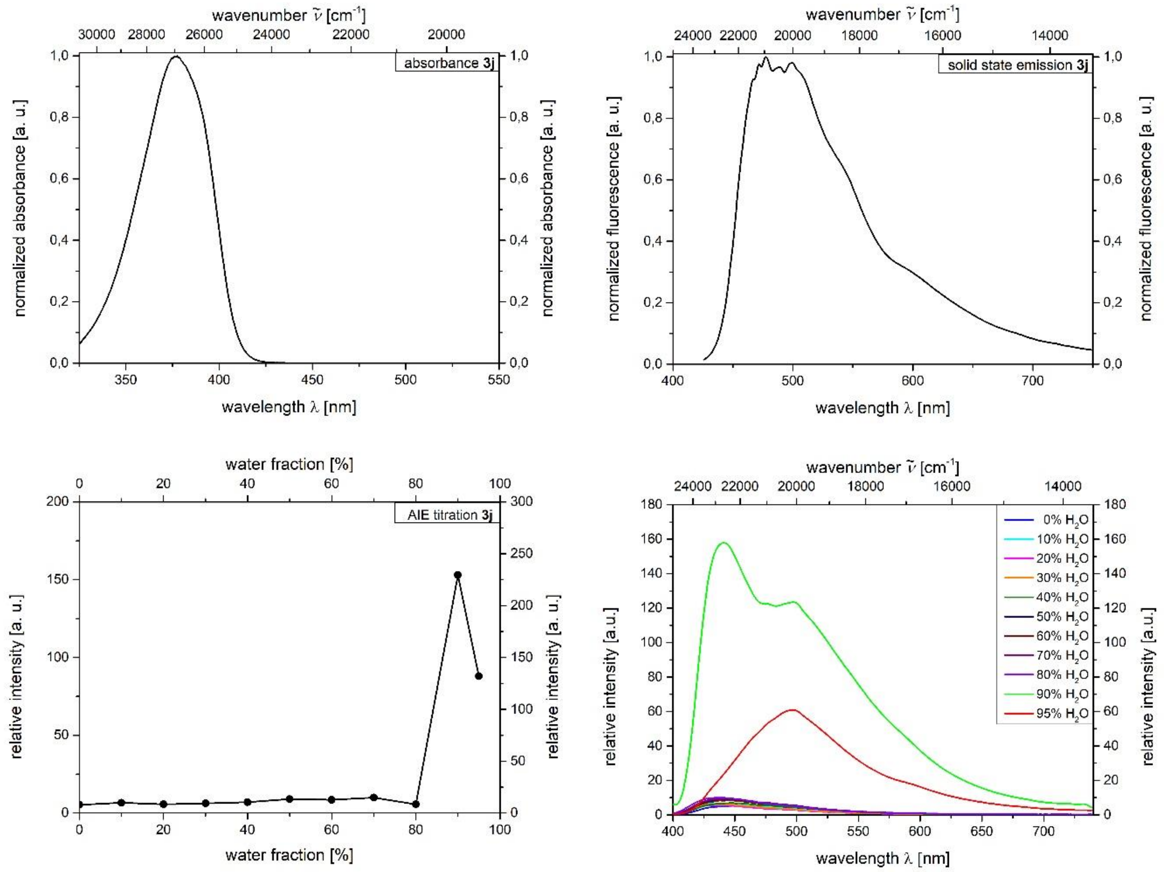

Figure S14: Absorption spectrum of compound **3j** in ethanol (top, left), solid state emission spectrum (top, right), and AIE-induced changes in emission (bottom, left) and AIE-related emission spectra of compound **3j**. The latter spectra were measured in ethanol/water mixtures of varying water content.

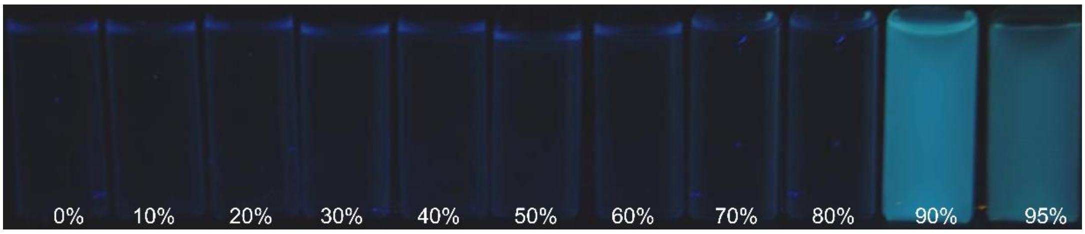

Figure S15: Optical impression of AIE-emission properties of **3j** (ethanol/water mixtures, hand held UV-lamp, λ_{exc} = 365 nm, T = 298 K).

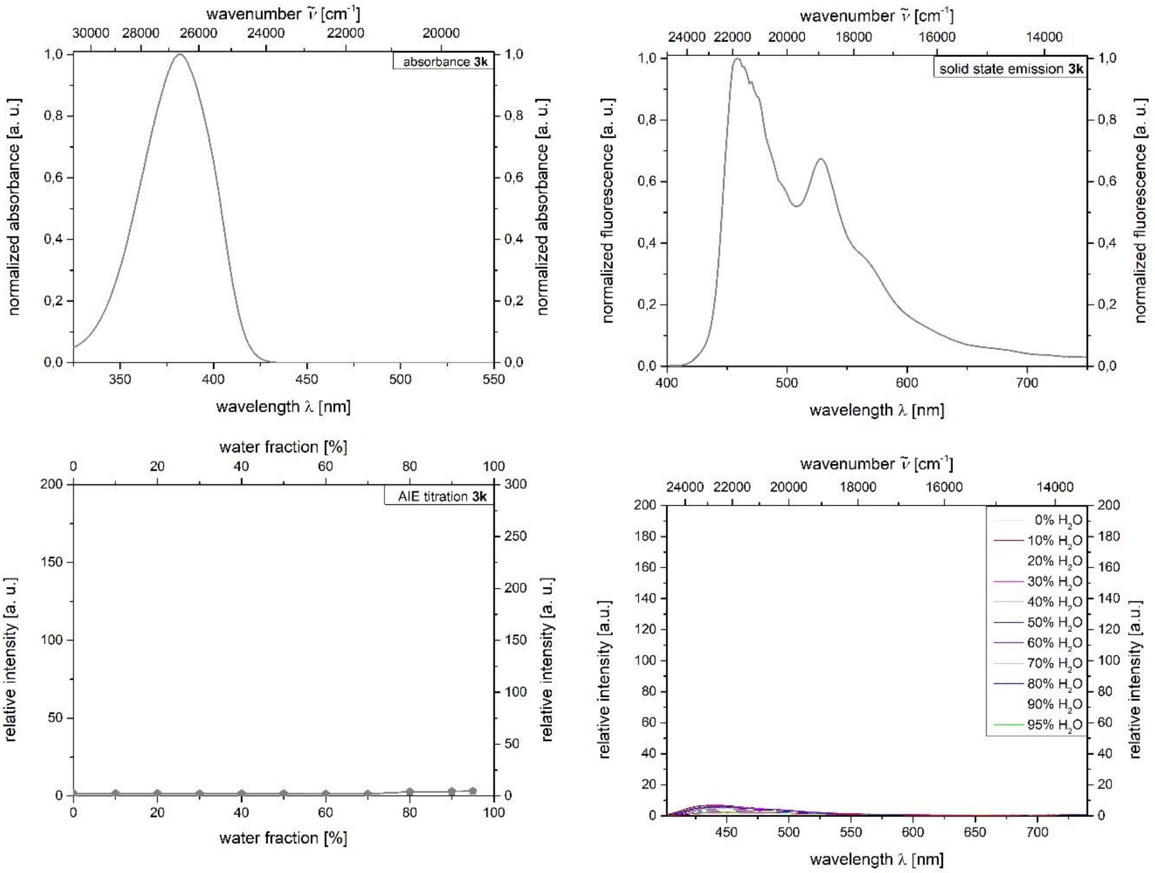

Figure S16: Absorption spectrum of compound **3k** in ethanol (top, left), solid state emission spectrum (top, right), and AIE-induced changes in emission (bottom, left) and AIE-related emission spectra of compound **3k**. The latter spectra were measured in ethanol/water mixtures of varying water content.

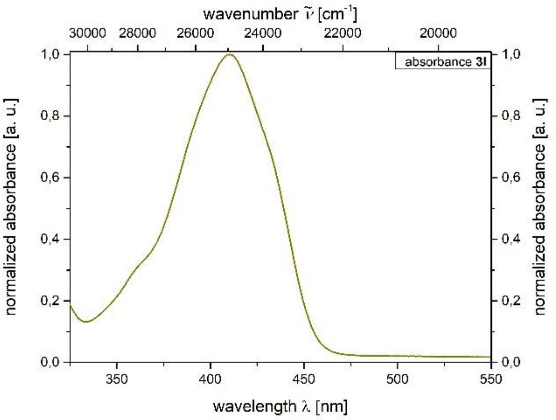

Figure S17: Absorption spectrum of compound **3l** in ethanol

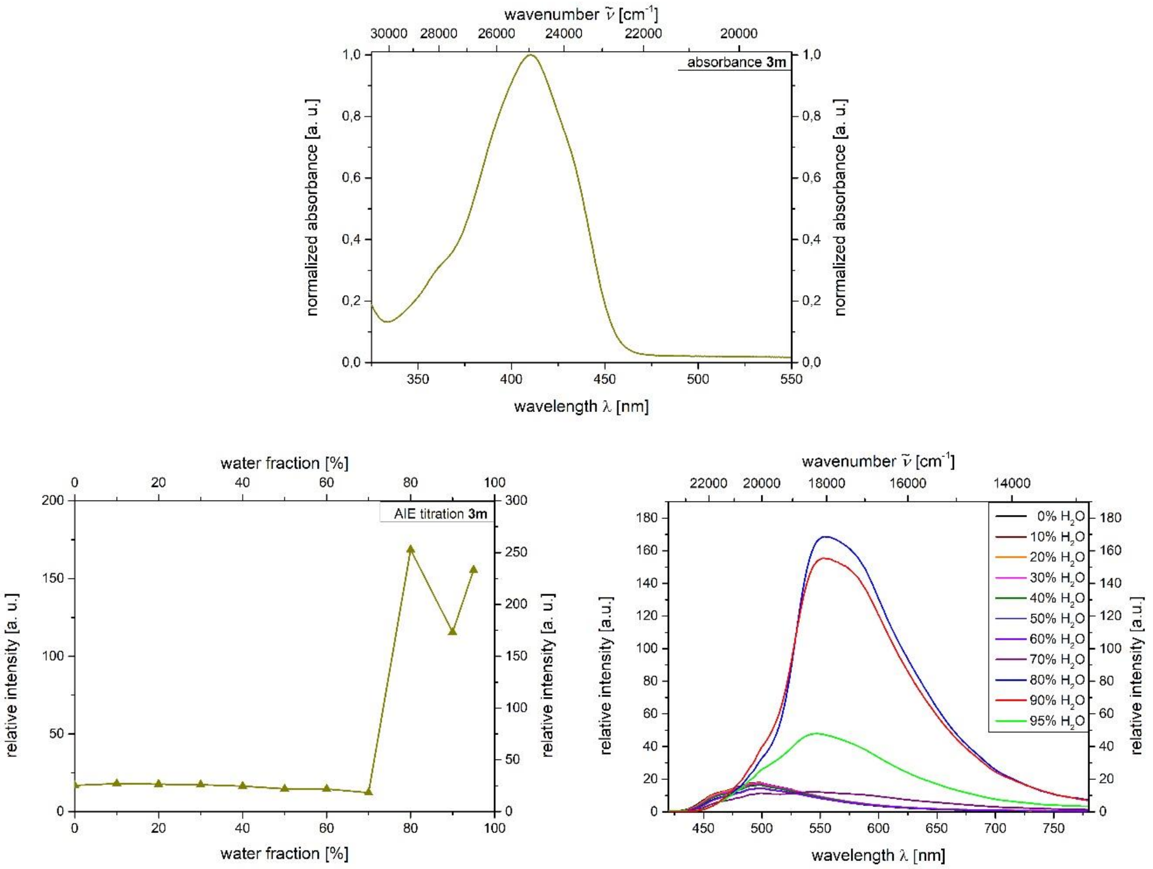

Figure S18: Absorption spectrum of compound **3m** in ethanol (top) and AIE-induced changes in emission (bottom, left) and AIE-related emission spectra of compound **3m**. The latter spectra were measured in ethanol/water mixtures of varying water content.

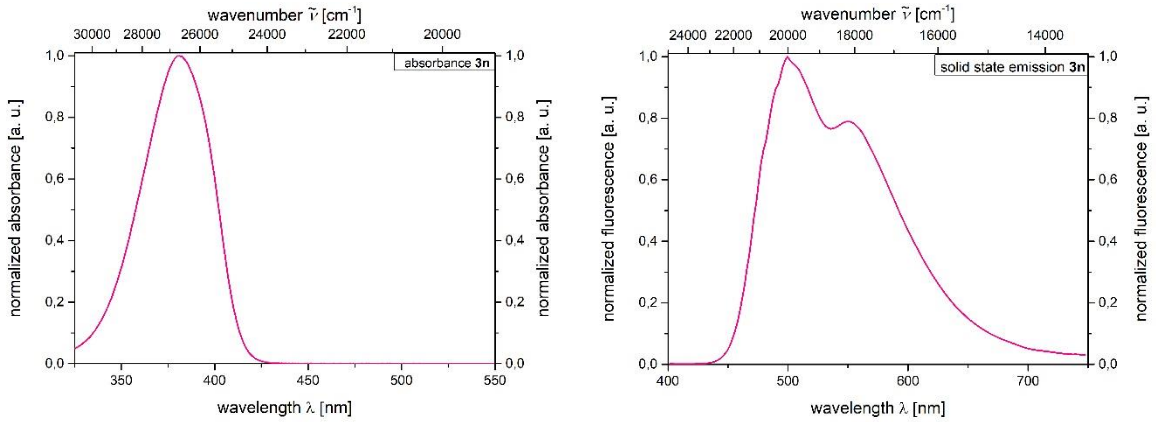

Figure S19: Absorption spectrum of compound **3n** in ethanol and solid state emission spectrum of compound **3n**

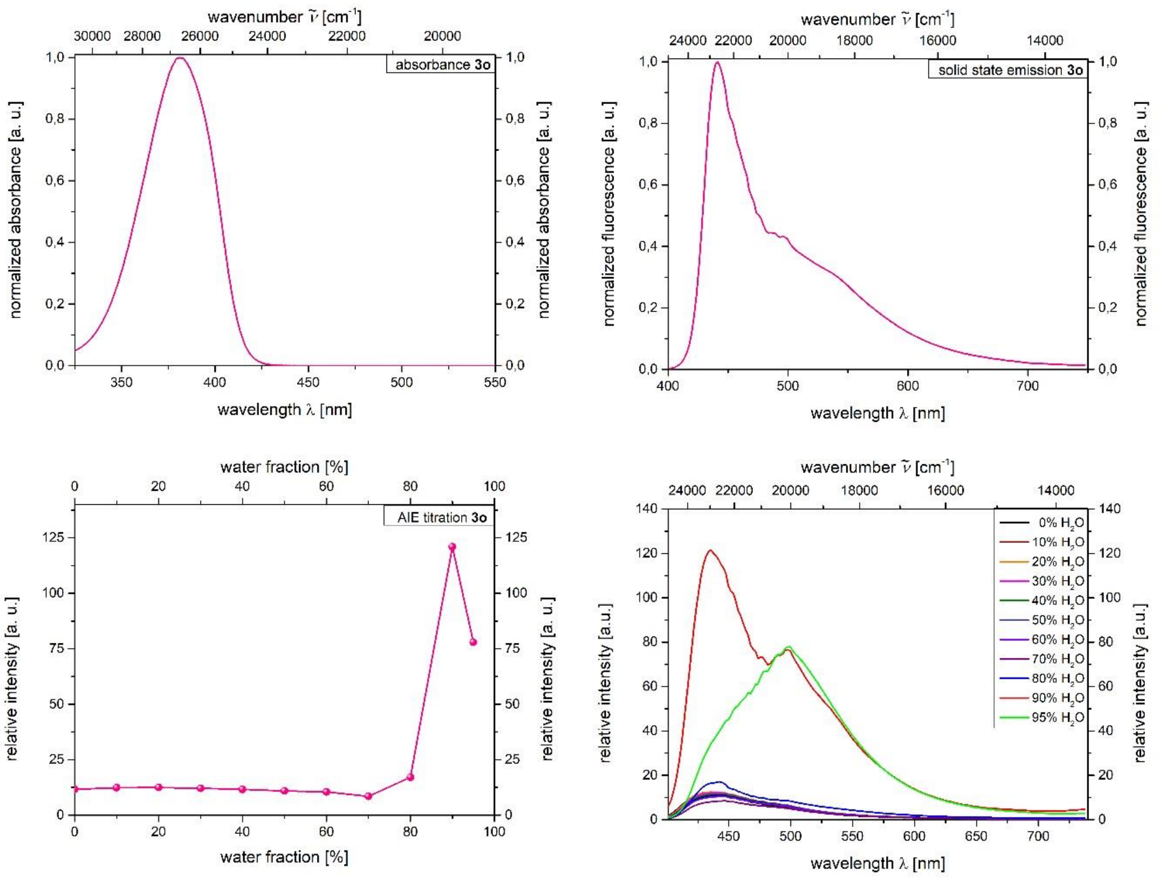

Figure S20: Absorption spectrum of compound **3o** in ethanol (top, left), solid state emission spectrum (top, right), and AIE-induced changes in emission (bottom, left) and AIE-related emission spectra of compound **3o**. The latter spectra were measured in ethanol/water mixtures of varying water content.

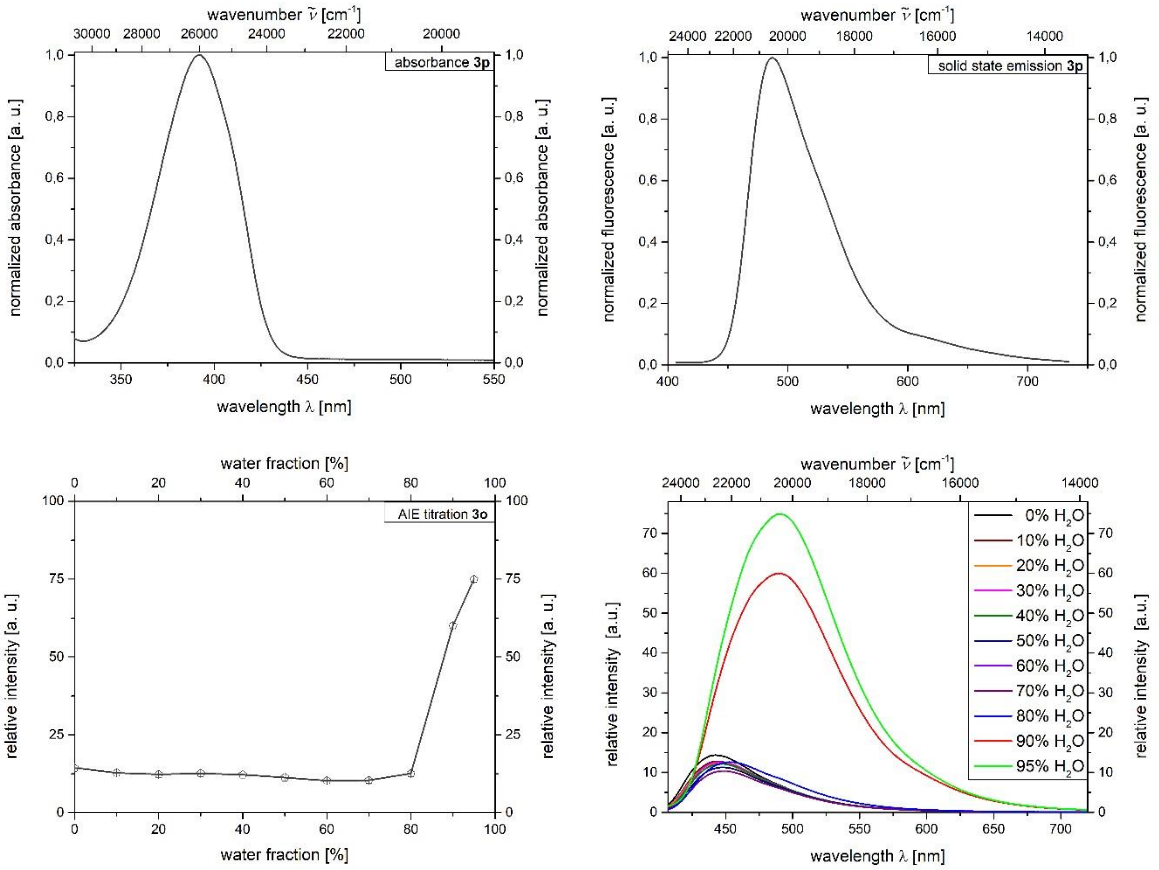

Figure S21: Absorption spectrum of compound **3p** in ethanol (top, left), solid state emission spectrum (top, right), and AIE-induced changes in emission (bottom, left) and AIE-related emission spectra of compound **3p**. The latter spectra were measured in ethanol/water mixtures of varying water content.

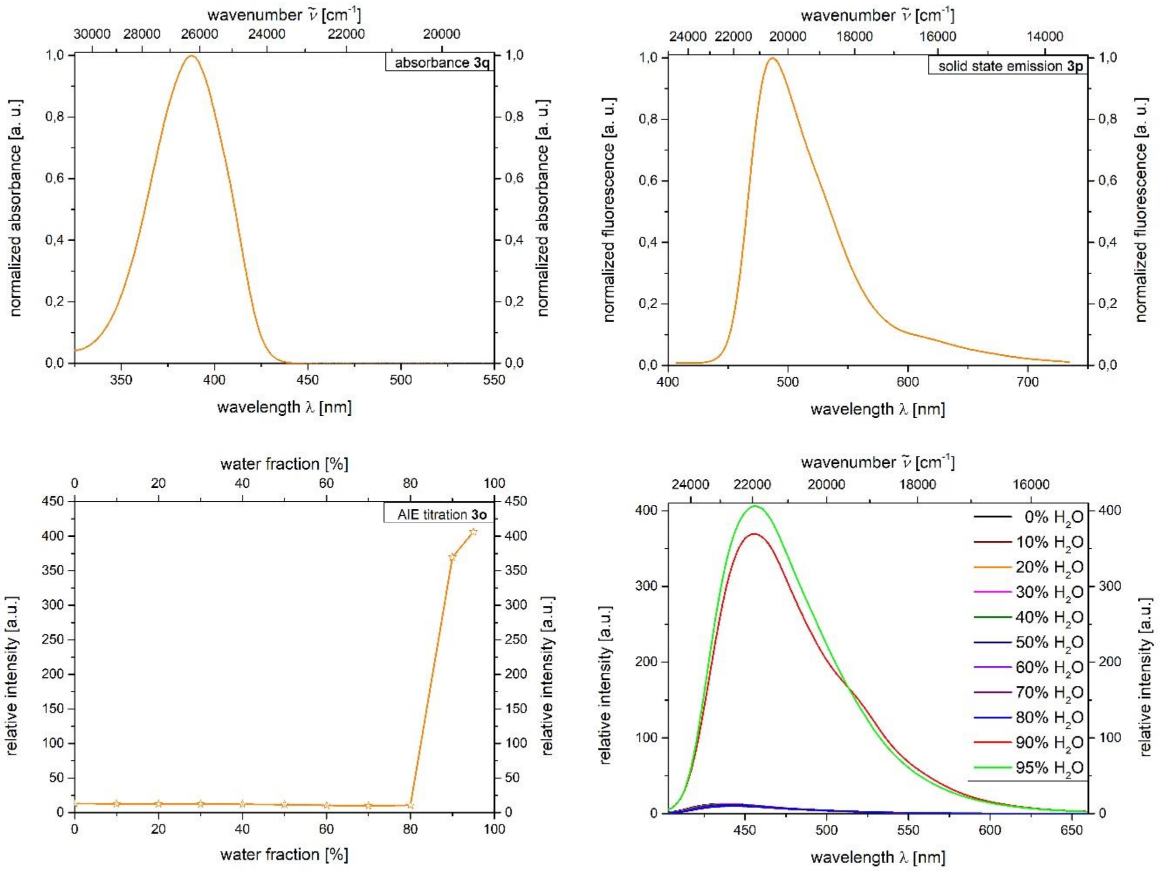

Figure S22: Absorption spectrum of compound **3q** in ethanol (top, left), solid state emission spectrum (top, right), and AIE-induced changes in emission (bottom, left) and AIE-related emission spectra of compound **3q**. The latter spectra were measured in ethanol/water mixtures of varying water content.

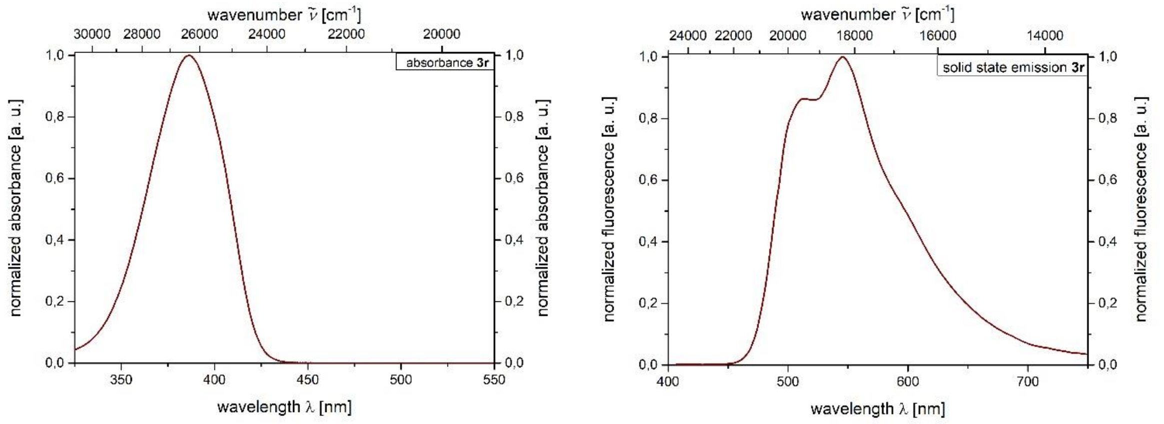

Figure S23: Absorption spectrum of compound **3r** in ethanol and solid state emission spectrum of compound **3r**

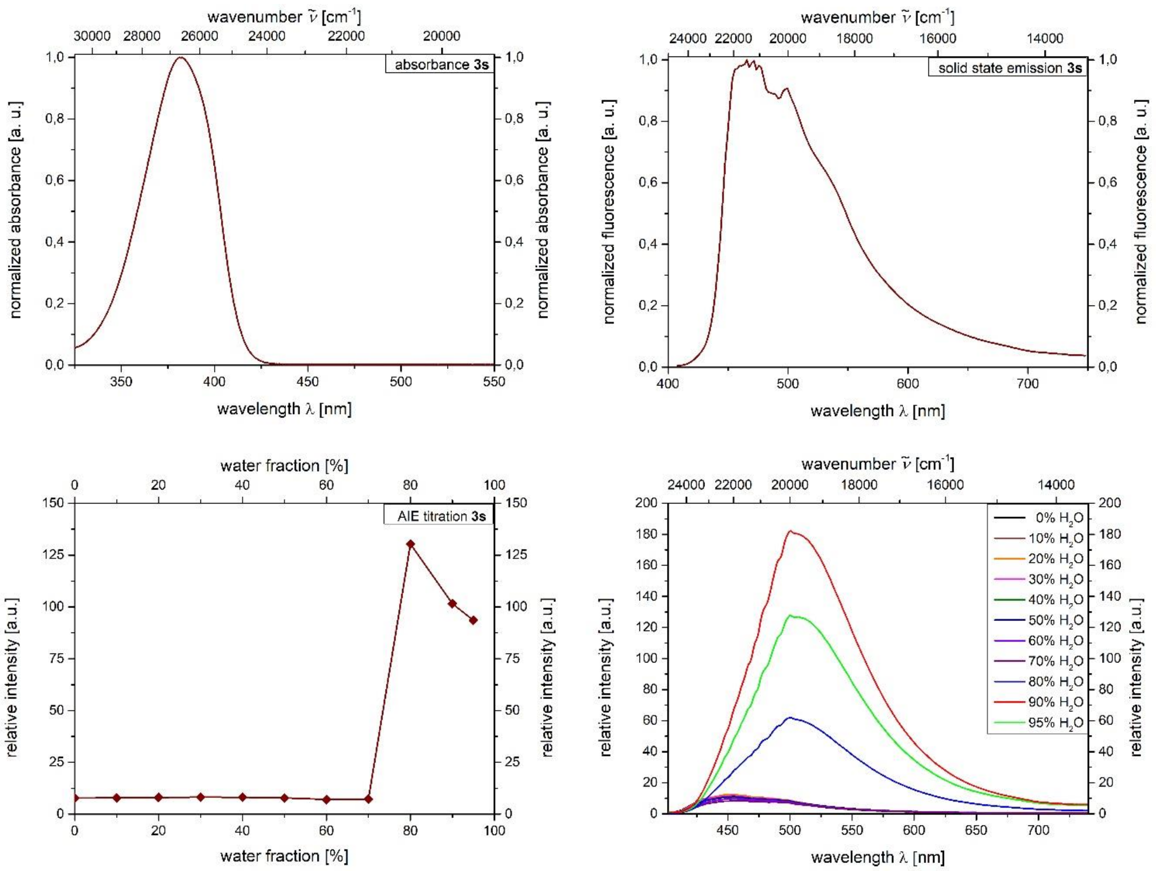

Figure S24: Absorption spectrum of compound **3s** in ethanol (top, left), solid state emission spectrum (top, right), and AIE-induced changes in emission (bottom, left) and AIE-related emission spectra of compound **3s**. The latter spectra were measured in ethanol/water mixtures of varying water content.

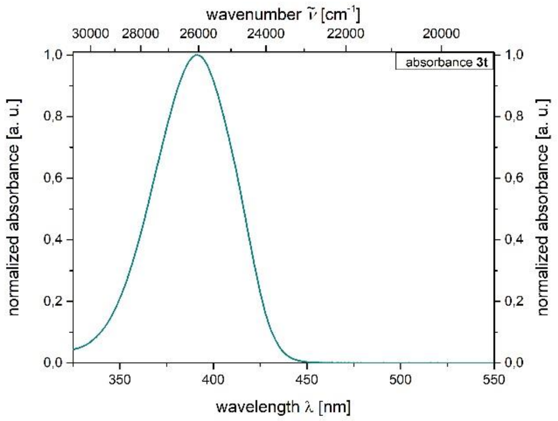

Figure S25: Absorption spectrum of compound **3t** in ethanol

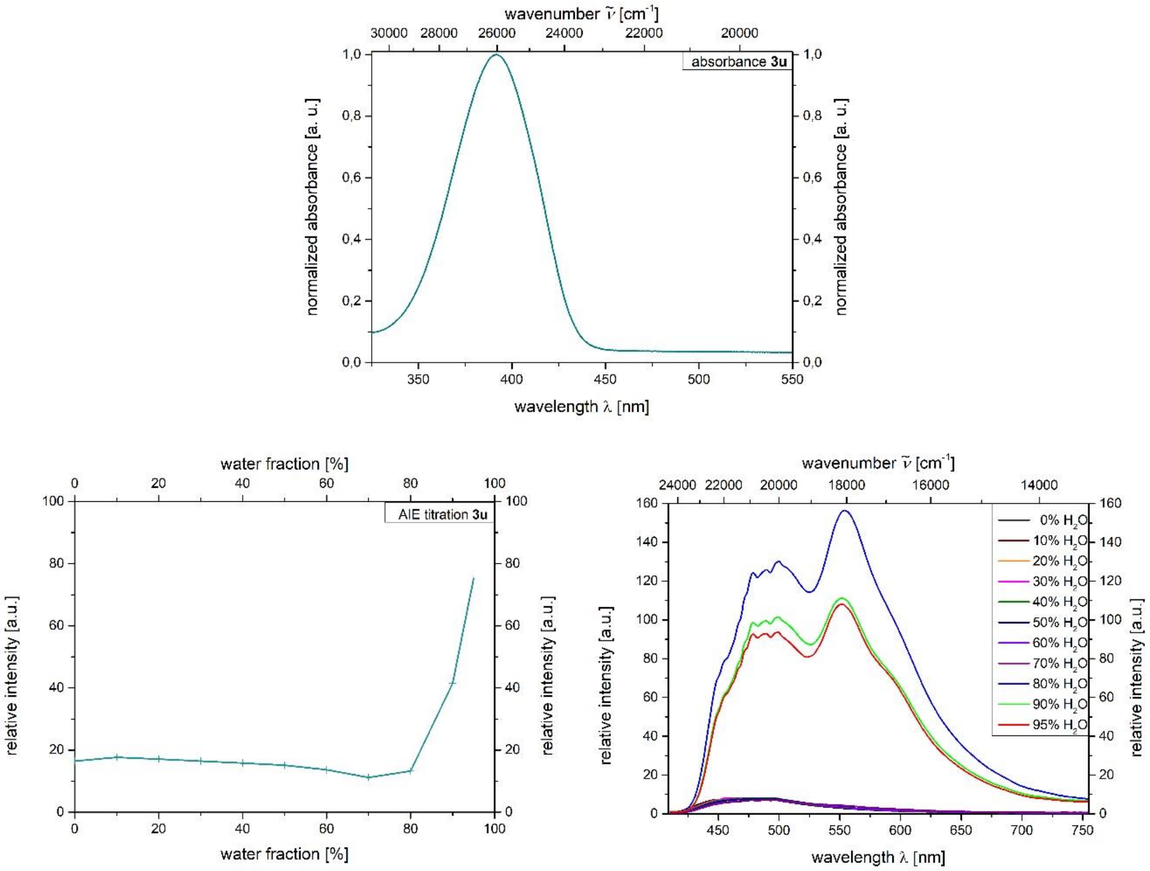

Figure S26: Absorption spectrum of compound **3u** in ethanol (top) and AIE-induced changes in emission (bottom, left) and AIE-related emission spectra of compound **3u**. The latter spectra were measured in ethanol/water mixtures of varying water content.

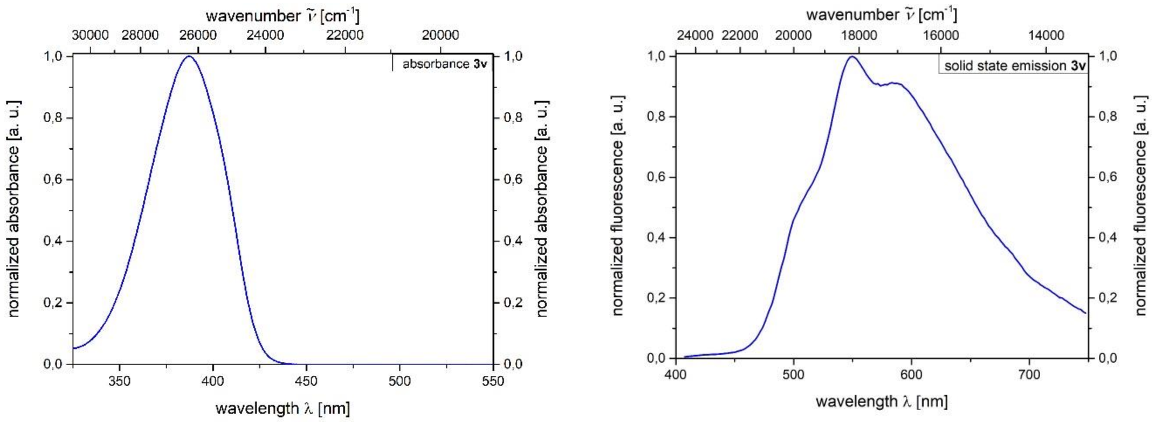

Figure S27: Absorption spectrum of compound **3v** in ethanol and solid state emission spectrum of compound **3v**

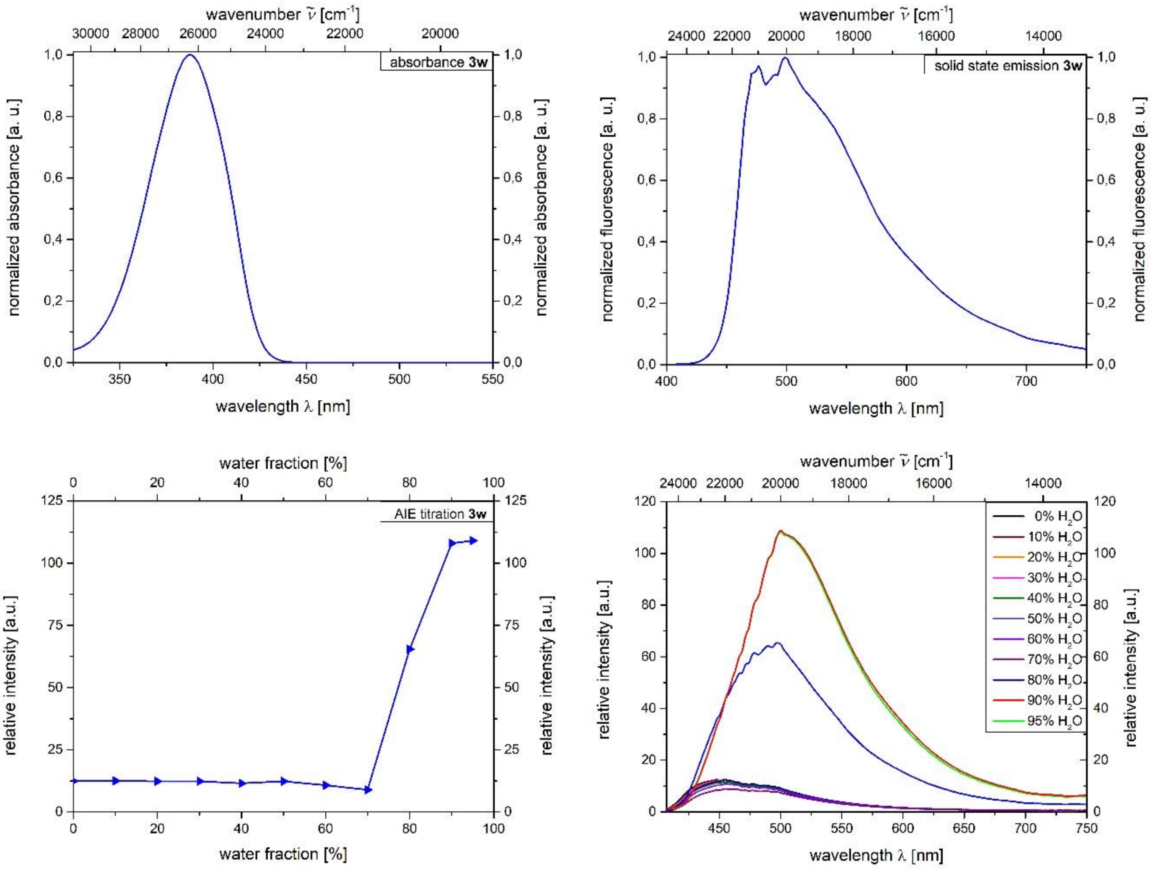

Figure S28: Absorption spectrum of compound **3w** in ethanol (top, left), solid state emission spectrum (top, right), and AIE-induced changes in emission (bottom, left) and AIE-related emission spectra of compound **3w**. The latter spectra were measured in ethanol/water mixtures of varying water content.

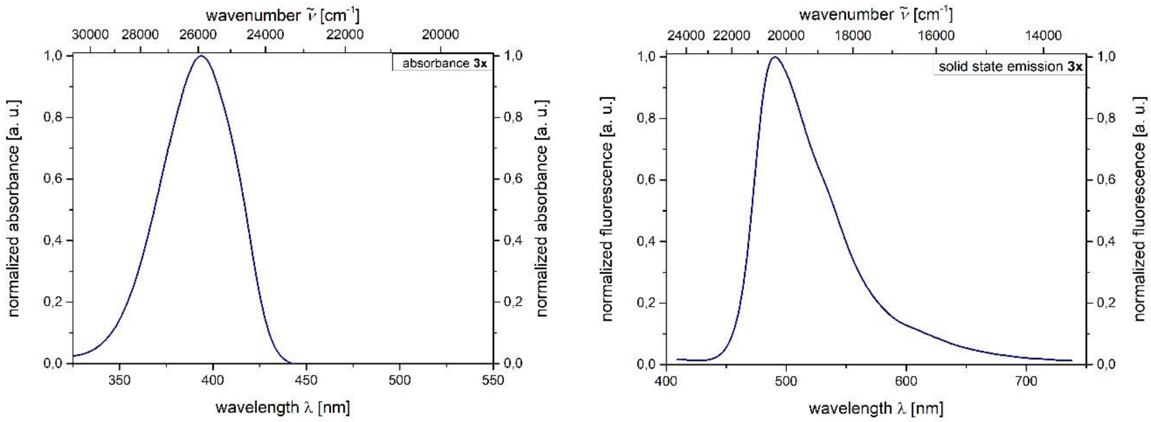

Figure S29: Absorption spectrum of compound **3x** in ethanol and solid state emission spectrum of compound **3x**

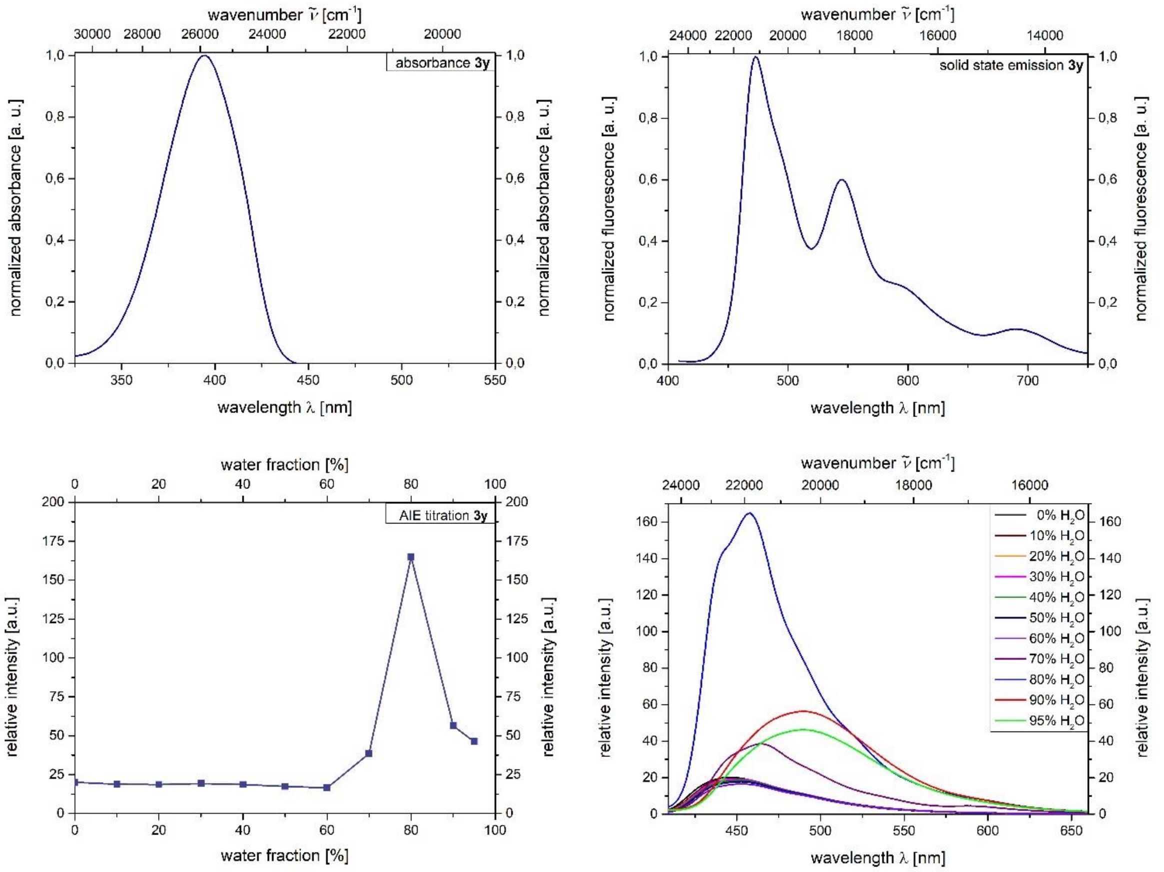

Figure S30: Absorption spectrum of compound **3y** in ethanol (top, left), solid state emission spectrum (top, right), and AIE-induced changes in emission (bottom, left) and AIE-related emission spectra of compound **3y**. The latter spectra were measured in ethanol/water mixtures of varying water content.

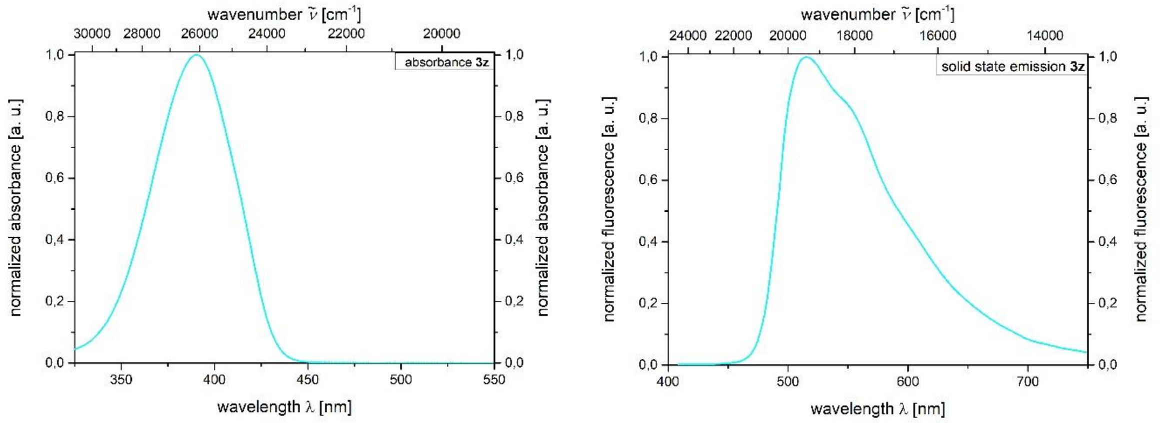

Figure S31: Absorption spectrum of compound **3z** in ethanol and solid state emission spectrum of compound **3z**

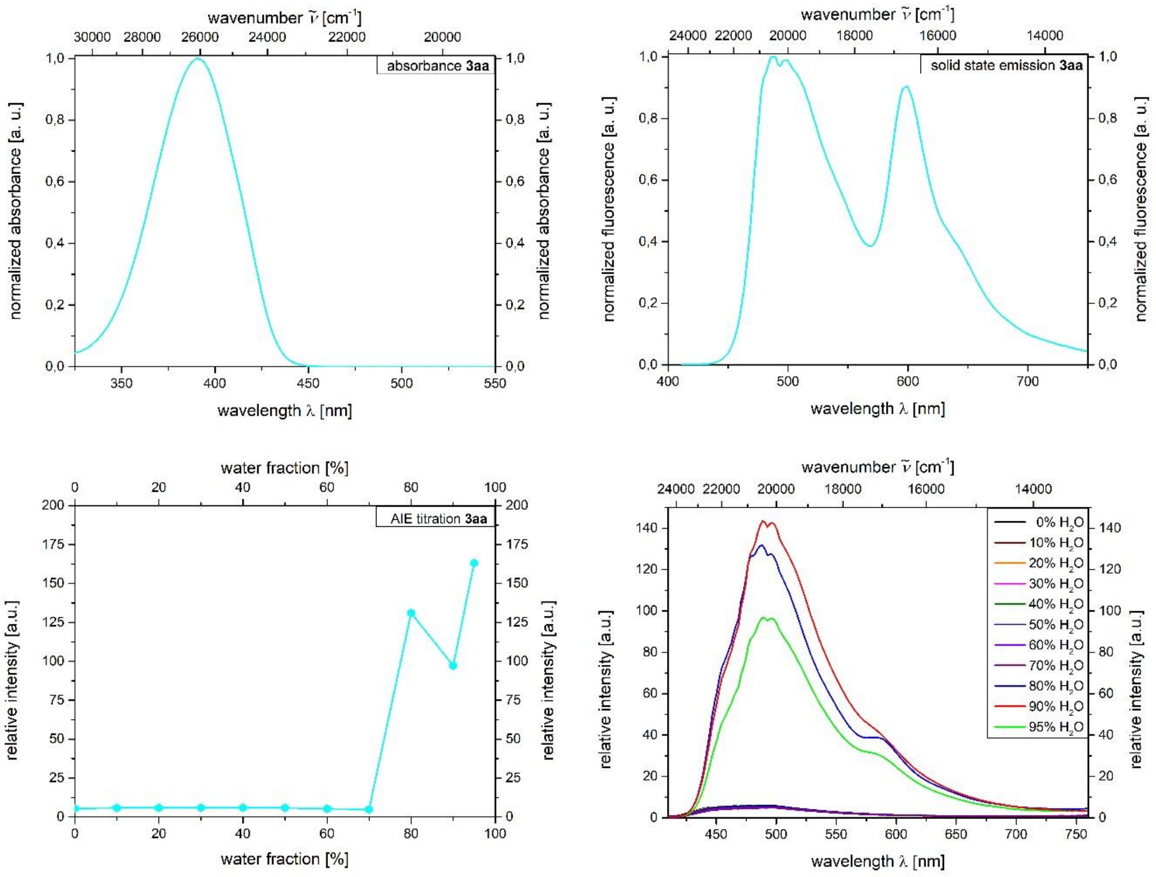

Figure S32: Absorption spectrum of compound **3aa** in ethanol (top, left), solid state emission spectrum (top, right), and AIE-induced changes in emission (bottom, left) and AIE-related emission spectra of compound **3aa**. The latter spectra were measured in ethanol/water mixtures of varying water content.

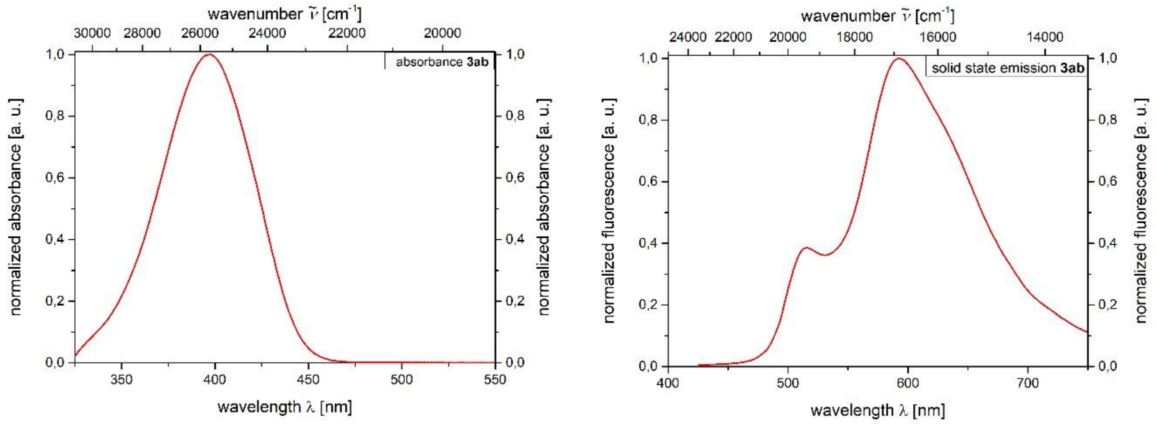

Figure S33: Absorption spectrum of compound **3ab** in ethanol and solid state emission spectrum of compound **3ab**

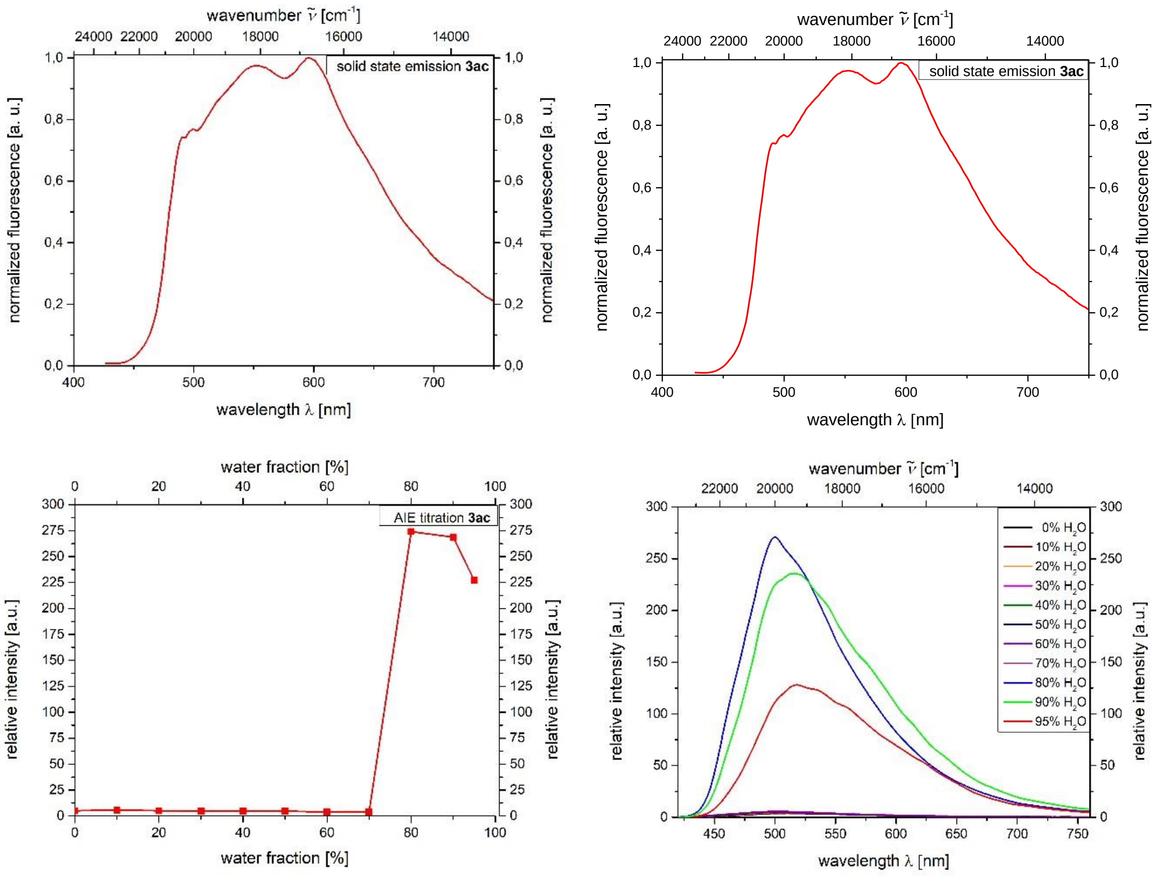

Figure S34: Absorption spectrum of compound **3ac** in ethanol (top, left), solid state emission spectrum (top, right), and AIE-induced changes in emission (bottom, left) and AIE-related emission spectra of compound **3ac**. The latter spectra were measured in ethanol/water mixtures of varying water content.

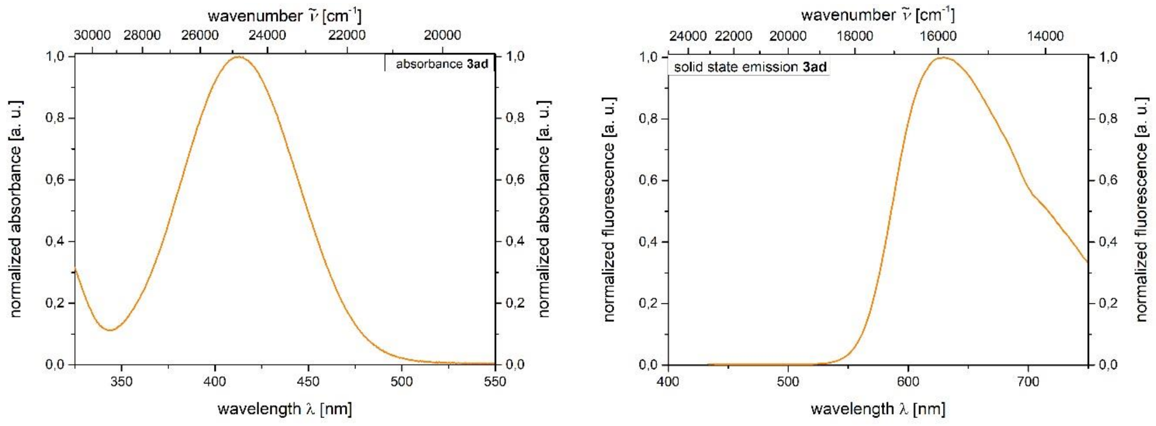

Figure S35: Absorption spectrum of compound **3ad** in ethanol and solid state emission spectrum of compound **3ad**

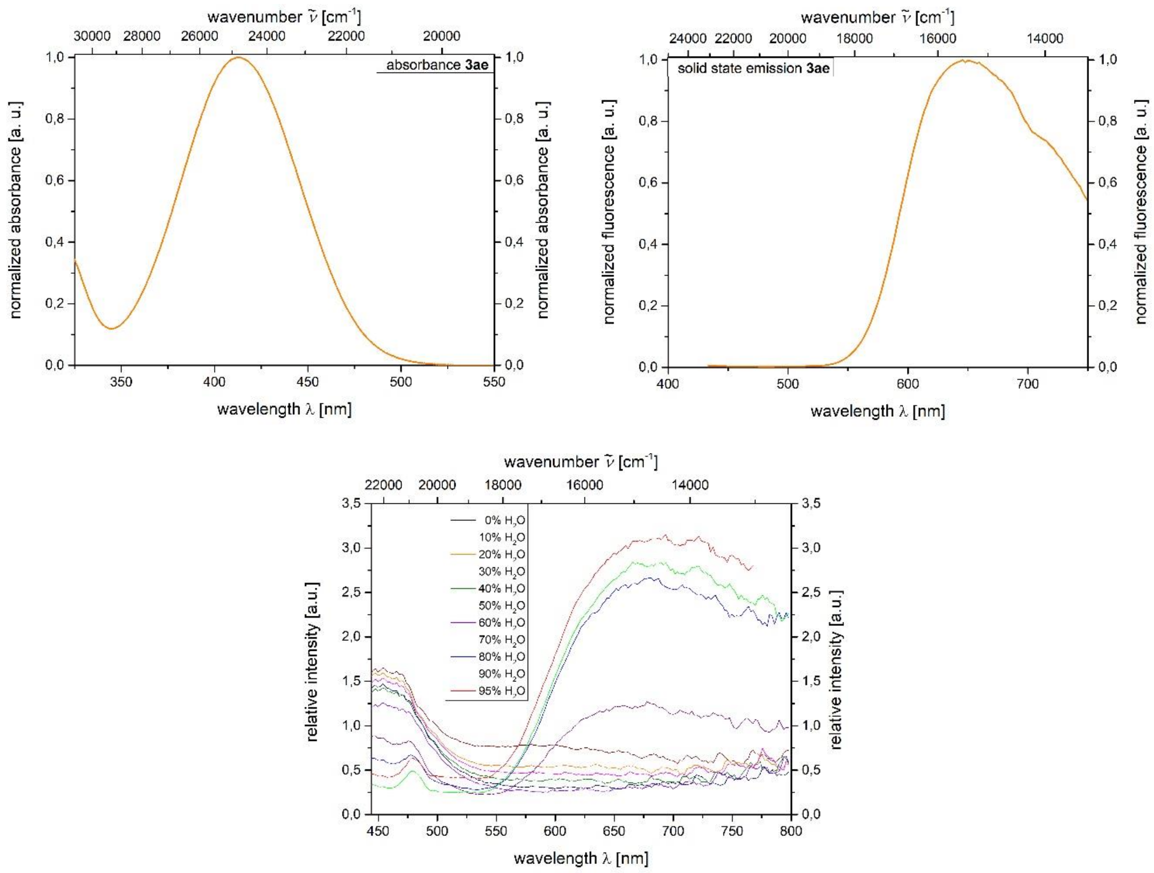

Figure S36: Absorption spectrum of compound **3ae** in ethanol (top, left), solid state emission spectrum (top, right) and AIE-related emission spectra of compound **3ae**. The latter spectra were measured in ethanol/water mixtures of varying water content.

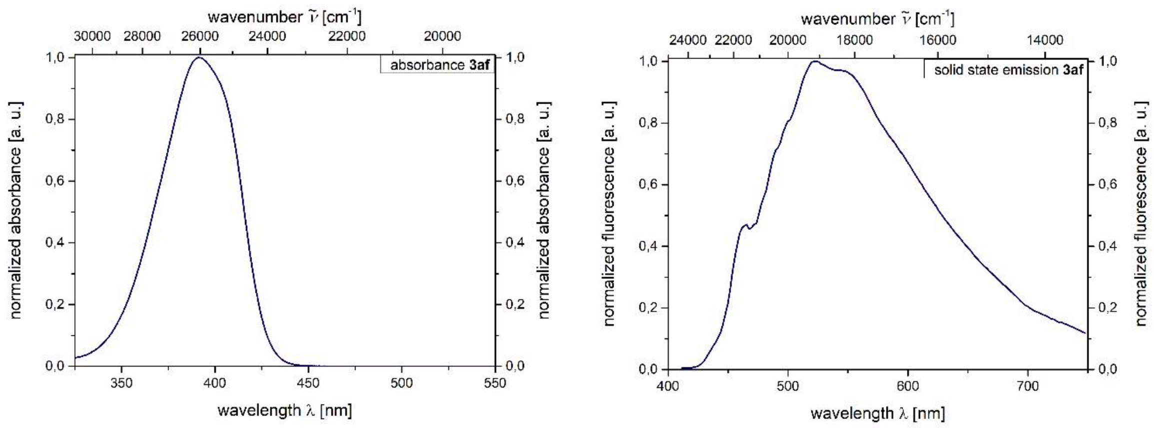

Figure S37: Absorption spectrum of compound **3af** in ethanol and solid state emission spectrum of compound **3af**

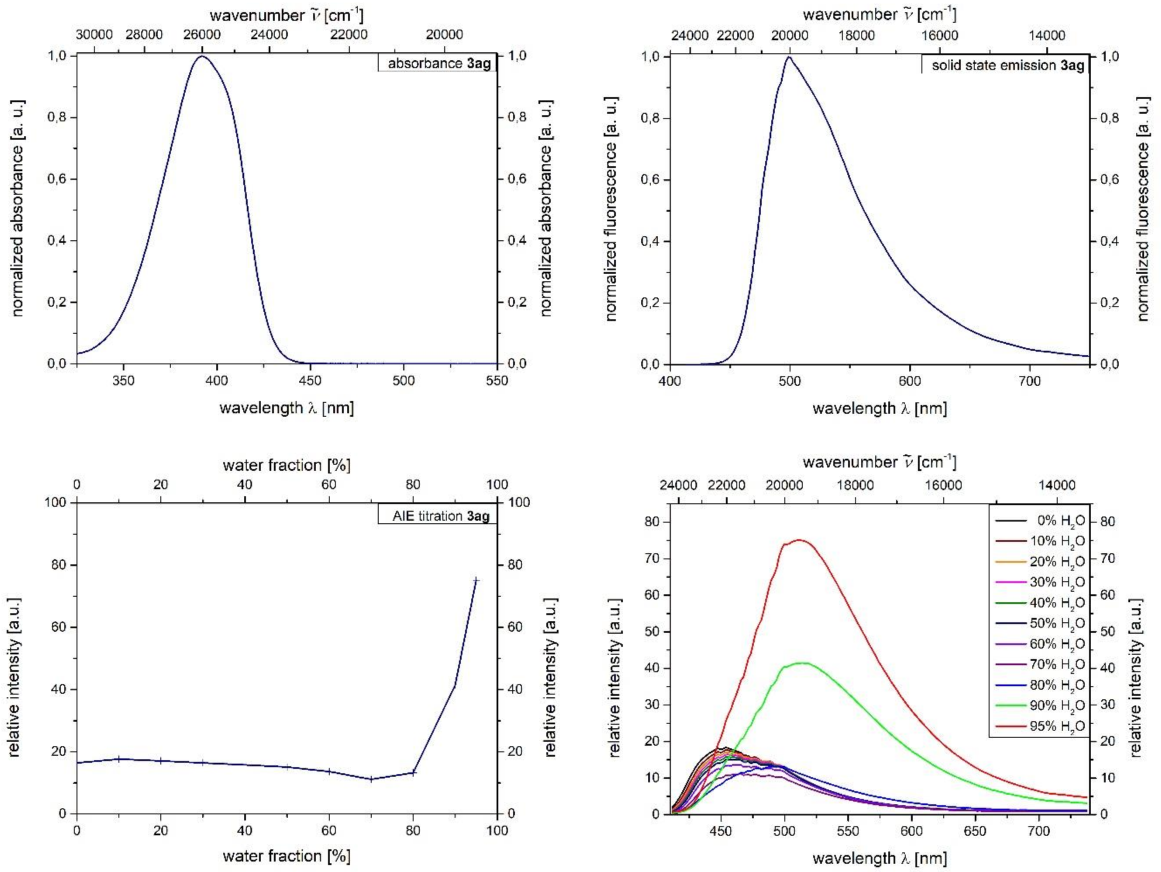

Figure S38: Absorption spectrum of compound **3ag** in ethanol (top, left), solid state emission spectrum (top, right), and AIE-induced changes in emission (bottom, left) and AIE-related emission spectra of compound **3ag**. The latter spectra were measured in ethanol/water mixtures of varying water content.

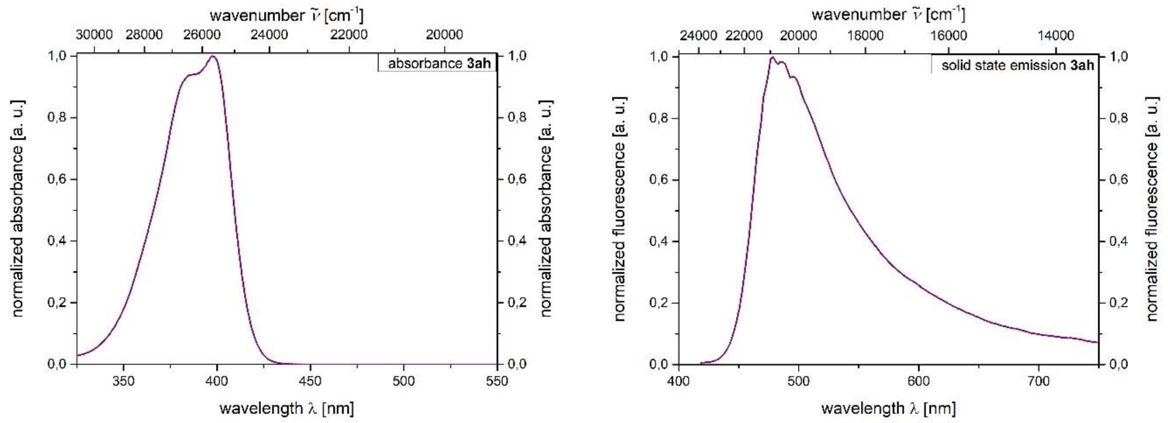

Figure S39: Absorption spectrum of compound **3ah** in ethanol and solid state emission spectrum of compound **3ah**

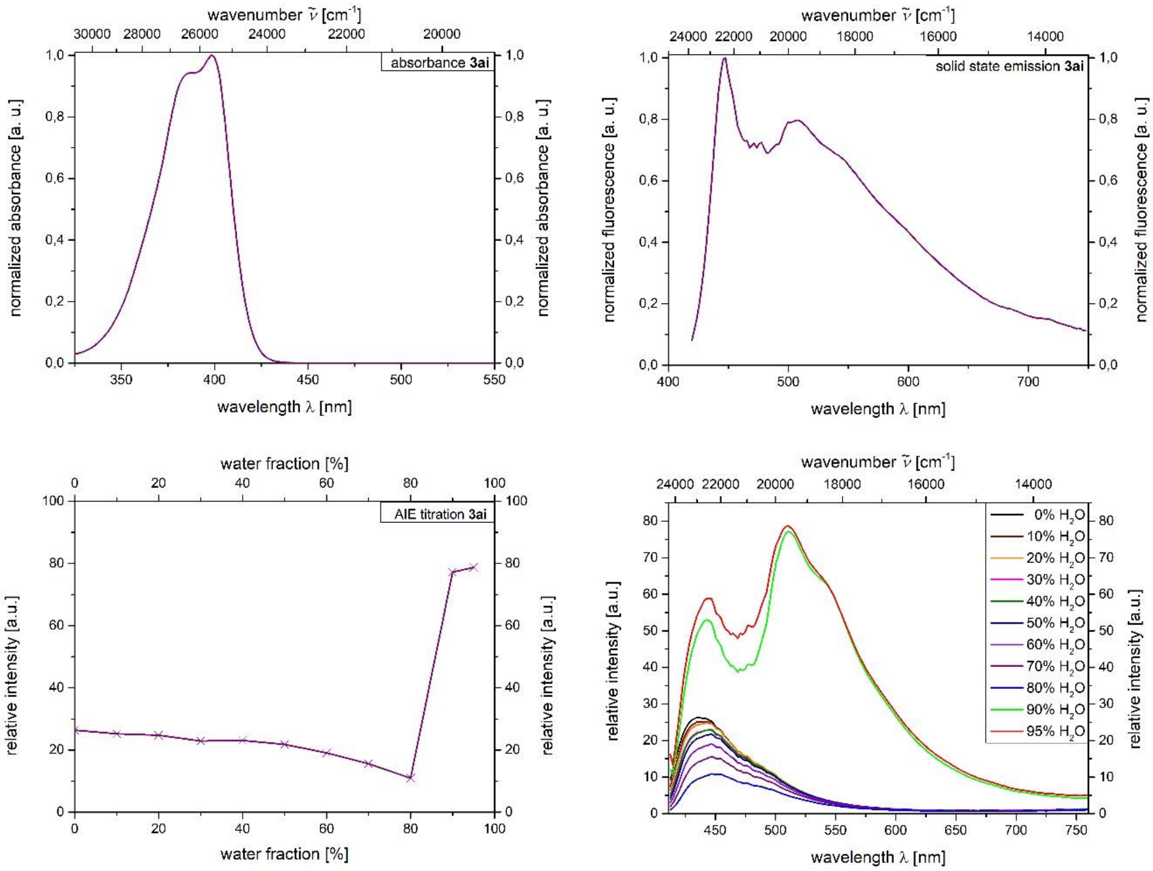

Figure S40: Absorption spectrum of compound **3ai** in ethanol (top, left), solid state emission spectrum (top, right), and AIE-induced changes in emission (bottom, left) and AIE-related emission spectra of compound **3ai**. The latter spectra were measured in ethanol/water mixtures of varying water content.

Additional measurements of dyes **3d**, **3j**, **3s**, **3ac** and **3ag** were performed at the Division Biophotonics, Bundesanstalt für Materialforschung und -prüfung (BAM) in Berlin by Dr. N. Nirmalananthan-Budau, Dr. K. Hoffmann and Dr. U. Resch-Genger.

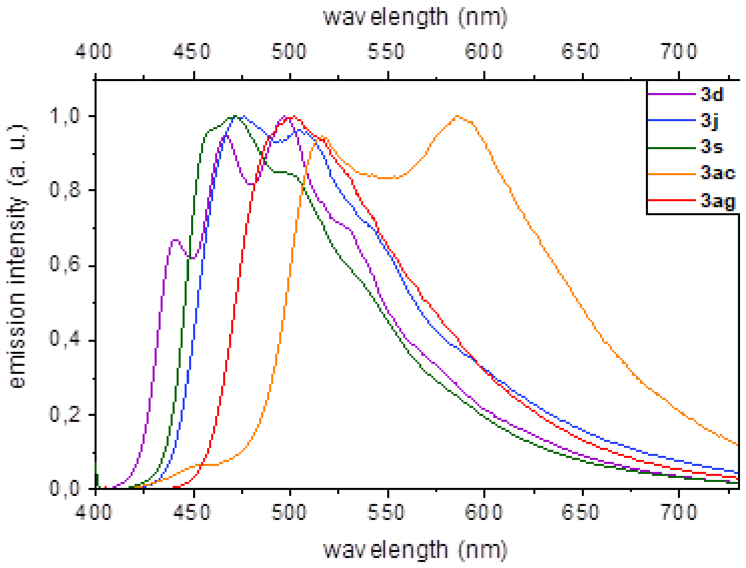

Figure S41: Solid state emission spectra of compounds **3d**, **3j**, **3s**, **3ac** and **3ag**

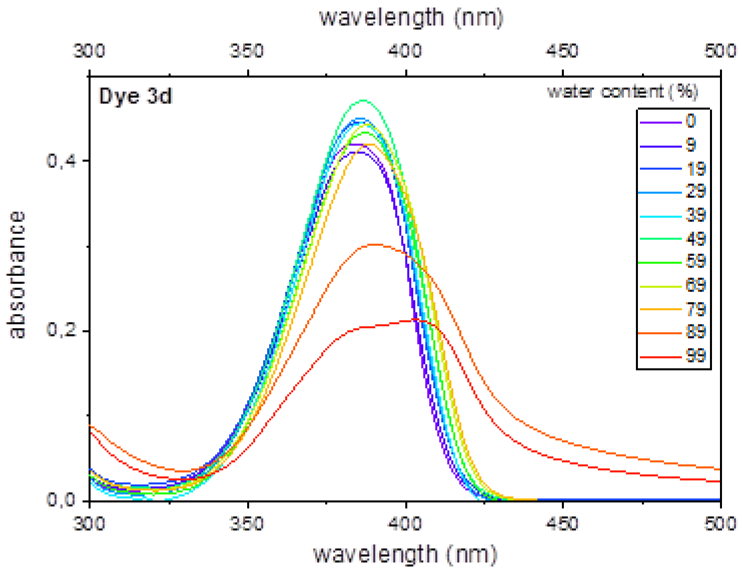

Figure S42: Absorption spectra of compound **3d** in ethanol/water mixtures of varying water content

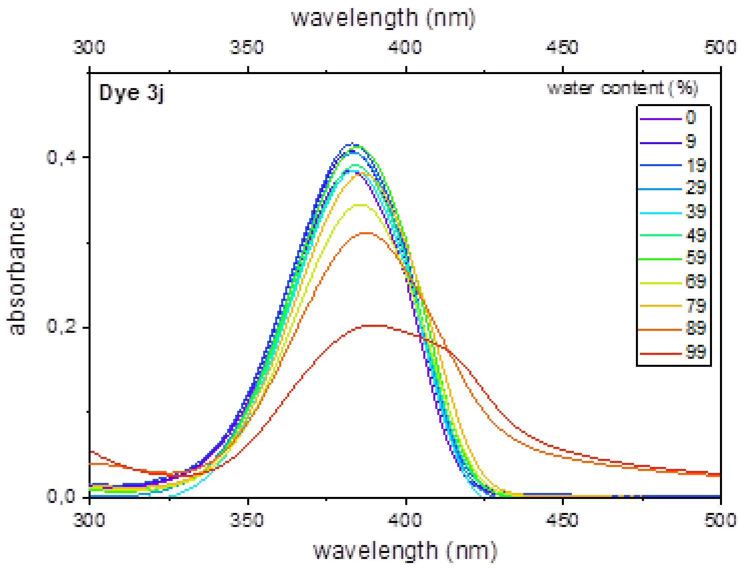

Figure S43: Absorption spectra of compound **3j** in ethanol/water mixtures of varying water content

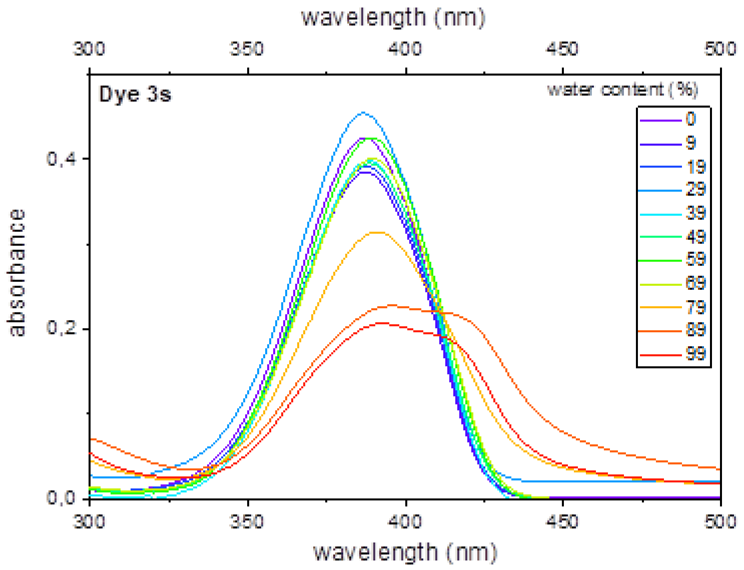

Figure S44: Absorption spectra of compound **3s** in ethanol/water mixtures of varying water content

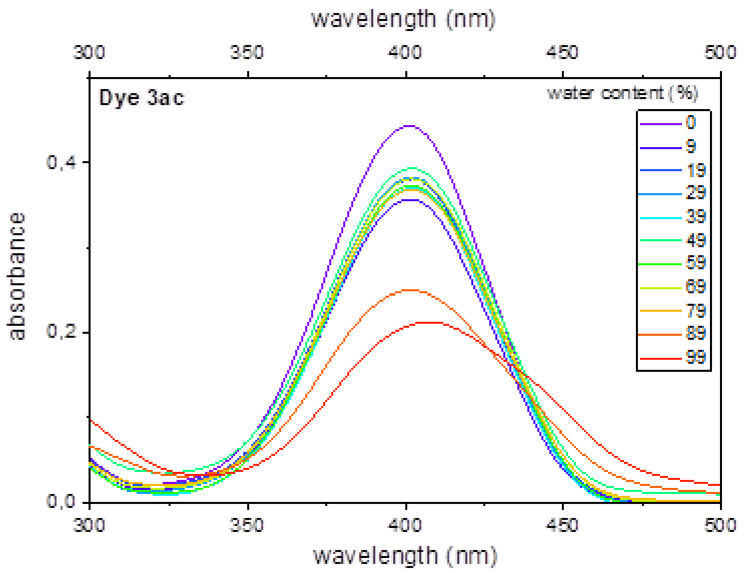

Figure S45: Absorption spectra of compound **3ac** in ethanol/water mixtures of varying water content

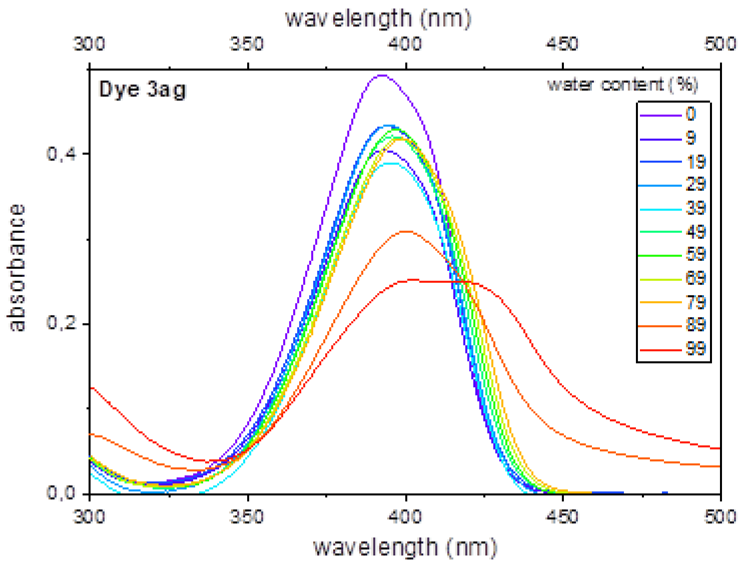

Figure S46: Absorption spectra of compound **3ag** in ethanol/water mixtures of varying water content

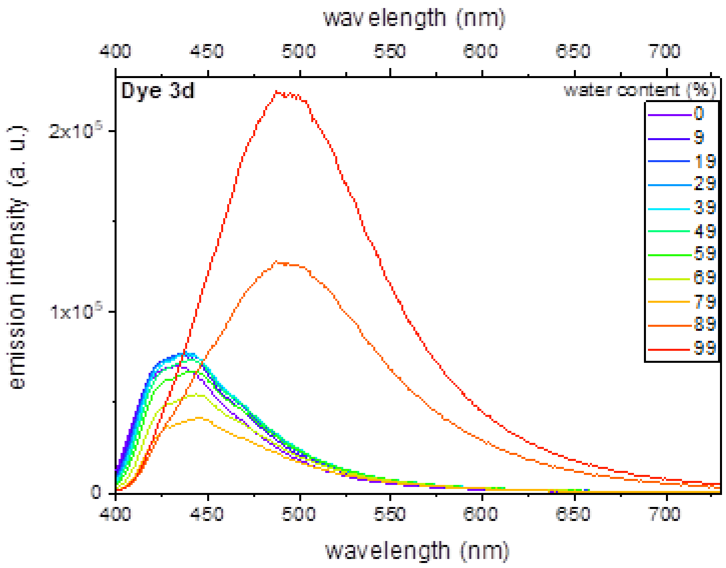

Figure S47: AIE emission spectra of compound **3d** obtained in ethanol/water mixtures of varying water content

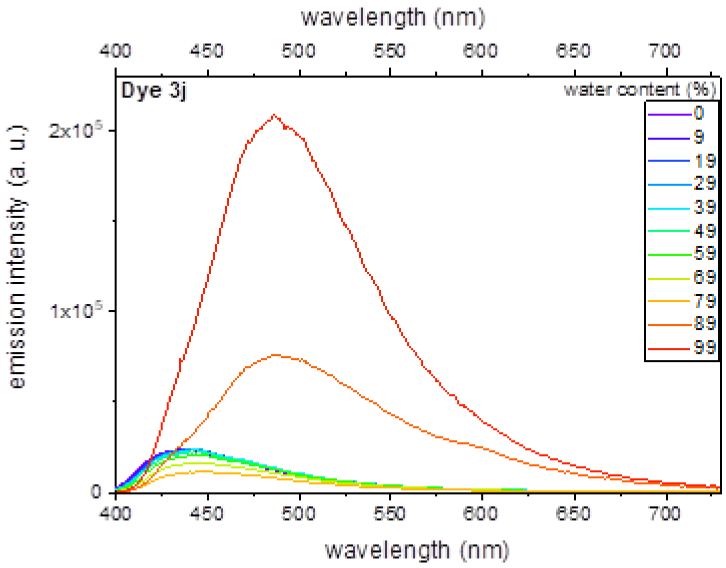

Figure S48: AIE emission spectra of compound **3j** obtained in ethanol/water mixtures of varying water content

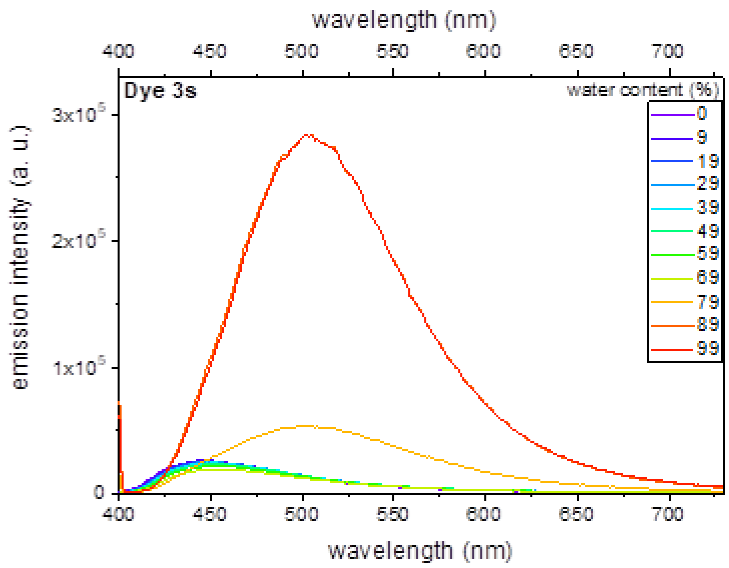

Figure S49: AIE emission spectra of compound **3s** obtained in ethanol/water mixtures of varying water content

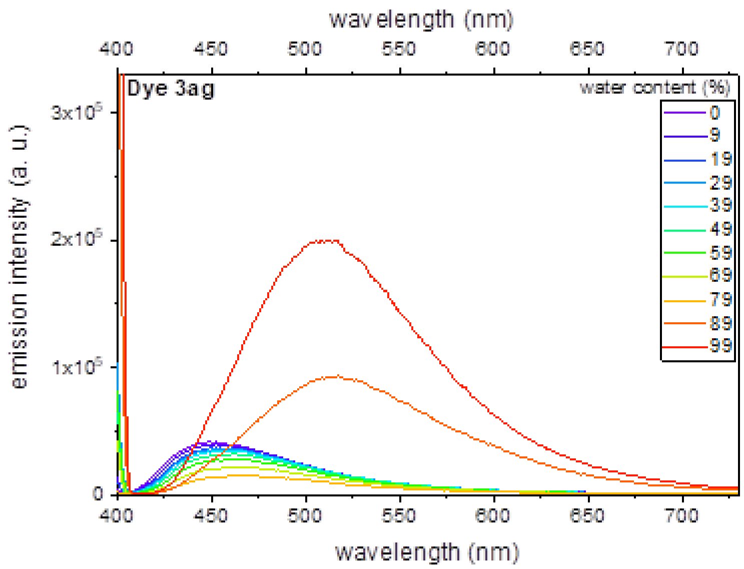

Figure S50: AIE emission spectra of compound **3ag** obtained in ethanol/water mixtures of varying water content

Table S5: Tabular overview of quantum yields and lifetimes of selected aroyl-*S,N*-ketene acetals.

Compound	Quantum yield [%]	Lifetimes [ns]
**3d**	4 ± 1	≤ 0.20 (em 437 nm)
		1.62 (em 467 nm)
		2.27 (em 494 nm)
**3j**	10 ± 1	1.53 (em 470 nm)
		2.70 (em 503 nm)
**3s**	9 ± 2	1.27 (em 469 nm)
**3ac**	8 ± 2	0.415 (em 500 nm)
**3ag**	14 ± 2	0.411 (em 513 nm)

6.1 Solvatochromism study of aroyl-*S,N*-ketene acetal 3b

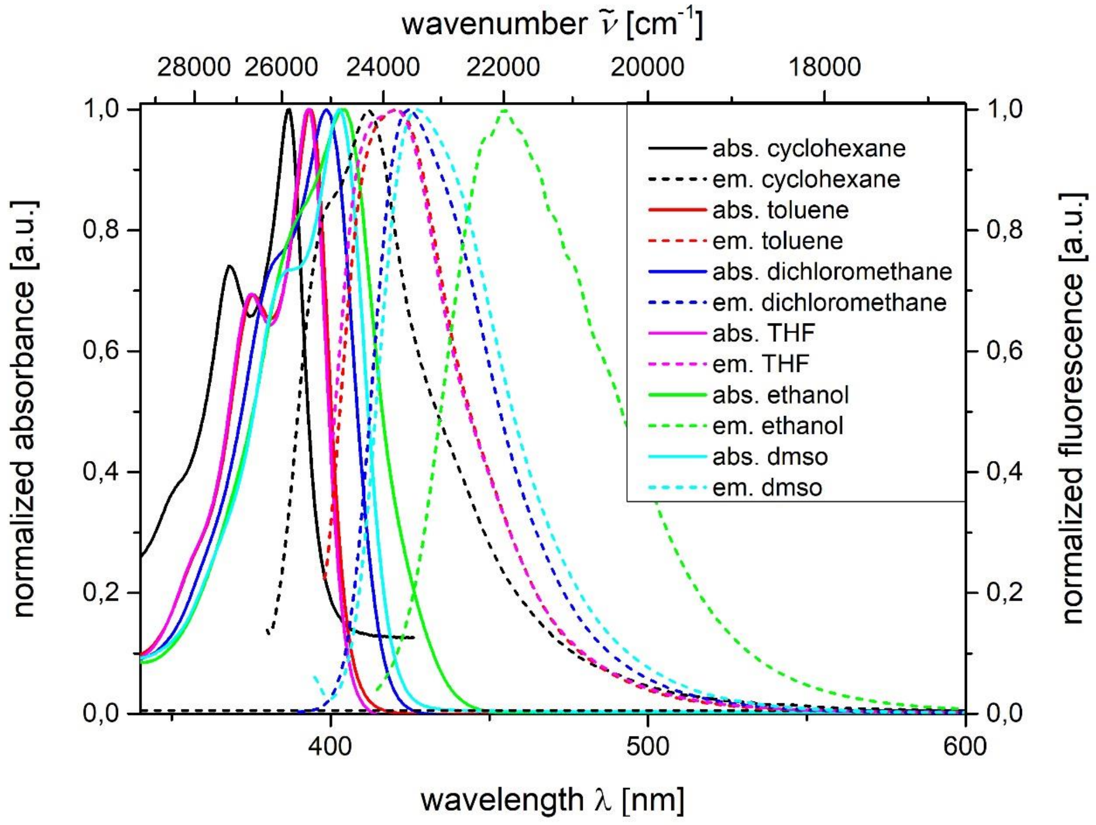

Figure S51: Normalized absorption (solid lines) and emission spectra (dashed lines) of aroyl-*S,N*-ketene acetal **3b** measured in six solvents of different polarity (recorded at T = 298 K).

Table S6: Tabular overview of utilized parameters for empiric consideration of dipole character with E_T(30) values.

Entry	Solvent	E_T(30)[7] (kcal·mol^{-1})	$\lambda_{max,abs}$[a] [nm]	$\tilde{\nu}_{max,abs}$[b] [cm^{-1}]
1	cyclohexane	30.9	387	25900
2	toluene	33.9	383	25400
3	dichloromethane	40.7	399	25100
4	THF	37.4	393	26200
5	ethanol	51.9	404	24800
6	dmso	45.1	403	24700

[a]: Recorded in the corresponding solvent, c(**3b**) = 10^{-5} M at T = 298 K, [b]: λ_{exc} = $\lambda_{max,abs}$ at T = 298 K.

λ_{max} = -51.71·E_T(30) + 27289 [cm^{-1}]

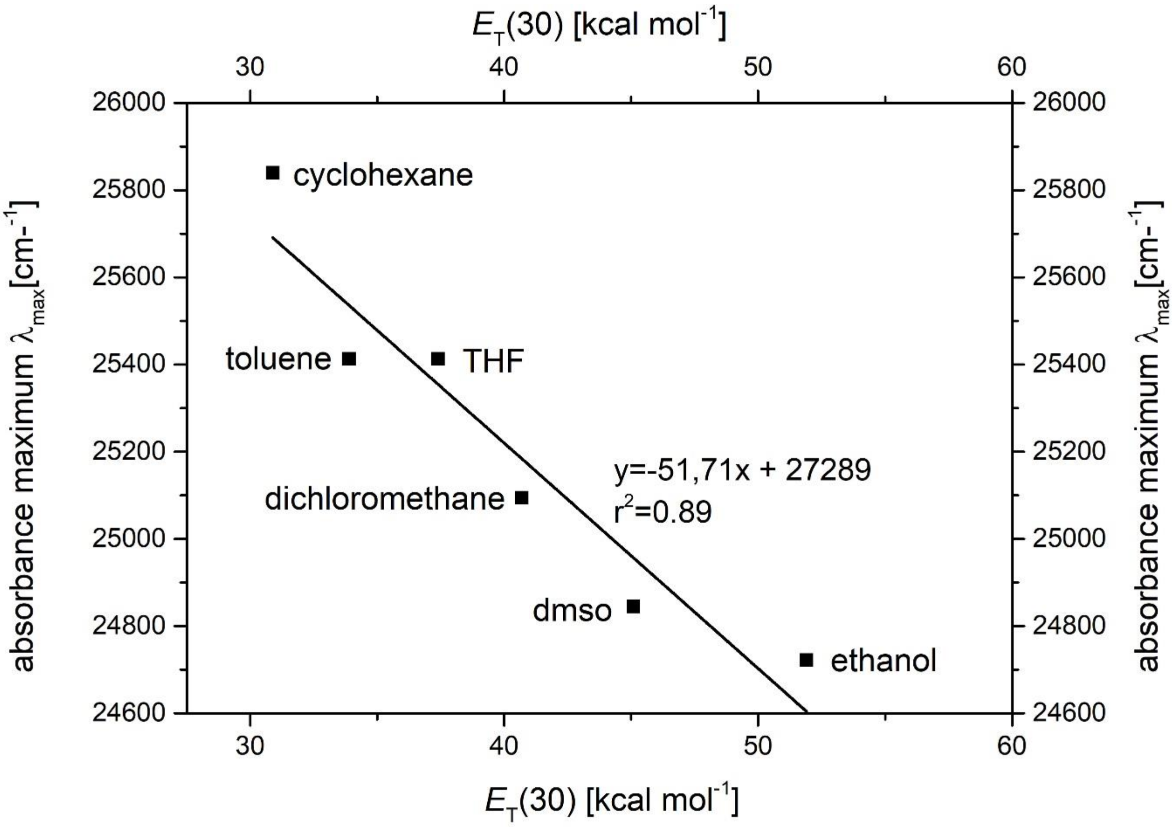

Figure S52: Empiric consideration of dipole character with E_T(30) values.

6.2 *Hammett* correlation

Table S7: Tabular overview of utilized parameters.[8]

Entry	*p*-substituents	σ_p[8]	σ_R[8]	σ_p^-[8]	σ_p^+[8]	$\lambda_{max,abs}$ [nm][a]	$\tilde{\nu}_{max,abs}$ [cm^{-1}][a]	$\lambda_{max,em}$ solid state [nm][b]	$\tilde{\nu}_{max,em}$ solid state [cm^{-1}][b]
1	OMe	-0.27	-0.16	-0.26	-0.78	384	26000	439	22600
2	^{t}Bu	-0.20	-0.16	-0.13	-0.26	383	26100	489	20400
3	H	0	0	0	0	376	26600	477	21000
4	F	0.06	-0.33	-0.03	-0.07	381	26200	441	22700
5	Cl	0.23	-0.16	0.19	0.11	392	25800	471	21200
6	Br	0.48	-0.16	0.25	0.15	388	25800	500	20000
7	I	0.18	-0.12	0.27	0.14	389	25700	472	21200
8	CF_3	0.54	0.09	0.65	0.61	391	25600	489	20400
9	CN	0.66	0.18	1	0.66	397	25200	593	16900
10	NO_2	0.78	0.16	1.27	0.79	413	24200	646	15500

[a]: Recorded in ethanol, $c = 10^{-5}$ M at T = 298 K, [b]: $\lambda_{exc} = \lambda_{max,abs}$ at T = 298 K.

Absorption

$\lambda_{max,abs} = -1431 \cdot \sigma_p + 26702$ [cm^{-1}] ($R^2 = 0.57$)

$\lambda_{max,abs} = -2557 \cdot \sigma_R + 25551$ [cm^{-1}] ($R^2 = 0.35$)

$\lambda_{max,abs} = 1187 \cdot \sigma_p^- + 26110$ [cm^{-1}] ($R^2 = 0.80$)

$\lambda_{max,abs} = -982 \cdot \sigma_p^+ + 25852$ [cm^{-1}] ($R^2 = 0.44$)

Solid state emission

$\lambda_{max,em} = -5105 \cdot \sigma_p + 21446$ [cm^{-1}] ($R^2 = 0.59$)

$\lambda_{max,em} = -11374 \cdot \sigma_R + 19439$ [cm^{-1}] ($R^2 = 0.63$)

$\lambda_{max,em} = -4226 \cdot \sigma_p^- + 21565$ [cm^{-1}] ($R^2 = 0.80$)

$\lambda_{max,em} = -3847 \cdot \sigma_p^+ + 20709$ [cm^{-1}] ($R^2 = 0.57$)

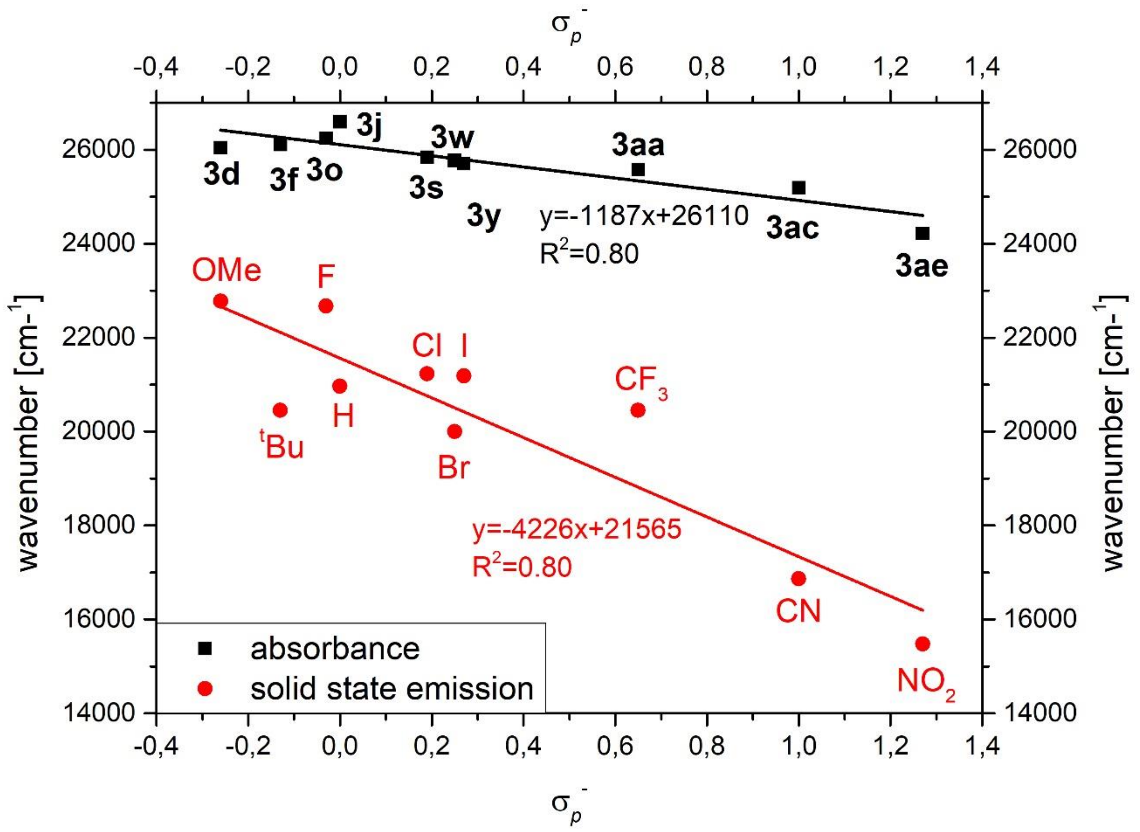

Figure S53: Linear-correlation plot of the absorption (black squares) and solid state emission maxima (red circles) [cm^{-1}] of aroyl-*S,N*-ketene acetals against the *Hammett*-parameters σ_p^-.[8]

6.3 Fluorescence excitation and emisison spectra of dye-loaded PSP

The fluorescence microscopic images of the dye-loaded PSP were recorded with an Olympus FluoView FV1000 (Olympus GmbH, Hamburg, Germany). For UV excitation, a DPSS Cobolt Zouk® (355 nm; 10 mW) was used as excitation source. The excitation light was were reflected by a dichroic mirror DM 351/488/543 and focused onto the sample through an Olympus objective UPLSAPO 60xW (numerical aperture N.A. 1.20). The emitted photons were recollected with the same objective. Emission signals were detected in a wavelength range between 400 nm and 475 nm.

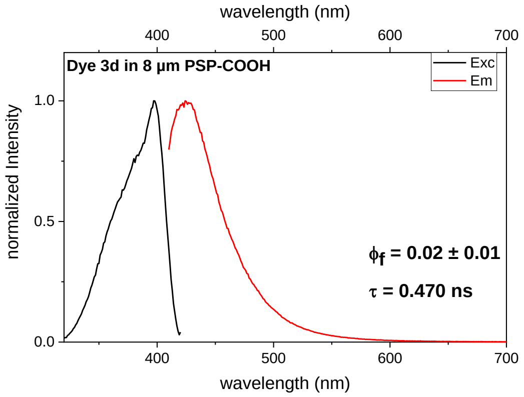

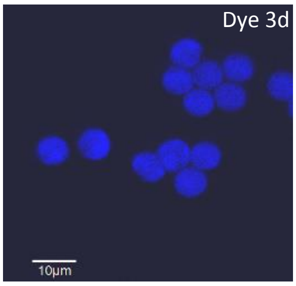

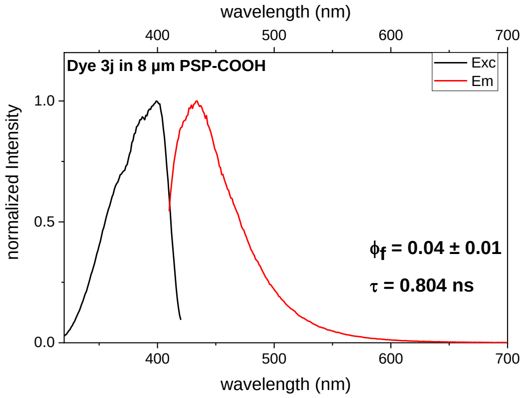

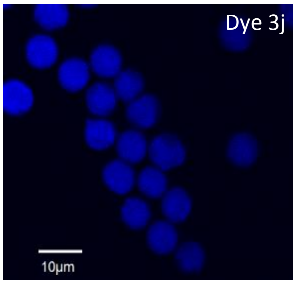

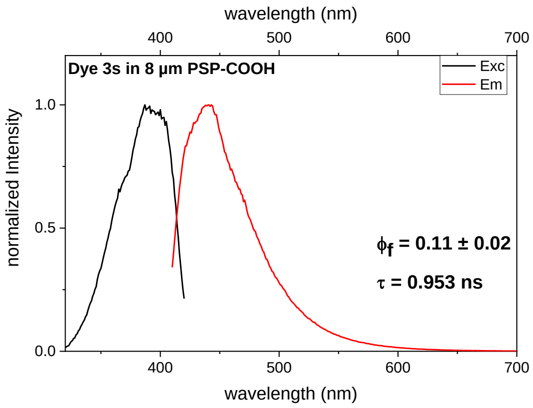

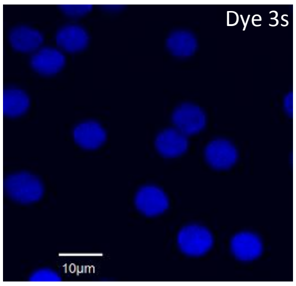

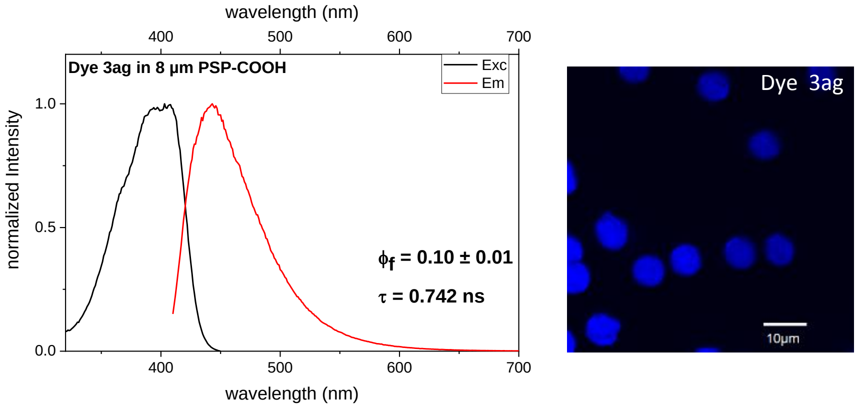

Figure S54: Normalized excitation and emission spectra (λ_{exc} = 395 nm) of dispersed 8 µm-sized PSP loaded with dyes (left) and CLSM image of the dye-loaded PSP (right).

Table S8: Tabular overview of quantum yields and lifetimes of selected aroyl-*S,N*-ketene acetals of dye-loaded PSP.

Compound	Quantum yield [%]	Lifetimes [ns]
**3d**	2 ± 1	0.470
**3j**	4 ± 1	0.804
**3s**	11 ± 2	0.953
**3ac**	35 ± 2	2.15
**3ag**	10 ± 1	0.742

7 DFT calculations

Table S9: TD-DFT calculations (B3LYP/6-31G**) of the UV/Vis absorption maxima of selected examples **3** applying PCM with ethanol as the solvent using Gaussian 09.[9]

Compound	Substituent R^1	Substituent R^2	$\lambda_{max(Abs.)}$ [nm][(a)] (ε [L·mol^{-1}·cm^{-1}])	$\lambda_{max(calcd.)}$ [nm]	Most dominant contributions	Oscillator strength
**3b**	4-Me_2N-C_6H_4	$C_6H_5CH_2$	404 (60300)	382	HOMO→LUMO	1.1951
**3d**	4-MeO-C_6H_4	$C_6H_5CH_2$	384 (43500)	359	HOMO→LUMO	0.8866
**3f**	4-^{t}Bu-C_6H_4	$C_6H_5CH_2$	383 (42700)	369	HOMO→LUMO	0.7620
**3h**	4-Me-C_6H_4	$C_6H_5CH_2$	382 (40000)	365	HOMO→LUMO	0.7839
**3j**	C_6H_5	$C_6H_5CH_2$	376 (39100)	369	HOMO→LUMO	0.7006
**3k**	C_6H_5	CH_3	382 (41400)	367	HOMO→LUMO	0.7476
**3l**	4-F-$C_6H_4C_2H_2$	$C_6H_5CH_2$	411 (11000)	423	HOMO→LUMO	0.5175
**3o**	4-F-C_6H_4	$C_6H_5CH_2$	381 (47200)	364	HOMO→LUMO	0.7305
**3p**	3-F-C_6H_4	$C_6H_5CH_2$	387 (74700)	380	HOMO→LUMO	0.7850
**3q**	2-F-C_6H_4	$C_6H_5CH_2$	383 (85400)	367	HOMO→LUMO	0.6210
**3r**	4-Cl-C_6H_4	$C_6H_4CH_2$	387 (39000)	372	HOMO→LUMO	0.8802
**3u**	4-Cl-C_5H_3N	$C_6H_5CH_2$	392 (34800)	381	HOMO→LUMO	0.8197
**3z**	4-F_3C-C_6H_4	4-Br-$C_6H_4CH_2$	390 (36600)	372	HOMO→LUMO	0.6736
**3ac**	4-NC-C_6H_4	$C_6H_5CH_2$	397 (43600)	402	HOMO→LUMO	0.5639
**3ad**	4-O_2N-C_6H_4	4-Br-$C_6H_4CH_2$	413 (22000)	409	HOMO→LUMO+1	0.3127
**3ag**	C_4H_4S	$C_6H_5CH_2$	392 (39200)	384	HOMO→LUMO	0.5931
**3ai**	C_4H_4O	$C_6H_5CH_2$	399 (43900)	419	HOMO→LUMO	0.6334

(a): Recorded in ethanol, T = 298 K, $c = 10^{-5}$ M.

The ground state geometries of the aroyl-*S,N*-ketene acetals **5** were optimized in a DFT calculation with the B3LYP functional and the 6-31G** basis set in the program package Gaussian09. The minima structures were confirmed by analytical frequency analysis.

7.1 Computed xyz-coordinates of selected aroyl-*S,N*-ketene acetals 3

(*Z*)-2-(3-Benzyl)benzo[*d*]thiazol-2(3*H*)-ylidene)-1-(4-((dimethylamino)phenyl)ethan-1-one (3b) (B3LYP)

C 0	-5.768762	2.201279	-1.145588
C 0	-6.236039	2.839600	0.003188
C 0	-6.507961	2.096765	1.152061
C 0	-6.312708	0.715687	1.152040
C 0	-5.845530	0.077356	0.003258
C 0	-5.573509	0.820201	-1.145609
C 0	-5.656488	-1.260353	0.003258
C 0	-4.403477	-1.765492	0.003258
O 0	-6.607838	-2.004797	0.003258
C 0	-3.350534	-0.941550	0.003258
N 0	-2.146497	-1.332766	0.003258
C 0	-1.349777	-0.348899	0.003258
C 0	-2.018277	0.808977	0.003258
S 0	-3.459314	0.580739	0.003258
C 0	-0.012835	-0.348899	-0.009224
C 0	0.655623	0.808976	-0.016816
C 0	-0.012864	1.966735	-0.021705
C 0	-1.349806	1.966802	-0.009224
C 0	-1.728995	-2.742231	0.003258
C 0	-2.940093	-3.622145	0.003258
C 0	-3.284872	-4.334040	1.152093
C 0	-4.413282	-5.154006	1.152017
C 0	-5.196880	-5.261923	0.003178
C 0	-4.852168	-4.549970	-1.145612
C 0	-3.723691	-3.730062	-1.145581
N 0	-6.413356	4.093121	0.003145
C 0	-6.905825	4.765817	1.213870
C 0	-6.126776	4.875934	-1.207629
H 0	-5.554223	2.787069	-2.051581
H 0	-6.876386	2.600132	2.058088
H 0	-6.527176	0.129910	2.058058
H 0	-5.205013	0.316839	-2.051610
H 0	-4.249557	-2.854670	0.003258
H 0	0.537151	-1.301527	-0.013246
H 0	1.755621	0.809001	-0.019039
H 0	0.537066	2.919296	-0.035996
H 0	-1.899782	2.919444	-0.009224
H 0	-1.121173	-2.948559	-0.906001
H 0	-1.120572	-2.948763	0.912067
H 0	-2.666937	-4.248843	2.058127
H 0	-4.685180	-5.715531	2.057976
H 0	-6.086755	-5.908546	0.003159
H 0	-5.470195	-4.635057	-2.051594
H 0	-3.451755	-3.168599	-2.051566
H 0	-6.997042	5.858165	1.020931
H 0	-7.902778	4.353395	1.487297
H 0	-6.190878	4.595303	2.049659

H 0	-6.342114	5.950737	-1.014766
H 0	-5.054546	4.756190	-1.481038
H 0	-6.766371	4.513756	-2.043401

SCF Done: E(RB3LYP) = -1510.38732405 A.U. after 15 cycles

Sum of electronic and zero-point Energies= -1510.632729

Sum of electronic and thermal Energies= -1510.608742

Sum of electronic and thermal Enthalpies= -1510.607798

Sum of electronic and thermal Free Energies= -1510.689656

(*Z*)-2-(3-Benzyl)benzo[*d*]thiazol-2(3*H*)-ylidene)-1-(4-(methoxyphenyl)ethan-1-one (3d) (B3LYP)

C 0	-1.808463	-2.148596	5.977679
C 0	-2.054735	-1.316852	7.069943
C 0	-2.446615	0.006019	6.864770
C 0	-2.592073	0.497076	5.567385
C 0	-2.345713	-0.334619	4.475133
C 0	-1.953921	-1.657539	4.680293
C 0	-2.486613	0.141052	3.218517
C 0	-2.247980	-0.664560	2.160573
O 0	-2.825974	1.286713	3.040873
C 0	-2.387420	-0.193818	0.916978
N 0	-2.183761	-0.881360	-0.126365
C 0	-2.387420	-0.193819	-1.169709
C 0	-2.756657	1.052703	-0.857593
S 0	-2.810744	1.235298	0.588924
C 0	-2.291543	-0.561441	-2.451595
C 0	-2.560213	0.318844	-3.421381
C 0	-2.934105	1.563867	-3.109244
C 0	-3.030001	1.931549	-1.827377
C 0	-1.766258	-2.290825	-0.126365
C 0	-2.977357	-3.170740	-0.126365
C 0	-3.322136	-3.882635	1.022471
C 0	-4.450546	-4.702601	1.022395
C 0	-5.234144	-4.810518	-0.126444
C 0	-4.889432	-4.098564	-1.275234
C 0	-3.760954	-3.278657	-1.275203
O 0	-1.913307	-1.793887	8.330285
C 0	-2.207540	-0.800284	9.274629
H 0	-1.499515	-3.191852	6.139448
H 0	-2.640940	0.661979	7.726137
H 0	-2.901093	1.540310	5.405620
H 0	-1.759670	-2.313517	3.818923
H 0	-1.938960	-1.707795	2.322335
H 0	-1.991616	-1.588139	-2.708372
H 0	-2.473621	0.018685	-4.476088
H 0	-3.162850	2.285774	-3.907070
H 0	-3.333788	2.957116	-1.570614
H 0	-1.158436	-2.497153	-1.035623
H 0	-1.157835	-2.497357	0.782445
H 0	-2.704201	-3.797437	1.928504

H 0	-4.722444	-5.264125	1.928354
H 0	-6.124019	-5.457141	-0.126464
H 0	-5.507459	-4.183651	-2.181216
H 0	-3.489019	-2.717193	-2.181189
H 0	-2.084731	-1.214541	10.300338
H 0	-1.513790	0.059040	9.136633
H 0	-3.257163	-0.456734	9.136665

SCF Done: E(RB3LYP) = -1491.09698957 A.U. after 14 cycles

Sum of electronic and zero-point Energies= -1491.190182

Sum of electronic and thermal Energies= -1491.167167

Sum of electronic and thermal Enthalpies= -1491.166223

Sum of electronic and thermal Free Energies= -1491.246062

(*Z*)-2-(3-Benzyl)benzo[*d*]thiazol-2(3*H*)-ylidene)-1-(4-*tert*-butylphenyl)ethan-1-one (3f) (B3LYP)

C 0	-4.836857	2.999794	-1.145075
C 0	-5.304122	3.638123	0.003703
C 0	-5.576031	2.895298	1.152584
C 0	-5.380777	1.514220	1.152572
C 0	-4.913612	0.875880	0.003789
C 0	-4.641604	1.618715	-1.145087
C 0	-4.724570	-0.461829	0.003798
C 0	-4.272069	-1.080101	-1.108939
O 0	-4.960143	-1.105115	0.998761
C 0	-4.084986	-2.403948	-1.108931
N 0	-3.664743	-3.058731	-2.107636
C 0	-3.578969	-4.292635	-1.837677
C 0	-3.957844	-4.535111	-0.578618
S 0	-4.353027	-3.302749	0.095025
C 0	-3.167813	-5.297895	-2.617402
C 0	-3.140132	-6.546294	-2.139591
C 0	-3.514381	-6.788053	-0.879117
C 0	-3.925547	-5.782836	-0.099340
C 0	-3.321912	-2.465128	-3.408021
C 0	-4.534239	-2.451119	-4.286130
C 0	-4.621007 -	3.325749 -	5.369174
C 0	-5.750586	-3.312582	-6.187423
C 0	-6.793340	-2.424897	-5.922508
C 0	-6.706624	-1.550345	-4.839429
C 0	-5.576993	-1.563434	-4.021216
C 0	-5.488021	4.938181	0.003650
C 0	-4.844947	5.404404	0.783305
C 0	-6.554087	5.162616	0.231467
C 0	-5.209729	5.352732	-0.991071
H 0	-4.622329	3.585577	-2.051075
H 0	-5.944444	3.398672	2.058612
H 0	-5.595235	0.928450	2.058597
H 0	-4.273119	1.115346	-2.051088
H 0	-4.057558	-0.494328	-2.014949
H 0	-2.852314	-5.097856	-3.652025
H 0	-2.809425	-7.374480	-2.783594

H 0	-3.484031	-7.814018	-0.483546
H 0	-4.237256	-5.982357	0.936531
H 0	-2.962374	-1.422797	-3.256211
H 0	-2.520922	-3.068136	-3.891309
H 0	-3.798681	-4.025867	-5.578018
H 0	-5.819024	-4.002262	-7.041623
H 0	-7.684127	-2.414554	-6.567792
H 0	-7.529020	-0.850335	-4.630503
H 0	-5.508527	-0.873699	-3.167062
H 0	-5.000834	6.506433	0.783260
H 0	-3.775871	5.189656	0.560303
H 0	-5.120230	4.999540	1.782842
H 0	-6.709974	6.264645	0.231422
H 0	-6.811877	4.760227	1.236652
H 0	-7.204708	4.704586	-0.546781
H 0	-5.365616	6.454761	-0.991116
H 0	-5.852046	4.895877	-1.776870
H 0	-4.136920	5.138513	-1.195913

SCF Done: E(RB3LYP) = -1533.09580952 A.U. after 14 cycles

Sum of electronic and zero-point Energies= -1533.882902

Sum of electronic and thermal Energies= -1533.857986

Sum of electronic and thermal Enthalpies= -1533.857041

Sum of electronic and thermal Free Energies= -1533.939651

(*Z*)-2-(3-Benzyl)benzo[*d*]thiazol-2(3*H*)-ylidene)-1-(*p*-tolyl)ethan-1-one (3h) (B3LYP)

C 0	-4.836857	2.999794	-1.145075
C 0	-5.304122	3.638123	0.003703
C 0	-5.576031	2.895298	1.152584
C 0	-5.380777	1.514220	1.152572
C 0	-4.913612	0.875880	0.003789
C 0	-4.641604	1.618715	-1.145087
C 0	-4.724570	-0.461829	0.003798
C 0	-4.272069	-1.080101	-1.108939
O 0	-4.960143	-1.105115	0.998761
C 0	-4.084986	-2.403948	-1.108931
N 0	-3.664743	-3.058731	-2.107636
C 0	-3.578969	-4.292635	-1.837677
C 0	-3.957844	-4.535111	-0.578618
S 0	-4.353027	-3.302749	0.095025
C 0	-3.167813	-5.297895	-2.617402
C 0	-3.140132	-6.546294	-2.139591
C 0	-3.514381	-6.788053	-0.879117
C 0	-3.925547	-5.782836	-0.099340
C 0	-3.321912	-2.465128	-3.408021
C 0	-4.534239	-2.451119	-4.286130
C 0	-4.621007	-3.325749	-5.369174
C 0	-5.750586	-3.312582	-6.187423
C 0	-6.793340	-2.424897	-5.922508
C 0	-6.706624	-1.550345	-4.839429
C 0	-5.576993	-1.563434	-4.021216
C 0	-5.488021	4.938181	0.003650

H 0	-4.622329	3.585577	-2.051075
H 0	-5.944444	3.398672	2.058612
H 0	-5.595235	0.928450	2.058597
H 0	-4.273119	1.115346	-2.051088
H 0	-4.057558	-0.494328	-2.014949
H 0	-2.852314	-5.097856	-3.652025
H 0	-2.809425	-7.374480	-2.783594
H 0	-3.484031	-7.814018	-0.483546
H 0	-4.237256	-5.982357	0.936531
H 0	-2.962374	-1.422797	-3.256211
H 0	-2.520922	-3.068136	-3.891309
H 0	-3.798681	-4.025867	-5.578018
H 0	-5.819024	-4.002262	-7.041623
H 0	-7.684127	-2.414554	-6.567792
H 0	-7.529020	-0.850335	-4.630503
H 0	-5.508527	-0.873699	-3.167062
H 0	-4.844947	5.404404	0.783305
H 0	-6.554087	5.162616	0.231467
H 0	-5.209729	5.352732	-0.991071

SCF Done: E(RB3LYP) = -1415.86741677   A.U. after   14 cycles

Sum of electronic and zero-point Energies=   -1415.999159

Sum of electronic and thermal Energies=   -1415.977050

Sum of electronic and thermal Enthalpies=   -1415.976106

Sum of electronic and thermal Free Energies=   -1416.054695

(*Z*)-2-(3-Benzylbenzo[*d*]thiazol-2(3*H*)-ylidene)-1-phenylethan-1-one (3j) (B3LYP)

C 0	-1.932246	-1.653985	-6.104507
C 0	-2.178695	-0.822294	-7.196771
C 0	-2.570447	0.500615	-6.991600
C 0	-2.715837	0.991693	-5.694215
C 0	-2.469488	0.159996	-4.601962
C 0	-2.077636	-1.162908	-4.807121
C 0	-2.610388	0.635668	-3.345347
C 0	-2.371764	-0.169945	-2.287402
O 0	-2.949741	1.781332	-3.167704
C 0	-2.511204	0.300797	-1.043808
N 0	-2.307553	-0.386747	-0.000463
C 0	-2.511210	0.300795	1.042880
C 0	-2.880436	1.547321	0.730763
S 0	-2.934518	1.729917	-0.715755
C 0	-2.391403	-0.059737	2.324766
C 0	-2.645511	0.824862	3.294551
C 0	-3.010018	2.072664	2.982413
C 0	-3.129841	2.433259	1.700546
C 0	-1.890060	-1.796214	-0.000463
C 0	-3.101165	-2.676121	-0.000467
C 0	-3.445952	-3.388015	1.148367
C 0	-4.574368	-4.207973	1.148287
C 0	-5.357963	-4.315884	-0.000554
C 0	-5.013244	-3.603931	-1.149342
C 0	-3.884760	-2.784032	-1.149308

H 0	-1.623214	-2.697215	-6.266276
H 0	-2.064121	-1.209626	-8.219927
H 0	-3.024797	2.034945	-5.532450
H 0	-1.883285	-1.818855	-3.945751
H 0	-2.062751	-1.213182	-2.449163
H 0	-2.083772	-1.084152	2.581543
H 0	-2.554661	0.525966	4.349258
H 0	-3.211353	2.802691	3.780239
H 0	-3.433619	3.458827	1.443784
H 0	-1.282237	-2.002546	-0.909721
H 0	-1.281641	-2.002751	0.908347
H 0	-2.828019	-3.302822	2.054401
H 0	-4.846272	-4.769496	2.054245
H 0	-6.247843	-4.962501	-0.000577
H 0	-5.631269	-3.689013	-2.055326
H 0	-3.612819	-2.222569	-2.055293
H 0	-2.803582	1.287784	-8.025240

SCF Done: E(RB3LYP) = -1376.55729253   A.U. after   14 cycles

Sum of electronic and zero-point Energies=   -1376.698113

Sum of electronic and thermal Energies=   -1376.677886

Sum of electronic and thermal Enthalpies=   -1376.676942

Sum of electronic and thermal Free Energies=   -1376.750958

(*Z*)-2-(3-Methyl)benzo[*d*]thiazol-2(3*H*)-ylidene)-1-phenylethan-1-one (3k) (B3LYP)

C 0	-4.836857	2.999794	-1.145075
C 0	-5.304122	3.638123	0.003703
C 0	-5.576031	2.895298	1.152584
C 0	-5.380777	1.514220	1.152572
C 0	-4.913612	0.875880	0.003789
C 0	-4.641604	1.618715	-1.145087
C 0	-4.724570	-0.461829	0.003798
C 0	-4.272069	-1.080101	-1.108939
O 0	-4.960143	-1.105115	0.998761
C 0	-4.084986	-2.403948	-1.108931
N 0	-3.664743	-3.058731	-2.107636
C 0	-3.578969	-4.292635	-1.837677
C 0	-3.957844	-4.535111	-0.578618
S 0	-4.353027	-3.302749	0.095025
C 0	-3.167813	-5.297895	-2.617402
C 0	-3.140132	-6.546294	-2.139591
C 0	-3.514381	-6.788053	-0.879117
C 0	-3.925547	-5.782836	-0.099340
C 0	-3.321912	-2.465128	-3.408021
H 0	-4.622329	3.585577	-2.051075
H 0	-5.488021	4.938181	0.003650
H 0	-5.944444	3.398672	2.058612
H 0	-5.595235	0.928450	2.058597
H 0	-4.273119	1.115346	-2.051088
H 0	-4.057558	-0.494328	-2.014949
H 0	-2.852314	-5.097856	-3.652025
H 0	-2.809425	-7.374480	-2.783594

H 0	-3.484031	-7.814018	-0.483546
H 0	-4.237256	-5.982357	0.936531
H 0	-4.230070	-2.456414	-4.066851
H 0	-2.962224	-1.422687	-3.257324
H 0	-2.521368	-3.068036	-3.892171

SCF Done: E(RB3LYP) = -1145.50751868 A.U. after 14 cycles

Sum of electronic and zero-point Energies= -1145.674652

Sum of electronic and thermal Energies= -1145.658701

Sum of electronic and thermal Enthalpies= -1145.657757

Sum of electronic and thermal Free Energies= -1145.719770

(1*Z*)(3*E*)-2-(3-Benzyl)benzo[*d*]thiazol-2(3*H*)-ylidene)-1-(4-fluorphenyl)but-3-en-2-one (3m) (B3LYP)

C 0	-3.435462	-2.451273	0.003565
C 0	-4.729112	-1.929777	0.003661
C 0	-4.924243	-0.548626	0.003565
C 0	-3.825742	0.310908	0.003494
C 0	-2.532110	-0.210570	0.003494
C 0	-2.336962	-1.591739	0.003494
C 0	-1.479167	0.613371	0.003494
C 0	-0.239141	0.113467	0.003494
C 0	0.824827	0.946036	0.003494
C 0	2.077838	0.440897	0.003494
O 0	0.655795	2.142152	0.003494
C 0	3.130781	1.264839	0.003494
N 0	4.334818	0.873623	0.003494
C 0	5.131538	1.857490	0.003494
C 0	4.463038	3.015366	0.003494
S 0	3.022001	2.787128	0.003494
C 0	6.468480	1.857490	-0.008988
C 0	7.136938	3.015365	-0.016580
C 0	6.468451	4.173124	-0.021469
C 0	5.131509	4.173191	-0.008988
C 0	4.752321	-0.535842	0.003494
C 0	3.541222	-1.415756	0.003494
C 0	3.196443	-2.127651	1.152329
C 0	2.068033	-2.947617	1.152253
C 0	1.284435	-3.055534	0.003415
C 0	1.629147	-2.343581	-1.145376
C 0	2.757625	-1.523673	-1.145345
F 0	-5.768675	-2.743232	0.003844
H 0	-3.281550	-3.540452	0.003545
H 0	-5.944444	-0.137301	0.003545
H 0	-3.979658	1.400086	0.003438
H 0	-1.316758	-2.003059	0.003438
H 0	-1.633088	1.702549	0.003494
H 0	-0.085221	-0.975711	0.003494
H 0	2.231758	-0.648281	0.003494
H 0	7.018466	0.904862	-0.013010
H 0	8.236936	3.015390	-0.018803

H 0	7.018381	5.125685	-0.035760
H 0	4.581533	5.125833	-0.008988
H 0	5.360142	-0.742170	-0.905764
H 0	5.360744	-0.742374	0.912304
H 0	3.814378	-2.042454	2.058363
H 0	1.796135	-3.509142	2.058212
H 0	0.394560	-3.702158	0.003395
H 0	1.011120	-2.428668	-2.051358
H 0	3.029560	-0.962210	-2.051330

SCF Done: E(RB3LYP) = -1553.10254159 A.U. after 15 cycles

Sum of electronic and zero-point Energies= -1553.363558

Sum of electronic and thermal Energies= -1553.340238

Sum of electronic and thermal Enthalpies= -1553.339294

Sum of electronic and thermal Free Energies= -1553.420868

(*Z*)-2-(3-Benzyl)benzo[*d*]thiazol-2(3*H*)-ylidene)-1-(4-fluor)phenylethan-1-one (3o) (B3LYP)

C 0	-4.836857	2.999794	-1.145075
C 0	-5.304122	3.638123	0.003703
C 0	-5.576031	2.895298	1.152584
C 0	-5.380777	1.514220	1.152572
C 0	-4.913612	0.875880	0.003789
C 0	-4.641604	1.618715	-1.145087
C 0	-4.724570	-0.461829	0.003798
C 0	-4.272069	-1.080101	-1.108939
O 0	-4.960143	-1.105115	0.998761
C 0	-4.084986	-2.403948	-1.108931
N 0	-3.664743	-3.058731	-2.107636
C 0	-3.578969	-4.292635	-1.837677
C 0	-3.957844	-4.535111	-0.578618
S 0	-4.353027	-3.302749	0.095025
C 0	-3.167813	-5.297895	-2.617402
C 0	-3.140132	-6.546294	-2.139591
C 0	-3.514381	-6.788053	-0.879117
C 0	-3.925547	-5.782836	-0.099340
C 0	-3.321912	-2.465128	-3.408021
C 0	-4.534239	-2.451119	-4.286130
C 0	-4.621007	-3.325749	-5.369174
C 0	-5.750586	-3.312582	-6.187423
C 0	-6.793340	-2.424897	-5.922508
C 0	-6.706624	-1.550345	-4.839429
C 0	-5.576993	-1.563434	-4.021216
F 0	-5.488021	4.938181	0.003650
H 0	-4.613366	3.586823	-2.048097
H 0	-5.953403	3.397385	2.055634
H 0	-5.604196	0.927204	2.055620
H 0	-4.264162	1.116633	-2.048110
H 0	-4.048595	-0.493082	-2.011971
H 0	-2.843380	-5.096522	-3.648999
H 0	-2.800466	-7.373134	- 2.780654

H 0	-3.475074	-7.812586	-0.480634
H 0	-4.246196	-5.983717	0.933535
H 0	-2.962374	- 1.422797	-3.256211
H 0	-2.520922	- 3.068136	-3.891309
H 0	-3.802838	-4.032356	-5.572418
H 0	-5.823148	-4.008752	-7.036000
H 0	-7.688220	-2.421070	-6.562177
H 0	-7.533115	-0.856878	-4.624919
H 0	-5.504404	-0.867209	- 3.172686

SCF Done: E(RB3LYP) = -1475.81110751   A.U. after   14 cycles

Sum of electronic and zero-point Energies=   -1475.971132

Sum of electronic and thermal Energies=   -1475.950043

Sum of electronic and thermal Enthalpies=   -1475.949099

Sum of electronic and thermal Free Energies=   -1476.025586

(*Z*)-2-(3-Benzyl)benzo[*d*]thiazol-2(3*H*)-ylidene)-1-(3-fluor)phenylethan-1-one (3p) (B3LYP)

C 0	-5.9298	3.1473	-1.14931
C 0	-6.39708	3.78562	-0.00053
C 0	-6.669	3.04279	1.14834
C 0	-6.47375	1.66171	1.14832
C 0	-6.00657	1.02338	-0.00046
C 0	-5.73455	1.76622	-1.14933
F 0	-7.11111	3.64683	2.23557
C 0	-5.81753	-0.31433	-0.00046
C 0	-4.56452	-0.81947	-0.00046
O 0	-6.76888	-1.05877	-0.00046
C 0	-3.51158	0.00447	-0.00046
N 0	-2.30754	-0.38674	-0.00046
C 0	-1.51082	0.59712	-0.00046
C 0	-2.17932	1.755	-0.00046
S 0	-3.62035	1.52676	-0.00046
C 0	-0.17388	0.59712	-0.01295
C 0	0.49458	1.755	-0.02054
C 0	-0.17391	2.91276	-0.02543
C 0	-1.51085	2.91283	-0.01295
C 0	-1.89004	-1.79621	-0.00046
C 0	-3.10113	-2.67612	-0.00046
C 0	-3.44591	-3.38802	1.14837
C 0	-4.57432	-4.20798	1.1483
C 0	-5.35792	-4.3159	-0.00054
C 0	-5.01321	-3.60395	-1.14933
C 0	-3.88473	-2.78404	-1.1493
H 0	-5.71526	3.73309	-2.0553
H 0	-6.55115	4.87478	-0.00057
H 0	-6.68822	1.07593	2.05434
H 0	-5.36605	1.26286	-2.05533
H 0	-4.4106	-1.90865	-0.00046
H 0	0.37611	-0.3555	-0.01697
H 0	1.59458	1.75503	-0.02276

H 0	0.37603	3.86532	-0.03972
H 0	-2.06082	3.86547	-0.01295
H 0	-1.28221	-2.00253	-0.90972
H 0	-1.28161	-2.00274	0.90835
H 0	-2.82798	-3.30282	2.05441
H 0	-4.84622	-4.76951	2.05425
H 0	-6.2478	-4.96252	-0.00056
H 0	-5.63124	-3.68903	-2.05532
H 0	-3.6128	-2.22257	-2.05529

SCF Done: E(RB3LYP) = -1475.65995693 A.U. after 15 cycles

Sum of electronic and zero-point Energies= -1475.970928

Sum of electronic and thermal Energies= -1475.949881

Sum of electronic and thermal Enthalpies= -1475.948937

Sum of electronic and thermal Free Energies= -1476.024575

(*Z*)-2-(3-Benzyl)benzo[*d*]thiazol-2(3*H*)-ylidene)-1-(2-fluor)phenylethan-1-one (3q) (B3LYP)

C 0	-1.932246	-1.653985	-6.104507
C 0	-2.178695	-0.822294	-7.196771
C 0	-2.570447	0.500615	-6.991600
C 0	-2.715837	0.991693	-5.694215
C 0	-2.469488	0.159996	-4.601962
C 0	-2.077636	-1.162908	-4.807121
C 0	-2.610388	0.635668	-3.345347
C 0	-2.371764	-0.169945	-2.287402
O 0	-2.949741	1.781332	-3.167704
C 0	-2.511204	0.300797	-1.043808
N 0	-2.307553	-0.386747	-0.000463
C 0	-2.511210	0.300795	1.042880
C 0	-2.880436	1.547321	0.730763
S 0	-2.934518	1.729917	-0.715755
C 0	-2.391403	-0.059737	2.324766
C 0	-2.645511	0.824862	3.294551
C 0	-3.010018	2.072664	2.982413
C 0	-3.129841	2.433259	1.700546
C 0	-1.890060	-1.796214	-0.000463
C 0	-3.101165	-2.676121	-0.000467
C 0	-3.445952	-3.388015	1.148367
C 0	-4.574368	-4.207973	1.148287
C 0	-5.357963	-4.315884	-0.000554
C 0	-5.013244	-3.603931	-1.149342
C 0	-3.884760	-2.784032	-1.149308
F 0	-3.024797	2.034945	-5.532450
H 0	-1.623214	-2.697215	-6.266276
H 0	-2.064121	-1.209626	-8.219927
H 0	-2.803582	1.287784	-8.025240
H 0	-1.883285	-1.818855	-3.945751
H 0	-2.062751	-1.213182	-2.449163
H 0	-2.083772	-1.084152	2.581543
H 0	-2.554661	0.525966	4.349258

H 0	-3.211353	2.802691	3.780239
H 0	-3.433619	3.458827	1.443784
H 0	-1.282237	-2.002546	-0.909721
H 0	-1.281641	-2.002751	0.908347
H 0	-2.828019	-3.302822	2.054401
H 0	-4.846272	-4.769496	2.054245
H 0	-6.247843	-4.962501	-0.000577
H 0	-5.631269	-3.689013	-2.055326
H 0	-3.612819	-2.222569	-2.055293

SCF Done: E(RB3LYP) = -1475.98235589 A.U. after 14 cycles

Sum of electronic and zero-point Energies= -1475.939580

Sum of electronic and thermal Energies= -1475.918334

Sum of electronic and thermal Enthalpies= -1475.917389

Sum of electronic and thermal Free Energies= -1475.993014

(*Z*)-2-(3-Benzyl)benzo[*d*]thiazol-2(3*H*)-ylidene)-1-(4-chlor)phenylethan-1-one (3s)

C 0	-4.836857	2.999794	-1.145075
C 0	-5.304122	3.638123	0.003703
C 0	-5.576031	2.895298	1.152584
C 0	-5.380777	1.514220	1.152572
C 0	-4.913612	0.875880	0.003789
C 0	-4.641604	1.618715	-1.145087
C 0	-4.724570	-0.461829	0.003798
C 0	-4.272069	-1.080101	-1.108939
O 0	-4.960143	-1.105115	0.998761
C 0	-4.084986	-2.403948	-1.108931
N 0	-3.664743	-3.058731	-2.107636
C 0	-3.578969	-4.292635	-1.837677
C 0	-3.957844	-4.535111	-0.578618
S 0	-4.353027	-3.302749	0.095025
C 0	-3.167813	-5.297895	-2.617402
C 0	-3.140132	-6.546294	-2.139591
C 0	-3.514381	-6.788053	-0.879117
C 0	-3.925547	-5.782836	-0.099340
C 0	-3.321912	-2.465128	-3.408021
C 0	-4.534239	-2.451119	-4.286130
C 0	-4.621007	-3.325749	-5.369174
C 0	-5.750586	-3.312582	-6.187423
C 0	-6.793340	-2.424897	-5.922508
C 0	-6.706624	-1.550345	-4.839429
C 0	-5.576993	-1.563434	-4.021216
Cl 0	-5.488021	4.938181	0.003650
H 0	-4.613366	3.586823	-2.048097
H 0	-5.953403	3.397385	2.055634
H 0	-5.604196	0.927204	2.055620
H 0	-4.264162	1.116633	-2.048110
H 0	-4.048595	-0.493082	-2.011971
H 0	-2.843380	-5.096522	-3.648999
H 0	-2.800466	-7.373134	-2.780654
H 0	-3.475074	-7.812586	-0.480634

H 0	-4.246196	-5.983717	0.933535
H 0	-2.962374	-1.422797	-3.256211
H 0	-2.520922	-3.068136	-3.891309
H 0	-3.802838	-4.032356	-5.572418
H 0	-5.823148	-4.008752	-7.036000
H 0	-7.688220	-2.421070	-6.562177
H 0	-7.533115	-0.856878	-4.624919
H 0	-5.504404	-0.867209	-3.172686

SCF Done: E(RB3LYP) = -1835.98731838 A.U. after 14 cycles

Sum of electronic and zero-point Energies= -1836.329823

Sum of electronic and thermal Energies= -1836.308380

Sum of electronic and thermal Enthalpies= -1836.307436

Sum of electronic and thermal Free Energies= -1836.384198

(*Z*)-2-(3-Benzyl)benzo[*d*]thiazol-2(3*H*)-ylidene)-1-(6-chlorpyridin-3-yl)phenylethan-1-one (3u)

N 0	-4.836857	2.999794	-1.145075
C 0	-5.304122	3.638123	0.003703
C 0	-5.576031	2.895298	1.152584
C 0	-5.380777	1.514220	1.152572
C 0	-4.913612	0.875880	0.003789
C 0	-4.641604	1.618715	-1.145087
C 0	-4.724570	-0.461829	0.003798
C 0	-4.272069	-1.080101	-1.108939
O 0	-4.960143	-1.105115	0.998761
C 0	-4.084986	-2.403948	-1.108931
N 0	-3.664743	-3.058731	-2.107636
C 0	-3.578969	-4.292635	-1.837677
C 0	-3.957844	-4.535111	-0.578618
S 0	-4.353027	-3.302749	0.095025
C 0	-3.167813	-5.297895	-2.617402
C 0	-3.140132	-6.546294	-2.139591
C 0	-3.514381	-6.788053	-0.879117
C 0	-3.925547	-5.782836	-0.099340
C 0	-3.321912	-2.465128	-3.408021
C 0	-4.534239	-2.451119	-4.286130
C 0	-4.621007	-3.325749	-5.369174
C 0	-5.750586	-3.312582	-6.187423
C 0	-6.793340	-2.424897	-5.922508
C 0	-6.706624	-1.550345	-4.839429
C 0	-5.576993	-1.563434	-4.021216
Cl 0	-5.488021	4.938181	0.003650
H 0	-5.953403	3.397385	2.055634
H 0	-5.604196	0.927204	2.055620
H 0	-4.264162	1.116634	-2.048111
H 0	-4.048595	-0.493082	-2.011971
H 0	-2.843379	-5.096522	-3.648999
H 0	-2.800466	-7.373134	-2.780654
H 0	-3.475074	-7.812586	-0.480634
H 0	-4.246196	-5.983717	0.933535

H 0	-2.962374	-1.422797	-3.256211
H 0	-2.520922	-3.068136	-3.891309
H 0	-3.802838	-4.032356	-5.572418
H 0	-5.823148	-4.008752	-7.036000
H 0	-7.688220	-2.421070	-6.562177
H 0	-7.533115	-0.856878	-4.624919
H 0	-5.504404	-0.867209	-3.172686

SCF Done: E(RB3LYP) = -1852.02719436 A.U. after 14 cycles

Sum of electronic and zero-point Energies= -1852.384154

Sum of electronic and thermal Energies= -1852.362827

Sum of electronic and thermal Enthalpies= -1852.361883

Sum of electronic and thermal Free Energies= -1852.438725

(*Z*)-2-(3-(4-Brombenzyl)benzo[*d*]thiazol-2(3*H*)-ylidene)-1-(4-(trifluormethyl)phenyl)ethan-1-one (3z)

C 0	-4.836857	2.999794	-1.145075
C 0	-5.304122	3.638123	0.003703
C 0	-5.576031	2.895298	1.152584
C 0	-5.380777	1.514220	1.152572
C 0	-4.913612	0.875880	0.003789
C 0	-4.641604	1.618715	-1.145087
C 0	-4.724570	-0.461829	0.003798
C 0	-4.272069	-1.080101	-1.108939
O 0	-4.960143	-1.105115	0.998761
C 0	-4.084986	-2.403948	-1.108931
N 0	-3.664743	-3.058731	-2.107636
C 0	-3.578969	-4.292635	-1.837677
C 0	-3.957844	-4.535111	-0.578618
S 0	-4.353027	-3.302749	0.095025
C 0	-3.167813	-5.297895	-2.617402
C 0	-3.140132	-6.546294	-2.139591
C 0	-3.514381	-6.788053	-0.879117
C 0	-3.925547	-5.782836	-0.099340
C 0	-3.321912	-2.465128	-3.408021
C 0	-4.534239	-2.451119	-4.286130
C 0	-4.621007	-3.325749	-5.369174
C 0	-5.750586	-3.312582	-6.187423
C 0	-6.793340	-2.424897	-5.922508
C 0	-6.706624	-1.550345	-4.839429
C 0	-5.576993	-1.563434	-4.021216
C 0	-5.488021	4.938181	0.003650
F 0	-4.844947	5.404404	0.783305
F 0	-6.554087	5.162616	0.231467
F 0	-5.209729	5.352732	-0.991071
Br 0	-7.684127	-2.414554	-6.567792
H 0	-4.622329	3.585577	-2.051075
H 0	-5.944444	3.398672	2.058612
H 0	-5.595235	0.928450	2.058597
H 0	-4.273119	1.115346	-2.051088
H 0	-4.057558	- 0.494328	-2.014949
H 0	-2.852314	-5.097856	-3.652025
H 0	-2.809425	-7.374480	-2.783594

H 0	-3.484031	-7.814018	-0.483546
H 0	-4.237256	-5.982357	0.936531
H 0	-2.962374	-1.422797	-3.256211
H 0	-2.520922	-3.068136	-3.891309
H 0	-3.798681	-4.025867	-5.578018
H 0	-5.819024	-4.002262	-7.041623
H 0	-7.529020	-0.850335	-4.630503
H 0	-5.508527	-0.873699	-3.167062

SCF Done: E(RB3LYP) = -1713.30223161 A.U. after 14 cycles

Sum of electronic and zero-point Energies= -1713.833011

Sum of electronic and thermal Energies= -1713.810014

Sum of electronic and thermal Enthalpies= -1713.809069

Sum of electronic and thermal Free Energies= -1713.889374

(*Z*)-4-(2-(3-Benzylbenzo[*d*]thiazol-2(3*H*)-ylidene)acetyl)benzonitrile (3ac)

C 0	-4.836857	2.999794	-1.145075
C 0	-5.304122	3.638123	0.003703
C 0	-5.576031	2.895298	1.152584
C 0	-5.380777	1.514220	1.152572
C 0	-4.913612	0.875880	0.003789
C 0	-4.641604	1.618715	-1.145087
C 0	-4.724570	-0.461829	0.003798
C 0	-4.272069	-1.080101	-1.108939
O 0	-4.960143	-1.105115	0.998761
C 0	-4.084986	-2.403948	-1.108931
N 0	-3.664743	-3.058731	-2.107636
C 0	-3.578969	-4.292635	-1.837677
C 0	-3.957844	-4.535111	-0.578618
S 0	-4.353027	-3.302749	0.095025
C 0	-3.167813	-5.297895	-2.617402
C 0	-3.140132	-6.546294	-2.139591
C 0	-3.514381	-6.788053	-0.879117
C 0	-3.925547	-5.782836	-0.099340
C 0	-3.321912	-2.465128	-3.408021
C 0	-4.534239	-2.451119	-4.286130
C 0	-4.621007	-3.325749	-5.369174
C 0	-5.750586	-3.312582	-6.187423
C 0	-6.793340	-2.424897	-5.922508
C 0	-6.706624	-1.550345	-4.839429
C 0	-5.576993	-1.563434	-4.021216
C 0	-5.488021	4.938181	0.003650
N 0	-5.650212	6.084766	0.003603
H 0	-4.622329	3.585577	-2.051075
H 0	-5.944444	3.398672	2.058612
H 0	-5.595235	0.928450	2.058597
H 0	-4.273119	1.115346	-2.051088
H 0	-4.057558	-0.494328	-2.014949
H 0	-2.852314	-5.097856	-3.652025
H 0	-2.809425	-7.374480	-2.783594
H 0	-3.484031	-7.814018	-0.483546
H 0	-4.237256	-5.982357	0.936531

H 0	-2.962374	-1.422797	-3.256211
H 0	-2.520922	-3.068136	-3.891309
H 0	-3.798681	-4.025867	-5.578018
H 0	-5.819024	-4.002262	-7.041623
H 0	-7.684127	-2.414554	-6.567792
H 0	-7.529020	-0.850335	-4.630503
H 0	-5.508527	-0.873699	-3.167062

SCF Done: E(RB3LYP) = -1468.81450640 A.U. after 14 cycles

Sum of electronic and zero-point Energies= -1468.967763

Sum of electronic and thermal Energies= -1468.945774

Sum of electronic and thermal Enthalpies= -1468.944829

Sum of electronic and thermal Free Energies= -1469.022526

(*Z*)-2-(3-(4-Brombenzyl)benzo[*d*]thiazol-2(3*H*)-ylidene)-1-(4-nitrophenyl)ethan-1-one (3ad)

C 0	-4.836857	2.999794	-1.145075
C 0	-5.304122	3.638123	0.003703
C 0	-5.576031	2.895298	1.152584
C 0	-5.380777	1.514220	1.152572
C 0	-4.913612	0.875880	0.003789
C 0	-4.641604	1.618715	-1.145087
C 0	-4.724570	-0.461829	0.003798
C 0	-4.272069	-1.080101	-1.108939
O 0	-4.960143	-1.105115	0.998761
C 0	-4.084986	-2.403948	-1.108931
N 0	-3.664743	-3.058731	-2.107636
C 0	-3.578969	-4.292635	-1.837677
C 0	-3.957844	-4.535111	-0.578618
S 0	-4.353027	-3.302749	0.095025
C 0	-3.167813	-5.297895	-2.617402
C 0	-3.140132	-6.546294	-2.139591
C 0	-3.514381	-6.788053	-0.879117
C 0	-3.925547	-5.782836	-0.099340
C 0	-3.321912	-2.465128	-3.408021
C 0	-4.534239	-2.451119	-4.286130
C 0	-4.621007	-3.325749	-5.369174
C 0	-5.750586	-3.312582	-6.187423
C 0	-6.793340	-2.424897	-5.922508
C 0	-6.706624	-1.550345	-4.839429
C 0	-5.576993	-1.563434	-4.021216
N 0	-5.574887	5.032864	0.041820
O 0	-4.555755	5.640604	0.551772
O 0	-6.800306	5.430108	0.298211
Br 0	-7.684127	-2.414554	-6.567792
H 0	-4.622329	3.585577	-2.051075
H 0	-5.944444	3.398672	2.058612
H 0	-5.595235	0.928450	2.058597
H 0	-4.273119	1.115346	-2.051088
H 0	-4.057558	-0.494328	-2.014949
H 0	-2.852314	-5.097856	-3.652025
H 0	-2.809425	-7.374480	-2.783594
H 0	-3.484031	-7.814018	-0.483546

H 0	-4.237256	-5.982357	0.936531
H 0	-2.962374	-1.422797	-3.256211
H 0	-2.520922	-3.068136	-3.891309
H 0	-3.798681	-4.025867	-5.578018
H 0	-5.819024	-4.002262	-7.041623
H 0	-7.529020	-0.850335	-4.630503
H 0	-5.508527	-0.873699	-3.167062

SCF Done: E(RB3LYP) = -1581.03846423   A.U. after   18 cycles

Sum of electronic and zero-point Energies=   -1581.254868

Sum of electronic and thermal Energies=   -1581.232067

Sum of electronic and thermal Enthalpies=   -1581.231122

Sum of electronic and thermal Free Energies=   -1581.311636

(*Z*)-2-(3-Benzyl)benzo[*d*]thiazol-2(3*H*)-ylidene)-1-(thiophen-2-yl)ethan-1-one (3ag)

C 0	-5.169690	1.242319	0.077962
C 0	-5.337589	1.996208	-1.062855
C 0	-5.507769	3.380191	-0.768578
C 0	-5.463191	3.629559	0.585665
S 0	-5.217338	2.200714	1.495313
C 0	-4.988657	-0.096497	0.077962
C 0	-4.837456	-0.757699	1.246358
O 0	-4.961982	-0.702387	-0.966763
C 0	-4.858860	-2.094389	1.265665
N 0	-4.724768	-2.788671	2.315783
C 0	-4.783825	-4.025584	2.052506
C 0	-4.970139	-4.228624	0.744213
S 0	-5.047802	-2.961950	0.024361
C 0	-4.698951	-5.068056	2.885344
C 0	-4.795547	-6.313667	2.409223
C 0	-4.986687	-6.516556	1.101704
C 0	-5.071570	-5.474126	0.268814
C 0	-4.526790	-2.232472	3.662017
C 0	-3.062133	-2.068592	3.924566
C 0	-2.414513	-2.920118	4.819542
C 0	-1.049806	-2.767311	5.064244
C 0	-0.332810	-1.763097	4.413882
C 0	-0.980391	-0.911642	3.518847
C 0	-2.345137	-1.064378	3.274204
H 0	-5.340005	1.579134	-2.080717
H 0	-5.657078	4.157508	-1.532440
H 0	-5.572765	4.629747	1.030206
H 0	-4.696736	-0.196314	2.181796
H 0	-4.549648	-4.900925	3.962273
H 0	-4.717750	-7.171542	3.093326
H 0	-5.074122	-7.541154	0.711123
H 0	-5.224858	-5.641202	-0.807563
H 0	-5.028665	-1.241440	3.730897
H 0	-4.963754	-2.923884	4.416856
H 0	-2.979963	-3.712132	5.332358
H 0	-0.539038	-3.438769	5.770120
H 0	0.743409	-1.642632	4.606863

H 0	-0.414901	-0.119727	3.005922
H 0	-2.855915	-0.392869	2.568384

SCF Done: E(RB3LYP) = -1697.33876658 A.U. after 14 cycles

Sum of electronic and zero-point Energies= -1697.495377

Sum of electronic and thermal Energies= -1697.475360

Sum of electronic and thermal Enthalpies= -1697.474416

Sum of electronic and thermal Free Energies= -1697.548587

(*Z*)-2-(3-Benzyl)benzo[*d*]thiazol-2(3*H*)-ylidene)-1-(furan-2-yl)ethan-1-on (3ai)

C 0	-5.169690	1.242319	0.077962
C 0	-5.337589	1.996208	-1.062855
C 0	-5.507769	3.380191	-0.768578
C 0	-5.463191	3.629559	0.585665
O 0	-5.217338	2.200714	1.495313
C 0	-4.988657	-0.096497	0.077962
C 0	-4.837456	-0.757699	1.246358
O 0	-4.961982	-0.702387	-0.966763
C 0	-4.858860	-2.094389	1.265665
N 0	-4.724768	-2.788671	2.315783
C 0	-4.783825	-4.025584	2.052506
C 0	-4.970139	-4.228624	0.744213
S 0	-5.047802	-2.961950	0.024361
C 0	-4.698951	-5.068056	2.885344
C 0	-4.795547	-6.313667	2.409223
C 0	-4.986687	-6.516556	1.101704
C 0	-5.071570	-5.474126	0.268814
C 0	-4.526790	-2.232472	3.662017
C 0	-3.062133	-2.068592	3.924566
C 0	-2.414513	-2.920118	4.819542
C 0	-1.049806	-2.767311	5.064244
C 0	-0.332810	-1.763097	4.413882
C 0	-0.980391	-0.911642	3.518847
C 0	-2.345137	-1.064378	3.274204
H 0	-5.340005	1.579134	-2.080717
H 0	-5.657078	4.157508	-1.532440
H 0	-5.572765	4.629747	1.030206
H 0	-4.696736	-0.196314	2.181796
H 0	-4.549648	-4.900925	3.962273
H 0	-4.717750	-7.171542	3.093326
H 0	-5.074122	-7.541154	0.711123
H 0	-5.224858	-5.641202	-0.807563
H 0	-5.028665	-1.241440	3.730897
H 0	-4.963754	-2.923884	4.416856
H 0	-2.979963	-3.712132	5.332358
H 0	-0.539038	-3.438769	5.770120
H 0	0.743409	-1.642632	4.606863
H 0	-0.414901	-0.119727	3.005922
H 0	-2.855915	-0.392869	2.568384

SCF Done: E(RB3LYP) = -1374.26922937 A.U. after 15 cycles

Sum of electronic and zero-point Energies= -1374.510141

Sum of electronic and thermal Energies= -1374.490719

Sum of electronic and thermal Enthalpies= -1374.489775

Sum of electronic and thermal Free Energies= -1374.561187

8 Literature

[1]: M. Kuhnert-Brandstatter, *Sci. Pharm.* **1966**, *34*, 147-166.

[2]: M. Hesse, H. Meier, B. Zeeh, *Spektroskopische Methoden in der organischen Chemie*, Georg Thieme Verlag, **2005**.

[3]: S. K. Yen, L. L. Koh, F. E. Hahn, H. V. Huynh, T. A. Hor, *Organomet.* **2006**, *25*, 5105-5112.

[4]: A. R. Tyler, A. O. Okoh, C. L. Lawrence, V. C. Jones, C. Moffatt, R. B. Smith, *Eur. J. Med. Chem.* **2013**, *64*, 222-227.

[5]: J. Dason, D. Hagen, C.-Y. Cheung, J. Thomas, S. Yue, , Molecular Probes, INC. - WO2005/56687, **2005**.

[6]: R. Doenges, H. Ruckert, U. Geissler, H. Steppan, *Google Patents*, **1990**.

[7]: C. Reichardt, *Chem. Rev.* **1994**, *94*, 2319-2358.

[8]: a) L. P. Hammett, *J. Am. Chem. Soc.* **1937**, *59*, 96-103; b) Y. Yukawa, Y. Tsuno, *Bull. Chem. Soc. Jap.* **1959**, *32*, 965-971; c) C. G. Swain, E. C. Lupton, *J. Am. Chem. Soc.* **1968**, *90*, 4328-4337; d) C. Hansch, A. Leo, R. W. Taft, *Chem. Rev*. **1991**, *91*, 165-195.

[9]: Gaussian 09, Revision A.02, M. J. Frisch, G. W. Trucks, H. B. Schlegel, G. E. Scuseria, M. A. Robb, J. R. Cheeseman, G. Scalmani, V. Barone, B. Mennucci, G. A. Petersson, H. Nakatsuji, M. Caricato, X. Li, H. P. Hratchian, A. F. Izmaylov, J. Bloino, G. Zheng, J. L. Sonnenberg, M. Hada, M. Ehara, K. Toyota, R. Fukuda, J. Hasegawa, M. Ishida, T. Nakajima, Y. Honda, O. Kitao, H. Nakai, T. Vreven, J. A. Montgomery, Jr. , J. E. Peralta, F. Ogliaro, M. Bearpark, J. J. Heyd, E. Brothers, K. N. Kudin, V. N. Staroverov, R. Kobayashi, J. Normand, K. Raghavachari, A. Rendell, J. C. Burant, S. S. Iyengar, J. Tomasi, M. Cossi, N. Rega, J. M. Millam, M. Klene, J. E. Knox, J. B. Cross, V. Bakken, C. Adamo, J. Jaramillo, R. Gomperts, R. E. Stratmann, O. Yazyev, A. J. Austin, R. Cammi, C. Pomelli, J. W. Ochterski, R. L. Martin, K. Morokuma, V. G. Zakrzewski, G. A. Voth, P. Salvador, J. J. Dannenberg, S. Dapprich, A. D. Daniels, O. Farkas, J. B. Foresman, J. V. Ortiz, J. Cioslowski, D. J. Fox, Gaussian, Inc., Wallingford CT, **2009**.
